# Traceless Rhodium‐Catalyzed Hydroacylation Using Alkyl Aldehydes: The Enantioselective Synthesis of β‐Aryl Ketones

**DOI:** 10.1002/chem.201604035

**Published:** 2016-09-26

**Authors:** Anaïs Bouisseau, Ming Gao, Michael C. Willis

**Affiliations:** ^1^ Department of ChemistryUniversity of Oxford, Chemistry Research Laboratory, Mansfield RoadOxfordOX1 3TAUK

**Keywords:** boronic acid, diene ligands, ketones, rhodium catalysis, tandem reactions

## Abstract

A one‐pot three‐step sequence involving Rh‐catalyzed alkene hydroacylation, sulfide elimination and Rh‐catalyzed aryl boronic acid conjugate addition gave products of traceless chelation‐controlled hydroacylation employing alkyl aldehydes. The stereodefined β‐aryl ketones were obtained in good yields with excellent control of enantioselectivity. Good variation of all three reaction components is possible.

Methods based on C−H functionalization are revolutionizing synthetic chemistry, and are allowing non‐conventional disconnections to become routine in synthesis planning. The synthesis of ketones and enones using alkene and alkyne hydroacylation reactions, respectively, provides powerful illustrations of this concept.^1^ Despite the significant advances that have been achieved in metal‐catalyzed hydroacylation, processes based on the use of rhodium catalysts are limited by competing reductive‐decarbonylation pathways.^2^ The use of chelation‐controlled strategies, with the incorporation of the chelating group on either the aldehyde^3^ or the alkene/alkyne,^4^ has become an established and useful approach to deliver highly efficient transformations that proceed under mild conditions, often with high levels of selectivity.^5^ Although there are a number of examples of metal‐catalyzed intermolecular hydroacylation reactions that do not require the use of a chelate, these processes usually have strict substrate requirements in their own right.^6^ For the benefits accrued from a chelation‐controlled approach, there is also a penalty to pay, in that the additional coordinating group used to control reactivity is also present in the product. One approach to address this limitation has been to develop methods for the in situ removal, or derivatization of these substituents.^7^ Intermolecular hydroacylation methods based on S‐chelating substrates are some of the most general processes reported, with aryl‐, heteroaryl‐, alkenyl‐ and alkyl‐substituted aldehydes all proving to be excellent reaction partners.3k–3n To extend the utility of these methods, our laboratory has shown that alkenyl‐ and aryl‐derived S‐chelating aldehydes are useful substrates for a series of tandem catalytic processes that deliver traceless hydroacylation products (Scheme 1 a).^8^ Herein, we report the development of chemistry that allows S‐chelating *alkyl* aldehydes to give traceless products by a hydroacylation‐conjugate addition sequence, which ultimately replaces the S‐substituent with a stereochemically defined aryl group (Scheme 1 b).

**Scheme 1:**
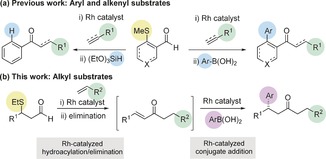
Traceless hydroacylation using *S*‐chelating aldehydes.

To begin our investigation, we established optimal conditions for the three independent steps of our proposed sequence: Rh‐catalyzed alkene hydroacylation, sulfide elimination and Rh‐catalyzed conjugate addition. A catalyst generated in situ from the commercially available precatalyst [Rh(nbd)_2_BF_4_] (nbd=norbornadiene) and the ligand bis(dicyclohexylphosphino)methane (dcpm) proved to be efficient for the combination of 1‐octene and aldehyde **1 a**,^9^ enabling the formation of ketone **2 a** in 99 % yield (Scheme 2, Eq. (1)). Two complementary sets of conditions were identified for the conversion of β‐sulfide **2 a** into enone **3 a**, with the first involving treatment with a mixture of potassium carbonate and methyl trifluoromethanesulfonate, affording the corresponding enone **3 a** in 92 % yield in 1 h at 55 °C; the second method employed copper(I) 3‐methylsalicylate (CuMeSal), which required a longer reaction time, but with the benefit of tolerating a greater range of functional groups [Eq. (2)]. Finally, of the many ligands reported for the rhodium‐catalyzed 1,4‐addition of aryl boronic acids to enones,^10^ catalysts generated from dienes **L1** and **L2** both delivered enantiomerically enriched ketone **4 a** in good yields with excellent selectivities [Eq. (3)].^11^

**Scheme 2:**
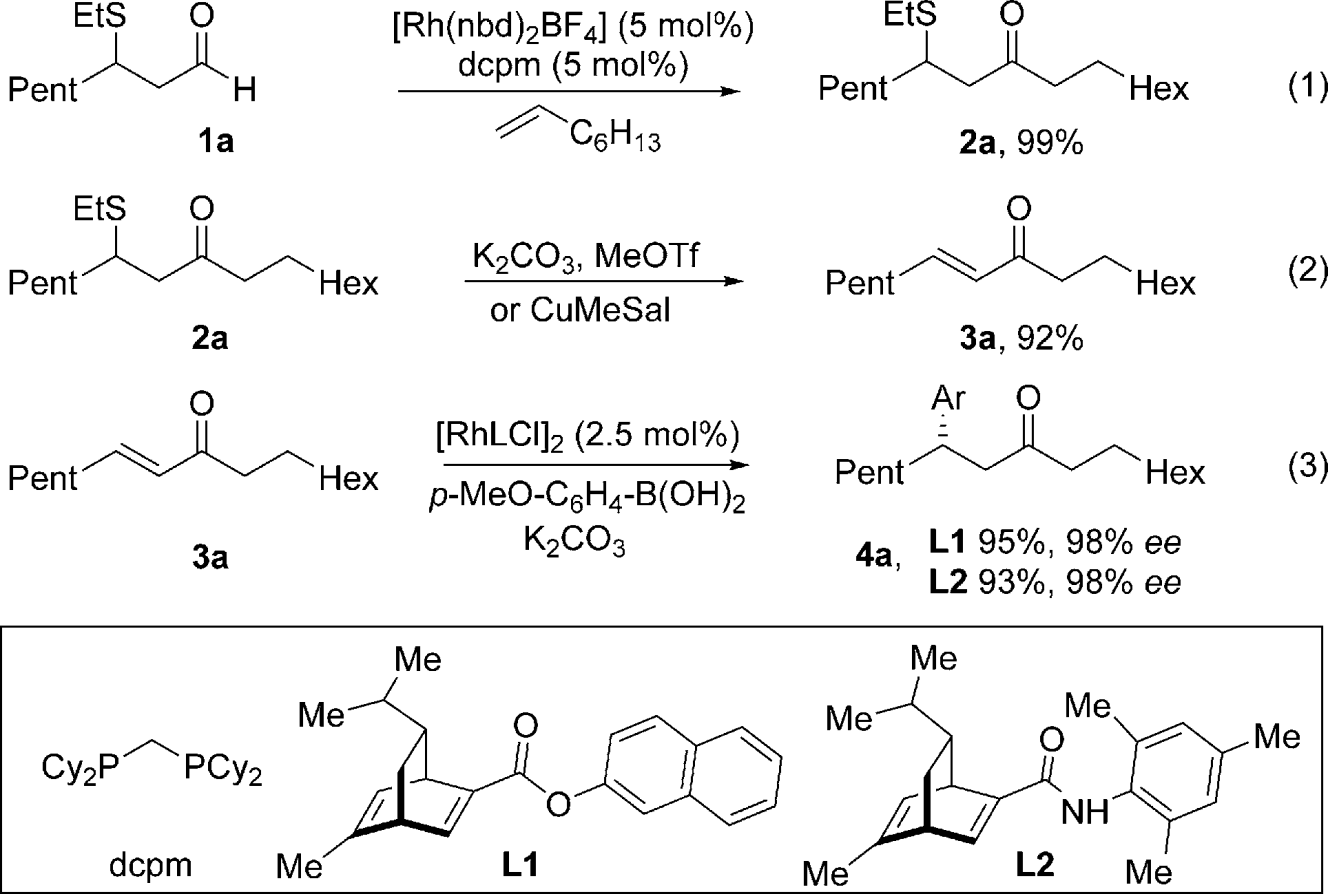
Optimal conditions for the three individual steps towards ketone **4 a**.

We next directed our attention to combining these three individual steps into a one‐pot procedure (Table 1). We initially explored the feasibility of both rhodium catalysts being present from the start of the reaction. However, by using this approach, only a poor yield of the desired ketone could be achieved, even when a high catalyst loading was used (entry 1). Two sequential additions of catalyst proved to be more successful; using a CuMeSal‐mediated elimination, followed by addition of the conjugate addition reagents along with the second rhodium catalyst provided ketone **4 a** in 83 % yield and 98 % *ee* (entry 2). Ligand **L2** was similarly successful (entry 3). Finally, using the MeOTf/K_2_CO_3_ elimination conditions allowed the whole three‐step/one‐pot sequence to be complete in three hours, delivering the ketone product in a yield of 85 % with excellent enantioselectivity (97 % *ee*, entry 4). Given the commercial availability of ligand **L1**, the catalyst incorporating this ligand was the first choice for exploration of reaction scope.

**Table 1:** Optimization of the one‐pot three‐component synthesis of ketone **4 a**.^[a]^

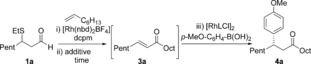						
Entry	Rh [%]^[i]^	Additive [equiv]^[ii]^	*t* (for step^[ii]^)	L [mol %]^[iii]^	Yield [%]	*ee* [%]
1^[b]^	7.5	CuMeSal (1.0)	1 h	L2 (7.5)	29	89
2	5.0	CuMeSal (1.0)	14 h	L1 (5.0)	83	98
3	5.0	CuMeSal (1.0)	14 h	L2 (5.0)	82	98
4	5.0	MeOTf (1.5) K_2_CO_2_ (2.5)	1 h	L1 (5.0)	85	97

[a] Reaction conditions: i) acetone (1.0 m), 55 °C, 1 h; ii) acetone (0.5 m), 55 °C; iii) acetone/MeOH (9:1, 0.1 m), 55 °C, 1 h. [b] Both Rh catalysts added at the start of the reaction.

With the optimal reaction conditions in hand, we set about exploring the scope of the process with respect to the boronic acid component (Table 2). We initially selected aldehyde **1 a** and 1‐octene as the hydroacylation coupling partners, but due to difficulties in the separation of several pairs of enantiomers, preventing the measurement of *ee*, we also employed alternative alkene substrates. The transformation was successfully applied to aryl boronic acids bearing a variety of both electron‐donating (**4 a**–**i**) and electron‐withdrawing (**4 j**–**l**) groups, with substituents being tolerated at all positions of the benzene ring. Notable amongst these examples are the use of free hydroxyl groups (**4 g**,**i**), a basic amine (**4 h**) and reactive ester (**4 k**) and cyano (**4 l**) groups. In addition, heterocyclic and alkenyl boronic acids were shown to be effective coupling partners (**4 m**–**o**).

**Table 2:** Variation of the boronic acid coupling partner.^[a]^

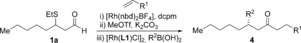		
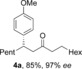	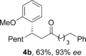	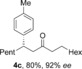
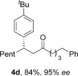	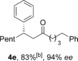	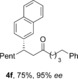
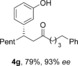	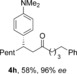	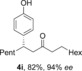
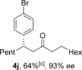	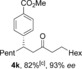	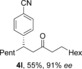
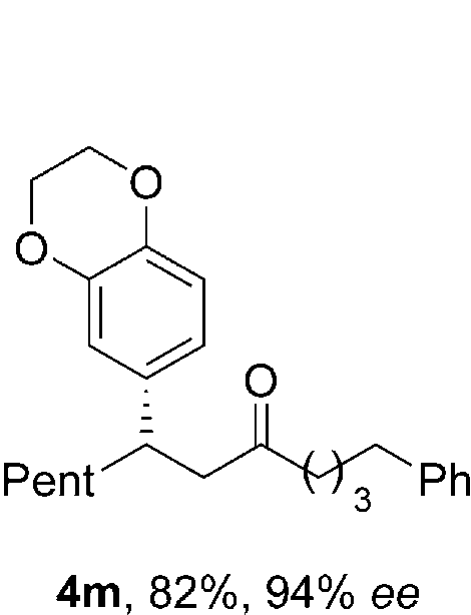	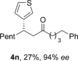	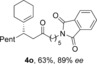

[a] Reaction conditions: i) [Rh(nbd)_2_BF_4_] (5 mol %), dcpm (5 mol %), aldehyde (1.0 equiv), alkene (3.0 equiv), acetone (1 m), 55 °C, 1 h; ii) MeOTf (1.5 equiv), K_2_CO_3_ (2.5 equiv), acetone (0.5 m), 55 °C, 1 h; iii) [Rh(**L1**)Cl]_2_ (2.5 mol %), boronic acid (1.5 equiv), acetone/MeOH (9:1, 0.1 m), 55 °C, 1 h. [b] [Rh(**L2**)Cl]_2_. [c] 1,2‐Dichloroethane (DCE) and [Rh(**L2**)Cl]_2_.

We next examined the scope of the reaction with respect to the alkene substrate, employing aldehyde **1 a** and *para*‐methoxyphenylboronic acid as the two remaining components (Table 3). Alkenes possessing alkyl, aryl or carbonyl groups were well tolerated, giving the corresponding ketone products in good yields and excellent enantioselectivities (**4 p**–**s**). Other useful functional groups, such as an alkyl bromide and a dialkyl acetal, could also be employed (**4 t**,**u**). Given the diverse synthetic utility of halide substituents, we carried out a gram‐scale semi‐preparative reaction using 6‐bromo‐1‐hexene. Starting with 5.5 mmol of aldehyde **1 a**, 1.9 grams of ketone **4 t** was obtained in good yield and *ee* using only 2 mol % and 1 mol % of catalysts for the hydroacylation and conjugate addition steps, respectively. Alkenes containing an acetate or phthalimide group were also excellent substrates (**4 x**,**y**). The use of CuMeSal for the elimination step allowed the use of free hydroxyl group and protected amine containing substrates (**4 v**–**w**).

**Table 3:** Variation of the alkene coupling partner.^[a]^

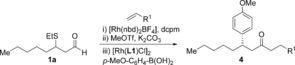		
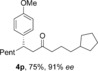	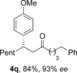	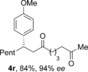
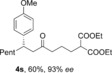	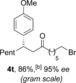	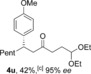
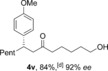		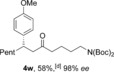
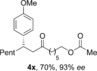		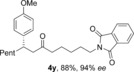

[a] Reaction conditions: See Table 2. [b] i) 2 mol % [Rh(nbd)_2_BF_4_]/dcpm; iii) 1 mol % [Rh(**L2**)Cl]_2_. [c] ArB(OH)_2_ (3 equiv), 14 h. [d] CuMeSal (1.0 equiv).

The aldehyde substrate was the final of the three reaction components to be evaluated for scope (Table 4). A variety of β‐alkyl substituted aldehydes were explored, demonstrating the tolerance of the chemistry to a variety of steric demand (**4 aa**–**ae**). In a similar manner, aryl substituents were also incorporated, giving products with excellent levels of stereocontrol (**4 af**–**ah**). A variety of functional groups, including ethers, esters and phthalimide were well tolerated (**4 ai**–**ak**). An *S*‐citronellal unit could also be introduced, giving *S*,*S*‐ketone **4 al** in good yield with excellent diastereocontrol, and the diastereomeric *S*,*R*‐product could also be obtained by using a Rh^I^/(*S*)‐(−)‐(1,1′‐binaphthalene‐2,2′‐diyl)bis(diphenylphosphine) (*S*‐BINAP) catalyst for the conjugate addition step, although the stereoselectivity was reduced in this case.

**Table 4:** Variation of the aldehyde coupling partner.^[a]^

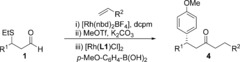		
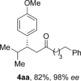	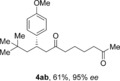	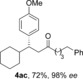
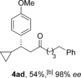	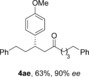	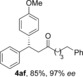
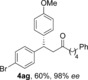	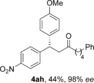	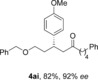
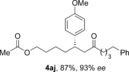		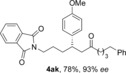
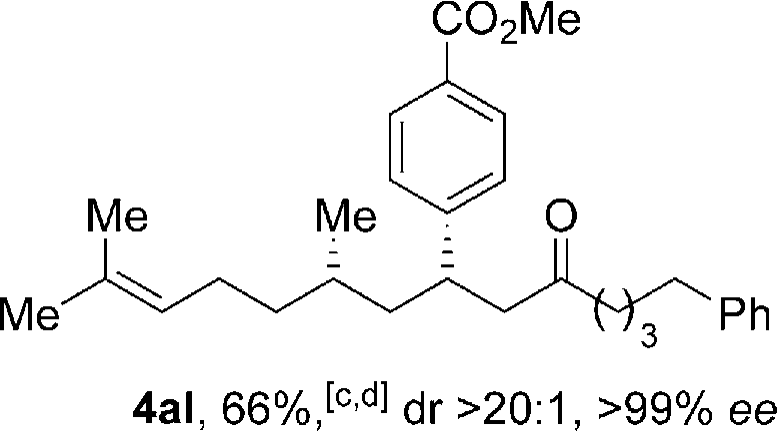		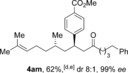

[a] Reaction conditions: See Table 2. [b] CuMeSal (1.0 equiv). [c] DCE and [Rh(**L2**)Cl]_2_. [d] *p*‐MeO_2_C‐C_6_H_4_‐B(OH)_2_ was used. [e] [Rh(nbd)_2_BF_4_]/*S*‐BINAP (5 mol %), 3.0 equiv of boronic acid.

Applying the developed chemistry to alkyne hydroacylation would give a bis‐enone after the elimination step, and we speculated that substrate control could be used to achieve a regioselective conjugate addition to these difunctional intermediates (Table 5). Pleasingly, the introduction of a sterically demanding *tert*‐butyl group on the alkyne completely suppressed conjugate addition at the adjacent carbon, allowing for the regio‐ and enantioselective formation of ketone **5 a** in high yield. Selective mono‐1,4‐addition could also be achieved by utilizing an internal alkyne as a substrate (**5 b**), with the presence of an α‐substituent attenuating nucleophilic addition to the β‐carbon. A double conjugate addition was possible when using cyclohexylacetylene as the hydroacylation coupling partner, giving diarylated ketone **5 c** in a good yield with excellent levels of diastereo‐ and enantiocontrol.

**Table 5:** Application of alkyne hydroacylation to the synthesis of ketones **5**.^[a]^

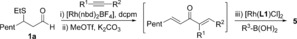		
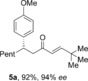	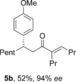	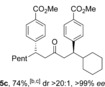

[a] Reaction conditions: See Table 2. [b] ArB(OH)_2_ (3.0 equiv), 3 h. [c] DCE and [Rh(**L2**)Cl]_2_.

With a range of functionalized, enantiomerically enriched ketones available, we next explored the utility of these building blocks for the synthesis of useful heterocyclic structures (Scheme 3). For example, we were able to easily generate tetrahydropyridine **6 a** in an 85 % yield with no loss of enantiopurity, by treating ketone **4 w** with trifluoroacetic acid (TFA) at room temperature. Following a similar strategy and inspired by a previous report on the synthesis of pyrrole derivatives,^12^ we obtained dihydropyrrole **6 b** with excellent stereocontrol. Finally, by employing 2‐hydroxyphenylboronic acid as the conjugate addition nucleophile, we were able to access chromane derivatives;^13^ both ketals **6 c** and **d** were obtained in good yields, the latter was then subjected to reduction conditions, giving *O*‐heterocycle **6 e** in excellent yield and enantioselectivity.

**Scheme 3:**
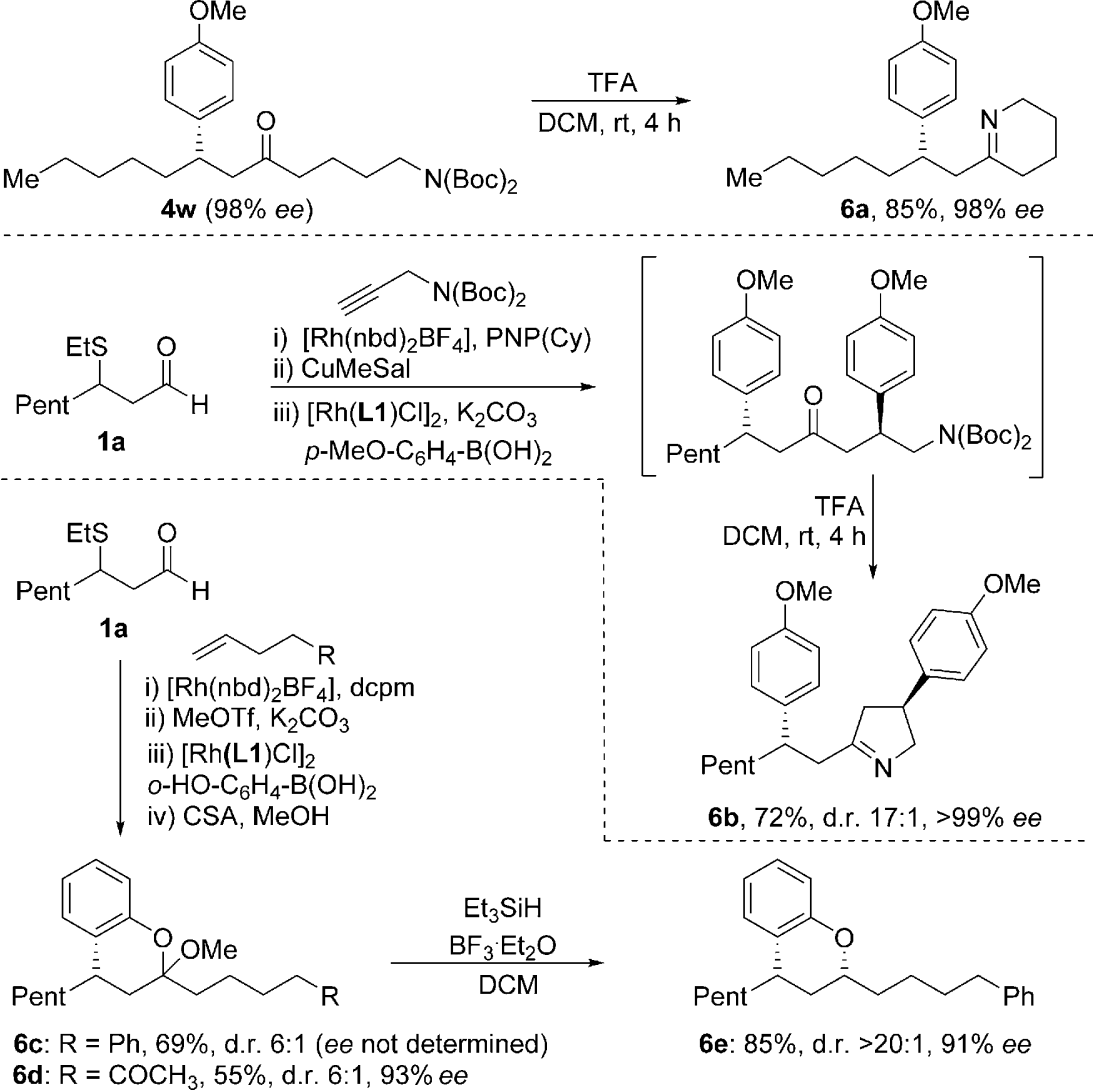
Utility of hydroacylation/conjugate addition products for N‐ and O‐heterocycle synthesis.

In conclusion, we have successfully combined Rh^I^‐catalyzed hydroacylation and conjugate addition into a three‐component one‐pot procedure, leading to highly functionalized ketones with excellent yields and levels of enantiocontrol. The process utilizes the benefits of chelation‐controlled hydroacylation—efficient reactions, good substrate scope, and mild reaction conditions—yet delivers products of traceless hydroacylation. The utility of our methodology was further demonstrated by transforming these products into a variety of useful heterocycle building blocks.

## Supporting information

As a service to our authors and readers, this journal provides supporting information supplied by the authors. Such materials are peer reviewed and may be re‐organized for online delivery, but are not copy‐edited or typeset. Technical support issues arising from supporting information (other than missing files) should be addressed to the authors.

Supplementary
