# Supplementary material for: Traceless Rhodium‐Catalyzed Hydroacylation Using Alkyl Aldehydes: The Enantioselective Synthesis of β‐Aryl Ketones
Source: Chemistry. 2016 Sep 26;22(44):15624–8. doi: 10.1002/chem.201604035 (PMC5396316; doi:10.1002/chem.201604035)
Supplement: Supplementary file 1 — Supplementary [file CHEM-22-15624-s001.pdf]

CHEMISTRY
A European Journal

Supporting Information

Traceless Rhodium-Catalyzed Hydroacylation Using Alkyl Aldehydes: The Enantioselective Synthesis of β-Aryl Ketones

Anaïs Bouisseau+, Ming Gao+, and Michael C. Willis*[a]

chem_201604035_sm_miscellaneous_information.pdf

Supporting Information

Table of Contents

1. **General consideration** **S2**

2. **Starting materials synthesis** **S3**

2.1. Synthesis of alkenes/alkynes S3

2.2. Synthesis of α,β-unsaturated aldehydes S4

2.3. Synthesis of β-S aldehydes for hydroacylation S6

3. **One-pot formation of β-substituted ketones** **S11**

3.1. Boronic acid scope S13

3.2. Alkene scope S20

3.3. Aldehyde scope S25

4. **Product derivatization** **S32**

5. **Determination of the absolute configuration** **S36**

6. **References** **S38**

7. **NMR spectra of novel compounds** **S39**

8. **Chiral HPLC chromatography** **S99**

1. General considerations

Reactions were performed under an inert nitrogen atmosphere (passed through a Drierite® and silica filled drying tube before use) and using anhydrous solvents unless otherwise stated. Glassware was oven-dried (>100 °C), and allowed to cool to room temperature under vacuum once assembled. Cooling to 0 °C was achieved using ice-water bath. Microwave conditions were achieved using a CEM Discover-S microwave synthesizer. Reagents were purchased from Sigma-Aldrich Chemical Co. Ltd., Acros Organics Ltd., Alfa Aesar, Fluorochem Ltd. or Strem Chemicals Inc. and were used as supplied. 1-Octene was distilled before use. 1,2-Dichloroethane was purchased from Rathburn (HPLC grade), distilled over CaH_2 and degassed; acetone was purchased from Fischer (HPLC grade), distilled over Drierite® and degassed. The other solvents were collected fresh from an in-house solvent purification system, which involves passing the solvent through anhydrous alumina columns using an Innovative Technology Inc. PS-400-7 solvent purification system. Reactions were monitored by thin layer chromatography (TLC) using pre-coated aluminium-backed silica plates (Merck Kieselgel 60 F254). Plates were visualised under ultraviolet light (254 nm) followed by staining with $KMnO_4$. Flash column chromatography was carried out using Geduran® Si 60, 40-63 micron silica gel; the compound to be purified was either loaded as oil or pre-absorbed onto silica. Pressure was applied to the column head by hand bellows. Petrol refers to the fraction of light petroleum ether boiling in the range 40-60 °C. Rhodium complexes were prepared using literature methods.[1] 1,1-Dicyclohexyl-*N*-(dicyclohexylphosphino)-*N*-methylphosphinamine (PNP(Cy)) was synthesized according to a reported procedure and was weighed out in air and stored in a Schlenk tube under nitrogen after use.[2] Diastereomeric ratios (dr) were determined by ^{1}H NMR on the crude mixture.

^{1}H NMR spectra were obtained on a Bruker AVIII400 (400 MHz) spectrometer using the residual solvent as an internal standard. ^{13}C NMR spectra were obtained on a Bruker AVIII400 (101 MHz) spectrometer using the residual solvent as an internal standard. Acquisitions were carried out at room temperature unless otherwise stated. Chemical shifts (δ) are reported in parts per million (ppm) from the residual solvent peak and coupling constants (*J*) were given in Hertz (Hz) and rounded to the nearest 0.5 Hz. Proton multiplicity is assigned using the following abbreviations: singlet (s), doublet (d), triplet (t), quartet (q), quintet (p), multiplet (m), broad (br), apparent (app.).

Low resolution ESI mass spectra were recorded on a Waters LCT Premier spectrometer. High resolution mass spectra were recorded either on a Brüker MicroTOF spectrometer under electrospray ionisation conditions (ESI) or on a Micromass LCT under the conditions of field iniosation (FI) by the internal service at Chemistry Research Laboratory, University of Oxford. Samples for mass spectra were prepared as 1 mg/mL solution in MeOH (LRMS, HRMS-ESI) or submitted neat (HRMS-FI). Values quoted are a ratio of mass to charge in Daltons and relative intensities of peaks observed are quoted as a percentage. High resolution values are calculated to four decimal places from the molecular formula, all found within a tolerance of five ppm.

Infrared spectra were recorded as thin films on a Bruker Tensor 27 FT-IR spectrometer. Melting points were determined using a Stuart Scientific Melting Point Apparatus SMP1. Optical rotations were measured on a Schmidt Haensch UniPol L2000 polarimeter. The

enantiomeric excess (ee) of the products was determined by chiral stationary phase HPLC in a Dionex P680 chromatograph with a Dionex UVD170U detector (Daicel Chiralpak AD-H, AS-H, IC, IA-3, ID-3, OD-H, AY-H columns).

2. Starting materials synthesis

2.1. Synthesis of alkenes/alkynes

2-(Pent-4-en-1-yl)isoindoline-1,3-dione (SM1a)

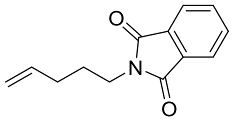

Under N_2 atmosphere, 5-bromo-1-pentene (0.95 mL, 8.0 mmol, 1.0 equiv.) was slowly added to a suspension of phthalimide potassium salt (1.63 g, 8.8 mmol, 1.1 equiv.) in DMF (16 mL, 0.5 M). The resulting mixture was stirred at 60 °C for 24 h. The mixture was cooled to room temperature, poured into a 1:1 mixture of water and brine (40 mL) and extracted with Et_2O (3 × 50 mL). The combined organic layers were washed with 5% LiCl (aq.), dried over $MgSO_4$, filtered and concentrated under reduced pressure. Flash column chromatography (petrol/Et_2O, 85:15) followed by recrystallization (petrol) afforded the alkene **SM1a** (1.28 g, 5.9 mmol, 74%) as a white solid. **M.p.** (petrol): 34-35 °C (lit. m.p. 35-37 °C); $\boldsymbol{\delta}_\mathbf{H}$ (400 MHz, $CDCl_3$): 7.85-7.80 (m, 2H, H_{Ar}), 7.72-7.67 (m, 2H, H_{Ar}), 5.80 (ddt, J = 17.0, 10.0, 6.5 Hz, 1H, C*H*=CH$_2$), 5.07-4.93 (m, 2H, CH=CH_2), 3.71-3.65 (m, 2H, CH_2-N), 2.14-2.06 (m, 2H, CH_2-CH$_2$-N), 1.82-1.72 (m, 2H, CH_2-CH=CH$_2$); $\boldsymbol{\delta}_\mathbf{C}$ (101 MHz, $CDCl_3$): 168.5, 137.4, 134.0, 132.3, 123.3, 115.4, 37.7, 31.1, 27.7; **LRMS** m/z (ESI$^+$) 270.1 ([M+MeOH+Na]$^+$, 100%), 517.2 ([2M+2MeOH+Na]$^+$, 90%).

The data is consistent with the literature.[3]

Di-*tert*-butyl *N*-3-butenyliminodicarboxylate (SM1b)

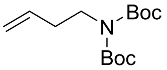

A 100 mL round-bottom flask, fitted with a condenser, was charged with di-*tert*-butyl-iminodicarboxylate (1.30 g, 6.0 mmol, 1.0 equiv.), Cs_2CO_3 (3.91 g, 12.0 mmol, 2.0 equiv.) and LiI (40 mg, 0.3 mmol, 0.05 equiv.). 2-Butanone (30 mL, 0.2 M) was added, followed by 4-bromo-1-butene (0.91 mL, 9.0 mmol, 1.5 equiv.). The resulting mixture was refluxed at 80 °C for 48 h and then cooled to room temperature. Brine (50 mL) was added and the product extracted with Et_2O (3 × 40 mL). The combined organic layers were dried over $MgSO_4$, filtered and concentrated under reduced pressure. Purification by flash column chromatography (petrol/Et_2O, 96:4) afforded the alkene **SM1b** (0.99 g, 3.6 mmol, 61%) as a colorless oil. $\boldsymbol{\delta}_\mathbf{H}$ (400 MHz, $CDCl_3$): 5.76 (ddt, J = 17.0, 10.0, 7.0 Hz, 1H, C*H*=CH$_2$), 5.09-4.98 (m, 2H, CH=CH_2), 3.65-3.59 (m, 2H, CH_2-N), 2.35-2.28 (m, 2H, CH_2-CH$_2$-N), 1.49 (s, 18H, 2 × C(CH_3)$_3$); $\boldsymbol{\delta}_\mathbf{C}$ (101 MHz, $CDCl_3$): 152.7, 135.2, 116.9, 82.3, 45.8, 33.7, 28.2; $\boldsymbol{\nu}_\mathbf{max}$ (film)/cm^{-1} 2980, 1747, 1694, 1367, 1127.

The data is consistent with the literature.[4]

Di-*tert*-butyl prop-2-ynylimidodicarbonate (SM1c)

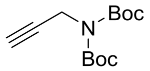

A solution of di-*tert*-butyl dicarbonate (5.24 g, 24.0 mmol, 3.0 equiv.) in MeCN (20 mL) was added to a solution of *N*-Boc-propargylamine (1.24 g, 8.0 mmol, 1.0 equiv.) in MeCN (20 mL). The resulting mixture was stirred at 22 °C for 14 h. The mixture was concentrated under reduced pressure and purified by flash column chromatography (petrol/Et_2O, 92:8) to afford the alkyne **SM1c** (1.89 g, 7.4 mmol, 93%) as a colorless oil. **δ_H** (400 MHz, $CDCl_3$): 4.33 (d, *J* = 2.5 Hz, 2H, C*H₂*), 2.16 (t, *J* = 2.5 Hz, 1H, C*H*), 1.50 (s, 18H, 2 × C(CH_3)$_3$); **δ_C** (101 MHz, $CDCl_3$): 151.7, 83.2, 79.7, 70.6, 35.9, 28.1; **LRMS** m/z (ESI$^+$) 256.1 ([M+H]$^+$, 100%).

The data is consistent with the literature.[5]

2.2. Synthesis of α,β-unsaturated aldehydes

General Procedure A: Synthesis of α,β-unsaturated aldehydes *via* Wittig reactions, as exemplified by the preparation of (*E*)-5-phenylpent-2-enal (SM2a)

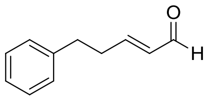

A solution of (triphenylphosphoranylidene)-acetaldehyde (335 mg, 1.1 mmol, 1.1 equiv.) and hydrocinnamaldehyde (0.14 mL, 1.0 mmol, 1.0 equiv.) in $CHCl_3$ (3.3 mL, 0.3 M) was heated at 50 °C for 16 h and then concentrated under reduced pressure. Purification by flash column chromatography (petrol/Et_2O, 9:1) afforded the aldehyde **SM2a** (120 mg, 0.7 mmol, 72%) as a yellow oil. **δ_H** (400 MHz, $CDCl_3$): 9.55 (d, *J* = 8.0 Hz, 1H, C*H*O), 7.47-7.20 (m, 5H, H_{Ar}), 6.92 (dt, *J* = 15.5, 6.5 Hz, 1H, *H*C=CH-CHO), 6.20 (ddt, *J* = 15.5, 8.0, 1.5 Hz, 1H, HC=C*H*-CHO), 2.90 (t, *J* = 7.5 Hz, 2H, Ph-CH_2-CH_2), 2.78-2.69 (m, 2H, CH_2-CH_2-CH); **δ_C** (101 MHz, $CDCl_3$): 194.1, 157.5, 140.4, 133.5, 128.7, 128.4, 126.5, 34.4, 34.2; **LRMS** *m/z* (ESI$^+$) 183.1 ([M+Na]$^+$, 100%).

The data is consistent with the literature.[6]

(*E*)-3-Cyclohexylacrylaldehyde (SM2b)

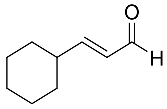

Prepared according to general procedure **A**, using (triphenylphosphoranylidene)-acetaldehyde (3.01 g, 9.9 mmol) and cyclohexanecarboxaldehyde (1.1 mL, 9.0 mmol). The mixture was refluxed with a condenser at 62 °C for 40 h. Purification by flash column chromatography (petrol/Et_2O, 96:4) afforded the aldehyde **SM2b** (258 mg, 1.9 mmol, 21%) as a yellow oil. **δ_H** (400 MHz, $CDCl_3$): 9.49 (d, *J* = 8.0 Hz, 1H, C*H*O), 6.77 (dd, *J* = 15.5, 6.5 Hz, 1H, C*H*=CH-CO), 6.06 (ddd, *J* = 15.5, 8.0, 1.5 Hz, 1H, CH=C*H*-CO), 2.35-2.16 (m, 1H, H_{Cy}), 1.85-1.61 (m, 5H, H_{Cy}), 1.40-1.10 (m, 5H, H_{Cy}); **δ_C** (101 MHz, $CDCl_3$): 194.7, 164.0, 130.7, 41.0, 31.6, 26.0, 25.7; **ν_{max}** (neat)/cm^{-1} 2925, 2852, 1686, 1632, 1449, 1121, 1099, 975.

The data is consistent with the literature.[7]

(*E*)-3-(4-Bromophenyl)acrylaldehyde (SM2c)

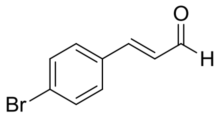

Prepared according to general procedure **A**, using (triphenylphosphoranylidene)-acetaldehyde (1.00 g, 3.3 mmol) and 4-bromobenzaldehyde (0.56 g, 3.0 mmol). Purification by flash column chromatography (petrol/Et_2O, 8:2) afforded the aldehyde **SM2c** (0.35 g, 1.7 mmol, 56%) as a yellow powder. **M.p.** (Et_2O): 80-82 °C (lit. m.p. 80-81 °C); δ_H (400 MHz, $CDCl_3$): 9.70 (d, *J* = 7.5 Hz, 1H, C*H*O), 7.58-7.55 (m, 2H, H_{Ar}), 7.47-7.39 (m, 3H, 2 × H_{Ar} and *H*C=CH-CHO), 6.69 (dd, *J* = 16.0, 7.5 Hz, 1H, HC=C*H*-CHO); δ_C (101 MHz, $CDCl_3$): 193.5, 151.3, 133.0, 132.5, 129.9, 129.1, 125.8; **LRMS** *m/z* (ESI^+) 265.1 ([^{79}BrM+MeOH+Na]$^+$, 50%), 267.1 [^{81}BrM+MeOH+Na]$^+$, 50%).

The data is consistent with the literature.[8]

(*S*,*E*)-5,9-Dimethyldeca-2,8-dienal (SM2d)

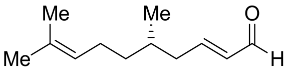

Prepared according to general procedure **A**, using (triphenylphosphoranylidene)-acetaldehyde (2.74 g, 9.0 mmol, 1.5 equiv.) and (*S*)-(-)-citronellal (1.1 mL, 6.0 mmol). The mixture was refluxed with a condenser at 62 °C for 60 h. Purification by flash column chromatography (petrol/Et_2O, 97:3) afforded the *aldehyde* **SM2d** (544 mg, 3.0 mmol, 50%, 97% ee) as a yellow oil. δ_H (400 MHz, $CDCl_3$): 9.49 (d, *J* = 8.0 Hz, 1H, C*H*O), 6.81 (dt, *J* = 15.5, 7.5 Hz, 1H, C*H*=CH-CO), 6.09 (ddt, *J* = 15.5, 8.0, 1.5 Hz, 1H, CH=C*H*-CO), 5.09-5.03 (m, 1H, C*H*=C(CH_3)$_2$), 2.38-2.29 (m, 1H, CH_aH$_b$-CH=CH-CO), 2.21-2.12 (m, 1H, CH$_a$$H_b$-CH=CH-CO), 2.06-1.90 (m, 2H, CH_2-CH=C(CH_3)$_2$), 1.74-1.63 (m, 4H, C*H*-CH_3 and (CH_3)$_a$-C=CH), 1.58 (s, 3H, (CH_3)$_b$-C=CH), 1.40-1.30 (m, 1H, CH_2-CH_cH$_d$-CH-CH_3), 1.25-1.15 (m, 1H, CH_2-CH$_c$$H_d$-CH-$CH_3$), 0.91 (d, *J* = 6.5 Hz, 3H, CH-CH_3); δ_C (101 MHz, $CDCl_3$): 194.1, 157.9, 134.3, 131.7, 124.3, 40.2, 36.8, 32.2, 25.8, 25.5, 19.6, 17.7; **HRMS** (CI^+) $C_{12}H_{21}O$ requires 181.1587 [M+H]$^+$, found 181.1594; ν_{max} (film)/cm^{-1} 2964, 2915, 2730, 1691, 1637, 1454, 1379, 1146; the **ee** was determined by HPLC using a Chiralpak AY-H column (*n*-hexane/*i*-PrOH, 99:1); flow rate 1.0 mL/min; τ_{major} = 7.83 min, τ_{minor} = 6.70 min (97% ee); $[\alpha]_D^{25}$: +9.1 (c = 1.0, $CHCl_3$).

(*E*)-6-(1,3-Dioxoisoindolin-2-yl)hex-2-enal (SM2e)

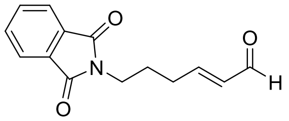

A 50 mL round-bottom flask charged with 2-(pent-4-en-1-yl)isoindoline-1,3-dione **SM1a** (323 mg, 1.5 mmol, 1.0 equiv.) and Hoveyda-Grubbs Catalyst 2nd Generation (47 mg, 0.075 mmol, 0.05 equiv.) (weighed with minimal light) was evacuated and back-filled with N_2 gas. Degassed CH_2Cl_2 (10 mL, 0.15 M) and distilled acrolein (0.30 mL, 4.5 mmol, 3.0 equiv.) were added and the resulting solution was stirred at 22 °C for 20 h. The reaction mixture was filtered through a pad of Celite® and concentrated under reduced pressure. Purification by flash column chromatography (gradient petrol/EtOAc, 9:1 to 1:1) afforded the aldehyde **SM2e** (322 mg, 1.3 mmol, 88%) as a sandy solid. **M.p.**

(EtOAc): 84-86 °C (lit. m.p. 90-91 °C); **δ_H** (400 MHz, $CDCl_3$): 9.46 (d, *J* = 8.0 Hz, 1H, C*H*O), 7.87-7.81 (m, 2H, H_{Ar}), 7.75-7.69 (m, 2H, H_{Ar}), 6.83 (dt, *J* = 15.5, 6.5 Hz, 1H, C*H*=CH-CO), 6.13 (ddt, *J* = 15.5, 8.0, 1.5 Hz, 1H, CH=C*H*-CO), 3.74 (t, *J* = 7.0 Hz, 2H, CH_2-N), 2.44-2.37 (m, 2H, CH_2-CH=CH), 1.95-1.87 (m, 2H, CH_2-CH_2-N); **δ_C** (101 MHz, $CDCl_3$): 193.9, 168.5, 156.7, 134.2, 133.6, 132.1, 123.4, 37.3, 30.1, 26.9; **LRMS** m/z (ESI^+) 266.0 ($[M+ Na]^+$, 100%).

The data is consistent with the literature.[9]

(*E*)-7-Oxohept-5-en-1-yl acetate (SM2f)

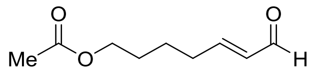

A 50 mL round-bottom flask charged with Hoveyda-Grubbs Catalyst 2nd Generation (47 mg, 0.075 mmol, 0.05 equiv.) (weighed with minimal light) was evacuated and back-filled with N_2 gas. Degassed CH_2Cl_2 (10 mL, 0.15 M), 5-hexenyl acetate (0.24 mL, 1.5 mmol, 1.0 equiv.) and distilled acrolein (0.30 mL, 4.5 mmol, 3.0 equiv.) were added to the flask and the resulting solution was stirred at 22 °C for 20 h. The reaction mixture was filtered through a pad of Celite® and concentrated under reduced pressure. Purification by flash column chromatography (gradient petrol/EtOAc, 9:1 to 7:3) afforded the *aldehyde* **SM2f** (234 mg, 1.4 mmol, 92%) as a pale yellow oil. **δ_H** (400 MHz, $CDCl_3$): 9.48 (d, *J* = 8.0 Hz, 1H, C*H*O), 6.82 (dt, *J* = 15.5, 6.5 Hz, 1H, C*H*=CH-CO), 6.10 (ddt, *J* = 15.5, 8.0, 1.5 Hz, 1H, CH=C*H*-CO), 4.05 (t, *J* = 6.5 Hz, 2H, CO_2-CH_2), 2.39-2.32 (m, 2H, CH_2-CH=CH), 2.02 (s, 3H, CH_3-CO), 1.70-1.61 (m, 2H, CO_2-CH_2-CH_2), 1.61-1.52 (m, 2H, CH_2-CH_2-CH=CH); **δ_C** (101 MHz, $CDCl_3$): 194.0, 171.2, 157.9, 133.3, 64.0, 32.3, 28.2, 24.3, 21.0; **HRMS** (ESI^+) $C_9H_{14}O_3Na$ requires 193.0835 $[M+Na]^+$, found 193.0836; **ν_{max}** (film)/cm^{-1} 2944, 2867, 2740, 1734, 1687, 1638, 1458, 1436, 1388, 1366, 1235, 1137, 1039.

2.3. Synthesis of β-S aldehydes for hydroacylation

General procedure B: Synthesis of β-S aldehydes *via* thia-Michael addition, as exemplified by the preparation of 3-(ethylthio)octanal 6 (1a)

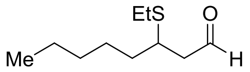

Triethylamine (0.14 mL, 1.0 mmol, 0.2 equiv.) was added to a solution of *trans*-2-octenal (0.75 mL, 5.0 mmol, 1.0 equiv.) and ethanethiol (0.71 mL, 10.0 mmol, 2.0 equiv.) in $CHCl_3$ (2.5 mL, 2.0 M). The resulting mixture was heated under microwave conditions at 110 °C for 1 h and then concentrated under reduced pressure. Purification by flash column chromatography (petrol/Et_2O, 98:2) afforded the aldehyde **1a** (923 mg, 4.9 mmol, 98%) as a colorless oil. **δ_H** (400 MHz, $CDCl_3$): 9.77 (t, *J* = 2.0 Hz, 1H, C*H*O), 3.23-2.98 (m, 1H, C*H*-S), 2.67-2.60 (m, 2H, CH_2-CO), 2.54 (q, *J* = 7.5 Hz, 2H, S-CH_2), 1.64-1.55 (m, 2H, CH-CH_2-CH_2), 1.48-1.26 (m, 6H, 3 × CH_2), 1.24 (t, *J* = 7.5 Hz, 3H, S-CH_2-CH_3), 0.88 (t, *J* = 7.0 Hz, CH_2-CH_2-CH_3); **δ_C** (101 MHz, $CDCl_3$): 201.3, 48.9, 39.4, 35.4, 31.7, 26.6, 24.6, 22.6, 14.9, 14.1; **LRMS** *m/z* (ESI^+) 243.1 ($[M+MeOH+Na]^+$, 100%).

The data is consistent with the literature.[10]

3-(Ethylthio)-4-methylpentanal (1b)

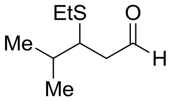

Prepared according to general procedure **B**, using triethylamine (0.11 mL, 0.8 mmol), 4-methyl-2-pentenal (95%, 0.49 mL, 4.0 mmol) and ethanethiol (0.57 mL, 8.0 mmol). Purification by flash column chromatography (petrol/Et_2O, 9:1) afforded the *aldehyde* **1b** (511 mg, 3.2 mmol, 79%) as a colorless oil. **δ_H** (400 MHz, $CDCl_3$): 9.79 (dd, *J* = 2.5, 1.5 Hz, 1H, C*H*O), 3.12-2.93 (m, 1H, C*H*-S), 2.69-2.51 (m, 4H, C*H₂*-CO and S-C*H₂*), 2.01-1.82 (m, 1H, C*H*(CH_3)$_2$), 1.23 (t, *J* = 7.5 Hz, 3H, S-CH_2-C*H₃*), 0.99 (d, *J* = 7.0 Hz, 3H, CH-(C*H₃*)$_a$), 0.97 (d, *J* = 7.0 Hz, 3H, CH-(C*H₃*)$_b$); **δ_C** (101 MHz, $CDCl_3$): 201.5, 46.50, 46.48, 32.5, 26.3, 19.5, 19.4, 14.0; **LRMS** *m/z* (ESI$^+$) 215.1 ([M+MeOH+Na]$^+$, 100%); **HRMS** (ESI$^+$) $C_8H_{16}OSNa$ requires 183.0814 [M+Na]$^+$, found 183.0816; **ν_{max}** (film)/cm^{-1} 2962, 2929, 2872, 1723, 1462, 1284, 1265.

3-(Ethylthio)-5,5-dimethylhexanal (1c)

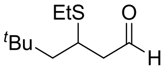

A solution of (triphenylphosphoranylidene)-acetaldehyde (3.01 g, 9.9 mmol) and 3,3-dimethylbutyraldehyde (1.1 mL, 9.0 mmol) in $CHCl_3$ (30 mL) was refluxed with a condenser at 62 °C for 24 h. The mixture was filtered through a pad of SiO_2 [10% Et_2O in petrol (b.p. 30-40 °C)] and concentrated under reduced pressure at room temperature (volatile) to afford a yellow oil (129 mg), which was used in the next step without further purification. The above-prepared liquid (129 mg) was dissolved in $CHCl_3$ (1.0 mL), followed by the addition of ethanethiol (0.15 mL, 2.0 mmol) and triethylamine (0.03 mL, 0.2 mmol). The resulting solution was stirred in a sealed tube at 40 °C for 20 h. Additional ethanethiol (0.07 mL, 1.0 mmol) was added, and the mixture was stirred at 40 °C for a further 40 h. The mixture was concentrated under reduced pressure and purified by flash column chromatography (petrol/Et_2O, 94:6) to afford the *aldehyde* **1c** (162 mg, 0.9 mmol, 10% over two steps) as a colorless oil. **δ_H** (400 MHz, $CDCl_3$): 9.77 (m, 1H, C*H*O), 3.20-3.13 (m, 1H, C*H*-S), 2.71 (ddd, *J* = 17.0, 5.5, 1.5 Hz, 1H, CH-CH_aH_b-CO), 2.64 (ddd, *J* = 17.0, 7.5, 2.5 Hz, 1H, CH-CH_aH_b-CO), 2.54 (q, *J* = 7.5 Hz, 2H, S-C*H₂*-CH_3), 1.63 (dd, *J* = 14.5, 5.5 Hz, 1H, CH_cH_d-CH-CH_2-CO), 1.51 (dd, *J* = 14.5, 6.0 Hz, 1H, CH_cH_d-CH-CH_2-CO), 1.25 (t, *J* = 7.5 Hz, 3H, S-CH_2-C*H₃*), 0.96 (s, 9H, C(C*H₃*)$_3$); **δ_C** (101 MHz, $CDCl_3$): 201.4, 50.7, 49.5, 35.2, 31.5, 29.9, 24.6, 14.6; **LRMS** *m/z* (ESI$^+$) 243.2 ([M+MeOH+Na]$^+$, 100%); **HRMS** (ESI$^+$) $C_{11}H_{24}O_2NaS$ requires 243.1389 [M+MeOH+Na]$^+$, found 243.1389; **ν_{max}** (film)/cm^{-1} 2956, 2869, 1723, 1469, 1366, 1248.

3-Cyclohexyl-3-(ethylthio)propanal (1d)

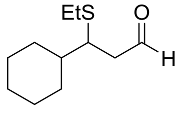

Prepared according to general procedure **B**, using triethylamine (0.04 mL, 0.29 mmol), (*E*)-3-cyclohexylacrylaldehyde **SM2b** (203 mg, 1.47 mmol) and ethanethiol (0.22 mL, 2.94 mmol) in $CHCl_3$ (1.5 mL, 1.0 M). The mixture was stirred in a sealed tube at 40 °C for 20 h. Purification by flash column chromatography (petrol/Et_2O, 94:6) afforded the

aldehyde **1d** (219 mg, 1.09 mmol, 74%) as a yellow oil. **δ$_H$** (400 MHz, $CDCl_3$): 9.78 (dd, *J* = 2.5, 1.5 Hz, 1H, C*H*O), 3.02-2.96 (m, 1H, C*H*-S), 2.72-2.52 (m, 4H, CH-C*H$_2$*-CO and S-C*H$_2$*-CH_3), 1.82-1.62 (m, 5H, *H$_{Cy}$*), 1.57-1.47 (m, 1H, *H$_{Cy}$*), 1.26-1.02 (m, 8H, S-CH_2-C*H$_3$* and *H$_{Cy}$*); **δ$_C$** (101 MHz, $CDCl_3$): 201.7, 46.7, 45.8, 42.6, 30.2, 30.0, 26.49, 26.47, 26.4, 26.3, 14.9; **LRMS** *m/z* (ESI$^+$) 255.1 ([M+MeOH+Na]$^+$, 100%); **HRMS** (ESI$^+$) $C_{12}H_{24}O_2NaS$ requires 255.1389 [M+MeOH+Na]$^+$, found 255.1389; **ν$_{max}$** (film)/cm^{-1} 2925, 2852, 1724, 1449.

3-Cyclopropyl-3-(ethylthio)propanal (1e)

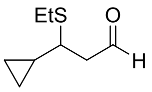

A solution of (triphenylphosphoranylidene)-acetaldehyde (3.01 g, 9.9 mmol) and cyclopropanecarboxaldehyde (0.67 mL, 9.0 mmol) in $CHCl_3$ (30 mL) was refluxed with a condenser at 62 °C for 16 h. The mixture was filtered through a pad of SiO_2 [20% Et_2O in petrol (b.p. 30-40 °C)] and concentrated under reduced pressure at room temperature (volatile) to afford a yellow oil (695 mg), which was used in the next step without further purification. The above-prepared liquid (510 mg) was dissolved in $CHCl_3$ (3 mL), followed by the addition of ethanethiol (0.44 mL, 6.0 mmol) and triethylamine (0.08 mL, 0.6 mmol). The resulting solution was stirred at 22 °C for 72 h. The mixture was concentrated under reduced pressure and purified by flash column chromatography (petrol/Et_2O, 92:8) to afford the *aldehyde* **1e** (176 mg, 1.1 mmol, 17% over two steps) as a colorless oil. **δ$_H$** (400 MHz, $CDCl_3$): 9.80 (t, *J* = 2.0 Hz, 1H, C*H*O), 2.81-2.68 (m, 2H, CH-C*H$_2$*-CO), 2.65 (qd, *J* = 7.5, 1.5 Hz, 2H, S-C*H$_2$*-CH_3), 2.54 (dt, *J* = 9.5, 7.0 Hz, 1H, C*H*-S), 1.25 (t, *J* = 7.5 Hz, 3H, S-CH_2-C*H$_3$*), 0.97 (dtt, *J* = 9.5, 8.0, 5.0 Hz, 1H, C*H*-CH_2-CH_2), 0.71-0.63 (m, 1H, CH-C*H$_a$*H$_b$-CH$_c$H$_d$), 0.60-0.52 (m, 1H, CH-CH$_a$H$_b$-C*H$_c$*H$_d$), 0.42-0.35 (m, 1H, CH-CH$_a$*H$_b$*-CH$_c$H$_d$), 0.26-0.19 (m, 1H, CH-CH$_a$H$_b$-CH$_c$*H$_d$*); **δ$_C$** (101 MHz, $CDCl_3$): 201.0, 49.6, 44.3, 24.9, 17.2, 14.9, 5.9, 4.9; **LRMS** *m/z* (ESI$^+$) 213.1 ([M+MeOH+Na]$^+$, 100%); **HRMS** (ESI$^+$) $C_8H_{14}ONaS$ requires 181.0658 [M+Na]$^+$, found 181.0658; **ν$_{max}$** (film)/cm^{-1} 3003, 2969, 2928, 2872, 2828, 1721, 1452, 1264.

3-(Ethylthio)-5-phenylpentanal (1f)

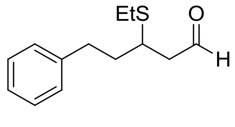

Prepared according to general procedure **B**, using triethylamine (0.02 mL, 0.14 mmol), (*E*)-5-phenylpent-2-enal **SM2a** (112 mg, 0.70 mmol) and ethanethiol (0.10 mL, 1.40 mmol) in $CHCl_3$ (0.4 mL, 1.75 M). Purification by flash column chromatography (petrol/Et_2O, 9:1) afforded the *aldehyde* **1f** (130 mg, 0.58 mmol, 83%) as a pale yellow oil. **δ$_H$** (400 MHz, $CDCl_3$): 9.79 (t, *J* = 2.0 Hz, 1H, C*H*O), 7.38-7.28 (m, 2H, *H$_{Ar}$*), 7.28-7.19 (m, 3H, *H$_{Ar}$*), 3.20-3.13 (m, 1H, C*H*-S), 2.86 (dd, *J* = 14.0, 8.0 Hz, 1H, C*H$_a$*H$_b$-CO), 2.79 (dd, *J* = 14.0, 8.0 Hz, 1H, CH$_a$*H$_b$*-CO), 2.74-2.71 (m, 2H, Ph-C*H$_2$*-CH_2), 2.59 (q, *J* = 7.5 Hz, 2H, S-C*H$_2$*-CH_3), 1.98-1.92 (m, 2H, Ph-CH_2-C*H$_2$*), 1.28 (t, *J* = 7.5 Hz, 3H, S-CH_2-C*H$_3$*); **δ$_C$** (101 MHz, $CDCl_3$): 201.0, 141.4, 128.6, 128.5, 126.2, 49.0, 38.8, 36.9, 33.1, 24.5, 14.9; **LRMS** *m/z* (ESI$^+$) 277.1 ([M+MeOH+Na]$^+$, 100%); **HRMS** (ESI$^+$) $C_{13}H_{19}OS$ requires 223.1151 [M+H]$^+$, found 223.1153; **ν$_{max}$** (film)/cm^{-1} 3026, 2927, 2360, 1721, 1496, 1454, 1265.

3-(Ethylthio)-3-phenylpropanal (1g)

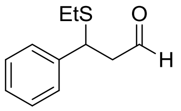

Prepared according to general procedure **B**, using triethylamine (0.11 mL, 0.8 mmol), *trans*-cinnamaldehyde (0.50 mL, 4.0 mmol) and ethanethiol (0.57 mL, 8.0 mmol). The resulting mixture was stirred at 22 °C for 12 h. Purification by flash column chromatography (petrol/CH_2Cl_2, 1:1) afforded the aldehyde **1g** (687 mg, 3.5 mmol, 88%) as a yellow oil. **δ_H** (400 MHz, $CDCl_3$): 9.71 (t, *J* = 2.0 Hz, 1H, C*H*O), 7.38-7.24 (m, 5H, H_{Ar}), 4.38 (t, *J* = 7.5 Hz, 1H, C*H*-S), 3.02-2.97 (m, 1H, CH_aH_b-CO), 2.96-2.91 (m, 1H, CH_aH_b-CO), 2.42-2.28 (m, 2H, S-CH_2), 1.17 (t, *J* = 7.5 Hz, 3H, S-CH_2-CH_3); **δ_C** (101 MHz, $CDCl_3$): 199.6, 141.4, 128.8, 127.7, 127.6, 49.9, 43.0, 25.3, 14.4; **LRMS** *m/z* (ESI^+) 249.1 ($[M+MeOH+Na]^+$, 100%).

The data is consistent with the literature.[11]

3-(4-Bromophenyl)-3-(ethylthio)propanal (1h)

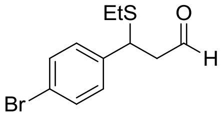

Prepared according to general procedure **B**, using triethylamine (0.04 mL, 0.27 mmol), (*E*)-3-(4-bromophenyl)acrylaldehyde **SM2c** (281 mg, 1.33 mmol) and ethanethiol (0.19 mL, 2.66 mmol). Purification by flash column chromatography (petrol/Et_2O, 85:15) afforded the *aldehyde* **1h** (308 mg, 1.13 mmol, 85%) as a yellow oil. **δ_H** (400 MHz, $CDCl_3$): 9.68 (t, *J* = 1.5 Hz, 1H, C*H*O), 7.48-7.42 (m, 2H, H_{Ar}), 7.25-7.23 (m, 2H, H_{Ar}), 4.33 (t, *J* = 7.5 Hz, 1H, C*H*-S), 2.93 (dd, *J* = 7.5, 1.5 Hz, 2H, CH_2-CO), 2.39-2.25 (m, 2H, S-CH_2-CH_3), 1.16 (t, *J* = 7.5 Hz, 3H, S-CH_2-CH_3); **δ_C** (101 MHz, $CDCl_3$): 199.1, 140.7, 131.9, 129.5, 121.3, 49.8, 42.3, 25.3, 14.4; **LRMS** *m/z* (ESI^+) 327.1 ($[^{79}BrM+MeOH+Na]^+$, 50%), 329.2 ($[^{81}BrM+MeOH+Na]^+$, 50%); **HRMS** (ESI^+) $C_{11}H_{13}OS^{79}BrNa$ requires 294.9763 $[M+Na]^+$, found 294.9764; **ν_{max}** (film)/cm^{-1} 2971, 2928, 2826, 1722, 1487, 1404, 1384, 1315.

3-(Ethylthio)-3-(4-nitrophenyl)propanal (1i)

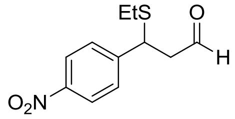

Prepared according to general procedure **B**, using triethylamine (0.06 mL, 0.4 mmol), 4-nitrocinnamaldehyde (354 mg, 2.0 mmol) and ethanethiol (0.29 mL, 4.0 mmol). Purification by flash column chromatography (petrol/CH_2Cl_2, 1:1) afforded the *aldehyde* **1i** (299 mg, 1.25 mmol, 63%) as an orange oil. **δ_H** (400 MHz, $CDCl_3$): 9.70 (t, *J* = 1.0 Hz, 1H, C*H*O), 8.23-8.10 (m, 2H, H_{Ar}), 7.57-7.52 (m, 2H, H_{Ar}), 4.45 (t, *J* = 7.5 Hz, 1H, C*H*-S), 3.08-3.03 (m, 1H, CH_aH_b-CO), 3.01-2.96 (m, 1H, CH_aH_b-CO), 2.41-2.26 (m, 2H, S-CH_2-CH_3), 1.17 (t, *J* = 7.5 Hz, 3H, S-CH_2-CH_3); **δ_C** (101 MHz, $CDCl_3$): 198.2, 149.5, 147.3, 128.9, 124.1, 49.7, 42.1, 25.5, 14.3; **LRMS** *m/z* (ESI^+) 294.1 ($[M+MeOH+Na]^+$, 100%); **HRMS** (ESI^+) $C_{11}H_{13}O_3NSNa$ requires 262.0508 $[M+Na]^+$, found 262.0509; **ν_{max}** (film)/cm^{-1} 2928, 1721, 1597, 1517, 1452, 1343, 1267.

5-(Benzyloxy)-3-(ethylthio)pentanal (1j)

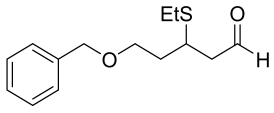

A solution of (triphenylphosphoranylidene)-acetaldehyde (1.00 g, 3.3 mmol) and 3-benzyloxypropionaldehyde (0.47 mL, 3.0 mmol) in $CHCl_3$ (10 mL) was refluxed with a condenser at 62 °C for 60 h. The mixture was concentrated under reduced pressure and purified by flash column chromatography (petrol/Et_2O, 4:1) to afford the enal (contains ~10% unreacted 3-benzyloxypropionaldehyde, not isolable from SiO_2) as a pale yellow oil (355 mg), which was used in the next step without further purification. The above-prepared liquid (355 mg) was dissolved in $CHCl_3$ (1.8 mL), followed by the addition of ethanethiol (0.27 mL, 3.6 mmol) and triethylamine (0.05 mL, 0.36 mmol). The resulting solution was stirred in a sealed tube at 40 °C for 14 h. The mixture was concentrated under reduced pressure and purified by flash column chromatography (gradient petrol/Et_2O, 88:12 to 82:18) to afford the *aldehyde* **1j** (365 mg, 1.4 mmol, 48% over two steps) as a colorless oil. $\boldsymbol{\delta}_H$ (400 MHz, $CDCl_3$): 9.75 (dd, *J* = 2.5, 1.5 Hz, 1H, C*H*O), 7.38-7.25 (m, 5H, H_{Ar}), 4.49 (s, 2H, Ph-CH_2-O), 3.71-3.56 (m, 2H, Ph-CH_2-O-CH_2), 3.39-3.30 (m, 1H, C*H*-S), 2.72 (ddd, *J* = 17.0, 6.0, 1.5 Hz, 1H, CH-C$H_a$$H_b$-CO), 2.64 (ddd, *J* = 17.0, 8.0, 2.5 Hz, 1H, CH-C$H_a$$H_b$-CO), 2.55 (qd, *J* = 7.5, 0.5 Hz, 2H, S-CH_2-CH_3), 1.98-1.83 (m, 2H, CH_2-CH_2-CH), 1.24 (t, *J* = 7.5 Hz, 3H, S-CH_2-CH_3); $\boldsymbol{\delta}_C$ (101 MHz, $CDCl_3$): 201.0, 138.3, 128.5, 127.84, 127.81, 73.2, 67.4, 49.1, 36.6, 35.5, 24.8, 14.9; **LRMS** *m/z* (ESI^+) 307.1 ($[M+MeOH+Na]^+$, 100%); **HRMS** (ESI^+) $C_{14}H_{20}O_2NaS$ requires 275.1076 $[M+Na]^+$, found 275.1078; $\boldsymbol{\nu}_{max}$ (film)/cm^{-1} 3031, 2927, 2861, 2725, 1722, 1454, 1364, 1264.

5-(Ethylthio)-7-oxoheptyl acetate (1k)

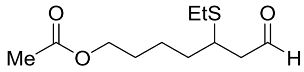

Prepared according to general procedure **B**, using triethylamine (0.03 mL, 0.24 mmol), (*E*)-7-oxohept-5-en-1-yl acetate **SM2f** (207 mg, 1.21 mmol) and ethanethiol (0.18 mL, 2.42 mmol) in $CHCl_3$ (1.2 mL, 1.0 M). The mixture was stirred at 22 °C for 22 h. Purification by flash column chromatography (gradient petrol/EtOAc, 90:10 to 85:15) afforded the *aldehyde* **1k** (202 mg, 0.87 mmol, 72%) as a colorless oil. $\boldsymbol{\delta}_H$ (400 MHz, $CDCl_3$): 9.77 (t, *J* = 2.0 Hz, 1H, C*H*O), 4.05 (t, *J* = 6.5 Hz, 2H, CO_2-CH_2), 3.11 (app. p, *J* = 7.0 Hz, 1H, C*H*-S), 2.66 (dd, *J* = 7.0, 2.0 Hz, 2H, CH-CH_2-CO), 2.54 (q, *J* = 7.5 Hz, 2H, S-CH_2-CH_3), 2.03 (s, 3H, CH_3-CO), 1.67-1.56 (m, 4H, 2 × CH_2), 1.55-1.40 (m, 2H, CH_2), 1.23 (t, *J* = 7.5 Hz, 3H, S-CH_2-CH_3); $\boldsymbol{\delta}_C$ (101 MHz, $CDCl_3$): 201.0, 171.3, 64.3, 49.1, 39.2, 34.9, 28.5, 24.7, 23.4, 21.1, 14.8; **LRMS** *m/z* (ESI^+) 233.0 ($[M+H]^+$, 100%); **HRMS** (ESI^+) $C_{11}H_{21}O_3S$ requires 233.1206 $[M+H]^+$, found 233.1208; $\boldsymbol{\nu}_{max}$ (film)/cm^{-1} 2931, 2868, 2726, 1724, 1455, 1388, 1366, 1236, 1036.

6-(1,3-Dioxoisoindolin-2-yl)-3-(ethylthio)hexanal (1l)

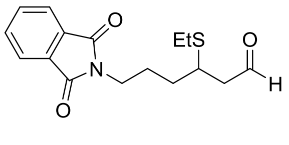

Prepared according to general procedure **B**, using triethylamine (0.03 mL, 0.25 mmol), (*E*)-6-(1,3-dioxoisoindolin-2-yl)hex-2-enal **SM2e** (303 mg, 1.25 mmol) and ethanethiol (0.19 mL, 2.50 mmol) in $CHCl_3$ (1.3 mL, 1.0 M). The mixture was stirred at 22 °C for 22 h. Purification by flash column chromatography (gradient petrol/EtOAc, 9:1 to 8:2) afforded the *aldehyde* **1l** (350 mg, 1.15 mmol, 92%) as a pale yellow oil. δ_H (400 MHz, $CDCl_3$): 9.73 (t, *J* = 2.0 Hz, 1H, C*H*O), 7.85-7.79 (m, 2H, H_{Ar}), 7.73-7.67 (m, 2H, H_{Ar}), 3.69 (t, *J* = 7.0 Hz, 2H, CH_2-N), 3.19-3.11 (m, 1H, C*H*-S), 2.64 (dd, *J* = 7.0, 2.0 Hz, 2H, CH-CH_2-CO), 2.52 (q, *J* = 7.5 Hz, 2H, S-CH_2-CH_3), 1.94-1.74 (m, 2H, CH_2-CH_2-N), 1.68-1.58 (m, 2H, CH_2-CH-CH_2-CO), 1.21 (t, *J* = 7.5 Hz, 3H, S-CH_2-CH_3); δ_C (101 MHz, $CDCl_3$): 200.8, 168.5, 134.1, 132.1, 123.3, 49.1, 38.8, 37.6, 32.4, 26.0, 24.6, 14.8; **LRMS** *m/z* (ESI^+) 360.2 ($[M+MeOH+Na]^+$, 100%); **HRMS** (ESI^+) $C_{17}H_{23}O_4NNaS$ requires 360.1240 $[M+MeOH+Na]^+$, found 360.1231; ν_{max} (film)/cm^{-1} 2929, 2869, 2727, 1771, 1704, 1615, 1437, 1396, 1371, 1361, 1263, 1188, 1052.

(5*S*)-3-(Ethylthio)-5,9-dimethyldec-8-enal (1m)

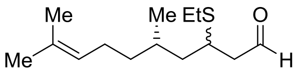

Prepared according to general procedure **B**, using triethylamine (0.04 mL, 0.3 mmol), (*S*,*E*)-5,9-dimethyldeca-2,8-dienal **SM2d** (270 mg, 1.5 mmol) and ethanethiol (0.22 mL, 3.0 mmol) in $CHCl_3$ (1.5 mL, 1.0 M). The mixture was stirred at 22 °C for 24 h. Purification by flash column chromatography (petrol/Et_2O, 97:3) afforded the *aldehyde* **1m** (268 mg, 1.1 mmol, 74%, mixture of diastereoisomers) as a pale yellow oil. δ_H (400 MHz, $CDCl_3$): 9.79-9.76 (m, 1H, C*H*O), 5.11-5.05 (m, 1H, C*H*=C(CH_3)$_2$), 3.22-3.13 (m, 1H, C*H*-S), 2.69-2.49 (m, 4H, CH-CH_2-CO and S-CH_2-CH_3), 2.06-1.88 (m, 2H, CH_2-CH=C(CH_3)$_2$), 1.75-1.10 (m, 14H, CH_2-C*H*(CH_3)-CH_2, 2 × CH_3-C=CH and S-CH_2-CH_3), 0.91 (m, 3H, CH-CH_3); δ_C (101 MHz, $CDCl_3$) (*denotes the second diastereoisomer): 201.4, 201.4*, 131.6, 131.5*, 124.7*, 124.6, 49.8*, 48.8, 43.2, 42.7*, 37.3, 37.2*, 37.0*, 36.8, 30.02, 29.99*, 25.8, 25.8*, 25.5*, 25.4, 24.5, 24.0*, 19.7, 19.4*, 17.8, 17.8*, 14.9, 14.8*; **HRMS** (CI^+) $C_{14}H_{27}OS$ requires 243.1777 $[M+H]^+$, found 243.1778; ν_{max} (film)/cm^{-1} 2965, 2915, 2849, 2725, 1724, 1453, 1378,1265.

3. One-pot formation of β-substituted ketones

General Procedure C: Rh-catalyzed one-pot synthesis of β-substituted ketones from aldehydes, alkenes (or alkynes) and boronic acids, as exemplified by the preparation of (*S*)-6-(4-methoxyphenyl)hexadecan-8-one (4a)

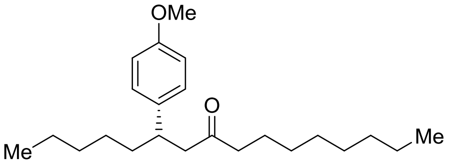

Acetone (~0.5 mL) was added to a microwave reaction vial charged with $Rh(nbd)_2BF_4$ (5.6 mg, 0.015 mmol, 0.05 equiv.) and dcpm (6.1 mg, 0.015 mmol, 0.05 equiv.) (previously evacuated and back-filled with N_2 gas). The solution was bubbled with H_2 gas for 2 min, followed by N_2 gas until dryness. Acetone (0.30 mL, 1.0 M) was added and the resulting solution was transferred to a N_2-purged microwave reaction vial containing 3-(ethylthio)octanal **1a** (56.5 mg, 0.30 mmol, 1.0 equiv.). 1-Octene (0.14 mL, 0.90 mmol, 3.0 equiv.) was added and the reaction mixture was stirred at 55 °C for 1 h.

The mixture was allowed to cool to room temperature and transferred to a N_2-purged microwave reaction vial charged with K_2CO_3 (103.7 mg, 0.75 mmol, 2.5 equiv.). Acetone (additional 0.30 mL, 0.5 M) then MeOTf (51 µL, 0.45 mmol, 1.5 equiv.) were added. The resulting suspension was stirred at 55 °C for 1 h, cooled to room temperature and transferred to a microwave reaction vial charged with 4-methoxyphenylboronic acid (68.4 mg, 0.45 mmol, 1.5 equiv.) and $[Rh(L1)Cl]_2$ (7.1 mg, 0.0075 mmol, 0.025 equiv.) (previously evacuated and back-filled with N_2 gas). Acetone (2.1 mL) and methanol (0.3 mL) (0.1 M) were added and the mixture was stirred at 55 °C for 1 h. Upon completion, the reaction mixture was filtered through a pad of SiO_2 and the filtrate concentrated under reduced pressure. Purification by flash column chromatography (petrol/Et_2O, 99:1) afforded the *ketone* **4a** (88.0 mg, 0.25 mmol, 85%, 97% ee) as a yellow oil. **δ_H** (400 MHz, $CDCl_3$): 7.08 (d, J = 8.5 Hz, 2H, H_{Ar}), 6.81 (d, J = 8.5 Hz, 2H, H_{Ar}), 3.77 (s, 3H, O-CH_3), 3.10-3.03 (m, 1H, CH-CH$_2$-CO), 2.66 (dd, J = 13.5, 5.0 Hz, 1H, CH-CH_aH$_b$-CO), 2.61 (dd, J = 13.5, 4.5 Hz, 1H, CH-CH$_a$$H_b$-CO), 2.31-2.14 (m, 2H, CH$_2$-CH_2-CO), 1.60-1.40 (m, 4H, 2 × CH_2), 1.29-1.08 (m, 16H, 8 × CH_2), 0.88-0.82 (m, 6H, 2 × CH$_2$-CH_3); **δ_C** (101 MHz, $CDCl_3$): 210.7, 158.1, 136.9, 128.5, 113.9, 55.3, 50.4, 43.8, 40.6, 36.7, 31.93, 31.86, 31.8, 29.5, 29.2, 27.2, 23.7, 22.8, 22.6, 14.21, 14.16; **LRMS** *m/z* (ESI$^+$) 369.3 ($[M+Na]^+$, 100%), 715.6 ($[2M+Na]^+$, 50%); **HRMS** (ESI$^+$) $C_{23}H_{38}O_2Na$ requires 369.2764 $[M+Na]^+$, found 369.2763; **ν_{max}** (film)/cm^{-1} 2925, 2855, 1713, 1611, 1512, 1373, 1246; the **ee** was determined by HPLC using a Chiralpak AD-H column (*n*-hexane/*i*-PrOH, 99:1); flow rate 1.0 mL/min; τ_{major} = 9.09 min, τ_{minor} = 9.58 min (97% ee); $[\alpha]_D^{25}$: +20.0 (c = 1.0, $CHCl_3$).

When 1,2-dichloroethane (DCE) is mentioned, it indicates that DCE was used instead of acetone for the whole process (volumes not modified).

When $[Rh(L2)Cl]_2$ is mentioned, it indicates that $[Rh(L2)Cl]_2$ was used instead of $[Rh(L1)Cl]_2$ for the conjugate addition step.

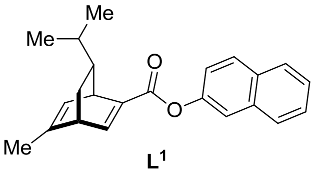

L¹

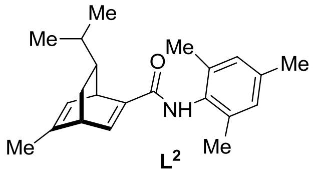

L²

General Procedure D: Rh-catalyzed one-pot synthesis of β-substituted ketones from aldehydes, alkenes and boronic acids, employing CuMeSal for the elimination step, as exemplified by the preparation of (*S*)-1-hydroxy-8-(4-methoxyphenyl)tridecan-6-one (4v)

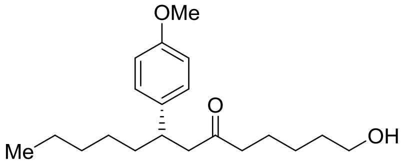

Acetone (~0.5 mL) was added to a microwave reaction vial charged with $Rh(nbd)_2BF_4$ (5.6 mg, 0.015 mmol, 0.05 equiv.) and dcpm (6.1 mg, 0.015 mmol, 0.05 equiv.) (previously evacuated and back-filled with N_2 gas). The solution was bubbled with H_2 gas for 2 min, followed by N_2 gas until dryness. Acetone (0.30 mL, 1.0 M) was added and the resulting solution was transferred to a N_2-purged microwave reaction vial

containing 3-(ethylthio)octanal **1a** (56.5 mg, 0.30 mmol, 1.0 equiv.). 4-Penten-1-ol (93 μL, 0.90 mmol, 3.0 equiv.) was added and the reaction mixture was stirred at 55 °C for 1 h. The mixture was allowed to cool to room temperature and transferred to a N_2-purged microwave reaction vial charged with CuMeSal (64.4 mg, 0.30 mmol, 1.0 equiv.). Acetone (additional 0.30 mL, 0.5 M) was added and the resulting suspension was stirred at 55 °C for 14 h, cooled to room temperature and filtered (through a thin Celite® layer under inert atmosphere) into a microwave reaction vial charged with 4-methoxyphenylboronic acid (91.2 mg, 0.60 mmol, 2.0 equiv.), K_2CO_3 (41.5 mg, 0.30 mmol, 1.0 equiv.) and $[Rh(L1)Cl]_2$ (7.1 mg, 0.0075 mmol, 0.025 equiv.) (previously evacuated and back-filled with N_2 gas). Acetone (2.1 mL) and methanol (0.3 mL) (0.1 M) were added and the mixture was stirred at 55 °C for 1 h. Upon completion, the reaction mixture was filtered through a pad of SiO_2 and the filtrate concentrated under reduced pressure. Purification by flash column chromatography (petrol/Et_2O, 1:1) afforded the *ketone* **4v** (80.7 mg, 0.25 mmol, 84%, 92% ee) as a yellow oil. $\boldsymbol{\delta_H}$ (400 MHz, $CDCl_3$): 7.07 (d, *J* = 8.5 Hz, 2H, H_{Ar}), 6.81 (d, *J* = 8.5 Hz, 2H, H_{Ar}), 3.77 (s, 3H, OCH_3), 3.57 (t, *J* = 6.5 Hz, 2H, CH_2-OH), 3.10-3.00 (m, 1H, C*H*-CH_2-CO), 2.65 (dd, *J* = 14.5, 6.5 Hz, 1H, CH-C$H_a$$H_b$-CO), 2.60 (dd, *J* = 14.5, 5.5 Hz, 1H, CH-C$H_a$$H_b$-CO), 2.29 (dt, *J* = 17.0, 7.5 Hz, 1H, CH_2-C$H_c$$H_d$-CO), 2.19 (dt, *J* = 17.0, 7.5 Hz, 1H, CH_2-C$H_c$$H_d$-CO), 1.61-1.43 (m, 6H, 3 × CH_2), 1.26-1.05 (m, 8H, 4 × CH_2), 0.81 (t, *J* = 7.0 Hz, 3H, CH_2-CH_3); $\boldsymbol{\delta_C}$ (101 MHz, $CDCl_3$): 210.6, 158.0, 136.8, 128.5, 113.9, 62.8, 55.3, 50.5, 43.6, 40.7, 36.7, 32.5, 31.8, 27.2, 25.3, 23.2, 22.6, 14.2; **LRMS** m/z (ESI^+) 321.3 ($[M+H]^+$, 20%), 343.2 ($[M+Na]^+$, 100%); **HRMS** (ESI^+) $C_{20}H_{32}O_3Na$ requires 343.2244 $[M+Na]^+$, found 343.2243; $\boldsymbol{\nu_{max}}$ (film)/cm^{-1} 3397 (br), 2928, 2858, 1710, 1611, 1513, 1462, 1247, 1178, 1038; the **ee** was determined by HPLC using a Chiralpak IC column (*n*-hexane/*i*-PrOH, 95:5); flow rate 1.0 mL/min; τ_{major} = 44.76 min, τ_{minor} = 58.30 min (92% ee); $[\alpha]_D^{25}$: +24.5 (c = 1.0, $CHCl_3$).

3.1. Boronic acid scope

(*S*)-7-(2-Methoxyphenyl)-1-phenyldodecan-5-one (4b)

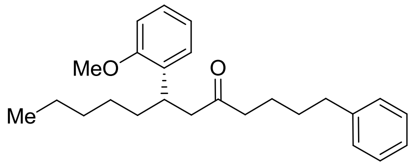

Prepared according to general procedure **C,** using 3-(ethylthio)octanal **1a** (56 mg, 0.30 mmol), 4-phenyl-1-butene (0.14 mL, 0.90 mmol) and 2-methoxyphenylboronic acid (91 mg, 0.60 mmol, 2.0 equiv.). Purification by flash column chromatography (petrol/Et_2O, 98:2) afforded the *ketone* **4b** (70 mg, 0.19 mmol, 63%, 93% ee) as a colorless oil. $\boldsymbol{\delta_H}$ (400 MHz, $CDCl_3$): 7.28-7.24 (m, 2H, H_{Ar}), 7.18-7.09 (m, 5H, H_{Ar}), 6.89 (td, *J* = 7.5, 1.0 Hz, 1H, H_{Ar}), 6.82 (dd, *J* = 8.0, 1.0 Hz, 1H, H_{Ar}), 3.79 (s, 3H, O-CH_3), 3.58-3.51 (m, 1H, C*H*-CH_2-CO), 2.71 (dd, *J* = 15.5, 7.0 Hz, 1H, CH-C$H_a$$H_b$-CO), 2.63 (dd, *J* = 15.5, 7.5 Hz, 1H, CH-C$H_a$$H_b$-CO), 2.55 (t, *J* = 7.0 Hz, 2H, CH_2-Ph), 2.34-2.29 (m, 2H, CH_2-CH_2-CO), 1.63-1.43 (m, 6H, 3 × CH_2), 1.26-1.10 (m, 6H, 3 × CH_2), 0.82 (t, *J* = 7.0 Hz, 3H, CH_2-CH_3); $\boldsymbol{\delta_C}$ (101 MHz, $CDCl_3$): 210.7, 157.4, 142.4, 132.7, 128.5, 128.4, 128.1, 127.2, 125.8, 120.7, 110.8, 55.4, 49.0, 42.9, 35.8, 35.0, 34.9, 31.9, 31.1, 27.2, 23.4, 22.7, 14.2; **LRMS** *m/z* (ESI^+) 367.3 ($[M+H]^+$, 40%), 389.3 ($[M+Na]^+$, 100%); **HRMS** (ESI^+) $C_{25}H_{32}O_2$ requires 367.2632 $[M+H]^+$, found 367.2635; $\boldsymbol{\nu_{max}}$ (film)/cm^{-1} 2927, 2857, 1711, 1600, 1492, 1455, 1438, 1369, 1240; the **ee** was determined by HPLC using a Chiralpak AD-H column (*n*-

hexane/*i*-PrOH, 99:1); flow rate 1.0 mL/min; τ_{major} = 10.27 min, τ_{minor} = 10.95 min (93% ee); $[\alpha]_D^{25}$: +7.8 (*c* = 1.0, $CHCl_3$).

(*S*)-6-(*p*-Tolyl)hexadecan-8-one 1b (4c)

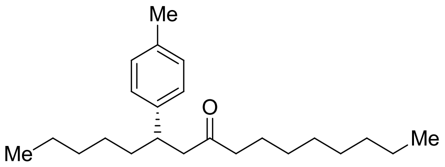

Prepared according to general procedure **C**, using 3-(ethylthio)octanal **1a** (56 mg, 0.30 mmol), 1-octene (0.14 mL, 0.90 mmol) and *p*-tolylboronic acid (61 mg, 0.45 mmol). Purification by flash column chromatography (petrol/Et_2O, 98:2) afforded the *ketone* **4c** (79 mg, 0.24 mmol, 80%, 92% ee) as a yellow oil. **δ_H** (400 MHz, $CDCl_3$): 7.09-7.04 (m, 4H, H_{Ar}), 3.12-3.04 (m, 1H, C*H*-CH_2-CO), 2.67 (dd, *J* = 13.5, 5.0 Hz, 1H, CH-C$H_a$$H_b$-CO), 2.62 (dd, *J* = 13.5, 4.5 Hz, 1H, CH-$CH_a$$H_b$-CO), 2.31 (s, 3H, ArC$H_3$), 2.28-2.16 (m, 2H, CH_2-CH_2-CO), 1.60-1.41 (m, 4H, 2 × CH_2), 1.28-1.09 (m, 16H, 8 × CH_2), 0.87 (t, *J* = 7.0 Hz, 3H, CH_2-CH_3), 0.82 (t, *J* = 7.0 Hz, 3H, CH_2-CH_3); **δ_C** (101 MHz, $CDCl_3$): 210.7, 141.8, 135.8, 129.2, 127.5, 50.3, 43.7, 41.0, 36.6, 32.0, 31.9, 29.5, 29.4, 29.3, 27.2, 23.7, 22.8, 22.6, 21.1, 14.24, 14.19; **LRMS** *m/z* (ESI^+) 331.3 ($[M+H]^+$, 80%), 353.3 ($[M+Na]^+$, 100%); **HRMS** (ESI^+) $C_{23}H_{39}O$ requires 331.2995 $[M+H]^+$, found 331.2996; **ν_{max}** (film)/cm^{-1} 2955, 2924, 2855, 1714, 1514, 1458, 1313; the **ee** was determined by HPLC using a Chiralpak AD-H column (*n*-hexane/*i*-PrOH, 99:1); flow rate 1.0 mL/min; τ_{major} = 4.87 min, τ_{minor} = 5.29 min (92% ee); $[\alpha]_D^{25}$: +18.0 (*c* = 1.0, $CHCl_3$).

(*S*)-7-[4-(*tert*-Butyl)phenyl]-1-phenyldodecan-5-one (4d)

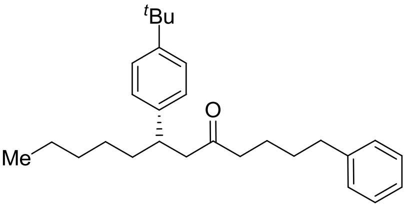

Prepared according to general procedure **C**, using 3-(ethylthio)octanal **1a** (56 mg, 0.30 mmol), 4-phenyl-1-butene (0.14 mL, 0.90 mmol) and 4-(*tert*-butyl)phenylboronic acid (80 mg, 0.45 mmol). Purification by flash column chromatography (petrol/Et_2O, 99:1) afforded the *ketone* **4d** (98 mg, 0.25 mmol, 84%, 95% ee) as a colorless oil. **δ_H** (400 MHz, $CDCl_3$): 7.27-7.21 (m, 4H, H_{Ar}), 7.16-7.13 (m, 1H, H_{Ar}), 7.11-7.09 (m, 2H, H_{Ar}), 7.05 (d, *J* = 8.5 Hz, 2H, H_{Ar}), 3.09-3.02 (m, 1H, C*H*-CH_2-CO), 2.64 (dd, *J* = 13.5, 5.0 Hz, 1H, CH-C$H_a$$H_b$-CO), 2.59 (dd, *J* = 13.5, 5.0 Hz, 1H, CH-$CH_a$$H_b$-CO), 2.51 (br t, *J* = 7.0 Hz, 2H, CH_2-Ph), 2.36-2.15 (m, 2H, CH_2-CH_2-CO), 1.59-1.44 (m, 6H, 3 × CH_2), 1.26 (s, 9H, $(CH_3)_3$), 1.22-1.07 (m, 6H, 3 × CH_2), 0.80 (t, *J* = 7.0 Hz, 3H, CH_2-CH_3); **δ_C** (101 MHz, $CDCl_3$): 210.5, 149.0, 142.4, 141.7, 128.5, 128.4, 127.2, 125.8, 125.4, 50.3, 43.4, 40.9, 36.5, 35.8, 34.5, 31.9, 31.5, 31.0, 27.3, 23.3, 22.7, 14.2; **LRMS** *m/z* (ESI^+) 393.3 ($[M+H]^+$, 20%), 415.3 ($[M+Na]^+$, 100%); **HRMS** (ESI^+) $C_{28}H_{41}O$ requires 393.3152 $[M+H]^+$, found 393.3151; **ν_{max}** (film)/cm^{-1} 2954, 2928, 2858, 1713, 1510, 1455, 1362, 1268; the **ee** was determined by HPLC using a Chiralpak AD-H column (*n*-hexane/*i*-PrOH, 99:1); flow rate 1.0 mL/min; τ_{major} = 7.07 min, τ_{minor} = 7.65 min (95% ee); $[\alpha]_D^{25}$: +18.5 (*c* = 1.0, $CHCl_3$).

(*S*)-1,7-Diphenyldodecan-5-one (4e)

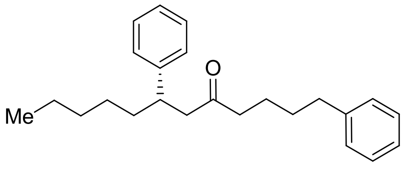

Prepared according to general procedure **C**, using 3-(ethylthio)octanal **1a** (38 mg, 0.20 mmol), 4-phenyl-1-butene (0.09 mL, 0.60 mmol), phenylboronic acid (49 mg, 0.40 mmol, 2.0 equiv.) and $[Rh(L2)Cl]_2$ (4.6 mg, 0.005 mmol). Purification by flash column chromatography (petrol/Et_2O, 99:1) afforded the *ketone* **4e** (56 mg, 0.17 mmol, 83%, 94% ee) as a colorless oil. **δ_H** (400 MHz, $CDCl_3$): 7.30-7.22 (m, 4H, H_{Ar}), 7.19-7.10 (m, 6H, H_{Ar}), 3.20-3.01 (m, 1H, C*H*-CH_2-CO), 2.68 (dd, *J* = 15.0, 6.5 Hz, 1H, CH-C$H_a$$H_b$-CO), 2.62 (dd, *J* = 15.0, 6.0 Hz, 1H, $CH_a$$H_b$-CO), 2.53 (t, *J* = 7.0 Hz, 2H, CH_2-Ph), 2.37-2.15 (m, 2H, CH_2-CH_2-CO), 1.61-1.43 (m, 6H, 3 × CH_2), 1.25-1.07 (m, 6H, 3 × CH_2), 0.81 (t, *J* = 7.0 Hz, 3H, CH_2-CH_3); **δ_C** (101 MHz, $CDCl_3$): 210.2, 144.9, 142.4, 128.6, 128.5, 128.4, 127.6, 126.4, 125.9, 50.2, 43.5, 41.4, 36.5, 35.8, 31.9, 31.0, 27.2, 23.3, 22.6, 14.2; **LRMS** *m/z* (ESI^+) 359.2 ($[M+Na]^+$, 100%); **HRMS** (ESI^+) $C_{24}H_{32}ONa$ requires 359.2345 $[M+Na]^+$, found 359.2346; **ν_{max}** (film)/cm^{-1} 3027, 2927, 2857, 1713, 1453, 1371; the **ee** was determined by HPLC using a Chiralpak IA-3 column (*n*-hexane/*i*-PrOH, 98:2); flow rate 0.5 mL/min; τ_{major} = 11.14 min, τ_{minor} = 11.66 min (94% ee); $[\alpha]_D^{25}$: +17.9 (*c* = 0.5, $CHCl_3$).

(*S*)-7-(Naphthalen-2-yl)-1-phenyldodecan-5-one (4f)

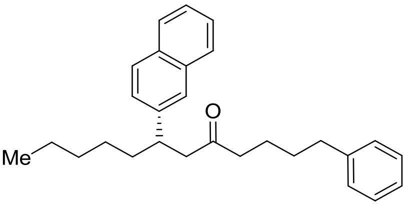

Prepared according to general procedure **C**, using 3-(ethylthio)octanal **1a** (56 mg, 0.30 mmol), 4-phenyl-1-butene (0.14 mL, 0.90 mmol) and 2-naphtaleneboronic acid (77 mg, 0.45 mmol). Purification by flash column chromatography (petrol/Et_2O, 98:2) afforded the *ketone* **4f** (87 mg, 0.23 mmol, 75%, 95% ee) as a colorless oil. **δ_H** (400 MHz, $CDCl_3$): 7.63-7.59 (m, 3H, H_{Ar}), 7.42 (s, 1H, H_{Ar}), 7.30-7.23 (m, 2H, H_{Ar}), 7.15 (dd, *J* = 8.5, 1.5 Hz, 1H, H_{Ar}), 7.08-7.04 (m, 2H, H_{Ar}), 6.99-6.96 (m, 1H, H_{Ar}), 6.89 (d, *J* = 7.0 Hz, 2H, H_{Ar}), 3.16-3.09 (m, 1H, C*H*-CH_2-CO), 2.61 (dd, *J* = 16.0, 7.5 Hz, 1H, CH-C$H_a$$H_b$-CO), 2.54 (dd, *J* = 16.0, 6.5 Hz, 1H, CH-$CH_a$$H_b$-CO), 2.31 (t, *J* = 7.0 Hz, 2H, CH_2-Ph), 2.18-2.00 (m, 2H, CH_2-CH_2-CO), 1.51-1.46 (m, 2H, CH-CH_2-CH_2), 1.35-1.24 (m, 4H, 2 × CH_2), 1.10-0.93 (m, 6H, 3 × CH_2), 0.64 (t, *J* = 6.5 Hz, 3H, CH_2-CH_3); **δ_C** (101 MHz, $CDCl_3$): 210.1, 142.30, 142.26, 133.6, 132.4, 128.5, 128.4, 128.3, 127.74, 127.71, 126.3, 126.1, 125.9, 125.8, 125.4, 50.2, 43.5, 41.5, 36.4, 35.8, 31.9, 30.9, 27.3 23.3, 22.6, 14.2; **LRMS** *m/z* (ESI^+) 387.3 ($[M+H]^+$, 30%), 409.3 ($[M+Na]^+$, 100%); **HRMS** (ESI^+) $C_{28}H_{34}ONa$ requires 409.2502 $[M+Na]^+$, found 409.2500; **ν_{max}** (film)/cm^{-1} 2927, 2856, 2360, 2341, 1711, 1454; the **ee** was determined by HPLC using a Chiralpak AD-H column (*n*-hexane/*i*-PrOH, 98:2); flow rate 1.0 mL/min; τ_{major} = 8.87 min, τ_{minor} = 9.80 min (95% ee); $[\alpha]_D^{25}$: +19.0 (*c* = 1.0, $CHCl_3$).

(*S*)-7-(3-Hydroxyphenyl)-1-phenyldodecan-5-one (4g)

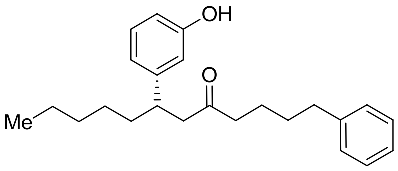

Prepared according to general procedure **C,** using 3-(ethylthio)octanal **1a** (56 mg, 0.30 mmol), 4-phenyl-1-butene (0.14 mL, 0.90 mmol) and 3-hydroxyphenylboronic acid (83 mg, 0.60 mmol, 2.0 equiv.). Purification by flash column chromatography (petrol/Et_2O, 9:1) afforded the *ketone* **4g** (83 mg, 0.24 mmol, 79%, 93% ee) as a yellow oil. **δ_H** (400 MHz, $CDCl_3$): 7.26-7.22 (m, 2H, H_{Ar}), 7.13-7.04 (m, 4H, H_{Ar}), 6.65 (d, *J* = 7.5 Hz, 1H, H_{Ar}), 6.59-6.56 (m, 2H, H_{Ar}), 4.92 (s, 1H, O*H*), 3.02-2.95 (m, 1H, C*H*-CH_2-CO), 2.60 (dd, *J* = 16.0, 7.5 Hz, 1H, CH-CH_aH_b-CO), 2.54 (dd, *J* = 16.0, 7.0 Hz, 1H, CH-CH_aH_b-CO), 2.47 (t, *J* = 7.0 Hz, 2H, CH_2-Ph), 2.27-2.14 (m, 2H, CH_2-CH_2-CO), 1.50-1.40 (m, 6H, 3 × CH_2), 1.18-1.03 (m, 6H, 3 × CH_2), 0.75 (t, *J* = 7.0 Hz, 3H, CH_2-CH_3); **δ_C** (101 MHz, $CDCl_3$): 211.2, 155.9, 146.7, 142.3, 129.7, 128.5, 128.4, 125.8, 119.7, 114.7, 113.4, 50.1, 43.6, 41.4, 36.5, 35.8, 31.9, 31.0, 27.2, 23.2, 22.6, 14.2; **LRMS** *m/z* (ESI^+) 353.3 ($[M+H]^+$, 30%), 375.2 ($[M+Na]^+$, 100%); **HRMS** (ESI^+) $C_{24}H_{33}O_2$ requires 353.2475 $[M+H]^+$, found 353.2476; **ν_{max}** (film)/cm^{-1} 3379 (br), 2928, 2856, 1699, 1589, 1485, 1454, 1374; the **ee** was determined by HPLC using a Chiralpak AD-H column (*n*-hexane/*i*-PrOH, 94:6); flow rate 1.0 mL/min; τ_{major} = 15.72 min, τ_{minor} = 19.15 min (93% ee); $[\alpha]_D^{25}$: +20.1 (*c* = 1.0, $CHCl_3$).

(*S*)-7-[4-(Dimethylamino)phenyl]-1-phenyldodecan-5-one (4h)

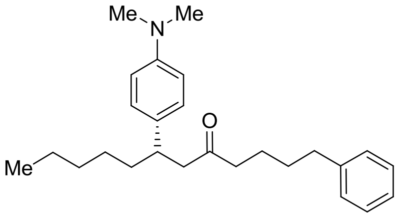

Prepared according to general procedure **C**, using 3-(ethylthio)octanal **1a** (56 mg, 0.30 mmol), 4-phenyl-1-butene (0.14 mL, 0.90 mmol) and [4-(dimethylamino)phenyl]boronic acid (99 mg, 0.60 mmol, 2.0 equiv.). Purification by flash column chromatography (petrol/Et_2O, 95:5) afforded the *ketone* **4h** (66 mg, 0.17 mmol, 58%, 96% ee) as a colorless oil. **δ_H** (400 MHz, $CDCl_3$): 7.30-7.26 (m, 2H, H_{Ar}), 7.20-7.14 (m, 3H, H_{Ar}), 7.04 (d, *J* = 8.5 Hz, 2H, H_{Ar}), 6.69 (d, *J* = 8.5 Hz, 2H, H_{Ar}), 3.06-2.99 (m, 1H, C*H*-CH_2-CO), 2.92 (s, 6H, N(CH_3)$_2$), 2.66 (dd, *J* = 14.5, 6.5 Hz, 1H, CH-CH_aH_b-CO), 2.61 (dd, *J* = 14.5, 6.0 Hz, 1H, CH-CH_aH_b-CO), 2.56 (t, *J* = 7.0 Hz, 2H, CH_2-Ph), 2.34-2.20 (m, 2H, CH_2-CH_2-CO), 1.56-1.47 (m, 6H, 3 × CH_2), 1.27-1.13 (m, 6H, 3 × CH_2), 0.85 (t, *J* = 7.0 Hz, 3H, CH_2-CH_3); **δ_C** (101 MHz, $CDCl_3$): 210.8, 149.3, 142.4, 132.7, 128.5, 128.4, 128.1, 125.8, 112.9, 50.6, 43.5, 40.9, 40.6, 36.7, 35.8, 31.9, 31.0, 27.3, 23.3, 22.7, 14.2; **LRMS** *m/z* (ESI^+) 380.3 ($[M+H]^+$, 100%); **HRMS** (ESI^+) $C_{26}H_{38}ON$ requires 380.2948 $[M+H]^+$, found 380.2940; **ν_{max}** (film)/cm^{-1} 2926, 2855, 1710, 1615, 1520, 1453, 1347, 1224; the **ee** was determined by HPLC using a Chiralpak AD-H column (*n*-hexane/*i*-PrOH, 98:2); flow rate 1.0 mL/min; τ_{major} = 8.95 min, τ_{minor} = 9.64 min (96% ee); $[\alpha]_D^{25}$: +25.1 (*c* = 1.0, $CHCl_3$).

(*S*)-6-(4-Hydroxyphenyl)hexadecan-8-one (4i)

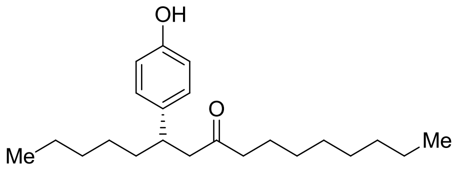

Prepared according to general procedure **C**, using 3-(ethylthio)octanal **1a** (56 mg, 0.30 mmol), 1-octene (0.14 mL, 0.90 mmol) and 4-hydroxyphenylboronic acid (62 mg, 0.45 mmol). Purification by flash column chromatography (petrol/Et_2O, 9:1) afforded the *ketone* **4i** (82 mg, 0.24 mmol, 82%, 94% ee) as a colorless oil. **δ_H** (400 MHz, $CDCl_3$): 7.01 (d, *J* = 8.5 Hz, 2H, H_{Ar}), 6.72 (d, *J* = 8.5 Hz, 2H, H_{Ar}), 5.14 (br s, 1H, O*H*), 3.11-2.99 (m, 1H, C*H*-CH_2-CO), 2.67 (dd, *J* = 13.5, 5.0 Hz, 1H, CH-C$H_a$$H_b$-CO), 2.62 (dd, *J* = 13.5, 4.5 Hz, 1H, CH-C$H_a$$H_b$-CO), 2.37-2.13 (m, 2H, CH_2-CH_2-CO), 1.61-1.33 (m, 4H, 2 × CH_2), 1.33-1.02 (m, 16H, 8 × CH_2), 0.86 (t, *J* = 7.0 Hz, 3H, CH_2-CH_3), 0.82 (t, *J* = 7.0 Hz, 3H, CH_2-CH_3); **δ_C** (101 MHz, $CDCl_3$): 211.7, 154.2, 136.6, 128.6, 115.4, 50.4, 43.7, 40.7, 36.8, 31.94, 31.86, 29.5, 29.3, 29.3, 27.2, 23.7, 22.8, 22.7, 14.24, 14.18; **LRMS** *m/z* (ESI^+) 355.1 ($[M+Na]^+$, 100%); **HRMS** (ESI^+) $C_{22}H_{36}O_2Na$ requires 355.2608 $[M+Na]^+$, found 355.2606; **ν_{max}** (film)/cm^{-1} 3381 (br), 2955, 2925, 2855, 1698, 1614, 1515, 1455, 1375, 1221; the **ee** was determined by HPLC using a Chiralpak IC column (*n*-hexane/*i*-PrOH, 94:6); flow rate 1.0 mL/min; τ_{major} = 7.73 min, τ_{minor} = 8.75 min (94% ee); $[\alpha]_D^{25}$: +21.5 (*c* = 1.0, $CHCl_3$).

(*S*)-6-(4-Bromophenyl)hexadecan-8-one (4j)

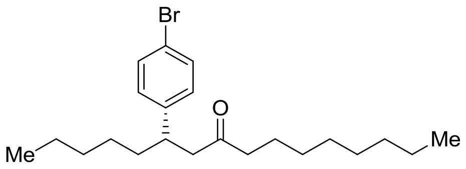

Prepared according to general procedure **C**, using 3-(ethylthio)octanal **1a** (56 mg, 0.30 mmol) and 1-octene (0.14 mL, 0.90 mmol) in DCE. The elimination step was carried out at 22 °C for 1.5 h and the conjugate addition step was performed using 4-bromophenylboronic acid (90 mg, 0.45 mmol), $[Rh(L2)Cl]_2$ (6.9 mg, 0.0075 mmol) and additional K_2CO_3 (41 mg, 0.30 mmol, 1.0 equiv.). Purification by flash column chromatography (petrol/Et_2O, 99:1) afforded the *ketone* **4j** (76 mg, 0.19 mmol, 64%, 93% ee) as a colorless oil. **δ_H** (400 MHz, $CDCl_3$): 7.40-7.37 (m, 2H, H_{Ar}), 7.06-7.03 (m, 2H, H_{Ar}), 3.12-3.08 (m, 1H, C*H*-CH_2-CO), 2.64 (d, *J* = 7.0 Hz, 2H, CH-CH_2-CO), 2.32-2.16 (m, 2H, CH_2-CH_2-CO), 1.59-1.41 (m, 4H, 2 × CH_2), 1.30-1.05 (m, 16H, 8 × CH_2), 0.87 (t, *J* = 7.0 Hz, 3H, CH_2-CH_3), 0.82 (t, *J* = 7.0 Hz, 3H, CH_2-CH_3); **δ_C** (101 MHz, $CDCl_3$): 210.0, 144.0, 131.6, 129.4, 120.0, 49.9, 43.8, 40.7, 36.4, 31.9, 31.8, 29.5, 29.3, 29.2, 27.1, 23.7, 22.8, 22.6, 14.2, 14.1; **LRMS** *m/z* (ESI^+) 395.1 ($[^{79}BrM+H]^+$, 50%), 397.1 ($[^{81}BrM+H]^+$, 50%), 417.2 ($[^{79}BrM+Na]^+$, 100%), 419.2 ($[^{81}BrM+Na]^+$, 100%); **HRMS** (ESI^+) $C_{22}H_{35}O^{79}BrNa$ requires 417.1764 $[M+Na]^+$, found 417.1763, $C_{22}H_{35}O^{81}BrNa$ requires 419.1743 found 419.1741; **ν_{max}** (film)/cm^{-1} 2955, 2925, 2855, 2360, 1714, 1485, 1465, 1375; the **ee** was determined by HPLC using a Chiralpak AD-H column (*n*-hexane/*i*-PrOH, 99:1); flow rate 1.0 mL/min; τ_{major} = 6.28 min, τ_{minor} = 6.79 min (93% ee); $[\alpha]_D^{25}$: +14.1 (*c* = 1.0, $CHCl_3$).

(*S*)-Methyl 4-(8-oxohexadecan-6-yl)benzoate (4k)

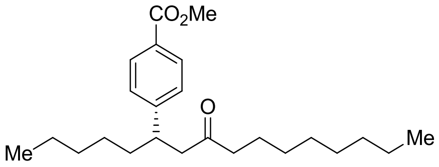

Prepared according to general procedure **C**, using 3-(ethylthio)octanal **1a** (56 mg, 0.30 mmol) and 1-octene (0.14 mL, 0.90 mmol) in DCE. The elimination step was carried out at 22 °C for 1.5 h and the conjugate addition was performed using 4-(methoxycarbonyl)phenylboronic acid (81 mg, 0.45 mmol), $[Rh(L2)Cl]_2$ (6.9 mg, 0.0075 mmol) and additional K_2CO_3 (41 mg, 0.30 mmol, 1.0 equiv.). Purification by flash column chromatography (petrol/Et_2O, 96:4) afforded the *ketone* **4k** (92 mg, 0.25 mmol, 82%, 93% ee) as a colorless oil. **δ_H** (400 MHz, $CDCl_3$): 7.95 (d, *J* = 8.5 Hz, 2H, H_{Ar}), 7.24 (d, *J* = 8.5 Hz, 2H, H_{Ar}), 3.89 (s, 3H, CO_2CH_3), 3.24-3.18 (m, 1H, C*H*-CH_2-CO), 2.71 (dd, *J* = 13.5, 4.5 Hz, 1H, CH-C$H_a$$H_b$-CO), 2.66 (dd, *J* = 13.5, 4.0 Hz, 1H, CH-$CH_a$$H_b$-CO), 2.32-2.16 (m, 2H, CH_2-CH_2-CO), 1.65-1.54 (m, 2H, CH-CH_2-CH_2), 1.48-1.40 (m, 2H, CH_2), 1.28-1.05 (m, 16H, 8 × CH_2), 0.86 (t, *J* = 7.0 Hz, 3H, CH_2-CH_3), 0.81 (t, *J* = 7.0 Hz, 3H, CH_2-CH_3); **δ_C** (101 MHz, $CDCl_3$): 209.9, 167.2, 150.6, 129.9, 128.4, 127.7, 52.1, 49.7, 43.7, 41.2, 36.3, 31.9, 31.8, 29.4, 29.2 (2C), 27.1, 23.7, 22.8, 22.6, 14.2, 14.1; **LRMS** *m/z* (ESI^+) 375.3 ($[M+H]^+$, 80%), 397.3 ($[M+Na]^+$, 100%); **HRMS** (ESI^+) $C_{24}H_{39}O_3$ requires 375.2894 $[M+H]^+$, found 375.2893; **ν_{max}** (film)/cm^{-1} 2928, 2857, 1715, 1437, 1376, 1281; the **ee** was determined by HPLC using a Chiralpak AD-H column (*n*-hexane/*i*-PrOH, 99:1); flow rate 1.0 mL/min; τ_{major} = 17.95 min, τ_{minor} = 24.03 min (93% ee); $[\alpha]_D^{25}$: +15.3 (*c* = 1.0, $CHCl_3$).

(*S*)-4-(8-Oxohexadecan-6-yl)benzonitrile 1d (4l)

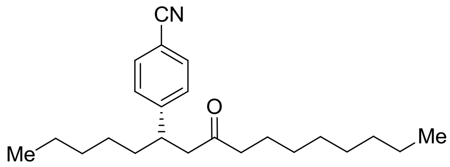

Prepared according to general procedure **C**, using 3-(ethylthio)octanal **1a** (56 mg, 0.30 mmol), 1-octene (0.14 mL, 0.90 mmol) and 4-cyanophenylboronic acid (67 mg, 0.45 mmol). Purification by flash column chromatography (petrol/Et_2O, 98:2) afforded the *ketone* **4l** (56 mg, 0.16 mmol, 55%, 91% ee) as a colorless oil. **δ_H** (400 MHz, $CDCl_3$): 7.56 (d, *J* = 8.0 Hz, 2H, H_{Ar}), 7.28 (d, *J* = 8.0 Hz, 2H, H_{Ar}), 3.12-3.08 (m, 1H, C*H*-CH_2-CO), 2.69 (d, *J* = 7.0 Hz, 2H, CH-CH_2-CO), 2.30 (dt, *J* = 16.5, 7.5 Hz, 1H, CH_2-C$H_a$$H_b$-CO), 2.21 (dt, *J* = 16.5, 7.0 Hz, 1H, CH_2-$CH_a$$H_b$-CO), 1.59-1.41 (m, 4H, 2 × CH_2), 1.30-1.05 (m, 16H, 8 × CH_2), 0.86 (t, *J* = 7.0 Hz, 3H, CH_3), 0.81 (t, *J* = 7.0 Hz, 3H, CH_3); **δ_C** (101 MHz, $CDCl_3$): 209.4, 150.8, 132.4, 128.6, 119.1, 110.2, 49.4, 43.7, 41.1, 36.1, 31.9, 31.7, 29.4, 29.2 (2C), 27.1, 23.7, 22.8, 22.6, 14.2, 14.1; **LRMS** *m/z* (ESI^+) 364.1 ($[M+Na]^+$, 100%); **HRMS** (ESI^+) $C_{23}H_{35}NONa$ requires 364.2611 $[M+Na]^+$, found 364.2612; **ν_{max}** (film)/cm^{-1} 2955, 2926, 2855, 2228, 1713, 1608, 1464, 1375; the **ee** was determined by HPLC using a Chiralpak AD-H column (*n*-hexane/*i*-PrOH, 99:1); flow rate 1.0 mL/min; τ_{major} = 17.15 min, τ_{minor} = 19.09 min (91% ee); $[\alpha]_D^{25}$: +10.2 (*c* = 1.0, $CHCl_3$).

(*S*)-7-(2,3-Dihydrobenzo[b][1,4]dioxin-6-yl)-1-phenyldodecan-5-one (4m)

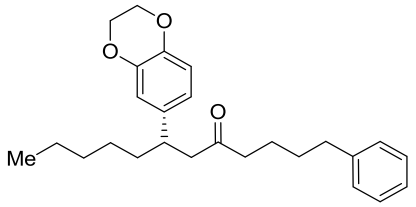

Prepared according to general procedure **C**, using 3-(ethylthio)octanal **1a** (56 mg, 0.30 mmol), 4-phenyl-1-butene (0.14 mL, 0.90 mmol), 1,4-benzodioxane-6-boronic acid (82 mg, 0.6 mmol, 2.0 equiv.) and $[Rh(L2)Cl]_2$ (6.9 mg, 0.0075 mmol). Purification by flash column chromatography (petrol/Et_2O, 9:1) afforded the *ketone* **4m** (98 mg, 0.25 mmol, 82%**,** 94% ee) as a yellow oil. $\boldsymbol{\delta_H}$ (400 MHz, $CDCl_3$): 7.23 (d, *J* = 7.5 Hz, 2H, H_{Ar}), 7.16-7.10 (m, 3H, H_{Ar}), 6.74 (d, *J* = 8.0 Hz, 1H, H_{Ar}), 6.64-6.59 (m, 2H, H_{Ar}), 4.19-4.17 (m, 4H, O-CH_2-CH_2-O), 3.01-2.94 (m, 1H, C*H*-CH_2-CO), 2.61 (dd, *J* = 16.0, 7.5 Hz, 1H, CH-C$H_a$$H_b$-CO), 2.58-2.51 (m, 3H, CH-$CH_a$$H_b$-CO + C$H_2$-Ph), 2.32-2.18 (m, 2H, CH_2-CH_2-CO), 1.51-1.42 (m, 6H, 3 × CH_2), 1.22-1.08 (m, 6H, 3 × CH_2), 0.81 (t, *J* = 7.0 Hz, 3H, CH_2-CH_3); $\boldsymbol{\delta_C}$ (101 MHz, $CDCl_3$): 210.3, 143.4, 142.4, 141.9, 138.2, 128.5, 128.4, 125.8, 120.6, 117.1, 116.0, 64.5, 64.4, 50.3, 43.5, 40.8, 36.6, 35.8, 31.9, 31.0, 27.2, 23.3, 22.6, 14.2; **LRMS** *m/z* (ESI^+) 417.3 ($[M+Na]^+$, 100%); **HRMS** (ESI^+) $C_{26}H_{34}O_3Na$ requires 417.2400 $[M+Na]^+$, found 417.2406; $\boldsymbol{\nu_{max}}$ (film)/cm^{-1} 2927, 2360, 1710, 1590, 1504, 1456, 1306, 1284, 1255; the **ee** was determined by HPLC using a Chiralpak AD-H column (*n*-hexane/*i*-PrOH, 98:2); flow rate 1.0 mL/min; τ_{major} = 18.21 min, τ_{minor} = 17.06 min (94% ee); $[\boldsymbol{\alpha}]_{\mathbf{D}}^{\mathbf{25}}$: +18.3 (*c* = 1.0, $CHCl_3$).

(*S*)-1-Phenyl-7-(thiophen-3-yl)dodecan-5-one (4n)

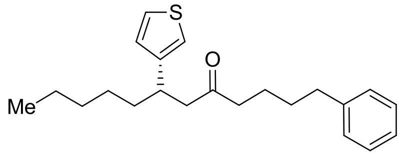

Prepared according to general procedure **C**, using 3-(ethylthio)octanal **1a** (56 mg, 0.30 mmol), 4-phenyl-1-butene (0.14 mL, 0.90 mmol) and 3-thiophenylboronic acid (115 mg, 0.90 mmol, 3.0 equiv.). Purification by flash column chromatography (petrol/Et_2O, 99:1) afforded the *ketone* **4n** (28 mg, 0.08 mmol, 27%, 94% ee) as a colorless oil. $\boldsymbol{\delta_H}$ (400 MHz, $CDCl_3$): 7.28-7.22 (m, 3H, H_{Ar}), 7.18-7.12 (m, 3H, H_{Ar}), 6.91 (d, *J* = 4.0 Hz, 2H, H_{Ar}), 3.30-3.23 (m, 1H, C*H*-CH_2-CO), 2.64 (dd, *J* = 15.0, 6.4 Hz, 1H, CH-C$H_a$$H_b$-CO), 2.61-2.53 (m, 3H, CH-$CH_a$$H_b$-CO + C$H_2$-Ph), 2.33-2.19 (m, 2H, CH_2-CH_2-CO), 1.61-1.47 (m, 6H, 3 × CH_2), 1.25-1.12 (m, 6H, 3 × CH_2), 0.83 (t, *J* = 7.0 Hz, 3H, CH_2-CH_3); $\boldsymbol{\delta_C}$ (101 MHz, $CDCl_3$): 210.2, 145.6, 142.3, 128.5, 128.4, 126.7, 125.9, 125.6, 120.3, 49.9, 43.5, 36.7, 36.3, 35.8, 31.8, 31.0, 27.1, 23.3, 22.7, 14.2; **LRMS** *m/z* (ESI^+) 343.2 ($[M+H]^+$, 15%), 365.2 ($[M+Na]^+$, 100%); **HRMS** (ESI^+) $C_{22}H_{30}OSNa$ requires 365.1910 $[M+Na]^+$, found 365.1910; $\boldsymbol{\nu_{max}}$ (film)/cm^{-1} 2927, 2857, 2361, 1712, 1496, 1454, 1409, 1369; the **ee** was determined by HPLC using a Chiralpak OD-H column (*n*-hexane/*i*-PrOH, 99:1); flow rate 1.0 mL/min; τ_{major} = 15.80 min, τ_{minor} = 21.44 min (94% ee); $[\boldsymbol{\alpha}]_{\mathbf{D}}^{\mathbf{25}}$: +14.5 (*c* = 1.0, $CHCl_3$).

(*S*)-2-[8-(Cyclohex-1-en-1-yl)-6-oxotridecyl]isoindoline-1,3-dione (4o)

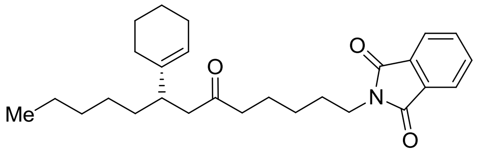

Prepared according to general procedure **C**, using 3-(ethylthio)octanal **1a** (38 mg, 0.20 mmol), 2-(pent-4-en-1-yl)isoindoline-1,3-dione **SM1a** (129 mg, 0.60 mmol), 1-cyclohexenylboronic acid (50 mg, 0.60 mmol, 2.0 equiv.) and $[Rh(L2)Cl]_2$ (4.6 mg, 0.005 mmol). Purification by flash column chromatography (petrol/EtOAc, 95:5) afforded the *ketone* **4o** (71 mg, 0.17 mmol, 84%, 89% ee) as a yellow oil. **δ_H** (400 MHz, $CDCl_3$): 7.84-7.82 (m, 2H, H_{Ar}), 7.72-7.69 (m, 2H, H_{Ar}), 5.38 (app. s, 1H, C=C*H*), 3.66 (t, *J* = 7.5 Hz, 2H, N-CH_2), 2.43-2.30 (m, 5H, C*H*-CH_2-CO and CH-CH_2-CO and CH_2-CH_2-CO), 1.98-1.93 (m, 2H, CH_2), 1.88-1.78 (m, 2H, CH_2), 1.66-1.50 (m, 8H, 4 × CH_2), 1.33-1.16 (m, 10H, 5 × CH_2), 0.85 (t, *J* = 7.0 Hz, 3H, CH_2-CH_3); **δ_C** (101 MHz, $CDCl_3$): 210.8, 168.5, 138.8, 134.0, 132.3, 123.3, 122.9, 47.7, 43.6, 43.0, 38.0, 33.3, 32.0, 28.6, 27.1, 26.6, 25.3, 24.8, 23.2, 23.1, 22.8, 22.7, 14.2; **LRMS** *m/z* (ESI^+) 424.3 ($[M+H]^+$, 40%), 446.2 ($[M+Na]^+$, 100%); **HRMS** (ESI^+) $C_{27}H_{38}NO_3$ requires 424.2846 $[M+H]^+$, found 424.2846; **ν_{max}** (film)/cm^{-1} 2925, 2856, 1773, 1709, 1466, 1395, 1367; the **ee** was determined by HPLC using a Chiralpak IA-3 column (*n*-hexane/*i*-PrOH, 90:10); flow rate 0.8 mL/min; τ_{major} = 9.69 min, τ_{minor} = 10.20 min (89% ee); $[\alpha]_D^{25}$: +5.5 (*c* = 1.0, $CHCl_3$).

3.2. Alkene scope

(*S*)-1-Cyclopentyl-6-(4-methoxyphenyl)undecan-4-one (4p)

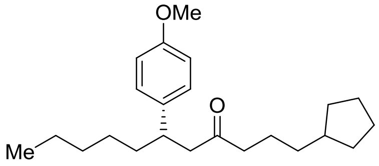

Prepared according to general procedure **C**, using 3-(ethylthio)octanal **1a** (56 mg, 0.30 mmol), allylcyclopentane (0.13 mL, 0.90 mmol) and 4-methoxyphenylboronic acid (68 mg, 0.45 mmol). Purification by flash column chromatography (petrol/Et_2O, 97:3) afforded the *ketone* **4p** (78 mg, 0.23 mmol, 75%, 91% ee) as a colorless oil. **δ_H** (400 MHz, $CDCl_3$): 7.08 (d, *J* = 8.5 Hz, 2H, H_{Ar}), 6.81 (d, *J* = 8.5 Hz, 2H, H_{Ar}), 3.78 (s, 3H, OCH_3), 3.11-3.02 (m, 1H, C*H*-CH_2-CO), 2.66 (dd, *J* = 14.0, 5.5 Hz, 1H, CH-C$H_a$$H_b$-CO), 2.60 (dd, *J* = 14.0, 5.0 Hz, 1H, CH-$CH_a$$H_b$-CO), 2.26 (dt, *J* = 16.5, 7.5 Hz, 1H, CH_2-C$H_c$$H_d$-CO), 2.18 (dt, *J* = 16.5, 7.5 Hz, 1H, CH_2-$CH_c$$H_d$-CO), 1.75-1.39 (m, 12H, 6 × CH_2), 1.27-0.93 (m, 9H, CO-$(CH_2)_3$-C*H* and 4 × CH_2), 0.82 (t, *J* = 7.0 Hz, 3H, CH_2-CH_3); **δ_C** (101 MHz, $CDCl_3$): 210.8, 158.0, 136.9, 128.5, 113.9, 55.3, 50.4, 44.0, 40.6, 40.0, 36.7, 35.7, 32.7, 31.9, 27.2, 25.3, 22.9, 22.7, 14.2; **LRMS** m/z (ESI^+) 367.3 ($[M+Na]^+$, 100%); **HRMS** (ESI^+) $C_{23}H_{36}O_2Na$ requires 367.2608 $[M+Na]^+$, found 367.2610; **ν_{max}** (film)/cm^{-1} 2928, 2857, 1712, 1611, 1512, 1456, 1247, 1178, 1038; the **ee** was determined by HPLC using Chiralpak IC/AS-H columns in series (*n*-hexane/*i*-PrOH, 99:1); flow rate 0.70 mL/min; τ_{major} = 20.15 min, τ_{minor} = 21.51 min (91% ee); $[\alpha]_D^{25}$: +19.9 (c = 1.0, $CHCl_3$).

(*S*)-7-(4-Methoxyphenyl)-1-phenyldodecan-5-one (4q)

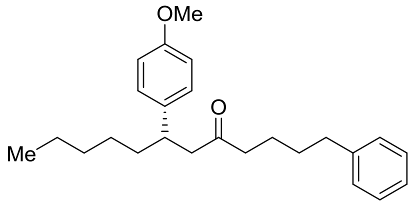

Prepared according to general procedure **C**, using 3-(ethylthio)octanal **1a** (56 mg, 0.30 mmol), 4-phenyl-1-butene (0.14 mL, 0.90 mmol) and 4-methoxyphenylboronic acid (68 mg, 0.45 mmol). Purification by flash column chromatography (petrol/Et_2O, 96:4) afforded the *ketone* **4q** (92 mg, 0.25 mmol, 84%, 93% ee) as a pale yellow oil. **δ_H** (400 MHz, $CDCl_3$): 7.32-7.25 (m, 2H, H_{Ar}), 7.23-7.13 (m, 3H, H_{Ar}), 7.10 (d, *J* = 8.5 Hz, 2H, H_{Ar}), 6.84 (d, *J* = 8.5 Hz, 2H, H_{Ar}), 3.80 (s, 3H, OC*H*$_3$), 3.14-3.04 (m, 1H, C*H*-CH_2-CO), 2.68 (dd, *J* = 14.0, 6.0 Hz, 1H, CH-C$H_a$$H_b$-CO), 2.63 (dd, *J* = 14.0, 5.5 Hz, 1H, CH-C$H_a$$H_b$-CO), 2.57 (t, *J* = 7.0 Hz, 2H, C*H*$_2$-Ph), 2.38-2.21 (m, 2H, CH_2-C*H*$_2$-CO), 1.64-1.47 (m, 6H, 3 × C*H*$_2$), 1.31-1.05 (m, 6H, 3 × C*H*$_2$), 0.85 (t, *J* = 7.0 Hz, 3H, CH_2-C*H*$_3$); **δ_C** (101 MHz, $CDCl_3$): 210.4, 158.1, 142.4, 136.8, 128.5 (2C), 128.4, 125.8, 113.9, 55.3, 50.4, 43.5, 40.7, 36.7, 35.8, 31.9, 31.0, 27.2, 23.3, 22.6, 14.2; **LRMS** m/z (ESI^+) 389.2 ($[M+Na]^+$, 100%); **HRMS** (ESI^+) $C_{25}H_{34}O_2Na$ requires 389.2462 $[M+Na]^+$, found 389.2451; **ν_{max}** (film)/cm^{-1} 2927, 2856, 1712, 1611, 1512, 1455, 1247, 1178, 1036; the **ee** was determined by HPLC using a Chiralpak AD-H column (*n*-hexane/*i*-PrOH, 99:1); flow rate 1.0 mL/min; τ_{major} = 15.99 min, τ_{minor} = 18.79 min (93% ee); $[\alpha]_D^{25}$: +19.6 (c = 1.0, $CHCl_3$).

(*S*)-9-(4-Methoxyphenyl)tetradecane-2,7-dione (4r)

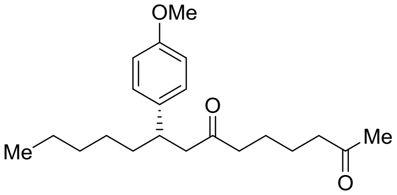

Prepared according to general procedure **C**, using 3-(ethylthio)octanal **1a** (56 mg, 0.30 mmol), 5-hexen-2-one (0.10 mL, 0.90 mmol) and 4-methoxyphenylboronic acid (68 mg, 0.45 mmol). Purification by flash column chromatography (gradient petrol/Et_2O, 9:1 to 7:3) afforded the *ketone* **4r** (84 mg, 0.25 mmol, 84%, 94% ee) as a pale yellow oil. **δ_H** (400 MHz, $CDCl_3$): 7.06 (d, *J* = 8.5 Hz, 2H, H_{Ar}), 6.81 (d, *J* = 8.5 Hz, 2H, H_{Ar}), 3.77 (s, 3H, OC*H*$_3$), 3.09-2.99 (m, 1H, C*H*-CH_2-CO), 2.65 (dd, *J* = 14.0, 6.0 Hz, 1H, CH-C$H_a$$H_b$-CO), 2.60 (dd, *J* = 14.0, 5.0 Hz, 1H, CH-C$H_a$$H_b$-CO), 2.34 (t, *J* = 7.0 Hz, 2H, C*H*$_2$-CO-CH_3), 2.31-2.13 (m, 2H, CH_2-C*H*$_2$-CO-CH_2), 2.09 (s, 3H, CO-C*H*$_3$), 1.60-1.46 (m, 2H, CH-C*H*$_2$-CH_2), 1.45-1.38 (m, 4H, C*H*$_2$-C*H*$_2$-CH_2-CO-CH_3), 1.27-1.02 (m, 6H, 3 × C*H*$_2$), 0.81 (t, *J* = 7.0 Hz, 3H, CH_2-C*H*$_3$); **δ_C** (101 MHz, $CDCl_3$): 210.1, 208.9, 158.1, 136.7, 128.5, 113.9, 55.3, 50.4, 43.5, 43.4, 40.7, 36.7, 31.8, 30.0, 27.2, 23.2, 23.0, 22.6, 14.2; **LRMS** m/z (ESI^+) 333.2 ($[M+H]^+$, 60%), 355.2 ($[M+Na]^+$, 100%); **HRMS** (ESI^+) $C_{21}H_{32}O_3Na$ requires 355.2244 $[M+Na]^+$, found 355.2244; **ν_{max}** (film)/cm^{-1} 2956, 2928, 2856, 1712, 1611, 1512, 1465, 1357, 1246, 1178, 1036; the **ee** was determined by HPLC using a Chiralpak IC column (*n*-hexane/*i*-PrOH, 98:2); flow rate 1.0 mL/min; τ_{major} = 99.08 min, τ_{minor} = 110.04 min (94% ee); $[\alpha]_D^{25}$: +23.4 (c = 1.0, $CHCl_3$).

(*S*)-Diethyl 2-[6-(4-methoxyphenyl)-4-oxoundecyl]malonate (4s)

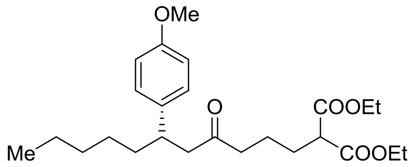

Prepared according to general procedure **C**, using 3-(ethylthio)octanal **1a** (56 mg, 0.30 mmol), diethyl allylmalonate (0.18 mL, 0.90 mmol) and 4-methoxyphenylboronic acid (68 mg, 0.45 mmol). Purification by flash column chromatography (gradient petrol/Et_2O, 9:1 to 7:3) afforded the *ketone* **4s** (78 mg, 0.18 mmol, 60%, 94% ee) as a pale yellow oil. **δ_H** (400 MHz, $CDCl_3$): 7.06 (d, *J* = 8.5 Hz, 2H, H_{Ar}), 6.81 (d, *J* = 8.5 Hz, 2H, H_{Ar}), 4.17 (app. qd, *J* = 7.0, 1.5 Hz, 4H, 2 × $CO_2CH_2CH_3$), 3.77 (s, 3H, OCH_3), 3.24 (t, *J* = 7.5 Hz, 1H, C*H*-CO_2Et), 3.09-2.99 (m, 1H, C*H*-CH_2-CO), 2.65 (dd, *J* = 13.0, 5.0 Hz, 1H, CH-C$H_a$$H_b$-CO), 2.60 (dd, *J* = 13.0, 4.5 Hz, 1H, CH-C$H_a$$H_b$-CO), 2.37-2.15 (m, 2H, CH_2-CH_2-CO), 1.80-1.71 (m, 2H, CH_2-CH-CO_2Et), 1.60-1.43 (m, 4H, CH_2-CH-CH_2-CO and CH_2-CH_2-CO), 1.29-1.02 (m, 12H, 2 × $CO_2CH_2CH_3$ and 3 × CH_2), 0.81 (t, *J* = 7.0 Hz, 3H, CH_2-CH_2-CH_3); **δ_C** (101 MHz, $CDCl_3$): 209.6, 169.4, 158.1, 136.7, 128.4, 113.9, 61.5, 55.3, 52.0, 50.4, 43.1, 40.7, 36.7, 31.8, 28.2, 27.2, 22.6, 21.3, 14.19, 14.17; **LRMS** m/z (ESI^+) 435.3 ($[M+H]^+$, 50%), 457.3 ($[M+Na]^+$, 100%); **HRMS** (ESI^+) $C_{25}H_{38}O_6Na$ requires 457.2561 $[M+Na]^+$, found 457.2558; **ν_{max}** (film)/cm^{-1} 2956, 2930, 2858, 1749, 1731, 1513, 1463, 1369, 1247, 1178, 1035; the **ee** was determined by HPLC using a Chiralpak AS-H column (*n*-hexane/*i*-PrOH, 99:1); flow rate 1.0 mL/min; τ_{major} = 19.17 min, τ_{minor} = 23.34 min (94% ee); $[\alpha]_D^{25}$: +19.2 (c = 1.0, $CHCl_3$).

(*S*)-1-Bromo-9-(4-methoxyphenyl)tetradecan-7-one (4t)

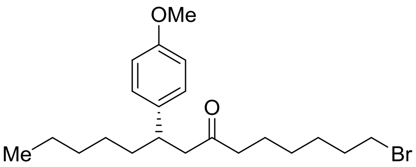

Prepared according to general procedure **C**, using 3-(ethylthio)octanal **1a** (56 mg, 0.30 mmol), 6-bromo-1-hexene (0.12 mL, 0.90 mmol) and 4-methoxyphenylboronic acid (68 mg, 0.45 mmol). Purification by flash column chromatography (petrol/Et_2O, 96:4) afforded the *ketone* **4t** (101 mg, 0.25 mmol, 85%, 92% ee) as a colorless oil. **δ_H** (400 MHz, $CDCl_3$): 7.07 (d, *J* = 8.5 Hz, 2H, H_{Ar}), 6.81 (d, *J* = 8.5 Hz, 2H, H_{Ar}), 3.78 (s, 3H, OCH_3), 3.36 (t, *J* = 7.0 Hz, 2H, CH_2-Br), 3.11-3.01 (m, 1H, C*H*-CH_2-CO), 2.66 (dd, *J* = 14.5, 6.5 Hz, 1H, CH-C$H_a$$H_b$-CO), 2.60 (dd, *J* = 14.5, 6.0 Hz, 1H, CH-C$H_a$$H_b$-CO), 2.32-2.14 (m, 2H, CH_2-CH_2-CO), 1.84-1.75 (m, 2H, CH_2-CH_2-Br), 1.62-1.50 (m, 2H, CH-CH_2-CH_2), 1.50-1.40 (m, 2H, CH_2-CH_2-CO), 1.39-1.29 (m, 2H, CH_2-CH_2-CH_2-Br), 1.28-1.02 (m, 8H, 4 × CH_2), 0.82 (t, *J* = 7.0 Hz, 3H, CH_2-CH_3); **δ_C** (101 MHz, $CDCl_3$): 210.4, 158.1, 136.8, 128.5, 113.9, 55.3, 50.5, 43.5, 40.7, 36.7, 33.9, 32.6, 31.9, 28.3, 28.0, 27.2, 23.3, 22.6, 14.2; **LRMS** m/z (ESI^+) 419.1 ($[^{79}BrM+Na]^+$, 100%), 421.1 ($[^{81}BrM+Na]^+$, 100%); **HRMS** (ESI^+) $C_{21}H_{33}O_2{}^{79}BrNa$ requires 419.1556 $[^{79}BrM+Na]^+$, found 419.1554, $C_{21}H_{33}O_2{}^{81}BrNa$ requires 421.1536 $[^{81}BrM+Na]^+$, found 421.1534; **ν_{max}** (film)/cm^{-1} 2928, 2856, 1712, 1611, 1512, 1462, 1246, 1178, 1037; the **ee** was determined by HPLC using a Chiralpak AS-H column (*n*-hexane/*i*-PrOH, 99:1); flow rate 0.60 mL/min; τ_{major} = 12.91 min, τ_{minor} = 15.42 min (92% ee); $[\alpha]_D^{25}$: +19.6 (c = 1.0, $CHCl_3$).

Gram Scale preparation:

- Rh(nbd)$_2$BF$_4$ (41 mg, 0.11 mmol, 2 mol%), dcpm (45 mg, 0.11 mmol, 2 mol%), 3-(ethylthio)octanal **1a** (1.04 g, 5.5 mmol, 1.0 equiv.), 6-bromo-1-hexene (2.2 mL, 16.5 mmol) in acetone (2.0 mL, 2.75 M) for the hydroacylation step;

- K_2CO_3 (1.90 g, 13.75 mmol), MeOTf (0.93 mL, 8.25 mmol) and additional acetone (total 11.0 mL, 0.5 M) for the elimination;

- 4-methoxyphenylboronic acid (1.25 g, 8.25 mmol.), additional K_2CO_3 (380 mg, 2.75 mmol, 0.5 equiv.) and [Rh(L2)Cl]$_2$ (51 mg, 0.055 mmol, 1 mol%) with additional acetone (total 49 mL) and methanol (5.5 mL) for the conjugate addition step.

- Purification by flash column chromatography afforded *ketone* **4t** (1.87 g, 4.7 mmol, 86%, 95% ee) as a colorless oil.

(*S*)-1,1-Diethoxy-6-(4-methoxyphenyl)undecan-4-one (4u)

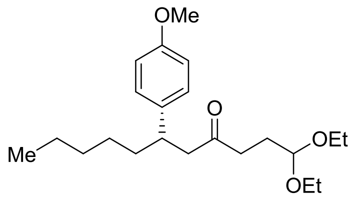

Prepared according to general procedure **C**, using 3-(ethylthio)octanal **1a** (56 mg, 0.30 mmol), distilled 3,3-diethoxy-1-propene (0.14 mL, 0.90 mmol) and 4-methoxyphenylboronic acid (137 mg, 0.90 mmol, 3.0 equiv.). The conjugate addition step was carried out for 14 h. Purification by flash column chromatography (petrol/Et_2O, 9:1) afforded the *ketone* **4u** (46 mg, 0.13 mmol, 42%, 95% ee) as a yellow oil. δ_H (400 MHz, $CDCl_3$): 7.07 (d, *J* = 8.5 Hz, 2H, H_{Ar}), 6.81 (d, *J* = 8.5 Hz, 2H, H_{Ar}), 4.38 (t, *J* = 5.5 Hz, 1H, C*H*-OEt), 3.77 (s, 3H, OCH_3), 3.62-3.53 (m, 2H, O-CH_2-CH$_3$), 3.47-3.35 (m, 2H, O-CH_2-CH$_3$), 3.11-3.02 (m, 1H, C*H*-CH$_2$-CO), 2.68 (dd, *J* = 13.0, 4.5 Hz, 1H, CH-CH_aH$_b$-CO), 2.63 (dd, *J* = 13.0, 4.0 Hz, 1H, CH-CH$_a$$H_b$-CO), 2.39 (dt, *J* = 17.5, 7.0 Hz, 1H, CH$_2$-CH_cH$_d$-CO), 2.28 (dt, *J* = 17.5, 7.0 Hz, 1H, CH$_2$-CH$_c$$H_d$-CO), 1.81-1.73 (m, 2H, CH_2-CH$_2$-CO), 1.61-1.44 (m, 2H, CH_2-CH-CH$_2$-CO), 1.26-1.04 (m, 12H, 2 × O-CH$_2$-CH_3 and 3 × CH_2), 0.82 (t, *J* = 7.0 Hz, 3H, CH$_2$-CH$_2$-CH_3); δ_C (101 MHz, $CDCl_3$): 209.8, 158.1, 136.8, 128.5, 113.9, 102.1, 61.7, 61.6, 55.3, 50.5, 40.6, 38.5, 36.7, 31.9, 27.6, 27.2, 22.6, 15.41, 15.40, 14.2; **LRMS** m/z (ESI$^+$) 387.3 ([M+Na]$^+$, 100%); **HRMS** (ESI$^+$) $C_{22}H_{36}O_4Na$ requires 387.2506 [M+Na]$^+$, found 387.2502; ν_{max} (film)/cm^{-1} 2972, 2929, 2873, 1714, 1611, 1513, 1458, 1373, 1248, 1122, 1062; the **ee** was determined by HPLC using a Chiralpak IC column (*n*-hexane/*i*-PrOH, 98:2]; flow rate 1.0 mL/min; τ_{major} = 17.33 min, τ_{minor} = 19.99 min (95% ee); $[\alpha]_D^{25}$: +14.3 (c = 1.0, $CHCl_3$).

(*S*)-*N*,*N*-Di-Boc-1-amino-7-(4-methoxyphenyl)dodecan-5-one (4w)

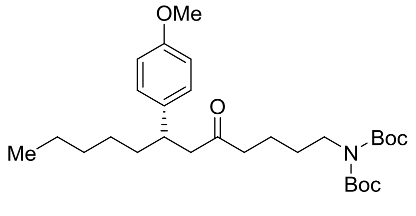

Prepared according to general procedure **D**, using 3-(ethylthio)octanal **1a** (38 mg, 0.20 mmol), di-*tert*-butyl *N*-3-butenyliminodicarboxylate **SM1b** (0.16 mL, 0.60 mmol) and 4-methoxyphenylboronic acid (61 mg, 0.40 mmol, 2.0 equiv.). Purification by flash column chromatography (petrol/Et_2O, 9:1) afforded the *ketone* **4w** (59 mg, 0.12 mmol, 58%, 98% ee) as a pale yellow oil. **δ_H** (400 MHz, $CDCl_3$): 7.06 (d, *J* = 8.5 Hz, 2H, H_{Ar}), 6.81 (d, *J* = 8.5 Hz, 2H, H_{Ar}), 3.77 (s, 3H, OCH_3), 3.49 (t, *J* = 6.5 Hz, 2H, CH_2-N), 3.10-3.01 (m, 1H, *CH*-CH_2-CO), 2.68-2.56 (m, 2H, CH-CH_2-CO), 2.36-2.15 (m, 2H, CH_2-CH_2-CO), 1.65-1.42 (m, 24H, 2 × $C(CH_3)_3$ and 3 × CH_2), 1.26-1.04 (m, 6H, 3 × CH_2), 0.81 (t, *J* = 6.5 Hz, 3H, CH_2-CH_3); **δ_C** (101 MHz, $CDCl_3$): 210.0, 158.1, 152.8, 136.8, 128.5, 113.9, 82.3, 55.3, 50.4, 46.1, 43.2, 40.6, 36.7, 31.9, 28.6, 28.2, 27.2, 22.6, 20.8, 14.2; **HRMS** (ESI^+) $C_{29}H_{47}O_6NNa$ requires 528.3296 $[M+Na]^+$, found 528.3287; **ν_{max}** (film)/cm^{-1} 2956, 2931, 2856, 1747, 1714, 1691, 1513, 1458, 1367, 1247, 1178, 1136, 1037; the **ee** was determined by HPLC using a Chiralpak IA-3 column (*n*-hexane/*i*-PrOH, 98:2); flow rate 1.0 mL/min; τ_{major} = 6.89 min, τ_{minor} = 7.59 min (98% ee); $[\alpha]_D^{25}$: +12.0 (c = 1.0, $CHCl_3$).

(*S*)-9-(4-Methoxyphenyl)-7-oxotetradecyl acetate (4x)

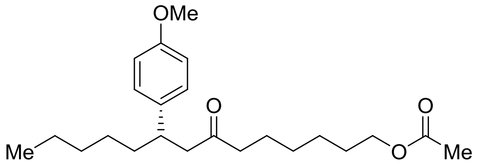

Prepared according to general procedure **C**, using 3-(ethylthio)octanal **1a** (56 mg, 0.30 mmol), 5-hexenyl acetate (0.14 mL, 0.90 mmol) and 4-methoxyphenylboronic acid (68 mg, 0.45 mmol). Purification by flash column chromatography (gradient petrol/Et_2O, 90:10 to 85:15) afforded the *ketone* **4x** (79 mg, 0.21 mmol, 70%, 93% ee) as a yellow oil. **δ_H** (400 MHz, $CDCl_3$): 7.07 (d, *J* = 8.5 Hz, 2H, H_{Ar}), 6.81 (d, *J* = 8.5 Hz, 2H, H_{Ar}), 4.01 (t, *J* = 6.5 Hz, 2H, CO_2CH_2), 3.77 (s, 3H, OCH_3), 3.10-3.01 (m, 1H, *CH*-CH_2-CO), 2.66 (dd, *J* = 14.0, 6.0 Hz, 1H, CH-CH_aH_b-CO), 2.60 (dd, *J* = 14.0, 5.0 Hz, 1H, CH-CH_aH_b-CO), 2.27 (dt, *J* = 17.0, 7.5 Hz, 1H, CH_2-CH_cH_d-CO), 2.18 (dt, *J* = 17.0, 7.5 Hz, 1H, CH_2-CH_cH_d-CO), 2.03 (s, 3H, CH_3-CO_2CH_2), 1.60-1.40 (m, 6H, CO_2CH_2-CH_2, CH-CH_2-CH_2 and CH_2-CH_2-CO), 1.31-1.05 (m, 10H, 5 × CH_2), 0.82 (t, *J* = 7.0 Hz, 3H, CH_2-CH_3); **δ_C** (101 MHz, $CDCl_3$): 210.5, 171.3, 158.1, 136.8, 128.5, 113.9, 64.6, 55.3, 50.4, 43.6, 40.7, 36.7, 31.8, 28.8, 28.5, 27.2, 25.8, 23.4, 22.6, 21.1, 14.2; **LRMS** m/z (ESI^+) 377.3 ($[M+H]^+$, 50%), 399.3 ($[M+Na]^+$, 100%); **HRMS** (ESI^+) $C_{23}H_{36}O_4Na$ requires 399.2506 $[M+Na]^+$, found 399.2501; **ν_{max}** (film)/cm^{-1} 2928, 2857, 1737, 1712, 1611, 1512, 1463, 1366, 1244, 1178, 1037; the **ee** was determined by HPLC using a Chiralpak AD-H column (*n*-hexane/*i*-PrOH, 98:2); flow rate 1.0 mL/min; τ_{major} = 14.58 min, τ_{minor} = 16.20 min (93% ee); $[\alpha]_D^{25}$: +18.2 (c = 1.0, $CHCl_3$).

(*S*)-2-[8-(4-Methoxyphenyl)-6-oxotridecyl]isoindoline-1,3-dione (4y)

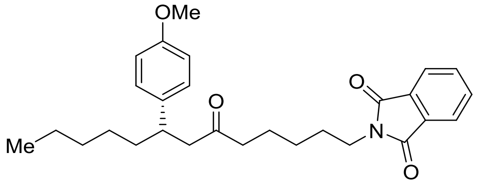

Prepared according to general procedure **C**, using 3-(ethylthio)octanal **1a** (56 mg, 0.30 mmol), 2-(pent-4-en-1-yl)isoindoline-1,3-dione **SM1a** (194 mg, 0.90 mmol) and 4-methoxyphenylboronic acid (68 mg, 0.45 mmol). Purification by flash column

chromatography (petrol/Et_2O, 3:1) afforded the *ketone* **4y** (119 mg, 0.26 mmol, 88%, 94% ee) as an off-white solid. **M.p.** (Et_2O): 39-41 °C; **δ_H** (400 MHz, $CDCl_3$): 7.88-7.83 (m, 2H, H_{Ar}), 7.76-7.70 (m, 2H, H_{Ar}), 7.06 (d, J = 8.5 Hz, 2H, H_{Ar}), 6.80 (d, J = 8.5 Hz, 2H, H_{Ar}), 3.76 (s, 3H, OCH_3), 3.62 (t, J = 7.5 Hz, 2H, CH_2-N), 3.09-2.99 (m, 1H, C*H*-CH_2-CO), 2.64 (dd, J = 13.5, 5.5 Hz, 1H, CH-C$H_a$$H_b$-CO), 2.59 (dd, J = 13.5, 4.5 Hz, 1H, CH-C$H_a$$H_b$-CO), 2.27 (dt, J = 17.0, 7.5 Hz, 1H, CH_2-C$H_c$$H_d$-CO), 2.17 (dt, J = 17.0, 7.5 Hz, 1H, CH_2-C$H_c$$H_d$-CO), 1.64-1.43 (m, 6H, CH_2-CH_2-N, CH-CH_2-CH_2 and CH_2-CH_2-CO), 1.27-1.02 (m, 8H, 4 × CH_2), 0.81 (t, J = 7.0 Hz, 3H, CH_2-CH_3); **δ_C** (101 MHz, $CDCl_3$): 210.3, 168.5, 158.0, 136.8, 134.0, 132.3, 128.5, 123.3, 113.9, 55.3, 50.4, 43.4, 40.7, 37.9, 36.6, 31.8, 28.5, 27.2, 26.4, 23.1, 22.6, 14.2; **LRMS** m/z (ESI$^+$) 450.3 ([M+H]$^+$, 100%), 472.3 ([M+Na]$^+$, 80%); **HRMS** (ESI$^+$) $C_{28}H_{36}O_4N$ requires 450.2639 [M+H]$^+$, found 450.2635; **ν_{max}** (film)/cm^{-1} 2928, 2857, 1772, 1709, 1611, 1512, 1466, 1438, 1396, 1369, 1246, 1178, 1037; the **ee** was determined by HPLC using a Chiralpak AS-H column (*n*-hexane/*i*-PrOH, 98:2); flow rate 1.0 mL/min; τ_{major} = 30.49 min, τ_{minor} = 37.93 min (94% ee); $[\alpha]_D^{25}$: +15.9 (c = 1.0, $CHCl_3$).

3.3. Aldehyde scope

(*R*)-7-(4-Methoxyphenyl)-8-methyl-1-phenylnonan-5-one (4aa)

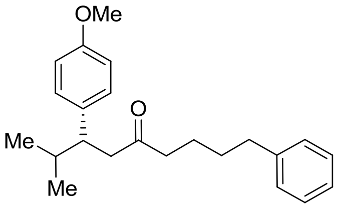

Prepared according to general procedure **C**, using 3-(ethylthio)-4-methylpentanal **1b** (48 mg, 0.30 mmol), 4-phenyl-1-butene (0.14 mL, 0.90 mmol) and 4-methoxyphenylboronic acid (62 mg, 0.45 mmol). Purification by flash column chromatography (petrol/Et_2O, 99:1) afforded the *ketone* **4aa** (83 mg, 0.25 mmol, 82%, 98% ee) as a yellow oil. **δ_H** (400 MHz, $CDCl_3$): 7.25 (d, J = 7.5 Hz, 2H, H_{Ar}), 7.20-7.09 (m, 3H, H_{Ar}), 7.06-7.03 (m, 2H, H_{Ar}), 6.83-6.78 (m, 2H, H_{Ar}), 3.76 (s, 3H, O-CH_3), 2.90-2.84 (m, 1H, C*H*-CH_2-CO), 2.72 (d, J = 7.5 Hz, 2H, CH-CH_2-CO), 2.51 (br t, J = 7.0 Hz, 2H, CH_2-Ph), 2.34-2.12 (m, 2H, CH_2-CH_2-CO), 1.78 (dq, J = 13.5, 7.0 Hz, 1H, C*H*(CH_3)$_2$), 1.48-1.44 (m, 4H, 2 × CH_2), 0.92 (d, J = 7.0 Hz, 3H, CH-CH_3), 0.73 (d, J = 6.5 Hz, 3H, CH-CH_3); **δ_C** (101 MHz, $CDCl_3$): 210.6, 158.0, 142.4, 135.4, 129.3, 128.5, 128.4, 125.8, 113.6, 55.3, 47.5, 47.1, 43.4, 35.8, 33.4, 30.9, 23.2, 20.9, 20.4; **LRMS** *m/z* (ESI$^+$) 361.2 ([M+Na]$^+$, 100%); **HRMS** (ESI$^+$) $C_{23}H_{30}O_2Na$ requires 361.2138 [M+Na]$^+$, found 361.2136; **ν_{max}** (film)/cm^{-1} 3027, 2834, 1711, 1611, 1511, 1367, 1302, 1247, 1179; the **ee** was determined by HPLC using a Chiralpak AD-H column (*n*-hexane/*i*-PrOH, 97:3); flow rate 1.0 mL/min; τ_{major} = 7.73 min, τ_{minor} = 8.69 min (98% ee); $[\alpha]_D^{25}$: +31.1 (c = 1.0, $CHCl_3$).

(*S*)-9-(4-Methoxyphenyl)-11,11-dimethyldodecane-2,7-dione (4ab)

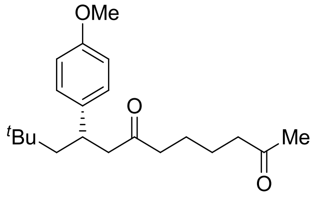

Prepared according to general procedure **C**, using 3-(ethylthio)-5,5-dimethylhexanal **1c** (38 mg, 0.20 mmol), 5-hexen-2-one (70 μL, 0.60 mmol) and 4-methoxyphenylboronic acid (46 mg, 0.30 mmol). Purification by flash column chromatography (gradient petrol/Et_2O, 9:1 to 3:1) afforded the *ketone* **4ab** (40 mg, 0.12 mmol, 60%, 95% ee) as a pale yellow oil. **δ_H** (400 MHz, $CDCl_3$): 7.10 (d, *J* = 8.5 Hz, 2H, H_{Ar}), 6.79 (d, *J* = 8.5 Hz, 2H, H_{Ar}), 3.77 (s, 3H, OCH_3), 3.27-3.19 (m, 1H, C*H*-CH_2-CO), 2.62 (dd, *J* = 15.5, 8.0 Hz, 1H, CH-C$H_a$$H_b$-CO), 2.55 (dd, *J* = 15.5, 6.5 Hz, 1H, CH-C$H_a$$H_b$-CO), 2.36-2.11 (m, 4H, 2 × CH_2-CH_2-CO), 2.09 (s, 3H, CO-CH_3), 1.69-1.44 (m, 2H, CH_2-CH-CH_2-CO), 1.43-1.38 (m, 4H, 2 × CH_2), 0.76 (s, 9H, C(CH_3)$_3$); **δ_C** (101 MHz, $CDCl_3$): 209.9, 208.8, 158.0, 138.2, 128.7, 113.9, 55.3, 52.9, 50.0, 43.53, 43.49, 37.7, 31.4, 30.2, 30.0, 23.3, 22.9; **LRMS** m/z (ESI^+) 355.2 ($[M+Na]^+$, 100%); **HRMS** (ESI^+) $C_{21}H_{32}O_3Na$ requires 355.2244 $[M+Na]^+$, found 355.2242; **ν_{max}** (film)/cm^{-1} 2950, 1713, 1512, 1465, 1364, 1246; the **ee** was determined by HPLC using a Chiralpak IC column (*n*-hexane/*i*-PrOH, 95:5); flow rate 1.0 mL/min; τ_{major} = 38.42 min, τ_{minor} = 33.58 min (95% ee); $[\alpha]_D^{25}$: +43.9 (c = 1.0, $CHCl_3$).

(*R*)-1-Cyclohexyl-1-(4-methoxyphenyl)-7-phenylheptan-3-one (4ac)

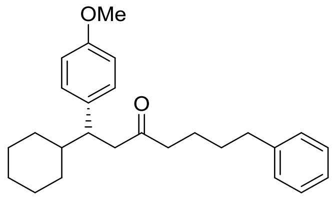

Prepared according to general procedure **C**, using 3-cyclohexyl-3-(ethylthio)propanal **1d** (60 mg, 0.30 mmol), 4-phenyl-1-butene (0.14 mL, 0.90 mmol) and 4-methoxyphenylboronic acid (68 mg, 0.45 mmol). Purification by flash column chromatography (petrol/Et_2O, 94:6) afforded the *ketone* **4ac** (82 mg, 0.22 mmol, 72%, 98% ee) as a colorless oil. **δ_H** (400 MHz, $CDCl_3$): 7.28-7.23 (m, 2H, H_{Ar}), 7.19-7.09 (m, 3H, H_{Ar}), 7.02 (d, *J* = 8.5 Hz, 2H, H_{Ar}), 6.79 (d, *J* = 8.5 Hz, 2H, H_{Ar}), 3.75 (s, 3H, OCH_3), 2.88 (ddd, *J* = 9.5, 7.5, 5.5 Hz, 1H, C*H*-CH_2-CO), 2.75 (dd, *J* = 15.5, 5.5 Hz, 1H, CH-C$H_a$$H_b$-CO), 2.67 (dd, *J* = 15.5, 9.5 Hz, 1H, CH-C$H_a$$H_b$-CO), 2.51 (t, *J* = 7.0 Hz, 2H, CH_2-Ph), 2.28 (dt, *J* = 17.0, 7.0 Hz, 1H, CH_2-C$H_c$$H_d$-CO), 2.17 (dt, *J* = 17.0, 7.0 Hz, 1H, CH_2-C$H_c$$H_d$-CO), 1.80-1.68 (m, 2H, H_{Cy}), 1.65-1.55 (m, 2H, H_{Cy}), 1.50-1.34 (m, 5H, CH_2-CH_2-CH_2-CO and Ar-CH-C*H*), 1.27-0.71 (m, 6H, H_{Cy}); **δ_C** (101 MHz, $CDCl_3$): 210.8, 158.0, 142.4, 135.6, 129.3, 128.5, 128.4, 125.8, 113.6, 55.3, 46.9, 46.6, 43.5, 43.2, 35.8, 31.4, 30.9, 30.8, 26.6, 26.5 (2C), 23.3; **LRMS** m/z (ESI^+) 401.3 ($[M+Na]^+$, 100%); **HRMS** (ESI^+) $C_{26}H_{34}O_2Na$ requires 401.2451 $[M+Na]^+$, found 401.2449; **ν_{max}** (film)/cm^{-1} 3027, 2925, 2851, 1712, 1611, 1511, 1451, 1246; the **ee** was determined by HPLC using a Chiralpak AS-H column (*n*-hexane/*i*-PrOH, 99:1); flow rate 1.0 mL/min; τ_{major} = 9.92 min, τ_{minor} = 13.14 min (98% ee); $[\alpha]_D^{25}$: +30.9 (c = 1.0, $CHCl_3$).

(*R*)-1-Cyclopropyl-1-(4-methoxyphenyl)-7-phenylheptan-3-one (4ad)

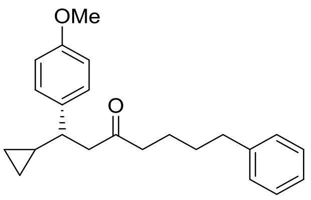

Prepared according to general procedure **D**, using 3-cyclopropyl-3-(ethylthio)propanal **1e** (47 mg, 0.30 mmol), 4-phenyl-1-butene (0.14 mL, 0.90 mmol) and 4-methoxyphenylboronic acid (91 mg, 0.60 mmol, 2.0 equiv.). Purification by flash column chromatography (petrol/Et_2O, 94:6) afforded the *ketone* **4ad** (54 mg, 0.16 mmol, 54%, 98% ee) as a pale yellow oil. **δ_H** (400 MHz, $CDCl_3$): 7.30-7.24 (m, 2H, H_{Ar}), 7.20-7.11 (m, 5H, H_{Ar}), 6.83 (d, *J* = 8.5 Hz, 2H, H_{Ar}), 3.77 (s, 3H, OCH_3), 2.83-2.78 (m, 2H, CH-CH_2-CO), 2.55 (t, *J* = 7.0 Hz, 2H, CH_2-Ph), 2.43-2.22 (m, 3H, C*H*-CH_2-CO and CH_2-CH_2-CO), 1.55-1.49 (m, 4H, CH_2-CH_2-CH_2-CO), 1.02-0.92 (m, 1H, Ar-CH-C*H*), 0.57-0.49 (m, 1H, CH-C$H_a$$H_b$-$CH_cH_d$), 0.44-0.36 (m, 1H, CH-$CH_aH_b$-C$H_c$$H_d$), 0.21 (td, *J* = 9.5, 5.0 Hz, 1H, CH-$CH_a$$H_b$-$CH_cH_d$), 0.13 (td, *J* = 9.5, 5.0 Hz, 1H, CH-CH_aH_b-$CH_c$$H_d$); **$\delta_C$** (101 MHz, $CDCl_3$): 210.2, 158.1, 142.3, 136.7, 128.5, 128.4 (2C), 125.8, 113.9, 55.3, 49.9, 45.6, 43.8, 35.8, 31.0, 23.3, 17.8, 5.4, 4.3; **LRMS** m/z (ESI^+) 337.2 ($[M+H]^+$, 30%), 359.2 ($[M+Na]^+$, 100%); **HRMS** (ESI^+) $C_{23}H_{28}O_2Na$ requires 359.1982 $[M+Na]^+$, found 359.1984; **ν_{max}** (film)/cm^{-1} 3026, 3000, 2934, 2858, 1711, 1611, 1512, 1455, 1245; the **ee** was determined by HPLC using a Chiralpak AD-H column (*n*-hexane/*i*-PrOH, 99:1); flow rate 1.0 mL/min; τ_{major} = 18.76 min, τ_{minor} = 22.94 min (98% ee); $[\alpha]_D^{25}$: –17.9 (c = 1.0, $CHCl_3$).

(*S*)-3-(4-Methoxyphenyl)-1,9-diphenylnonan-5-one (4ae)

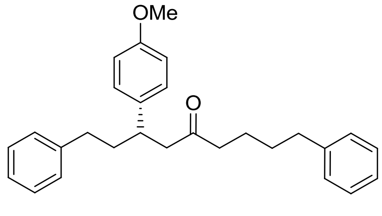

Prepared according to general procedure **C**, using 3-(ethylthio)-5-phenylpentanal **1f** (67 mg, 0.30 mmol), 4-phenyl-1-butene (0.14 mL, 0.90 mmol) and 4-methoxyphenylboronic acid (68 mg, 0.45 mmol). Purification by flash column chromatography (petrol/Et_2O, 98:2) afforded the *ketone* **4ae** (76 mg, 0.19 mmol, 63%, 90% ee) as a yellow oil. **δ_H** (400 MHz, $CDCl_3$): 7.20-7.15 (m, 4H, H_{Ar}), 7.12-6.98 (m, 8H, H_{Ar}), 6.80-6.75 (m, 2H, H_{Ar}), 3.70 (s, 3H, O-CH_3), 3.08-2.98 (m, 1H, C*H*-CH_2-CO), 2.60 (dd, *J* = 16.0, 7.5 Hz, 1H, CH-C$H_a$$H_b$-CO), 2.54 (dd, *J* = 16.0, 7.0 Hz, 1H, CH-$CH_a$$H_b$-CO), 2.45 (t, *J* = 7.0 Hz, 2H, CH_2-Ph), 2.35 (t, *J* = 8.0 Hz, 2H, CH_2-Ph), 2.23-2.06 (m, 2H, CH_2-CH_2-CO), 1.90-1.70 (m, 2H, CH-CH_2-CH_2), 1.42-1.39 (m, 4H, 2 × CH_2); **δ_C** (101 MHz, $CDCl_3$): 210.1, 158.3, 142.3, 142.2, 136.2, 128.6, 128.5 (2C), 128.42, 128.40, 125.9, 125.8, 114.1, 55.3, 50.5, 43.5, 40.3, 38.3, 35.8, 33.8, 31.0, 23.3; **LRMS** *m/z* (ESI^+) 423.3 ($[M+Na]^+$, 100%); **HRMS** (ESI^+) $C_{28}H_{32}O_2Na$ requires 423.2295 $[M+Na]^+$, found 423.2292; **ν_{max}** (film)/cm^{-1} 2930, 2361, 2341, 1710, 1610, 1511, 1368, 1247, 1178; the **ee** was determined by HPLC using a Chiralpak AD-H column (*n*-hexane/*i*-PrOH, 94:6); flow rate 1.0 mL/min; τ_{major} = 8.91 min, τ_{minor} = 9.54 min (90% ee); $[\alpha]_D^{25}$: +15.0 (*c* = 1.0, $CHCl_3$).

(*R*)-1-(4-Methoxyphenyl)-1,7-diphenylheptan-3-one (4af)

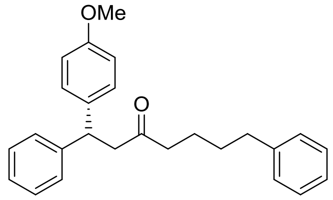

Prepared according to general procedure **C**, using 3-(ethylthio)-3-phenylpropanal **1g** (39 mg, 0.20 mmol), 4-phenyl-1-butene (0.09 mL, 0.60 mmol) and 4-methoxyphenylboronic acid (61 mg, 0.40 mmol, 2.0 equiv.). Purification by flash column chromatography (petrol/Et_2O, 95:5) afforded the *ketone* **4af** (63 mg, 0.17 mmol, 85%, 97% ee) as a yellow oil. $\boldsymbol{\delta_H}$ (400 MHz, $CDCl_3$): 7.26-7.22 (m, 4H, H_{Ar}), 7.18-7.09 (m, 8H, H_{Ar}), 6.78 (d, *J* = 8.5 Hz, 2H, H_{Ar}), 4.53 (t, *J* = 7.5 Hz, 1H, C*H*-CH_2-CO), 3.73 (s, 3H, O-CH_3), 3.08 (d, *J* = 7.5 Hz, 2H, CH-CH_2-CO), 2.51 (t, *J* = 7.0 Hz, 2H, CH_2-Ph), 2.31 (t, *J* = 7.0 Hz, 2H, CH_2-CH_2-CO), 1.55-1.42 (m, 4H, $(CH_2)_2$-CH_2-Ph); $\boldsymbol{\delta_C}$ (101 MHz, $CDCl_3$): 209.2, 158.2, 144.4, 142.3, 136.2, 128.8, 128.7, 128.5, 128.4, 127.7, 126.5, 125.9, 114.0, 55.3, 49.1, 45.4, 43.5, 35.8, 30.9, 23.2; **LRMS** *m/z* (ESI^+) 395.2 ($[M+Na]^+$, 100%); **HRMS** (ESI^+) $C_{26}H_{28}O_2Na$ requires 395.1982 $[M+Na]^+$, found 395.1983; $\boldsymbol{\nu_{max}}$ (film)/cm^{-1} 2933, 1712, 1610, 1510, 1495, 1452, 1368, 1302, 1248; the **ee** was determined by HPLC using a Chiralpak ID-3 column (*n*-hexane/*i*-PrOH, 95:5); flow rate 1.0 mL/min; τ_{major} = 11.25 min, τ_{minor} = 12.12 min (97% ee); $[\boldsymbol{\alpha}]_{\mathbf{D}}^{\mathbf{25}}$: +5.9 (*c* = 1.0, $CHCl_3$).

(*S*)-1-(4-Bromophenyl)-1-(4-methoxyphenyl)-7-phenylheptan-3-one (4ag)

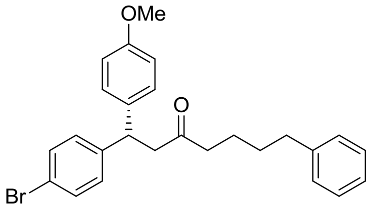

Prepared according to general procedure **C**, using 3-(4-bromophenyl)-3-(ethylthio)propanal **1h** (55 mg, 0.20 mmol), 4-phenyl-1-butene (0.09 mL, 0.60 mmol) and 4-methoxyphenylboronic acid (61 mg, 0.40 mmol, 2.0 equiv.). Purification by flash column chromatography (petrol/Et_2O, 95:5) afforded the *ketone* **4ag** (54 mg, 0.12 mmol, 60%, 98% ee) as a yellow oil. $\boldsymbol{\delta_H}$ (400 MHz, $CDCl_3$): 7.29-7.27 (m, 2H, H_{Ar}), 7.19-7.15 (m, 2H, H_{Ar}), 7.08 (d, *J* = 7.5 Hz, 1H, H_{Ar}), 7.03-6.96 (m, 6H, H_{Ar}), 6.72-6.70 (m, 2H, H_{Ar}), 4.41 (t, *J* = 7.5 Hz, 1H, C*H*-CH_2-CO), 3.66 (s, 3H, O-CH_3), 2.97 (d, *J* = 7.5 Hz, 2H, CH-CH_2-CO), 2.44 (t, *J* = 7.0 Hz, 2H, CH_2-Ph), 2.24 (t, *J* = 6.5 Hz, 2H, CH_2-CH_2-CO), 1.42-1.38 (m, 4H, 2 × CH_2); $\boldsymbol{\delta_C}$ (101 MHz, $CDCl_3$): 208.8, 158.4, 143.5, 142.3, 135.6, 131.7, 129.5, 128.7, 128.5, 128.4, 125.9, 120.3, 114.2, 55.4, 48.9, 44.7, 43.5, 35.8, 30.9, 23.3; **LRMS** *m/z* (ESI^+) 473.1 ($[^{79}BrM+Na]^+$, 50%), 472.2 ($[^{81}BrM+Na]^+$, 50%); **HRMS** (ESI^+) $C_{26}H_{27}O^{79}BrNa$ requires 473.1087 $[M+Na]^+$, found 473.1073; $\boldsymbol{\nu_{max}}$ (film)/cm^{-1} 3026, 2932, 1714, 1609, 1510, 1487, 1249; the **ee** was determined by HPLC using a Chiralpak OD-H column (*n*-hexane/*i*-PrOH, 9:1); flow rate 1.0 mL/min; τ_{major} = 21.36 min, τ_{minor} = 25.07 min (98% ee); $[\boldsymbol{\alpha}]_{\mathbf{D}}^{\mathbf{25}}$: +9.1 (*c* = 1.0, $CHCl_3$).

(*R*)-1-(4-Methoxyphenyl)-1-(4-nitrophenyl)-7-phenylheptan-3-one (4ah)

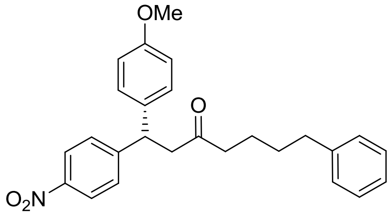

Prepared according to general procedure **C,** using 3-(ethylthio)-3-(4-nitrophenyl)propanal **1i** (72 mg, 0.30 mmol), 4-phenyl-1-butene (0.14 mL, 0.90 mmol)

and 4-methoxyphenylboronic acid (91 mg, 0.60 mmol, 2.0 equiv.). Purification by flash column chromatography (petrol/Et_2O, 85:15) afforded the *ketone* **4ah** (56 mg, 0.13 mmol, 44%, 98% ee) as a yellow oil. **δ_H** (400 MHz, $CDCl_3$): 8.14-8.05 (m, 2H, H_{Ar}), 7.37-7.32 (m, 2H, H_{Ar}), 7.28-7.20 (m, 2H, H_{Ar}), 7.18-7.05 (m, 5H, H_{Ar}), 6.83-6.78 (m, 2H, H_{Ar}), 4.64 (t, *J* = 7.5 Hz, 1H, C*H*-CH_2-CO), 3.74 (s, 3H, O-CH_3), 3.15 (dd, *J* = 14.0, 4.0 Hz, 1H, CH-C$H_a$$H_b$-CO), 3.09 (dd, *J* = 14.0, 4.5 Hz, 1H, CH-C$H_a$$H_b$-CO), 2.53 (t, *J* = 7.0 Hz, 2H, CH_2-Ph), 2.35 (t, *J* = 7.0 Hz, 2H, CH_2-CH_2-CO), 1.58-1.41 (m, 4H, 2 × CH_2); **δ_C** (101 MHz, $CDCl_3$): 208.2, 158.6, 152.2, 146.6, 142.1, 134.5, 128.8, 128.6, 128.5, 128.4, 125.9, 124.0, 114.4, 55.4, 48.5, 44.9, 43.5, 35.8, 30.9, 23.3; **LRMS** *m/z* (ESI^+) 440.2 ($[M+Na]^+$, 100%); **HRMS** (ESI^+) $C_{26}H_{47}O_4NNa$ requires 440.1832 $[M+Na]^+$, found 440.1828; **ν_{max}** (film)/cm^{-1} 2931, 1713, 1605, 1511, 1454, 1344, 1303, 1249; the **ee** was determined by HPLC using a Chiralpak OD-H column (*n*-hexane/*i*-PrOH, 7:3); flow rate 1.0 mL/min; τ_{major} = 18.00 min, τ_{minor} = 30.61 min (98% ee); $[\alpha]_D^{25}$: +9.6 (*c* = 1.0, $CHCl_3$).

(*S*)-1-(Benzyloxy)-3-(4-methoxyphenyl)-9-phenylnonan-5-one (4ai)

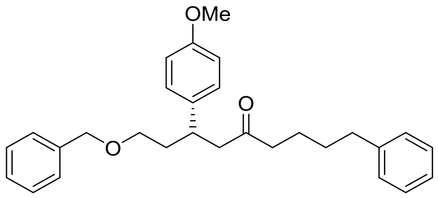

Prepared according to general procedure **C**, using 5-(benzyloxy)-3-(ethylthio)pentanal **1j** (76 mg, 0.30 mmol), 4-phenyl-1-butene (0.14 mL, 0.90 mmol) and 4-methoxyphenylboronic acid (68 mg, 0.45 mmol). Purification by flash column chromatography (petrol/Et_2O, 85:15) afforded the *ketone* **4ai** (107 mg, 0.25 mmol, 83%, 92% ee) as a yellow oil. **δ_H** (400 MHz, $CDCl_3$): 7.35-7.23 (m, 7H, H_{Ar}), 7.19-7.10 (m, 3H, H_{Ar}), 7.08 (d, *J* = 8.5 Hz, 2H, H_{Ar}), 6.80 (d, *J* = 8.5 Hz, 2H, H_{Ar}), 4.42 (d, *J* = 12.0 Hz, 1H, Ph-C$H_a$$H_b$-O), 4.38 (d, *J* = 12.0 Hz, 1H, Ph-C$H_a$$H_b$-O), 3.77 (s, 3H, OC$H_3$), 3.36-3.24 (m, 3H, C*H*-CH_2-CO and Ph-CH_2-O-CH_2), 2.70 (dd, *J* = 13.5, 5.0 Hz, 1H, CH-C$H_c$$H_d$-CO), 2.65 (dd, *J* = 13.5, 4.0 Hz, 1H, CH-C$H_c$$H_d$-CO), 2.53 (t, *J* = 7.0 Hz, 2H, CH_2-CH_2-Ph), 2.34-2.17 (m, 2H, CH_2-CH_2-CO), 2.01-1.91 (m, 1H, O-CH_2-C$H_e$$H_f$), 1.83-1.73 (m, 1H, O-$CH_2$-C$H_e$$H_f$), 1.54-1.45 (m, 4H, 2 × CH_2); **δ_C** (101 MHz, $CDCl_3$): 209.9, 158.2, 142.4, 138.6, 136.0, 128.6, 128.49, 128.46, 128.4, 127.8, 127.6, 125.8, 114.0, 73.0, 68.3, 55.3, 50.1, 43.3, 37.4, 36.5, 35.8, 31.0, 23.3; **LRMS** m/z (ESI^+) 431.3 ($[M+H]^+$, 50%), 453.2 ($[M+Na]^+$, 100%); **HRMS** (ESI^+) $C_{29}H_{34}O_3Na$ requires 453.2400 $[M+Na]^+$, found 453.2401; **ν_{max}** (film)/cm^{-1} 3028, 2933, 2858, 1712, 1611, 1512, 1454, 1366, 1248; the **ee** was determined by HPLC using a Chiralpak AD-H column (*n*-hexane/*i*-PrOH, 98:2); flow rate 1.0 mL/min; τ_{major} = 25.36 min, τ_{minor} = 29.33 min (92% ee); $[\alpha]_D^{25}$: –8.2 (c = 1.0, $CHCl_3$).

(*S*)-5-(4-Methoxyphenyl)-7-oxo-11-phenylundecyl acetate (4aj)

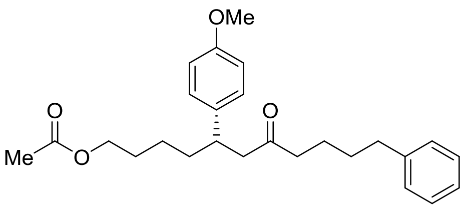

Prepared according to general procedure **C**, using 5-(ethylthio)-7-oxoheptyl acetate **1k** (46 mg, 0.20 mmol), 4-phenyl-1-butene (0.09 mL, 0.60 mmol) and

4-methoxyphenylboronic acid (46 mg, 0.30 mmol). Purification by flash column chromatography (gradient petrol/Et_2O, 9:1 to 8:2) afforded the *ketone* **4aj** (72 mg, 0.18 mmol, 87%, 93% ee) as a colorless oil. **δ_H** (400 MHz, $CDCl_3$): 7.28-7.23 (m, 2H, H_{Ar}), 7.19-7.10 (m, 3H, H_{Ar}), 7.06 (d, *J* = 8.5 Hz, 2H, H_{Ar}), 6.81 (d, *J* = 8.5 Hz, 2H, H_{Ar}), 4.02-3.91 (m, 2H, CO_2-CH_2), 3.76 (s, 3H, OCH_3), 3.11-3.02 (m, 1H, C*H*-CH_2-CO), 2.69-2.57 (m, 2H, CH-CH_2-CO), 2.54 (t, *J* = 7.0 Hz, 2H, CH_2-CH_2-Ph), 2.35-2.15 (m, 2H, CH_2-CH_2-CO), 2.00 (s, 3H, CH_3-CO), 1.63-1.47(m, 8H, 4 × CH_2), 1.26-1.09 (m, 2H, CH_2); **δ_C** (101 MHz, $CDCl_3$): 210.1, 171.3, 158.2, 142.3, 136.3, 128.5 (2C), 128.4, 125.8, 114.0, 64.4, 55.3, 50.3, 43.5, 40.4, 36.1, 35.5, 31.0, 28.5, 23.8, 23.3, 21.1; **LRMS** m/z (ESI^+) 433.2 ($[M+Na]^+$, 100%); **HRMS** (ESI^+) $C_{26}H_{34}O_4Na$ requires 433.2349 $[M+Na]^+$, found 433.2340; **ν_{max}** (film)/cm^{-1} 3027, 2935, 2859, 1736, 1712, 1611, 1512, 1455, 1365, 1245, 1179, 1035; the **ee** was determined by HPLC using a Chiralpak AD-H column (*n*-hexane/*i*-PrOH, 98:2); flow rate 1.0 mL/min; τ_{major} = 31.92 min, τ_{minor} = 36.98 min (93% ee); $[\alpha]_D^{25}$: +13.6 (c = 1.0, $CHCl_3$).

(*S*)-2-[4-(4-Methoxyphenyl)-6-oxo-10-phenyldecyl]isoindoline-1,3-dione (4ak)

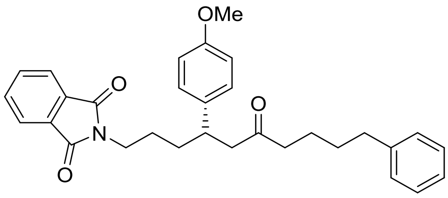

Prepared according to general procedure **C**, using 6-(1,3-dioxoisoindolin-2-yl)-3-(ethylthio)hexanal **1l** (61 mg, 0.20 mmol), 4-phenyl-1-butene (0.09 mL, 0.60 mmol) and 4-methoxyphenylboronic acid (46 mg, 0.30 mmol). Purification by flash column chromatography (gradient petrol/Et_2O, 9:1 to 7:3) afforded the *ketone* **4ak** (76 mg, 0.16 mmol, 79%, 93% ee) as a colorless oil. **δ_H** (400 MHz, $CDCl_3$): 7.83-7.78 (m, 2H, H_{Ar}), 7.72-7.66 (m, 2H, H_{Ar}), 7.28-7.22 (m, 2H, H_{Ar}), 7.18-7.09 (m, 3H, H_{Ar}), 7.05 (d, *J* = 8.5 Hz, 2H, H_{Ar}), 6.78 (d, *J* = 8.5 Hz, 2H, H_{Ar}), 3.74 (s, 3H, OCH_3), 3.61 (t, *J* = 6.5 Hz, 2H, CH_2-N), 3.13-3.05 (m, 1H, C*H*-CH_2-CO), 2.67 (dd, *J* = 16.0, 8.0 Hz, 1H, CH-C$H_a$$H_b$-CO), 2.60 (dd, *J* = 16.0, 6.5 Hz, 1H, CH-$CH_a$$H_b$-CO), 2.52 (t, *J* = 7.0 Hz, 2H, CH_2-CH_2-Ph), 2.33-2.16 (m, 2H, CH_2-CH_2-CO), 1.68-1.45 (m, 8H, 4 × CH_2); **δ_C** (101 MHz, $CDCl_3$): 209.9, 168.5, 158.2, 142.3, 135.9, 134.0, 132.2, 128.5 (2C), 128.4, 125.8, 123.3, 114.0, 55.3, 50.2, 43.5, 40.2, 38.0, 35.8, 33.8, 30.9, 26.7, 23.3; **LRMS** m/z (ESI^+) 506.2 ($[M+Na]^+$, 100%); **HRMS** (ESI^+) $C_{31}H_{34}O_4N$ requires 484.2482 $[M+H]^+$, found 484.2481; **ν_{max}** (film)/cm^{-1} 3027, 2935, 2858, 1771, 1709, 1611, 1512, 1454, 1437, 1396, 1370, 1248, 1179, 1033; the **ee** was determined by HPLC using a Chiralpak IA-3 column (*n*-hexane/*i*-PrOH, 95:5); flow rate 1.0 mL/min; τ_{major} = 42.42 min, τ_{minor} = 54.57 min (93% ee); $[\alpha]_D^{25}$: +4.1 (c = 1.0, $CHCl_3$).

Methyl 4-[(7*S*,9*S*)-9,13-dimethyl-5-oxo-1-phenyltetradec-12-en-7-yl]benzoate (4al)

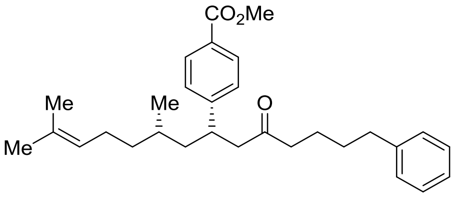

Prepared according to general procedure **C**, using (5*S*)-3-(ethylthio)-5,9-dimethyldec-8-enal **1m** (48 mg, 0.20 mmol), 4-phenyl-1-butene (0.09 mL, 0.60 mmol), 4-(methoxycarbonyl)phenylboronic acid (54 mg, 0.30 mmol) and $[Rh(L2)Cl]_2$ (4.6 mg,

0.005 mmol) in DCE. The conjugate addition step was carried out with additional K_2CO_3 (14 mg, 0.10 mmol, 0.5 equiv.); dr >20:1. Purification by flash column chromatography (petrol/Et_2O, 94:6) afforded the *ketone* **4al** (59 mg, 0.13 mmol, 66%, 93% ee) as a colorless oil. **δ_H** (400 MHz, $CDCl_3$): 7.94 (d, *J* = 8.5 Hz, 2H, H_{Ar}), 7.28-7.22 (m, 4H, H_{Ar}), 7.19-7.13 (m, 1H, H_{Ar}), 7.11 (d, *J* = 8.5 Hz, 2H, H_{Ar}), 5.06-4.99 (m, 1H, C*H*=C(CH_3)$_2$), 3.89 (s, 3H, CO_2CH_3), 3.37-3.29 (m, 1H, C*H*-CH_2-CO), 2.72-2.58 (m, 2H, CH-CH_2-CO), 2.53 (t, *J* = 7.0 Hz, 2H, CH_2-CH_2-Ph), 2.35-2.16 (m, 2H, CH_2-CH_2-CO), 2.03-1.92 (m, 1H, C$H_a$$H_b$-CH=C($CH_3$)$_2$), 1.88-1.76 (m, 1H, CH_aH_b-CH=C(CH_3)$_2$), 1.66 (s, 3H, (CH_3)$_a$-C=CH), 1.57 (s, 3H, (CH_3)$_b$-C=CH), 1.55-1.38 (m, 7H, C*H*-CH_3 and 3 × CH_2), 1.24-1.14 (m, 1H, CH_2-C$H_c$$H_d$-CH-$CH_3$), 1.13-1.04 (m, 1H, CH_2-CH_cH_d-CH-CH_3), 0.80 (d, *J* = 6.5 Hz, 3H, CH-CH_3); **δ_C** (101 MHz, $CDCl_3$): 209.5, 167.1, 150.7, 142.3, 131.4, 129.9, 128.5, 128.40, 128.35, 127.7, 125.9, 124.8, 52.1, 49.8, 44.0, 43.5, 38.8, 36.1, 35.8, 30.9, 29.9, 25.8, 25.2, 23.3, 20.3, 17.8; **LRMS** m/z (ESI$^+$) 471.3 ([M+Na]$^+$, 100%); **HRMS** (ESI$^+$) $C_{30}H_{40}O_3Na$ requires 471.2870 [M+Na]$^+$, found 471.2867; **ν_{max}** (film)/cm^{-1} 2951, 2915, 2852, 1721, 1610, 1435, 1278, 1180, 1113, 1019; the **ee** was determined by HPLC using two Chiralpak AD-H columns in series (*n*-hexane/*i*-PrOH, 99:1); flow rate 0.8 mL/min; τ_{major} = 61.81 min, τ_{minor} = 85.69 min (>99% ee); $[\alpha]_D^{25}$: −1.2 (c = 1.0, $CHCl_3$).

Methyl 4-[(7*R*,9*S*)-9,13-dimethyl-5-oxo-1-phenyltetradec-12-en-7-yl]benzoate (4am)

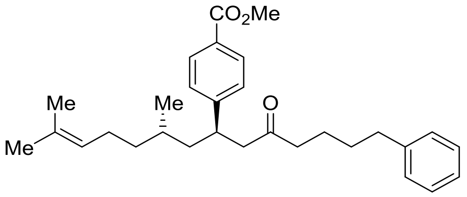

Prepared according to general procedure **C**, using (5*S*)-3-(ethylthio)-5,9-dimethyldec-8-enal **1m** (48 mg, 0.20 mmol), 4-phenyl-1-butene (0.09 mL, 0.60 mmol). The conjugate addition step was carried out in a microwave reaction vial charged with 4-(methoxycarbonyl)phenylboronic acid (108 mg, 0.60 mmol, 3.0 equiv.), K_2CO_3 (28 mg, 0.20 mmol, 1.0 equiv.), Rh(nbd)$_2$BF$_4$ (3.7 mg, 0.01 mmol, 0.05 equiv.) and (*S*)-BINAP (6.2 mg, 0.01 mmol, 0.05 equiv.) at 55 °C for 4 h; dr = 8:1. Purification by flash column chromatography (petrol/Et_2O, 94:6) afforded the *ketone* **4am** (56 mg, 0.12 mmol, 62%, 99% ee) as a colorless oil. **δ_H** (400 MHz, $CDCl_3$): 7.87 (d, *J* = 8.5 Hz, 2H, H_{Ar}), 7.21-7.14 (m, 4H, H_{Ar}), 7.12-7.06 (m, 1H, H_{Ar}), 7.04 (d, *J* = 7.0 Hz, 2H, H_{Ar}), 4.91-4.84 (m, 1H, C*H*=C(CH_3)$_2$), 3.82 (s, 3H, ArCO_2CH_3), 3.31-3.22 (m, 1H, C*H*-CH_2-CO), 2.61 (dd, *J* = 16.5, 7.5 Hz, 1H, CH-C$H_a$$H_b$-CO), 2.53 (dd, *J* = 16.5, 6.5 Hz, 1H, CH-CH_aH_b-CO), 2.46 (t, *J* = 7.0 Hz, 2H, CH_2-CH_2-Ph), 2.29-2.08 (m, 2H, CH_2-CH_2-CO), 1.83-1.69 (m, 2H, CH_2-CH=C(CH_3)$_2$), 1.54 (s, 3H, (CH_3)$_a$-C=CH), 1.46 (s, 3H, (CH_3)$_b$-C=CH), 1.44-0.95 (m, 9H, C*H*-CH_3 and 4 × CH_2), 0.79 (d, *J* = 6.0 Hz, 3H, CH-CH_3); **δ_C** (101 MHz, $CDCl_3$): 209.4, 167.1, 150.2, 142.2, 131.3, 129.9, 128.5, 128.4, 127.8, 127.7, 125.9, 124.7, 52.1, 50.5, 43.5, 43.1, 38.8, 37.9, 35.5, 30.9, 29.9, 25.8, 25.4, 23.3, 19.1, 17.7; the **ee** was determined by HPLC using two Chiralpak AD-H columns in series (*n*-hexane/*i*-PrOH, 99:1); flow rate 0.8 mL/min; τ_{major} = 75.33 min, τ_{minor} = 59.12 min (>99% ee).

4. Product derivatization

(*S*,*E*)-7-(4-Methoxyphenyl)-2,2-dimethyldodec-3-en-5-one (5a)

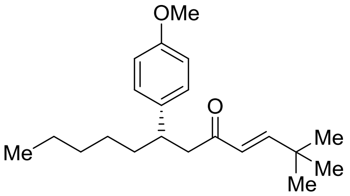

Prepared according to general procedure **C**, using 3-(ethylthio)octanal **1a** (56 mg, 0.30 mmol), 3,3-dimethyl-1-butyne (48 μL, 0.39 mmol, 1.3 equiv.) and 4-methoxyphenylboronic acid (68 mg, 0.45 mmol). Purification by flash column chromatography (petrol/Et_2O, 96:4) afforded the *enone* **5a** (87 mg, 0.27 mmol, 92%, 94% ee) as a pale yellow oil. δ_H (400 MHz, $CDCl_3$): 7.09 (d, *J* = 8.5 Hz, 2H, H_{Ar}), 6.82 (d, *J* = 8.5 Hz, 2H, H_{Ar}), 6.68 (d, *J* = 16.0 Hz, 1H, CO-CH=C*H*), 5.89 (d, *J* = 16.0 Hz, 1H, CO-C*H*), 3.77 (s, 3H, OCH_3), 3.13-3.04 (m, 1H, C*H*-CH_2-CO), 2.80 (dd, *J* = 14.0, 6.0 Hz, 1H, CH-C$H_a$$H_b$-CO), 2.75 (dd, *J* = 14.0, 5.5 Hz, 1H, CH-C$H_a$$H_b$-CO), 1.68-1.48 (m, 2H, CH-CH_2-CH_2), 1.27-1.07 (m, 6H, 3 × CH_2), 1.02 (s, 9H, C(CH_3)$_3$), 0.82 (t, *J* = 7.0 Hz, 3H, CH_2-CH_3); δ_C (101 MHz, $CDCl_3$): 200.6, 158.0, 157.2, 137.1, 128.5, 125.9, 113.9, 55.3, 48.1, 41.0, 36.5, 33.8, 31.9, 28.8, 27.2, 22.6, 14.2; **LRMS** m/z (ESI$^+$) 317.2 ([M+H]$^+$, 80%), 339.2 ([M+Na]$^+$, 100%); **HRMS** (ESI$^+$) $C_{21}H_{33}O_2$ requires 317.2475 [M+H]$^+$, found 317.2478; ν_{max} (film)/cm^{-1} 2958, 2929, 2860, 1672, 1622, 1513, 1463, 1365, 1301, 1247, 1178, 1039; the **ee** was determined by HPLC using a Chiralpak AD-H column (*n*-hexane/*i*-PrOH, 99:1); flow rate 1.0 mL/min; τ_{major} = 9.98 min, τ_{minor} = 11.22 min (94% ee); $[\alpha]_D^{25}$: +9.1 (c = 1.0, $CHCl_3$).

(*S*,*E*)-8-(4-Methoxyphenyl)-5-propyltridec-4-en-6-one (5b)

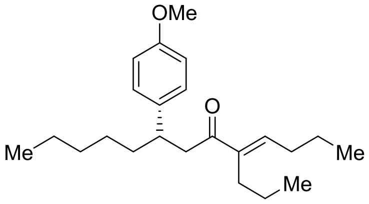

Prepared according to general procedure **C**, using 3-(ethylthio)octanal **1a** (56 mg, 0.30 mmol), 4-octyne (57 mL, 0.39 mmol, 1.3 equiv.) and 4-methoxyphenylboronic acid (68 mg, 0.45 mmol). Purification by flash column chromatography (gradient petrol/Et_2O, 99:1 to 97:3) afforded the *enone* **5b** (54 mg, 0.16 mmol, 52%, 93% ee) as a colorless oil. δ_H (400 MHz, $CDCl_3$): 7.08 (d, *J* = 8.5 Hz, 2H, H_{Ar}), 6.81 (d, *J* = 8.5 Hz, 2H, H_{Ar}), 6.48 (d, *J* = 7.5 Hz, 1H, CO-C=C*H*), 3.77 (s, 3H, OCH_3), 3.15-3.06 (m, 1H, C*H*-CH_2-CO), 2.91 (dd, *J* = 15.5, 7.0 Hz, 1H, CH-C$H_a$$H_b$-CO), 2.82 (dd, *J* = 15.5, 7.0 Hz, 1H, CH-C$H_a$$H_b$-CO), 2.22-2.13 (m, 4H, CH_2-C=CH-CH_2), 1.65-1.48 (m, 2H, CH_2-CH-CH_2-CO), 1.48-1.39 (m, 2H, C=CH-CH_2-CH_2), 1.28-1.06 (m, 8H, 4 × CH_2), 0.93 (t, *J* = 7.5 Hz, 3H, C=CH-CH_2-CH_2-CH_3), 0.82 (t, *J* = 7.5 Hz, 6H, 2 × CH_2-CH_3); δ_C (101 MHz, $CDCl_3$): 201.2, 158.0, 142.8, 142.5, 137.4, 128.6, 113.8, 55.3, 45.1, 41.5, 36.5, 31.9, 31.0, 27.8, 27.3, 22.7, 22.5, 22.3, 14.3, 14.2, 14.1; **LRMS** m/z (ESI$^+$) 367.2 ([M+Na]$^+$, 100%); **HRMS** (ESI$^+$) $C_{23}H_{36}O_2Na$ requires 367.2608 [M+Na]$^+$, found 367.2608; ν_{max} (film)/cm^{-1} 2958, 2929, 2871, 1667, 1512, 1463, 1378, 1247, 1178, 1103, 1039; the **ee** was determined by HPLC using a Chiralpak IA-3 column (*n*-hexane/*i*-PrOH, 99:1); flow rate 1.0 mL/min; τ_{major} = 5.69 min, τ_{minor} = 6.32 min (93% ee); $[\alpha]_D^{25}$: –13.3 (c = 1.0, $CHCl_3$).

Dimethyl 4,4'-[(1*R*,5*S*)-1-cyclohexyl-3-oxodecane-1,5-diyl]dibenzoate (5c)

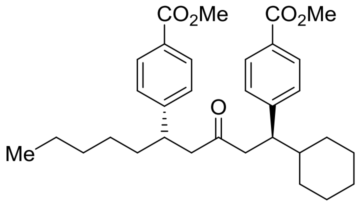

Prepared according to general procedure **C**, using 3-(ethylthio)octanal **1a** (56 mg, 0.30 mmol), ethynylcyclohexane (51 µL, 0.39 mmol, 1.3 equiv.), 4-methoxycarbonylphenylboronic acid (162 mg, 0.90 mmol, 3.0 equiv.) and $[Rh(L2)Cl]_2$ (4.6 mg, 0.005 mmol) in DCE. The conjugate addition step was carried out with additional K_2CO_3 (41 mg, 0.30 mmol, 1.0 equiv.) for 6 h; dr > 20:1. Purification by flash column chromatography (petrol/Et_2O, 9:1) afforded the *ketone* **5c** (113 mg, 0.22 mmol, 74%, >99% ee) as a white solid. **M.p.** (Et_2O): 113-117 °C; δ_H (400 MHz, $CDCl_3$): 7.91 (app. dd, *J* = 8.5, 1.5 Hz, 4H, H_{Ar}), 7.13 (app. dd, *J* = 8.5, 5.5 Hz, 4H, H_{Ar}), 3.89 (s, 6H, 2 × CO_2CH_3), 3.11-3.02 (m, 1H, CH_2-C*H*-CH_2-CO), 2.93 (td, *J* = 8.0, 6.0 Hz, 1H, Cy-C*H*-CH_2-CO), 2.72-2.41 (m, 4H, CH_2-CO-CH_2), 1.69-1.52 (m, 4H, H_{Cy}), 1.41-0.90 (m, 13H, C*H*-CH-CH_2-CO and 6 × CH_2), 0.84-0.63 (m, 5H, H_{Cy} and CH_2-CH_3); δ_C (101 MHz, $CDCl_3$): 208.3, 167.1 (2C), 150.4, 149.4, 129.9, 129.6, 128.5, 128.33, 128.28, 127.7, 52.1, 50.6, 47.2, 46.9, 42.8, 40.9, 36.1, 31.7, 31.1, 30.8, 27.0, 26.4, 26.3, 22.5, 14.1; **LRMS** m/z (ESI$^+$) 475.2 ([M-OMe]$^+$, 100%), 529.2 ([M+Na]$^+$, 25%); **HRMS** (ESI$^+$) $C_{32}H_{42}O_5Na$ requires 529.2924 [M+Na]$^+$, found 529.2917; ν_{max} (film)/cm^{-1} 2928, 2854, 1720, 1610, 1435, 1279, 1181, 1112; the **ee** was determined by HPLC using a Chiralpak AD-H column (*n*-hexane/*i*-PrOH, 97:3); flow rate 1.0 mL/min; τ_{major} = 54.84 min, τ_{minor} = 41.98 min (>99% ee); $[\alpha]_D^{25}$: +35.0 (c = 1.0, $CHCl_3$).

(*S*)-6-[2-(4-Methoxyphenyl)heptyl]-2,3,4,5-tetrahydropyridine (6a)

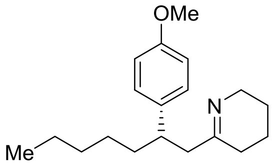

Trifluoroacetic acid (0.13 mL, 1.72 mmol, 20.0 equiv.) was added to a solution of (*S*)-*N*,*N*-di-Boc-1-amino-7-(4-methoxyphenyl)dodecan-5-one **4w** (44 mg, 0.086 mmol, 1.0 equiv.) in CH_2Cl_2 (0.5 mL, 0.16 M). The resulting solution was stirred at 22 °C for 4 h and cooled to 0 °C. The pH was adjusted to 12-13 using 1 M NaOH (aq.), and the mixture was extracted with CH_2Cl_2 (4 × 15 mL). The combined organic layers were washed with brine, dried over $MgSO_4$, filtered and concentrated under reduced pressure. Purification by flash column chromatography (1 drop triethylamine/100mL acetone) afforded the *imine* **6a** (21 mg, 0.073 mmol, 85%, 98% ee) as a yellow oil. δ_H (400 MHz, $CDCl_3$): 7.07 (d, *J* = 8.5 Hz, 2H, H_{Ar}), 6.82 (d, *J* = 8.5 Hz, 2H, H_{Ar}), 3.78 (s, 3H, OCH_3), 3.55-3.45 (m, 2H, CH_2-N), 2.91-2.82 (m, 1H, C*H*-CH_2-C=N), 2.44 (dd, *J* = 13.5, 7.0 Hz, 1H, CH-C$H_a$$H_b$-C=N), 2.30 (dd, *J* = 13.5, 8.5 Hz, 1H, CH-C$H_a$$H_b$-C=N), 1.94 (dt, *J* = 13.0, 6.5 Hz, 1H, CH_2-C$H_c$$H_d$-C=N), 1.78 (dt, *J* = 13.0, 6.0 Hz, 1H, CH_2-C$H_c$$H_d$-C=N), 1.65-1.40 (m, 6H, 3 × CH_2), 1.24-1.06 (m, 6H, 3 × CH_2), 0.81 (t, *J* = 6.5 Hz, 3H, CH_2-CH_3); δ_C (101 MHz, $CDCl_3$): 170.5, 157.9, 137.3, 128.6, 113.7, 55.3, 49.3, 48.8, 43.2, 36.5, 32.0, 30.2, 27.2, 22.7, 21.9, 19.7, 14.2; **LRMS** m/z (ESI$^+$) 288.2 ([M+H]$^+$, 100%); **HRMS** (ESI$^+$) $C_{19.}H_{30}ON$ requires 288.2322

$[M+H]^+$, found 288.2319; ν_{max} (film)/cm^{-1} 2928, 2853, 1658, 1611, 1512, 1464, 1246, 1178, 1039; the **ee** was determined by HPLC using a Chiralpak AD-H column (*n*-hexane/*i*-PrOH, 96:4); flow rate 1.0 mL/min; τ_{major} = 8.21 min, τ_{minor} = 7.33 min (98% ee); $[\alpha]_D^{25}$: +42.3 (c = 1.0, $CHCl_3$).

(*S*)-3-(4-Methoxyphenyl)-5-[(*S*)-2-(4-methoxyphenyl)heptyl]-3,4-dihydro-2*H*-pyrrole (6b)

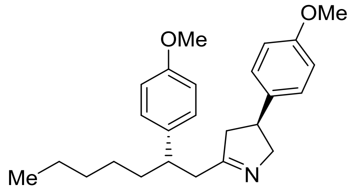

Prepared according to general procedure **C**, using 3-(ethylthio)octanal **1a** (38 mg, 0.20 mmol), di-*tert*-butyl prop-2-ynylimidodicarbonate **SM1c** (66 mg, 0.26 mmol, 1.3 equiv.) and 4-methoxyphenylboronic acid (122 mg, 0.80 mmol, 4.0 equiv.). The hydroacylation was carried out, using PNP(Cy) (4.2 mg, 0.01 mmol, 5 mol%) instead of dcpm as the ligand, at 22 °C for 3 h. The filtrate was concentrated under reduced pressure and dissolved in CH_2Cl_2 (2.0 mL), followed by the addition of trifluoroacetic acid (0.31 mL, 4.0 mmol, 20.0 equiv.). The resulting mixture was stirred at 22 °C for 4 h and cooled to 0 °C. The pH was adjusted to 12-13 using 1 M NaOH (aq.), and the mixture was extracted with CH_2Cl_2 (3 × 2 mL). The combined organic layers were dried over $MgSO_4$, filtered and concentrated under reduced pressure; dr = 17:1. Purification by flash column chromatography (gradient petrol/EtOAc, 9:1 to 7:3) afforded the *imine* **6b** (55 mg, 0.14 mmol, 72%, >99% ee) as a yellow oil. δ_H (400 MHz, $CDCl_3$): 7.10 (d, *J* = 8.5 Hz, 2H, H_{Ar}), 6.87 (d, *J* = 8.5 Hz, 2H, H_{Ar}), 6.83 (d, *J* = 8.5 Hz, 2H, H_{Ar}), 6.76 (d, *J* = 8.5 Hz, 2H, H_{Ar}), 4.15 (dd, *J* = 15.5, 8.5 Hz, 1H, CH_aH_b-N), 3.85-3.70 (m, 7H, 2 × OCH_3 and CH_aH_b-N), 3.34-3.25 (m, 1H, CH-CH_2-N), 2.99-2.90 (m, 1H, CH_2-CH_2-CH-CH_2), 2.79 (dd, *J* = 17.5, 9.5 Hz, 1H, CH_cH_d-CH-CH_2-N), 2.71 (dd, *J* = 14.0, 6.5 Hz, 1H, CH_2-CH_2-CH-CH_eH_f), 2.61 (dd, *J* = 14.0, 9.0 Hz, 1H, CH_2-CH_2-CH-CH_eH_f), 2.28 (dd, *J* = 17.5, 6.5 Hz, 1H, CH_cH_d-CH-CH_2-N), 1.70-1.49 (m, 2H, CH_2-CH_2-CH-CH_2), 1.28-1.11 (m, 6H, 3 × CH_2), 0.82 (t, *J* = 7.0 Hz, 3H, CH_2-CH_3); δ_C (101 MHz, $CDCl_3$): 177.0, 158.07, 158.06, 137.5, 137.0, 128.6, 127.7, 114.0, 113.9, 69.1, 55.4, 55.3, 47.3, 43.1, 41.9, 41.2, 37.1, 31.9, 27.2, 22.7, 14.2; **LRMS** m/z (ESI^+) 380.2 ($[M+H]^+$, 100%); **HRMS** (ESI^+) $C_{25}H_{34}O_2N$ requires 380.2584 $[M+H]^+$, found 380.2589; ν_{max} (film)/cm^{-1} 2928, 2856, 1642, 1611, 1584, 1512, 1463, 1301, 1246, 1178, 1037; the **ee** was determined by HPLC using a Chiralpak AD-H column (*n*-hexane/*i*-PrOH, 97:3); flow rate 1.0 mL/min; τ_{major} = 30.31 min, τ_{minor} = 21.10 min (>99% ee); $[\alpha]_D^{25}$: +113.8 (c = 1.0, $CHCl_3$).

(4*S*)-2-Methoxy-4-pentyl-2-(4-phenylbutyl)chroman (6c)

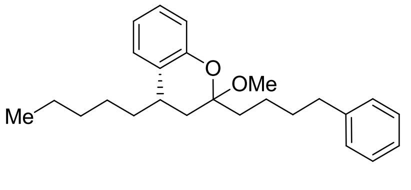

Prepared according to general procedure **C**, using 3-(ethylthio)octanal **1a** (56 mg, 0.30 mmol), 4-phenyl-1-butene (0.14 mL, 0.90 mmol) and 2-hydroxyphenylboronic acid (83 mg, 0.60 mmol, 2.0 equiv.). Purification by flash column chromatography (petrol/Et_2O,

97:3) afforded the ketone (102 mg, 0.29 mmol), that was dissolved in methanol (5.8 mL). Camphorsulfonic acid (13 mg, 0.058 mmol, 0.2 equiv.) was added and the mixture stirred at 22 °C for 16 h, then diluted with Et_2O (20 mL) and quenched with sat. $NaHCO_3$ (aq.) (10 mL). The aqueous phase was extracted with Et_2O (3 × 20 mL), dried over $MgSO_4$, filtered and concentrated under reduced pressure; dr = 6:1. Purification by flash column chromatography (petrol/Et_2O, 9:1) afforded the *desired product* **6c** (73 mg, 0.20 mmol, 69%) as a colorless oil. $\boldsymbol{\delta_H}$ (400 MHz, $CDCl_3$, major diasteromer described only): 7.34-7.23 (m, 3H, H_{Ar}), 7.21-7.14 (m, 3H, H_{Ar}), 7.09 (app. t, J = 7.5 Hz, 1H, H_{Ar}), 6.91 (app. td, J = 7.5, 1.0 Hz, 1H, H_{Ar}), 6.87-6.79 (m, 1H, H_{Ar}), 3.17 (s, 3H, O-CH_3), 3.03-2.97 (m, 1H, CH), 2.68-2.63 (m, 2H, CH_2-Ph), 2.13 (dd, J = 13.5, 6.0 Hz, 1H, CH-CH_aH$_b$-CO), 2.02-1.91 (m, 2H, CH-CH$_a$$H_b$-CO + CH$_2$-C$H_aH_b$-CO), 1.81-1.65 (m, 3H, CH$_2$-CH_aH$_b$-CO + CH_2), 1.50-1.40 (m, 4H, 2 × CH_2), 1.29-1.25 (m, 6H, 3 × CH_2), 0.91-0.88 (m, 3H, CH$_2$-CH_3); $\boldsymbol{\delta_C}$ (101 MHz, $CDCl_3$, major diasteromer described only): 152.3, 142.6, 128.54, 128.46, 127.2, 127.1, 126.8, 125.9, 120.9, 117.1, 100.2, 48.7, 36.0, 35.7, 35.2, 33.8, 32.4, 31.9, 29.7, 26.0, 23.5, 22.8, 14.3; **LRMS** *m/z* (ESI$^+$) 389.2 ([M+Na]$^+$, 100%); **HRMS** (ESI$^+$) $C_{25}H_{34}O_2N$ requires 389.2451 [M+H]$^+$, found 389.2454; $\boldsymbol{\nu_{max}}$ (film)/cm^{-1} 2928, 2856, 1581, 1487, 1453, 1297, 1223, 1187, 1143, 1050; $[\boldsymbol{\alpha}]_{\mathbf{D}}^{\mathbf{25}}$: −2.5 (*c* = 0.5, $CHCl_3$).

6-[(4*S*)-2-Methoxy-4-pentylchroman-2-yl]hexan-2-one (6d)

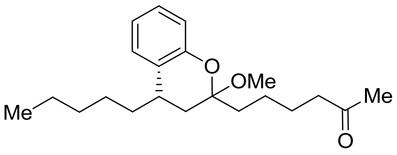

Prepared according to general procedure **C**, using 3-(ethylthio)octanal **1a** (85 mg, 0.45 mmol), 5-hexen-2-one (0.16 mL, 1.35 mmol) and 2-hydroxyphenylboronic acid (124 mg, 0.90 mmol, 2.0 equiv.). Purification by flash column chromatography (petrol/Et_2O, 9:1) afforded the ketone (72 mg, 0.23 mmol), that was dissolved in methanol (4.4 mL). Camphorsulfonic acid (16 mg, 0.069 mmol, 0.3 equiv.) was added and the mixture stirred at 22 °C for 16 h, then diluted with Et_2O (20 mL) and quenched with sat. $NaHCO_3$ (aq.) (10 mL). The aqueous phase was extracted with Et_2O (3 × 20 mL), dried over $MgSO_4$, filtered and concentrated under reduced pressure; dr = 6:1. Purification by flash column chromatography (petrol/Et_2O, 9:1) afforded the *desired product* **6d** (55 mg, 0.17 mmol, 55%, 93% ee) as a yellow oil. $\boldsymbol{\delta_H}$ (400 MHz, $CDCl_3$, major diasteromer described only): 7.27-7.25 (m, 1H, H_{Ar}), 7.10 (app. tdd, J = 8.0, 1.5, 1.0 Hz, 1H, H_{Ar}), 6.92 (app. td, J = 7.5, 1.5 Hz, 1H, H_{Ar}), 6.82 (dd, J = 8.0, 1.5 Hz, 1H, H_{Ar}), 3.18 (s, 3H, O-CH_3), 3.08-2.99 (m, 1H, CH), 2.49 (t, J = 7.5 Hz, 2H, CH-CH_2-CO), 2.16 (s, 3H, CO-CH_3), 2.02-1.91 (m, 2H, CH_2), 1.81-1.59 (m, 4H, 2 × CH_2), 1.49-1.25 (m, 10H, 5 × CH_2), 0.92-0.88 (m, 3H, CH$_2$-CH_3); $\boldsymbol{\delta_C}$ (101 MHz, $CDCl_3$, major diasteromer described only): 209.0, 152.2, 127.3, 127.1, 126.8, 121.0, 117.1, 100.0, 48.7, 43.7, 35.7, 35.1, 33.7, 32.3, 30.1, 29.7, 26.1, 24.1, 23.4, 22.8, 14.2; **LRMS** *m/z* (ESI$^+$) 355.2 ([M+Na]$^+$, 100%); **HRMS** (ESI$^+$) $C_{21}H_{32}O_3Na$ requires 355.2244 [M+Na]$^+$, found 355.2243; $\boldsymbol{\nu_{max}}$ (film)/cm^{-1} 2930, 2859, 1716, 1581, 1487, 1453, 1297, 1186, 1159. the **ee** was determined by HPLC using a Chiralpak IA-3 column (*n*-hexane/*i*-PrOH, 99:1); flow rate 0.9 mL/min; τ_{major} = 8.21 min, τ_{minor} = 10.31 min (93% ee); $[\boldsymbol{\alpha}]_{\mathbf{D}}^{\mathbf{25}}$: −4.4 (*c* = 1.0, $CHCl_3$).

(2*R*,4*S*)-4-Pentyl-2-(4-phenylbutyl)chroman (6e)

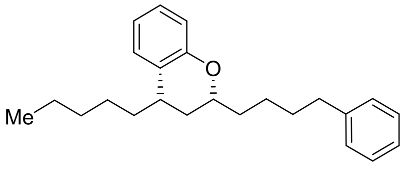

Triethylsilane (0.06 mL, 0.37 mmol, 3.1 equiv.) was added to a solution of (4*S*)-2-methoxy-4-pentyl-2-(4-phenylbutyl)chroman **6c** (44 mg, 0.12 mmol, 1.0 equiv.) in anhydrous CH_2Cl_2 (3.0 mL). Boron trifluoride diethyl etherate (0.04 mL, 0.36 mmol, 3.0 equiv.) was then added dropwise and the reaction mixture was stirred at 22 °C for 3 h. The reaction was quenched with sat. $NaHCO_3$ (aq.) (5 mL) and extracted with CH_2Cl_2 (3 × 10 mL). The combined layer was dried over $MgSO_4$, filtered and concentrated under reduced pressure; dr > 20:1. Purification by flash column chromatography (petrol/Et_2O, 98:2) afforded the *desired product* **6e** (34 mg, 0.10 mmol, 85%, 91% ee) as a colorless oil. δ_H (400 MHz, $CDCl_3$): 7.32-7.28 (m, 2H, H_{Ar}), 7.26-7.18 (m, 4H, H_{Ar}), 7.11-7.07 (m, 1H, H_{Ar}), 6.88 (app. td, *J* = 7.5, 1.5 Hz, 1H, H_{Ar}), 6.81 (dd, *J* = 8.0, 1.5 Hz, 1H, H_{Ar}), 3.98-3.92 (m, 1H, C*H*-O), 2.93-2.89 (m, 1H, C*H*-Ar), 2.68 (t, *J* = 7.5 Hz, 2H, CH_2-Ph), 2.05 (ddd, *J* = 13.5, 6.0, 1.5 Hz, 1H, CH-CH_aH$_b$-CH), 2.02-1.91 (m, 1H, Ar-CH-CH_aH$_b$-CH$_2$), 1.83-1.61 (m, 4H, 2 × CH_2), 1.57-1.27 (m, 10H, 4 × CH_2 and CH-CH$_a$$H_b$-CH + Ar-CH-CH$_a$$H_b$-CH$_2$), 0.92 (t, *J* = 6.5 Hz, 3H, CH_3); δ_C (101 MHz, $CDCl_3$: 155.5, 142.8, 128.6, 128.4, 127.3, 127.1, 126.8, 125.8, 120.3, 116.9, 76.0, 36.0, 36.0, 34.9, 34.4 (2C), 32.3, 31.7, 26.1, 25.1, 22.8, 14.3; **LRMS** *m/z* (ESI$^+$) 337.2 ([M+H]$^+$, 10%), 359.2 ([M+Na]$^+$, 100%); **HRMS** (ESI$^+$) $C_{24}H_{32}ONa$ requires 359.2345 [M+Na]$^+$, found 359.2344; ν_{max} (film)/cm^{-1} 2929, 2857, 1606, 1579, 1486, 1454, 1379, 1296, 1233. the **ee** was determined by HPLC using a Chiralpak OD-H column (*n*-hexane/*i*-PrOH, 98:2); flow rate 0.5 mL/min; τ_{major} = 10.63 min, τ_{minor} = 10.03 min (91% ee); $[\alpha]_D^{25}$: +57.0 (*c* = 1.0, $CHCl_3$).

5. Determination of the absolute configuration

The absolute configuration was determined by first synthesising the reported (*S*)-4-(phenylnonan-2-one). This can then be alkylated into (*S*)-1,7-diphenyldodecan-5-one which has also been prepared using our sequential procedure (ketone **4e**). The absolute configuration was determined by comparison of the optical rotation values.

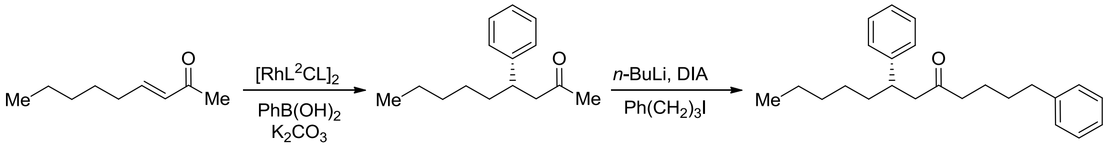

(*S*)-4-Phenylnonan-2-one:

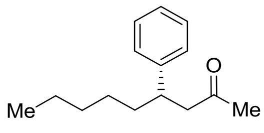

A mixture of 3-nonen-2-one (140 mg, 1.0 mmol, 1.0 equiv.), phenylboronic acid (244 mg, 2.0 mmol, 2.0 equiv.), K_2CO_3 (69 mg, 0.5 mmol, 0.5 equiv.) and [Rh(L2)Cl]$_2$ (18 mg, 0.02 mmol, 0.02 equiv.) in acetone/methanol (10 mL, 9:1) was stirred at 55 °C for 3 h then filtered through a pad of SiO_2 and concentrated under reduced pressure. Purification by flash column chromatography (petrol/Et_2O, 95:5) afforded the ketone (216 mg, 1.0

mmol, 99%, 98% ee) as a colorless oil. **δ_H** (400 MHz, $CDCl_3$): 7.25-7.21 (m, 2H, H_{Ar}), 7.17-7.11 (m, 3H, H_{Ar}), 3.12-3.02 (m, 1H, C*H*), 2.67 (dd, *J* = 7.0, 2.0 Hz, 2H, CH-CH_2-CO), 1.97 (s, 3H, CO-CH_3), 1.60-1.48 (m, 2H, CH_2), 1.20-1.13 (m, 6H, 3 × CH_2), 0.78 (t, *J* = 7.0 Hz, 3H, CH_3); **δ_C** (101 MHz, $CDCl_3$): 208.2, 144.7, 128.6, 127.6, 126.4, 51.1, 41.4, 36.6, 31.9, 30.8, 27.2, 22.6, 14.2; **LRMS** *m/z* (ESI^+) 241.1 ($[M+Na]^+$, 100%). The data is consistent with the literature.[12a]

The **ee** was determined by HPLC using a Chiralpak OD-H column (*n*-hexane/*i*-PrOH, 92:8); flow rate 0.3 mL/min; τ_{major} = 16.3 min, τ_{minor} = 17.4 min (98% ee). Reported values:[12b] Chiralpak OD-H column with (*n*-hexane/*i*-PrOH, 100:1), flow rate 0.5 mL/min; τ_{major} = 22.0 min [(*S*)-enantiomer], τ_{minor} = 23.1 min [(*R*)-enantiomer], 98.8% ee.

$[\alpha]_D^{25}$: +18.7 (*c* = 1.0, $CHCl_3$); (Reported values: $[\alpha]_D^{20}$: +16.9 (*c* = 0.5, $CHCl_3$, 92% ee for (*S*)-enantiomer)[12c]; $[\alpha]_D^{20}$: −17.0 (*c* = 1.3, $CHCl_3$, 92% ee for (*R*)-enantiomer).[12a,d]

(*S*)-1,7-Diphenyldodecan-5-one (4e)

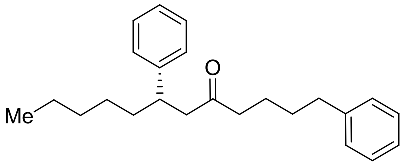

n-Butyllithium (1.6 M in hexane, 262 μL, 0.42 mmol, 1.05 equiv.) was added dropwise to a solution of diisopropylamine (70 μL, 0.48 mmol, 1.20 equiv.) in anhydrous THF (1.4 mL) at 0 °C. The mixture was stirred for 15 min and then added dropwise to a solution of (*S*)-1,7-diphenyldodecan-5-one (88 mg, 0.40 mmol, 1.00 equiv.) in THF (1.0 mL) . The reaction mixture was warmed up to room temperature, stirred for 1 h and cooled down to 0 °C. 1-Iodo-3-phenylpropane (70 μL, 0.22 mmol, 1.10 equiv.) was added dropwise and the solution stirred at 22 °C overnight. Sat. NH_4Cl (aq.) was added (5 mL) and the product extracted with Et_2O (3 × 10 mL). The combined organic layers were dried over $MgSO_4$, filtered and concentrated under reduced pressure. Purification by flash column chromatography (petrol/Et_2O, 99:1) afforded the *ketone* (11 mg, 0.032 mmol, 8%, 98% ee) as a colorless oil. The data is consistent with the one described for compound **4e**. The **ee** was determined by HPLC using a Chiralpak IA-3 column (*n*-hexane/*i*-PrOH, 98:2); flow rate 0.5 mL/min; τ_{major} = 11.42 min for [(*S*)-enantiomer], τ_{minor} = 11.95 min [(*R*)-enantiomer] (98% ee); $[\alpha]_D^{25}$: +17.4 (*c* = 0.5, $CHCl_3$) compared to $[\alpha]_D^{25}$: +17.9 (*c* = 0.5, $CHCl_3$) obtained for ketone **4e**.

6. References

[1] a) K. Okamoto, T. Hayashi, V. H. Rawal, *Chem. Commun.* **2009**, 4815; b) M. M. Hansmann, A. S. K. Hashmi, M. Lautens, *Org. Lett.* **2013**, *15*, 3226; c) I. D. Roy, A. R. Burns, G. Pattison, B. Michel, A. J. Parker, H. W. Lam, *Chem. Commun.* **2014**, *50*, 2865.

[2] I. Pernik, J. F. Hooper, A. B. Chaplin, A. S. Weller, M. C. Willis, *ACS Catal.* **2012**, *2*, 2779.

[3] A. M. Whittaker, G. Lalic, *Org. Lett.* **2013**, *15*, 1112.

[4] T. P. Boyle, J. B. Bremner, J. A. Coates, P. A. Keller, S. G. Pyne, *Tetrahedron* **2005**, *61*, 7271.

[5] P. K. Sasmal, A. Chandrasekhar, S. Sridhar, J. Iqbal, *Tetrahedron* **2008**, *64*, 11074.

[6] L. Palais, L. Babel, A. Quintard, S. Belot, A. Alexakis, *Org. Lett.* **2010**, *12*, 1988.

[7] J. Stiller, E. Marqués-López, R. P. Herrera, R. Fröhlich, C. Strohmann, M. Christmann, *Chem. Commun.* **2011**, *13*, 70.

[8] M. Yoshida, H. Otaka, T. Doi, *Eur. J. Org. Chem.* **2014**, *27*, 6010.

[9] M. G. Lauer, W. H. Henderson, A. Awad, J. P. Stambuli, *Org. Lett.* **2012**, *14*, 6000.

[10] C. González-Rodríguez, S. R. Parsons, A. L. Thompson, M. C. Willis, *Chem. Eur. J.* **2010**, *16*, 10950.

[11] M. C. Willis, H. E. Randell-Sly, R. L. Woodward, S. J. McNally, G. S. Currie, *J. Org. Chem.* **2006**, *71*, 5291.

[12] a) Y. Takaya, M. Ogasawara, T. Hayashi, *J. Am. Chem. Soc.* **1998,** *120,* 5579; b) K. Okamoto, T. Hayashi, V. H. Rawal, *Org. Lett.* **2008**, *10*, 4387; c) M. Pucheault, S. Darses, J.-P. Genêt, *Eur. J. Org. Chem.* **2002**, *21*, 3552; d) T. Hayashi, M. Takahashi, Y. Takaya, M. Ogasawara, *J. Am. Chem. Soc.* **2002**, *124*, 5052.

7. NMR spectra of novel compounds

(*S*,*E*)-5,9-Dimethyldeca-2,8-dienal (SM2d)

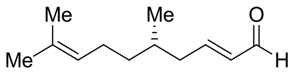

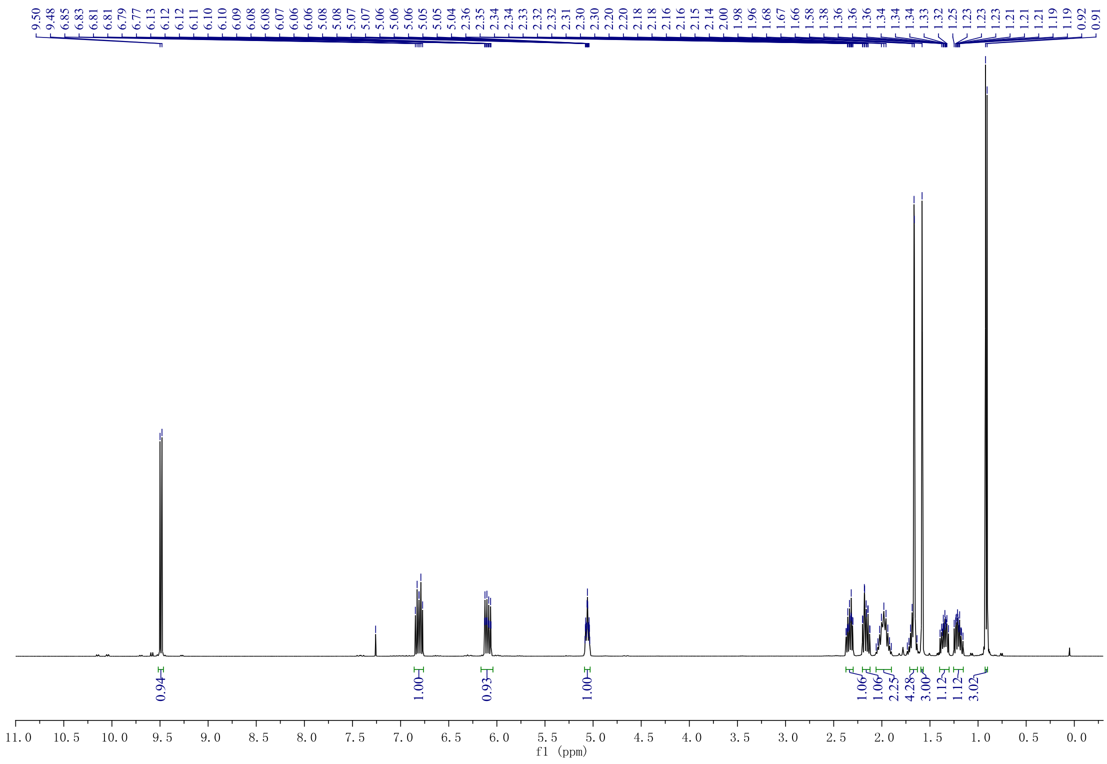

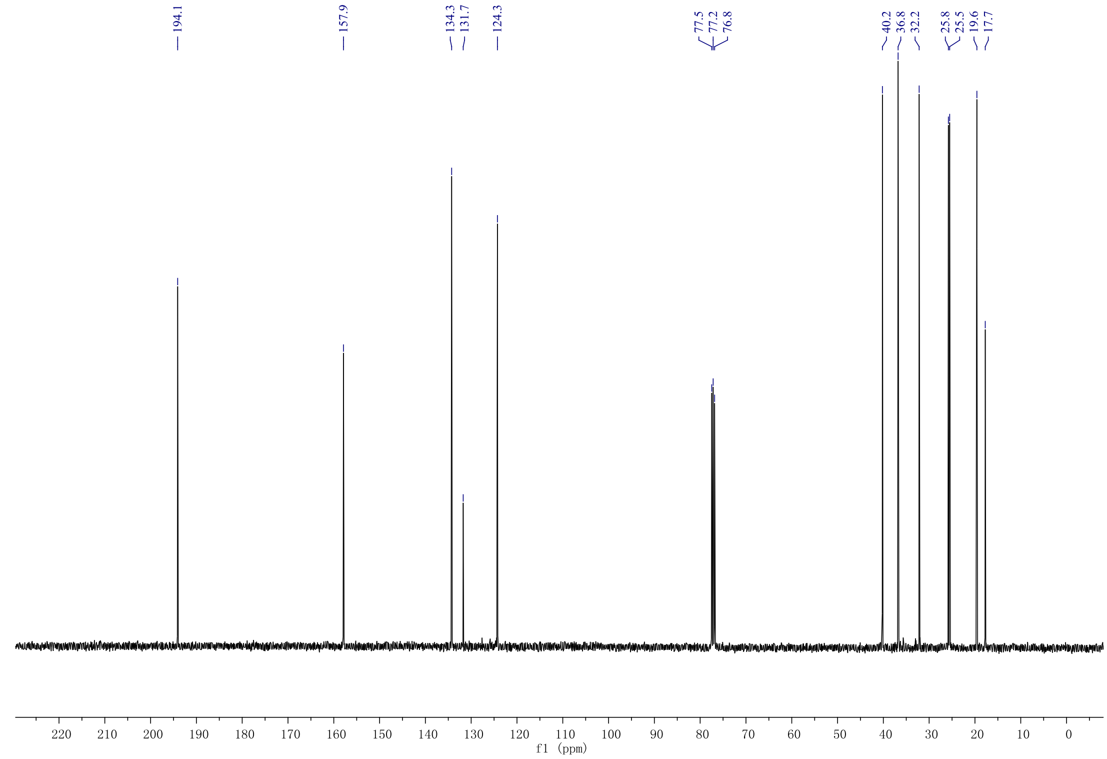

(*E*)-7-Oxohept-5-en-1-yl acetate (SM2f)

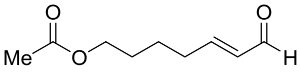

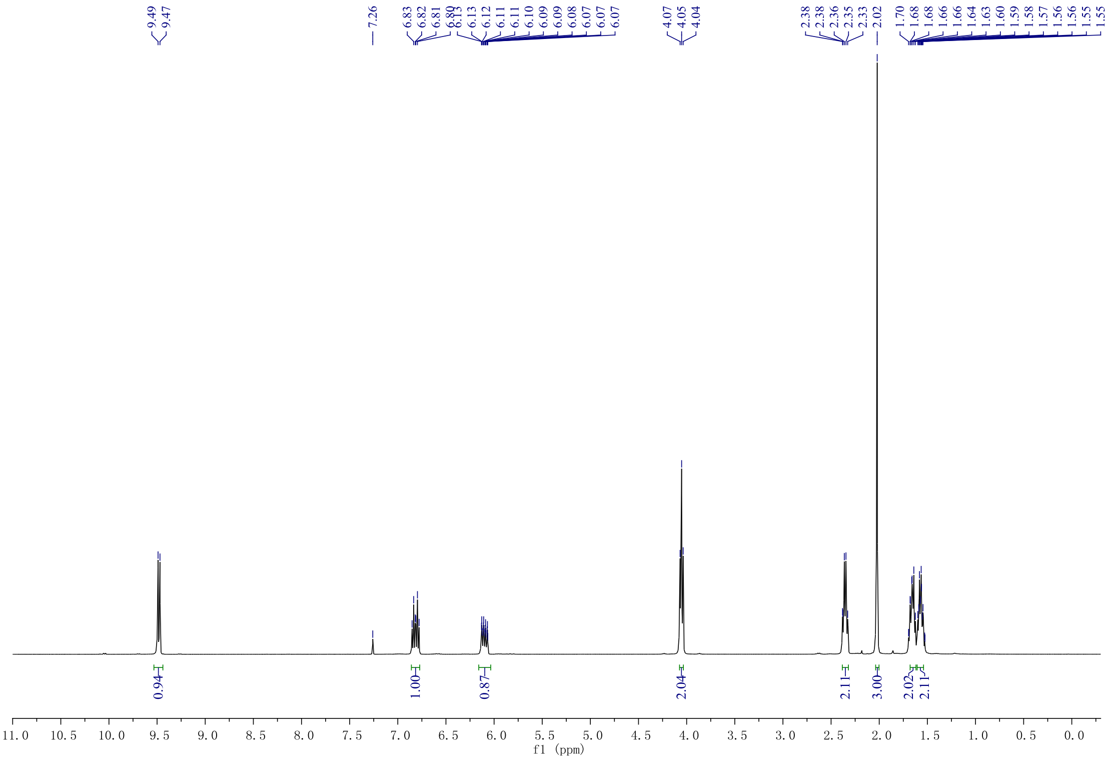

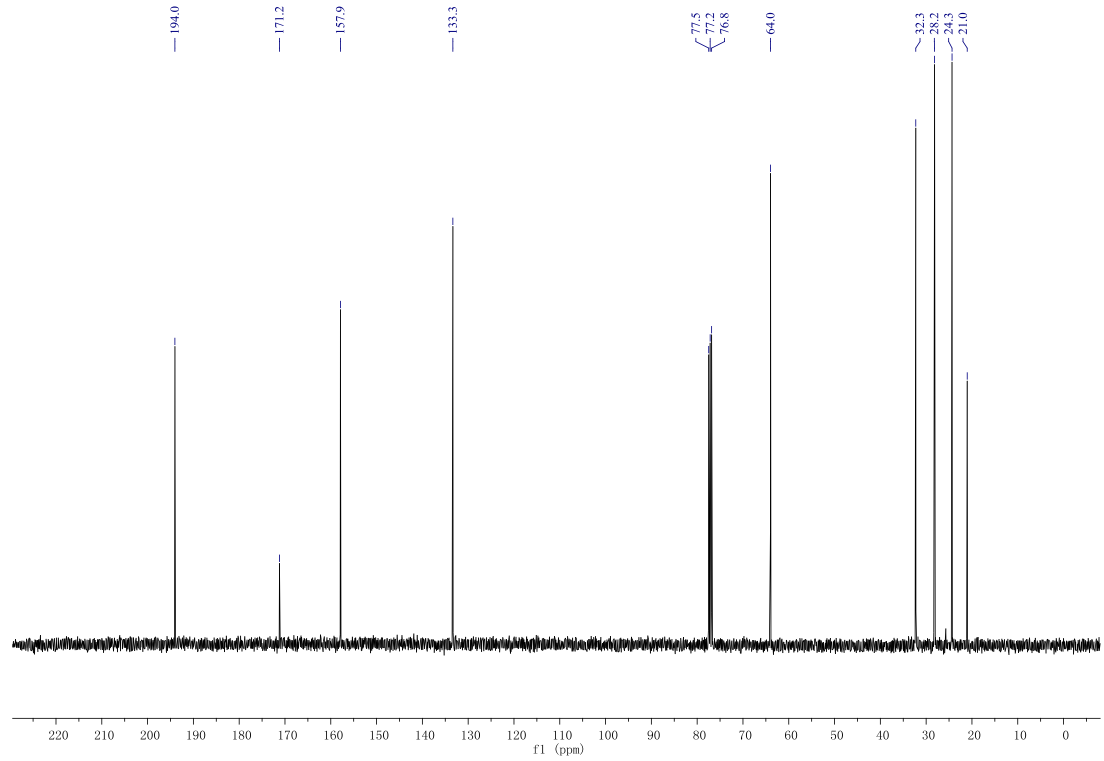

3-(Ethylthio)-4-methylpentanal (1b)

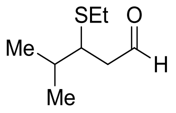

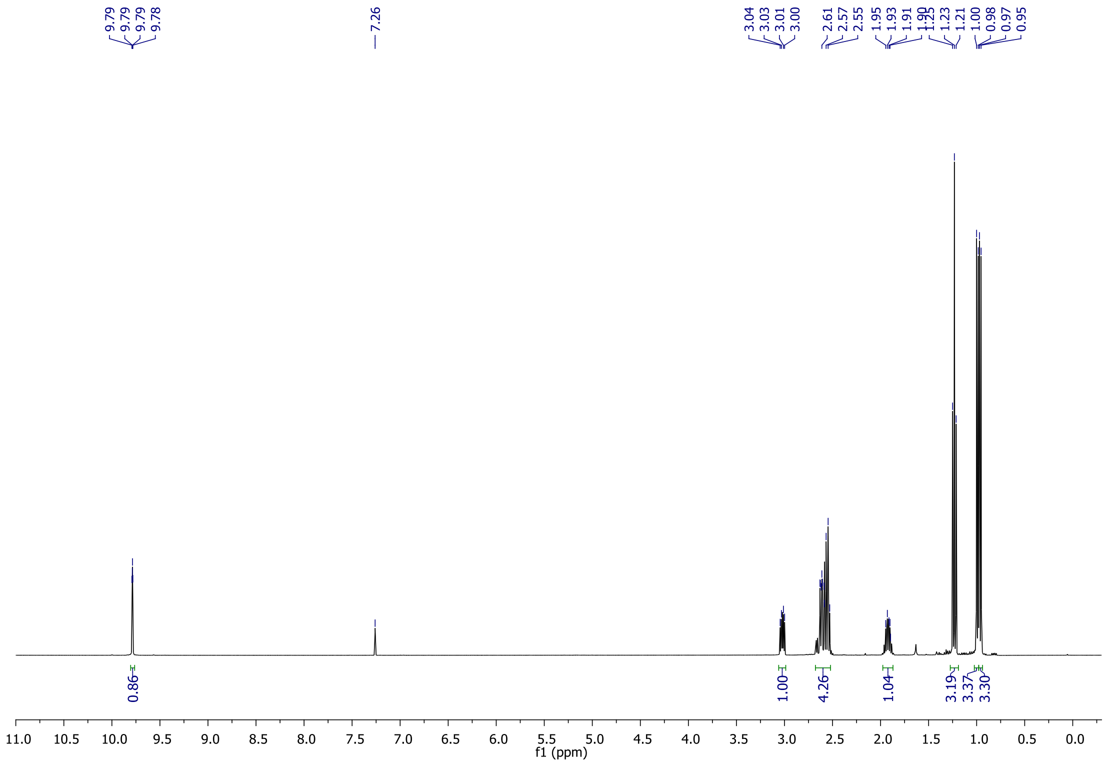

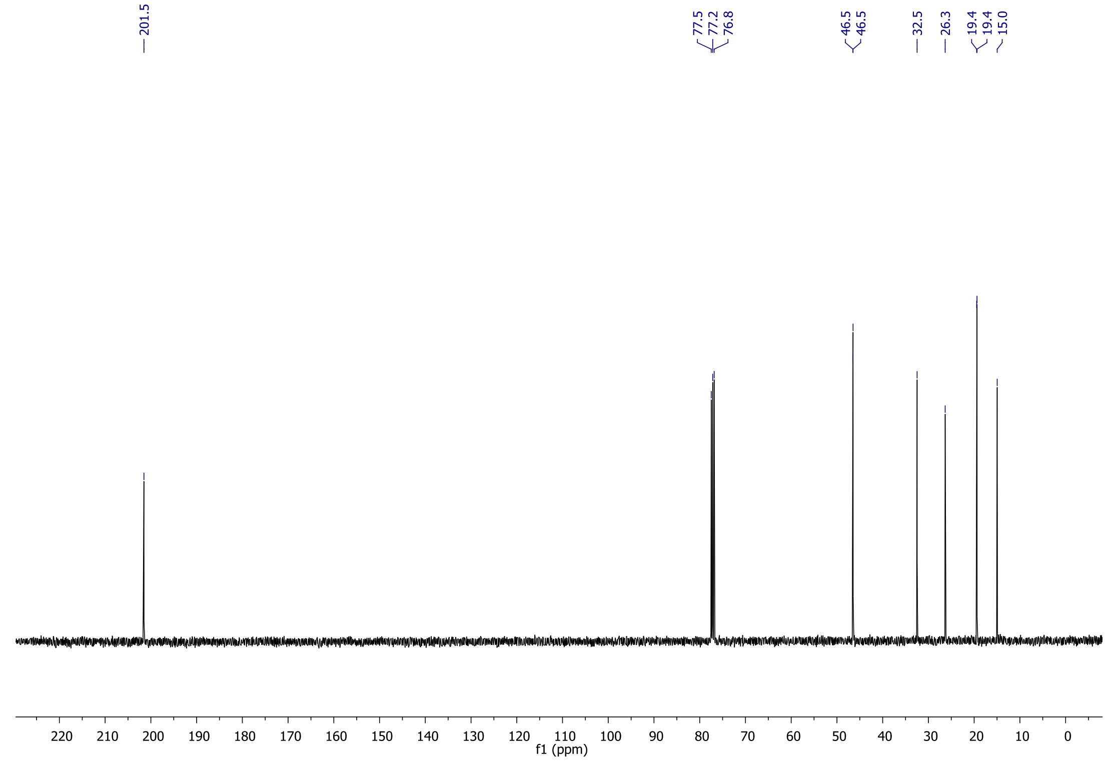

3-(Ethylthio)-5,5-dimethylhexanal (1c)

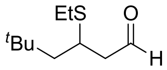

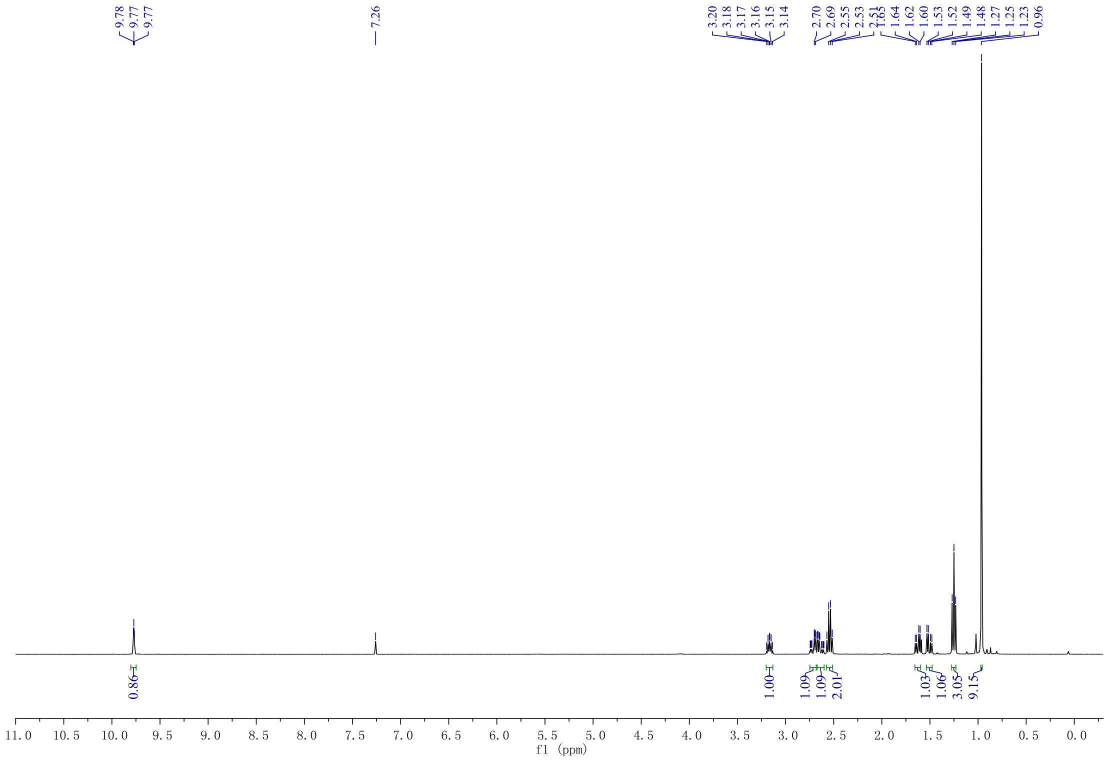

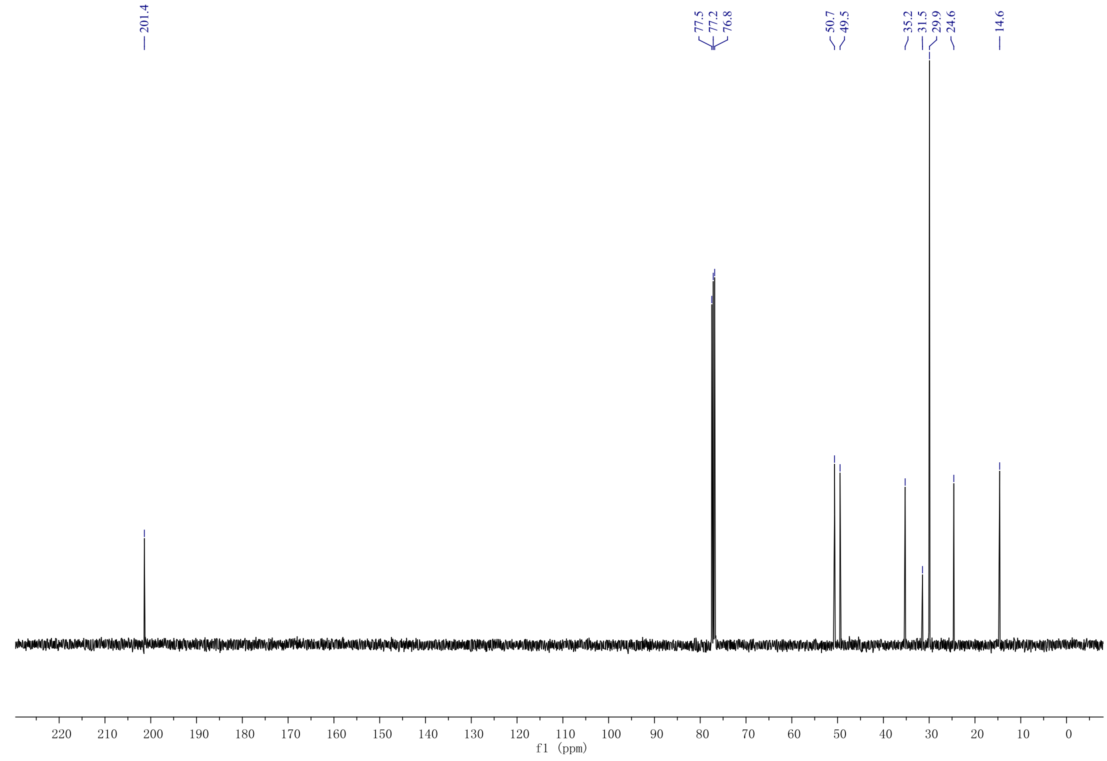

3-Cyclohexyl-3-(ethylthio)propanal (1d)

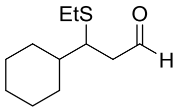

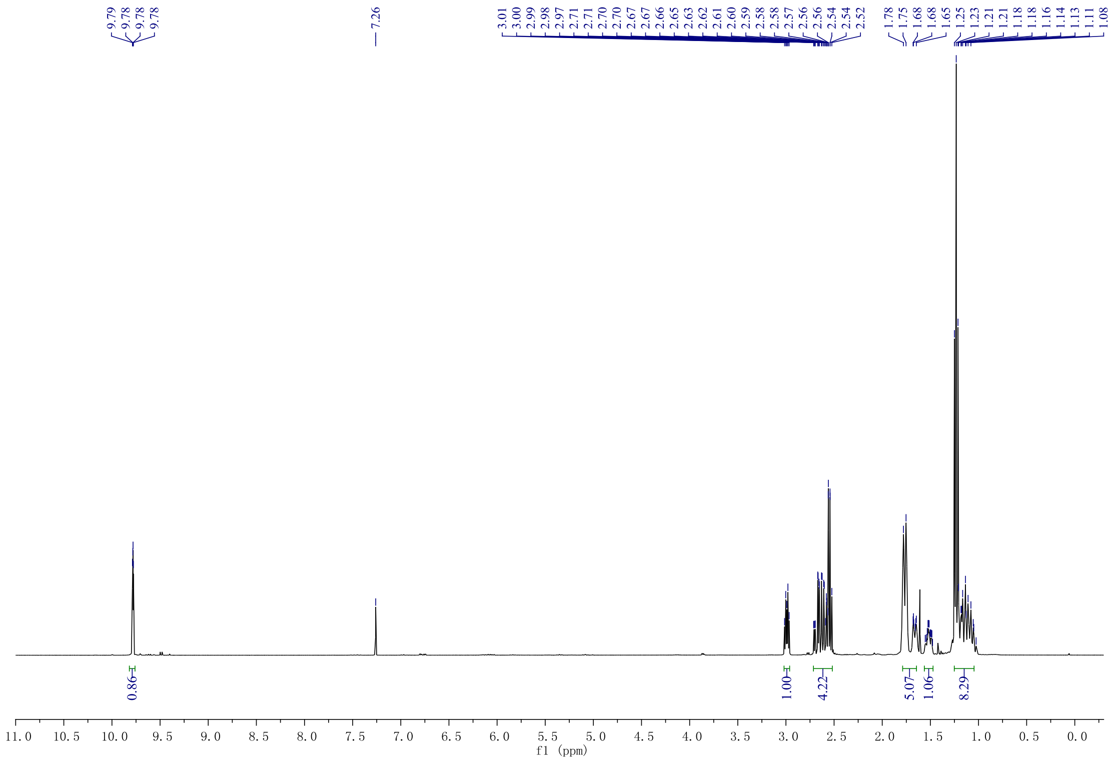

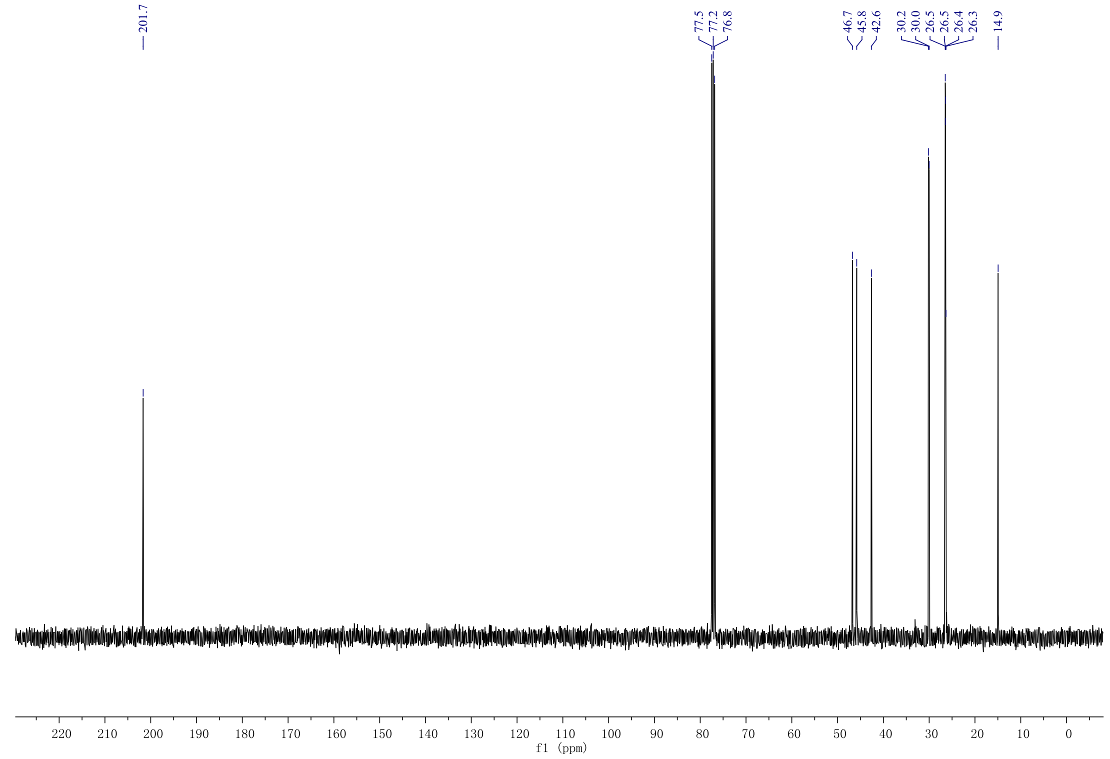

3-Cyclopropyl-3-(ethylthio)propanal (1e)

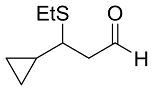

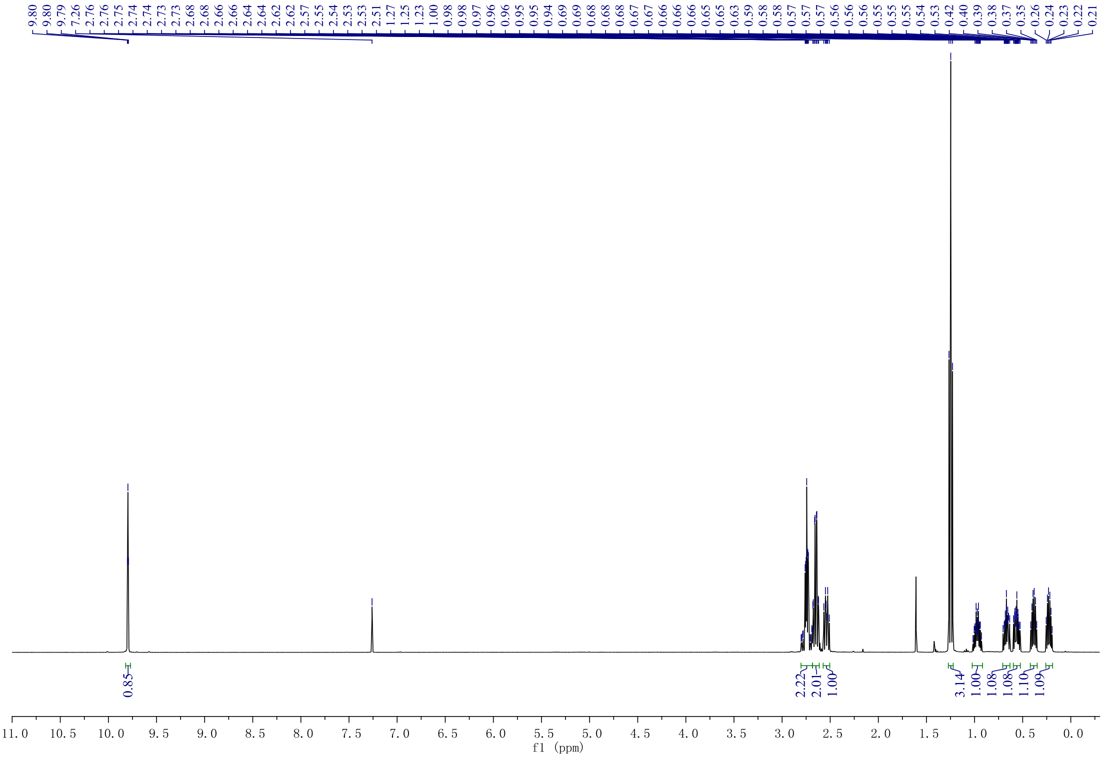

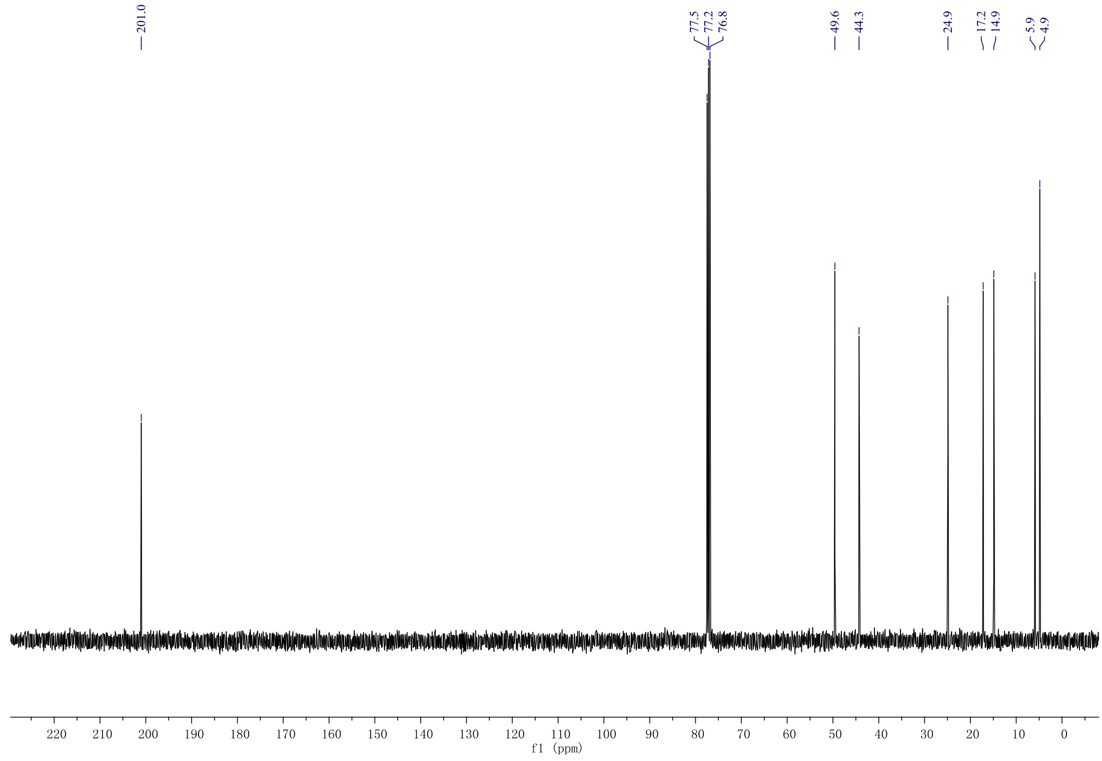

3-(Ethylthio)-5-phenylpentanal (1f)

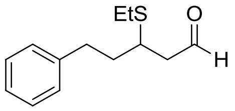

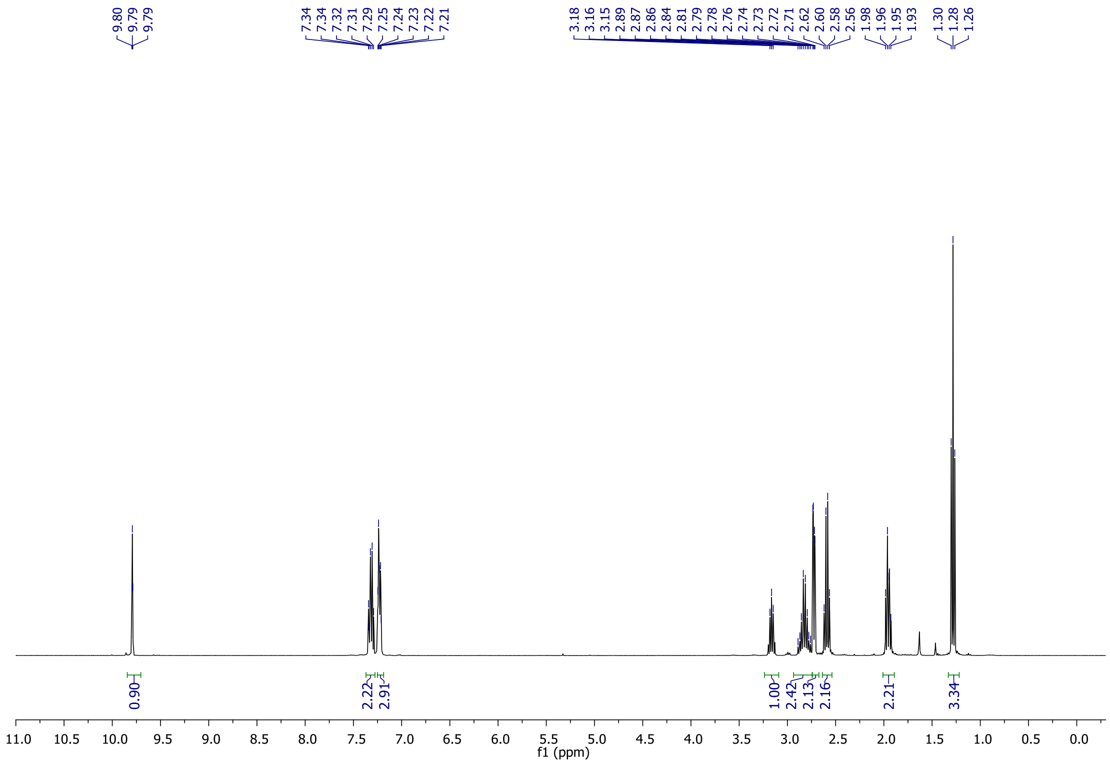

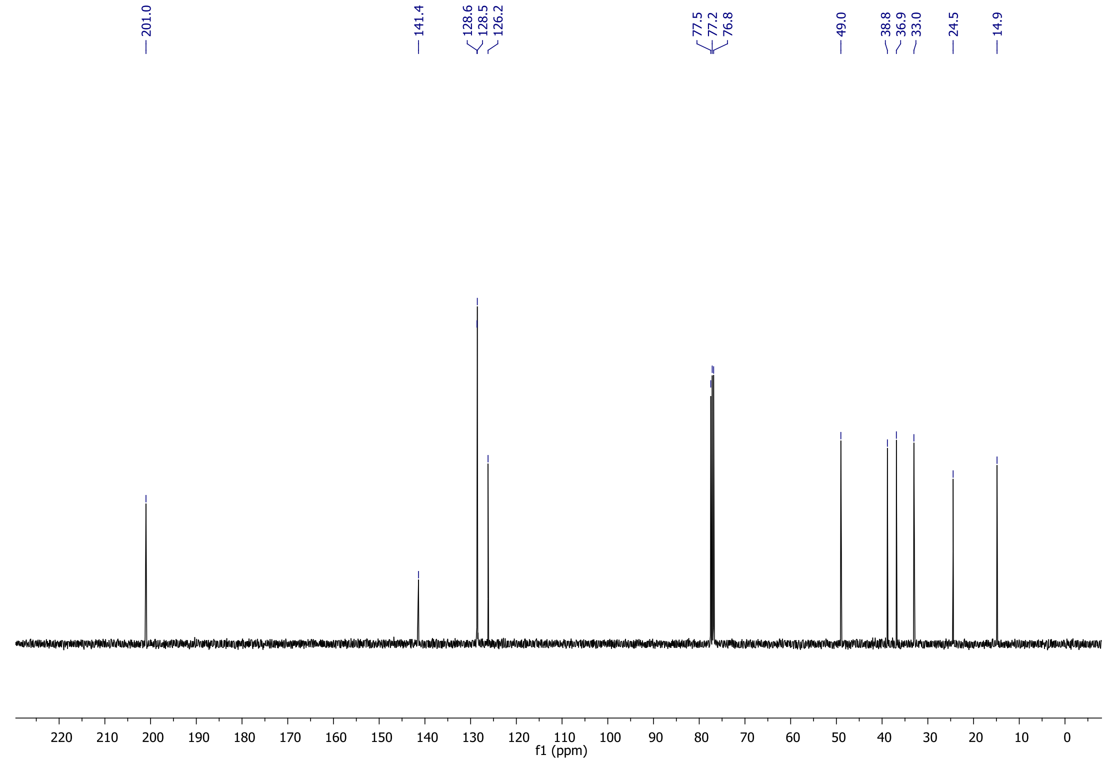

3-(4-Bromophenyl)-3-(ethylthio)propanal (1h)

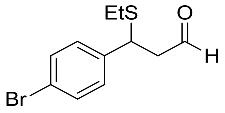

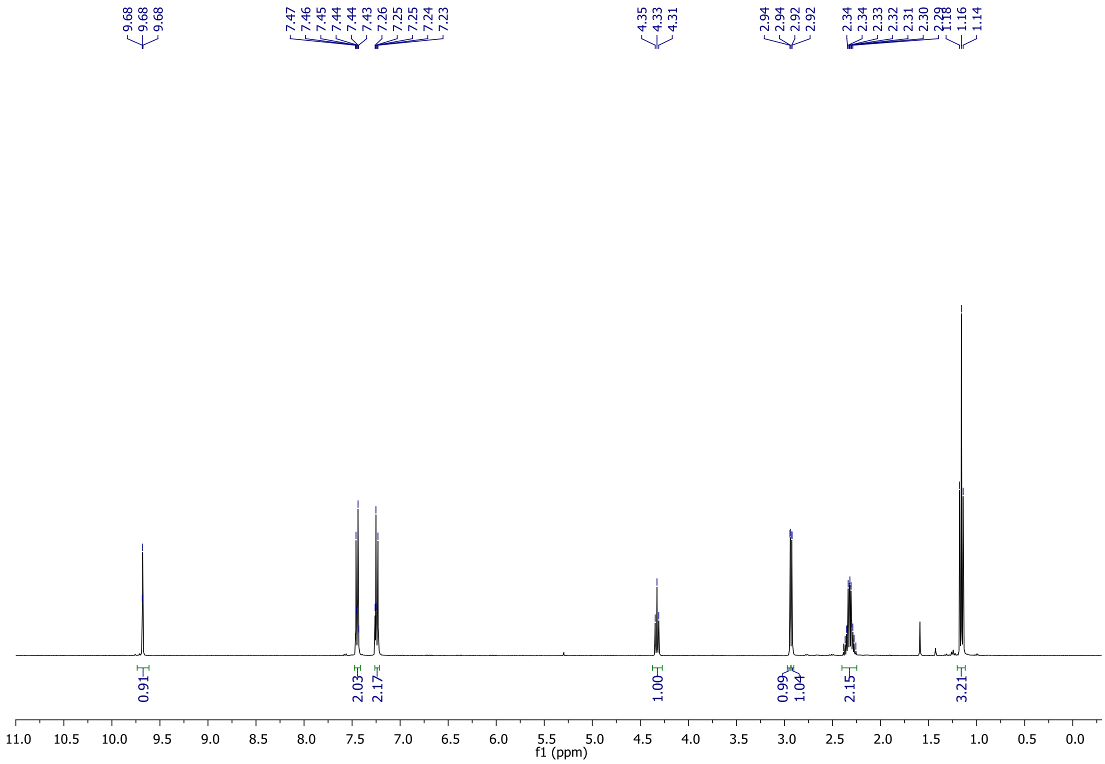

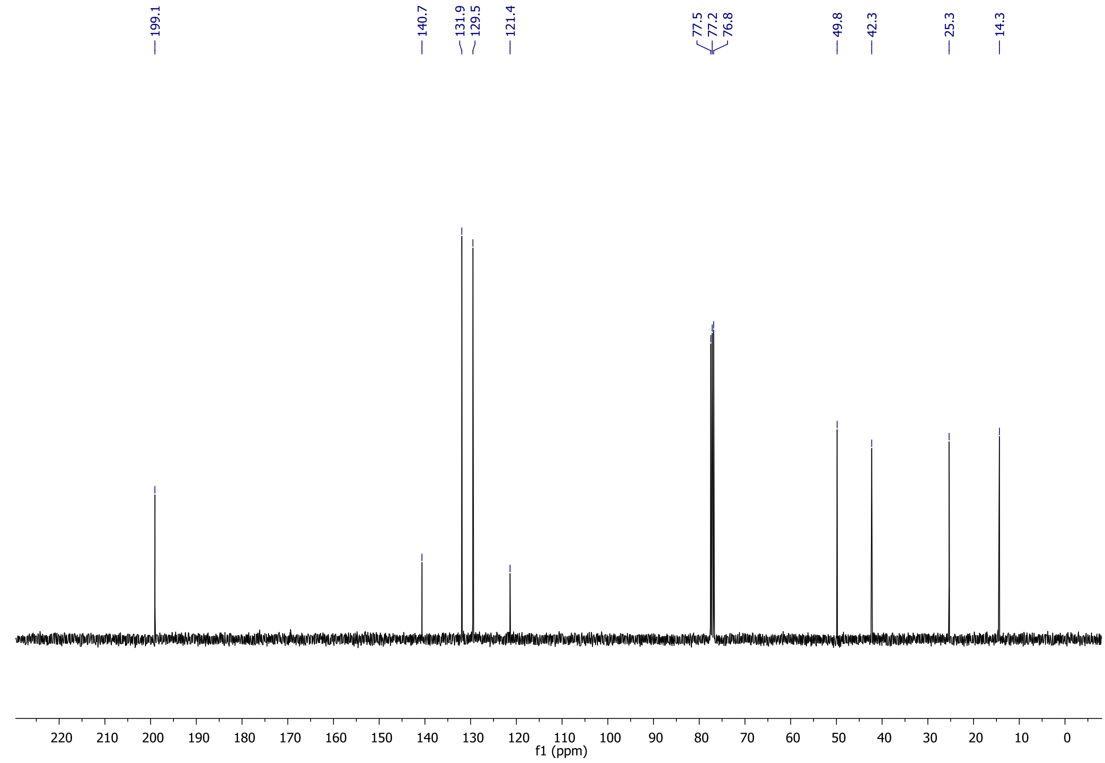

3-(Ethylthio)-3-(4-nitrophenyl)propanal (1i)

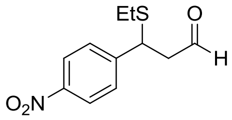

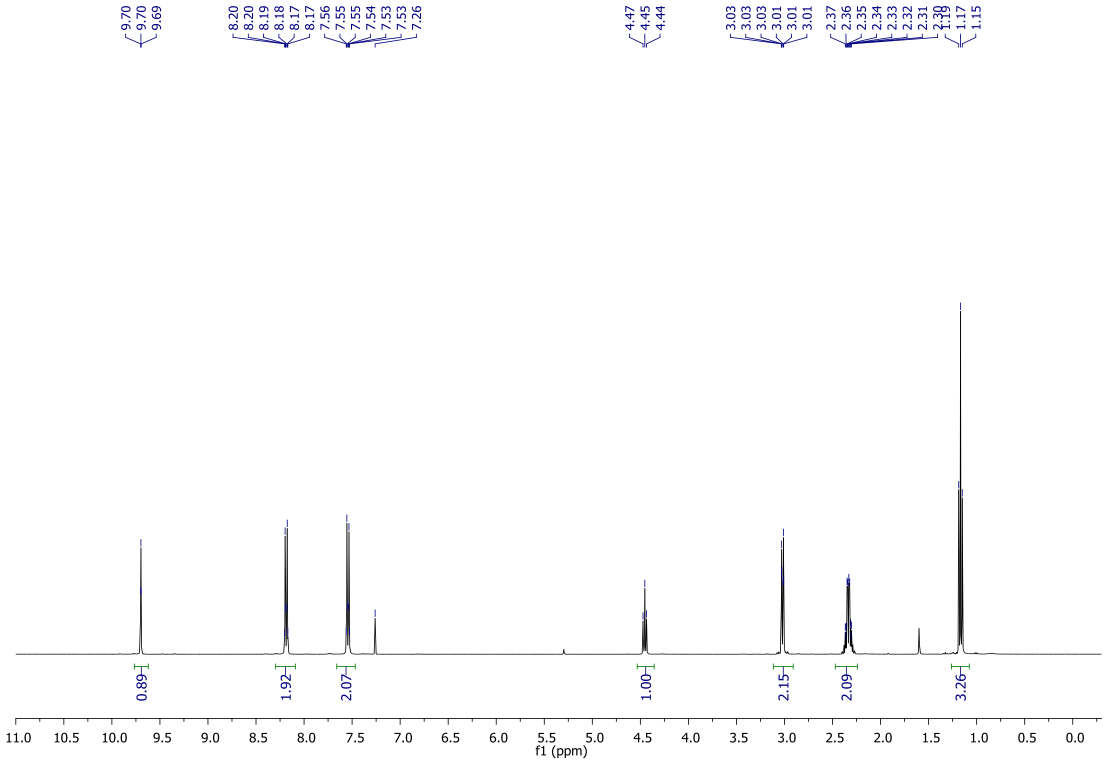

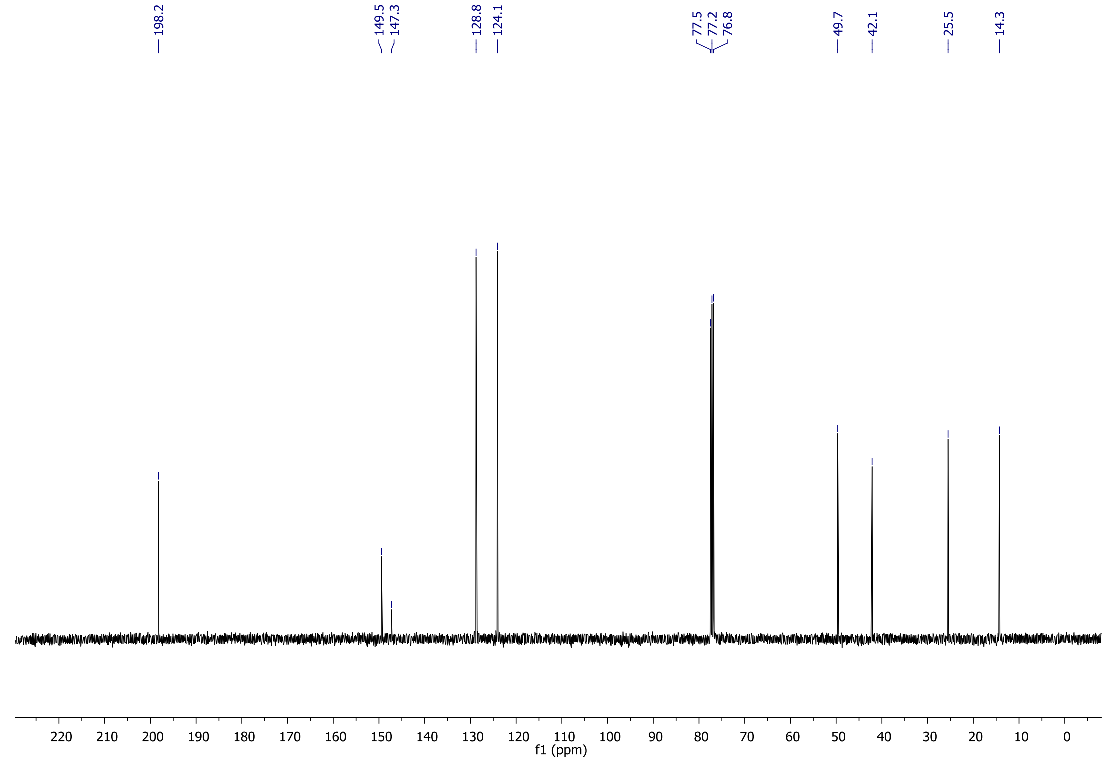

5-(Benzyloxy)-3-(ethylthio)pentanal (1j)

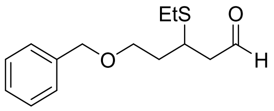

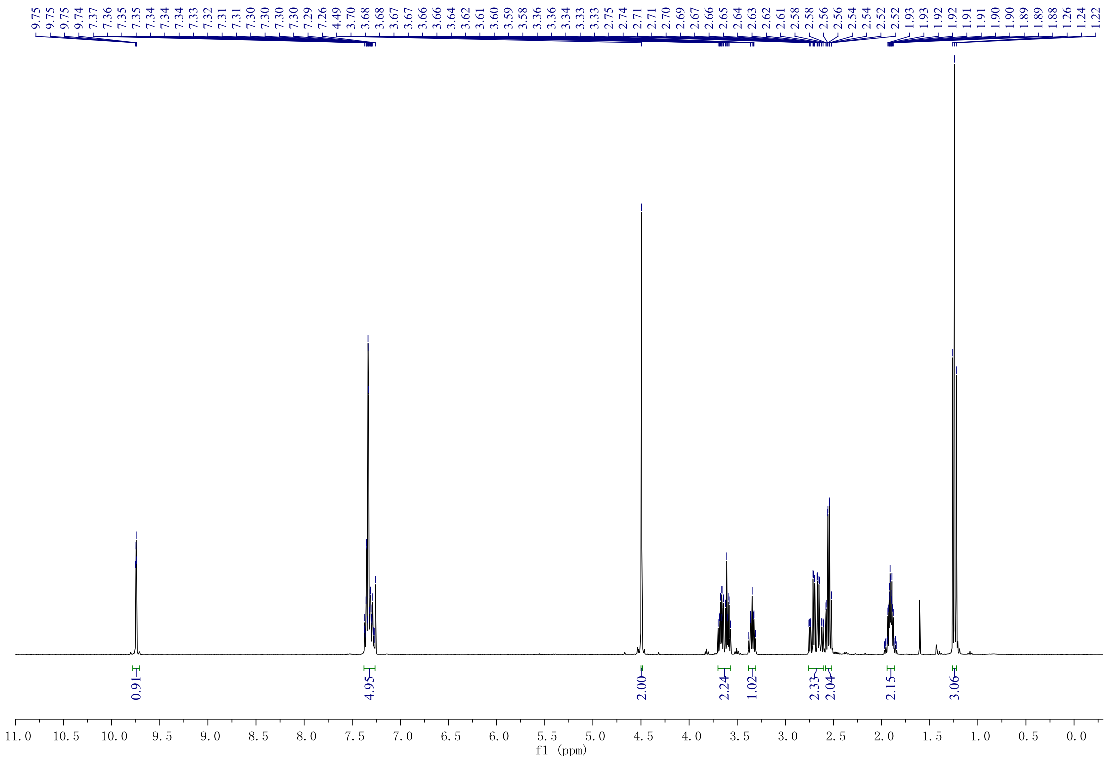

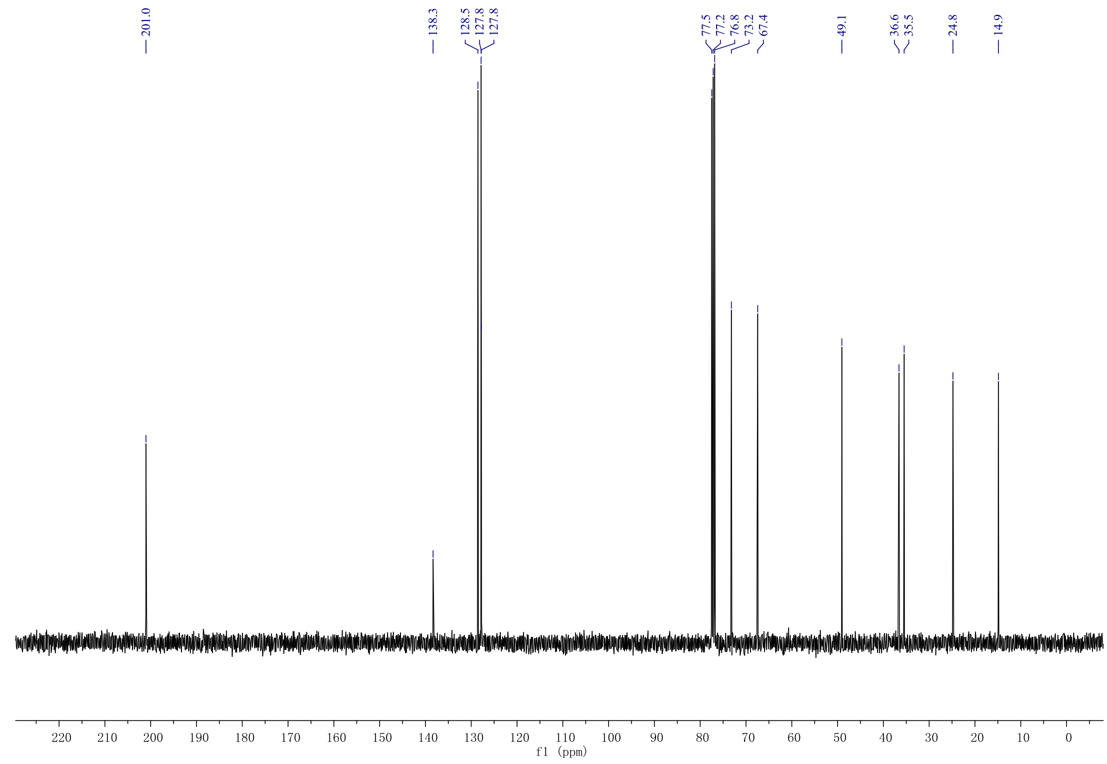

5-(Ethylthio)-7-oxoheptyl acetate (1k)

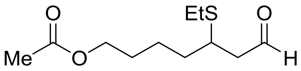

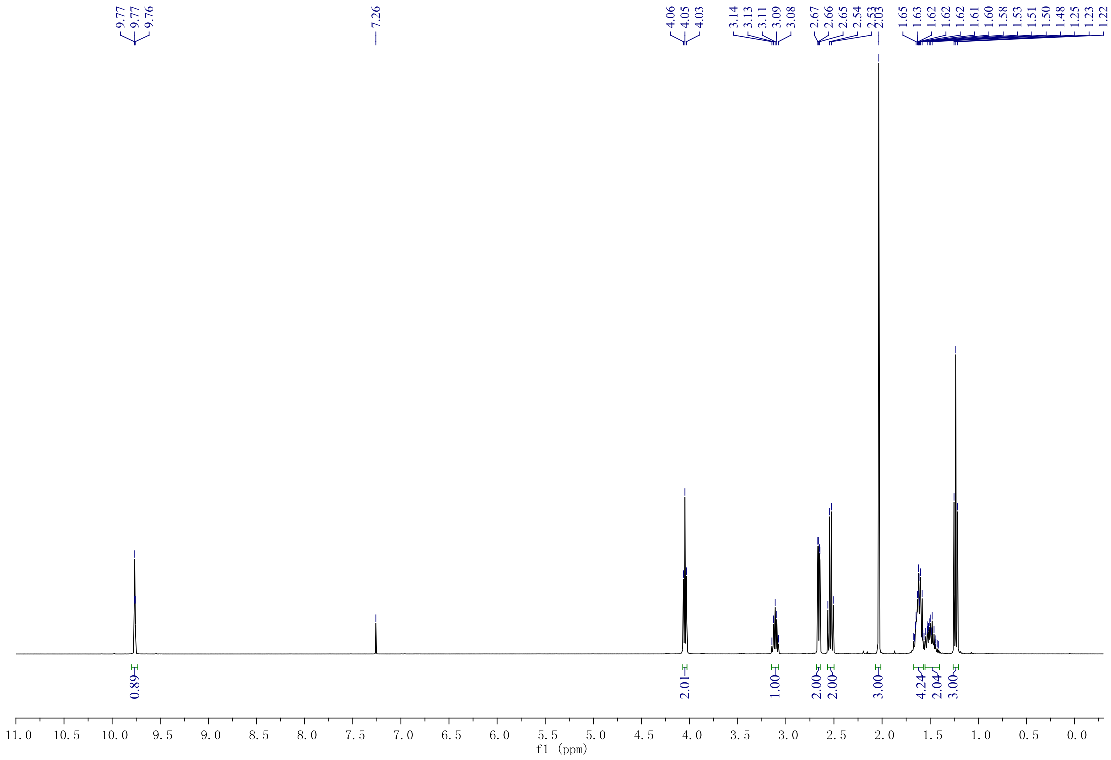

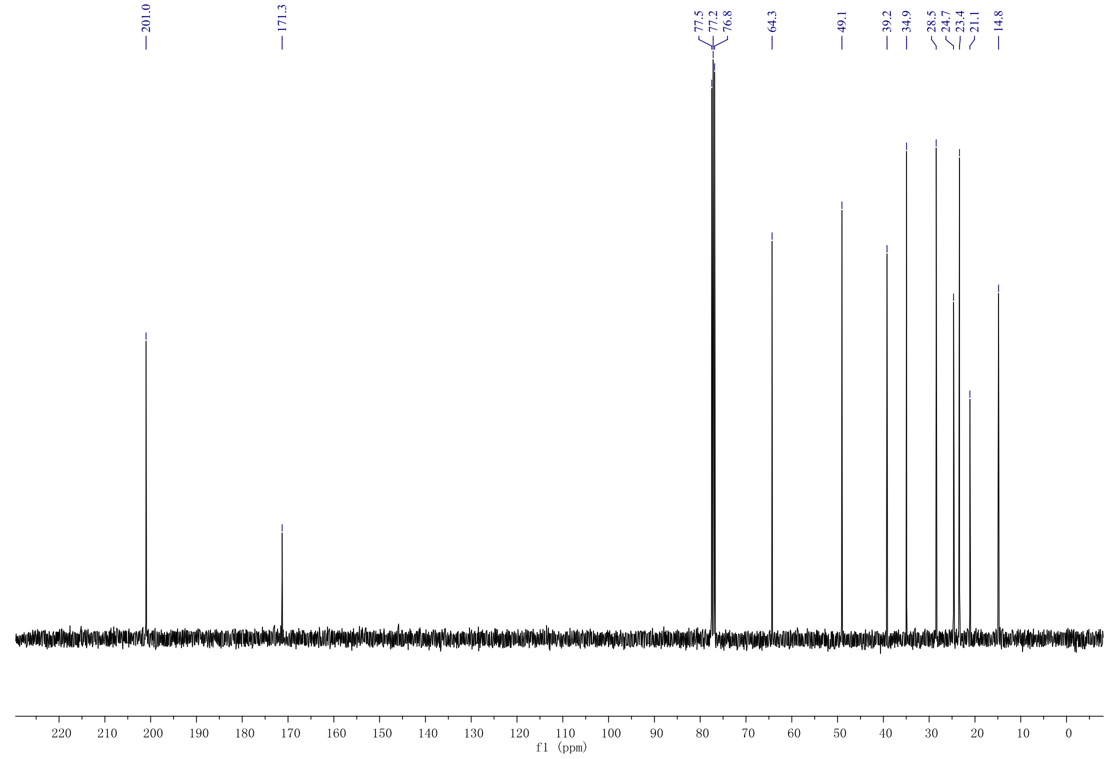

6-(1,3-Dioxoisoindolin-2-yl)-3-(ethylthio)hexanal (1l)

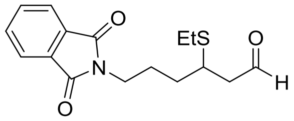

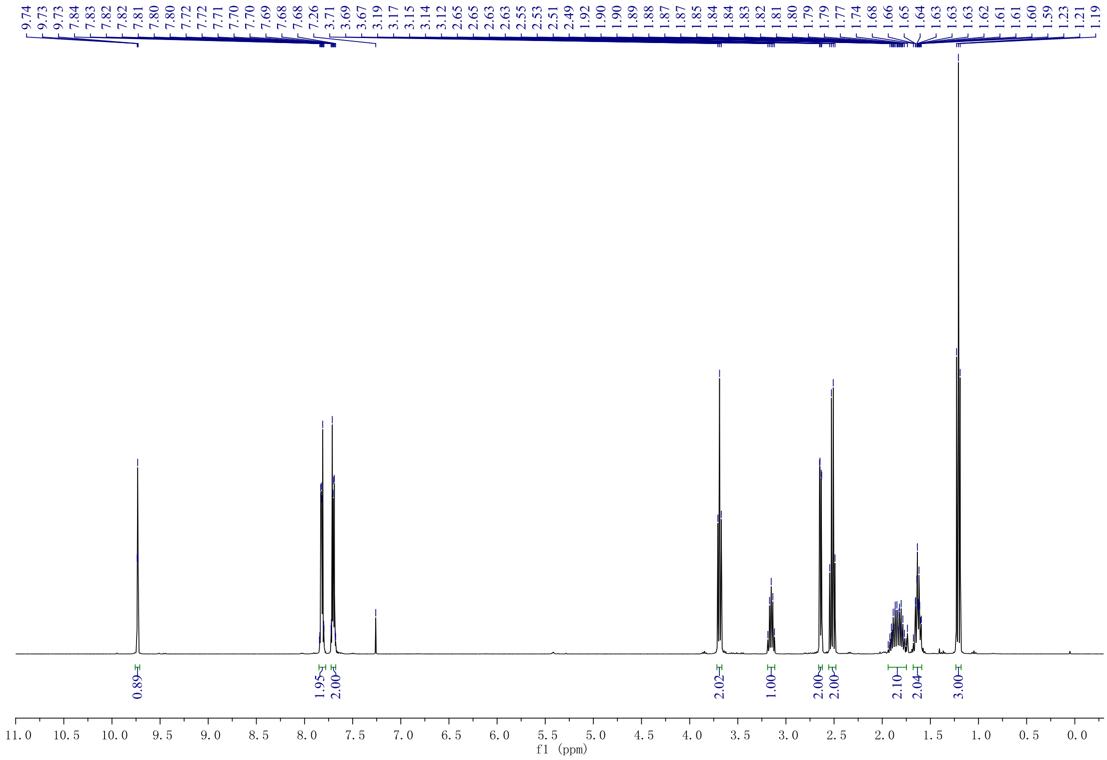

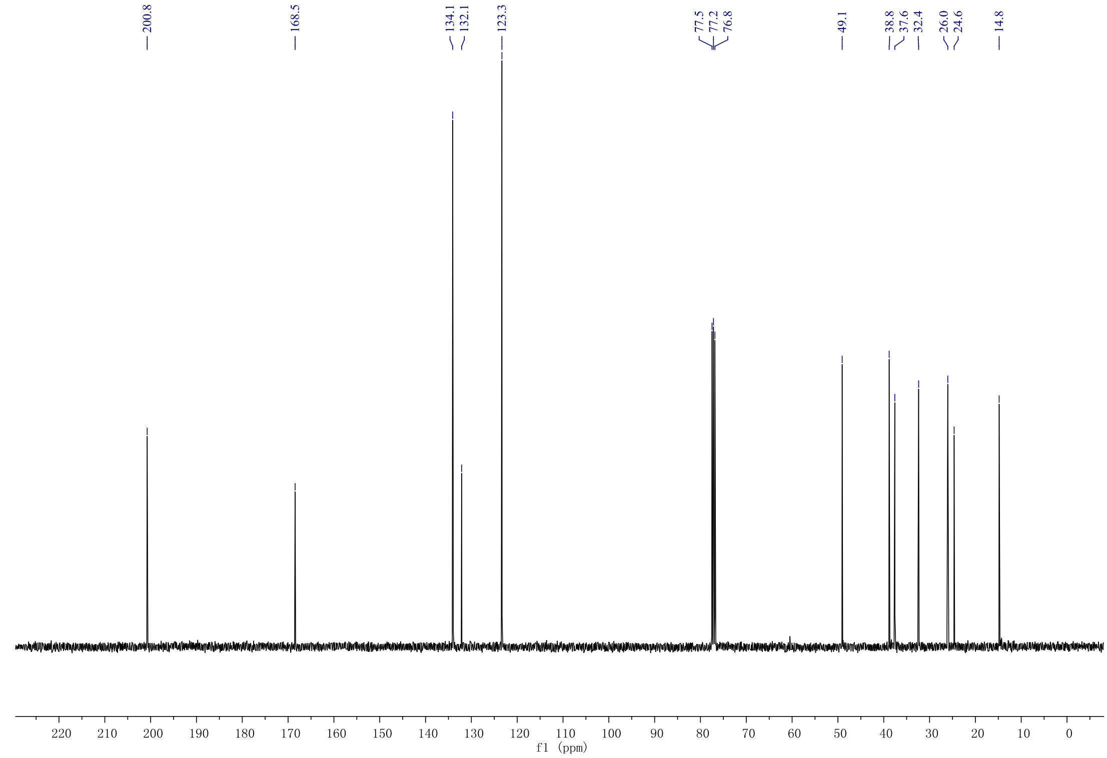

(5*S*)-3-(Ethylthio)-5,9-dimethyldec-8-enal (1m)

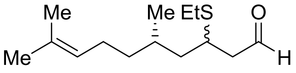

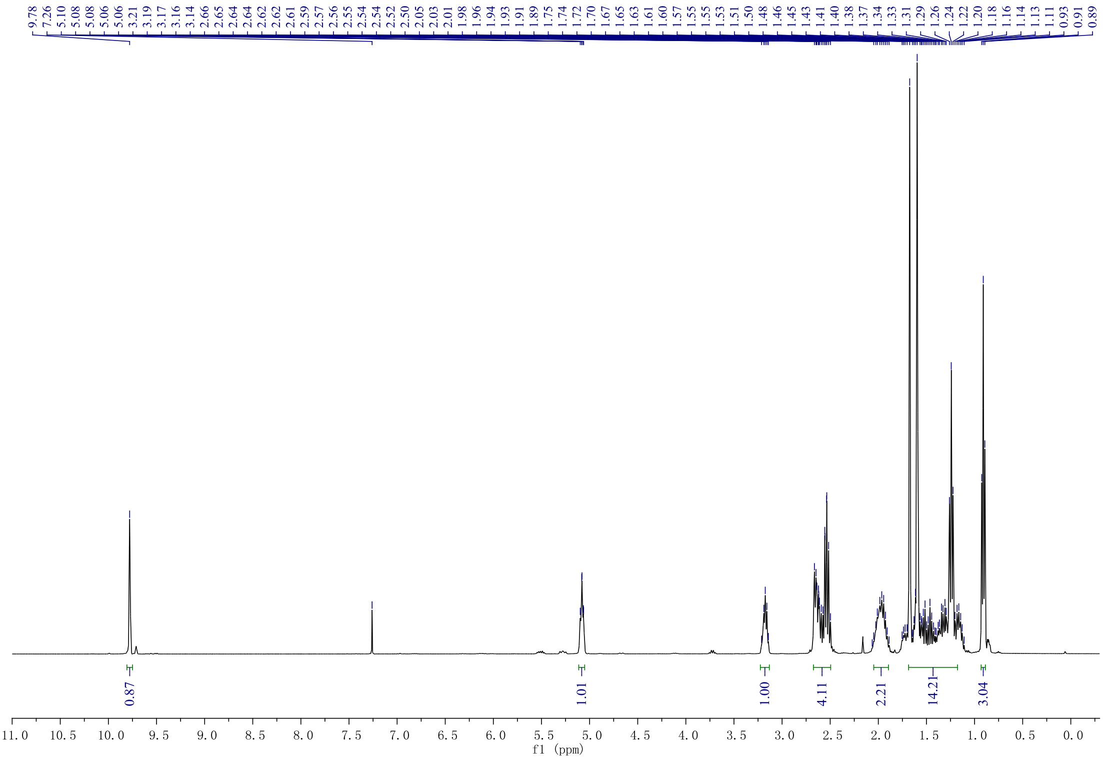

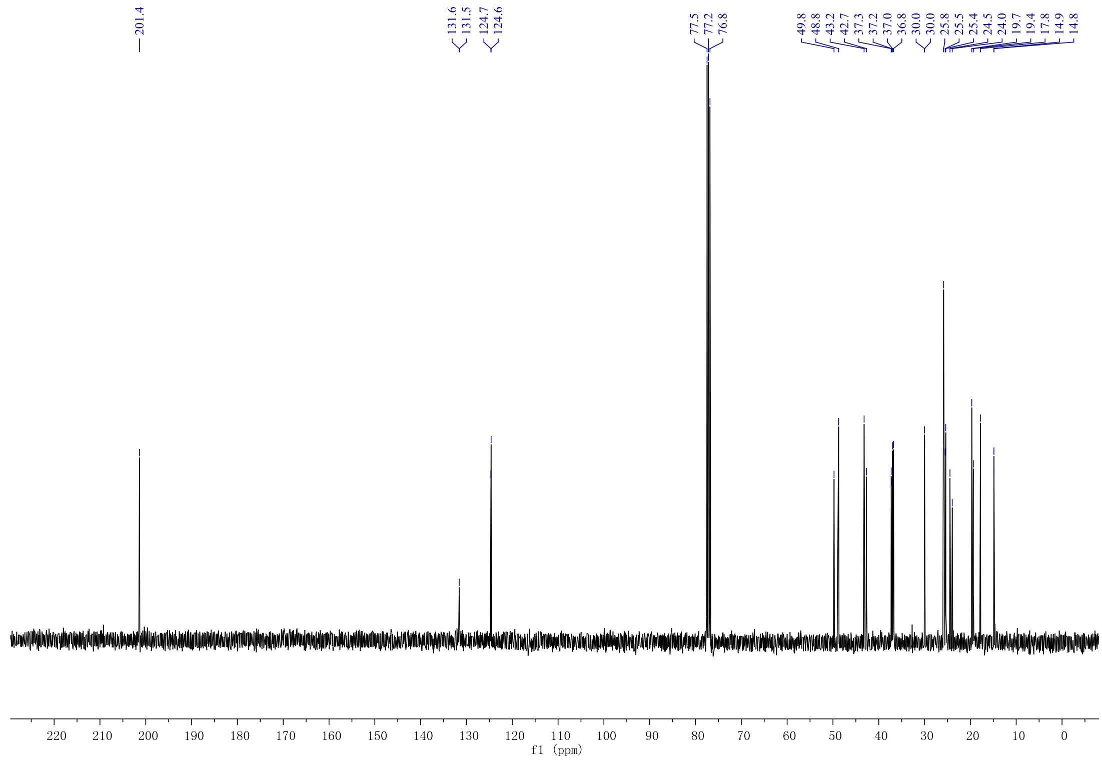

(*S*)-6-(4-methoxyphenyl)hexadecan-8-one (4a)

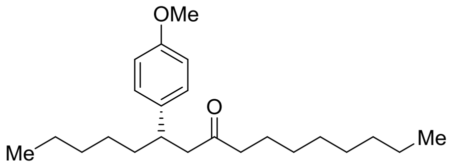

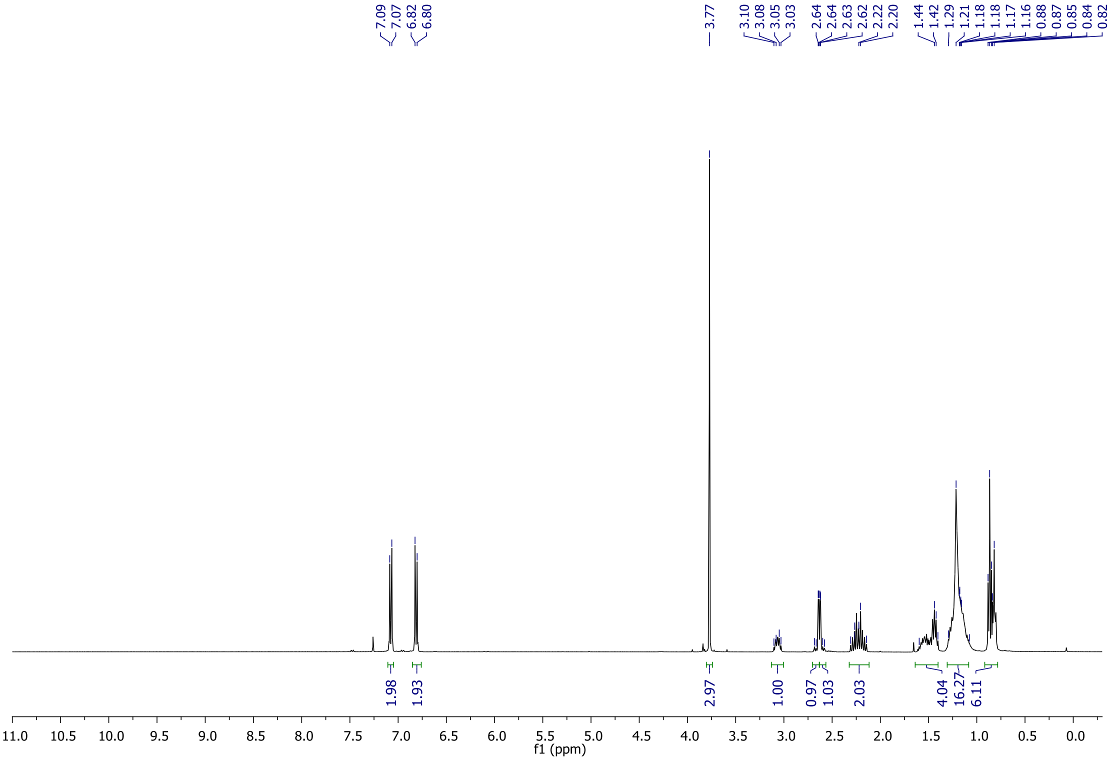

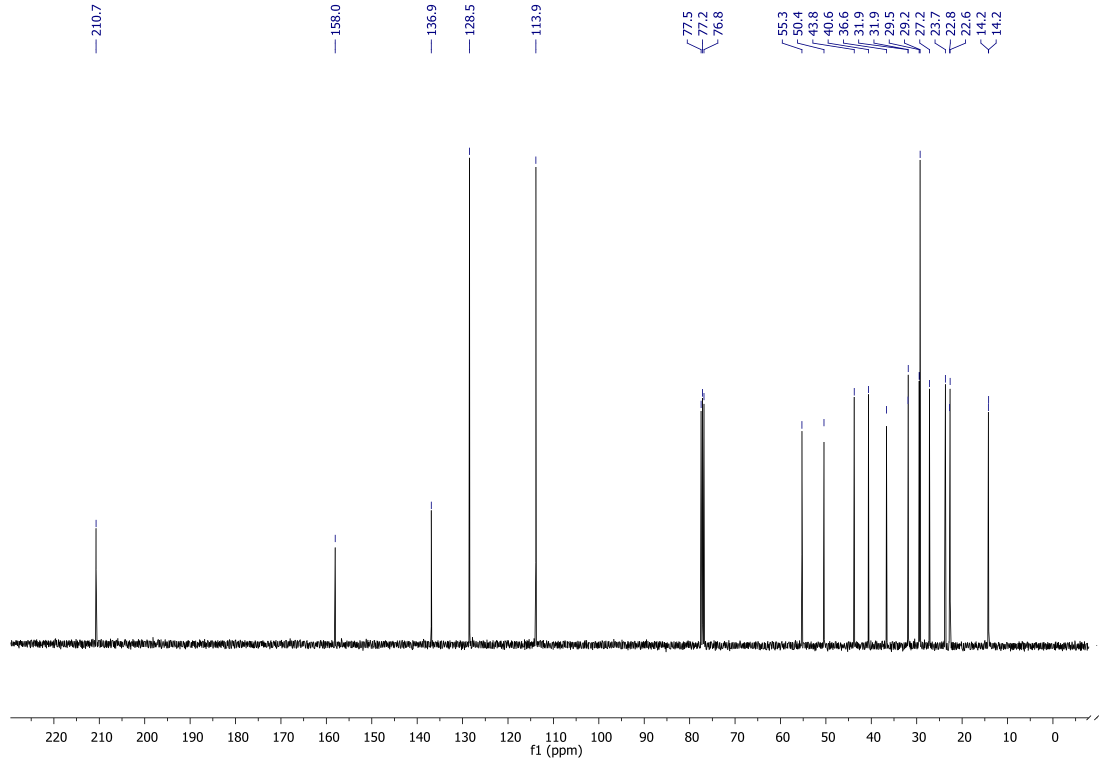

(*S*)-1-hydroxy-8-(4-methoxyphenyl)tridecan-6-one (4v)

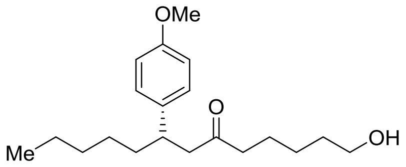

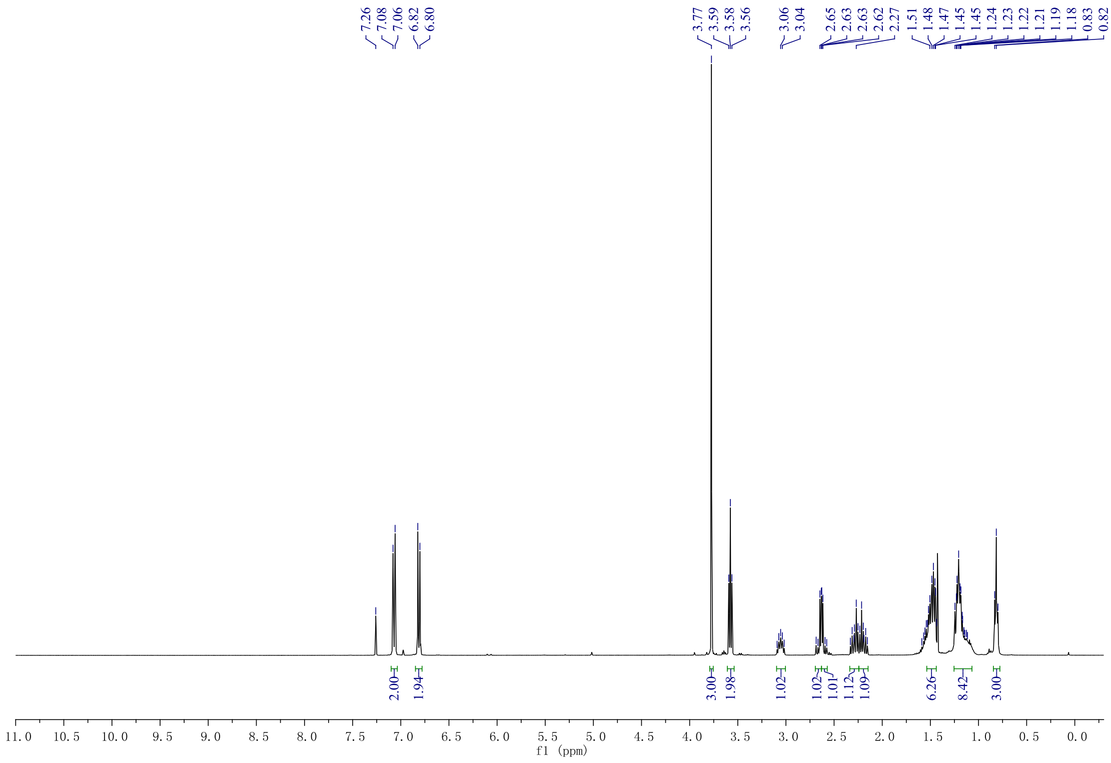

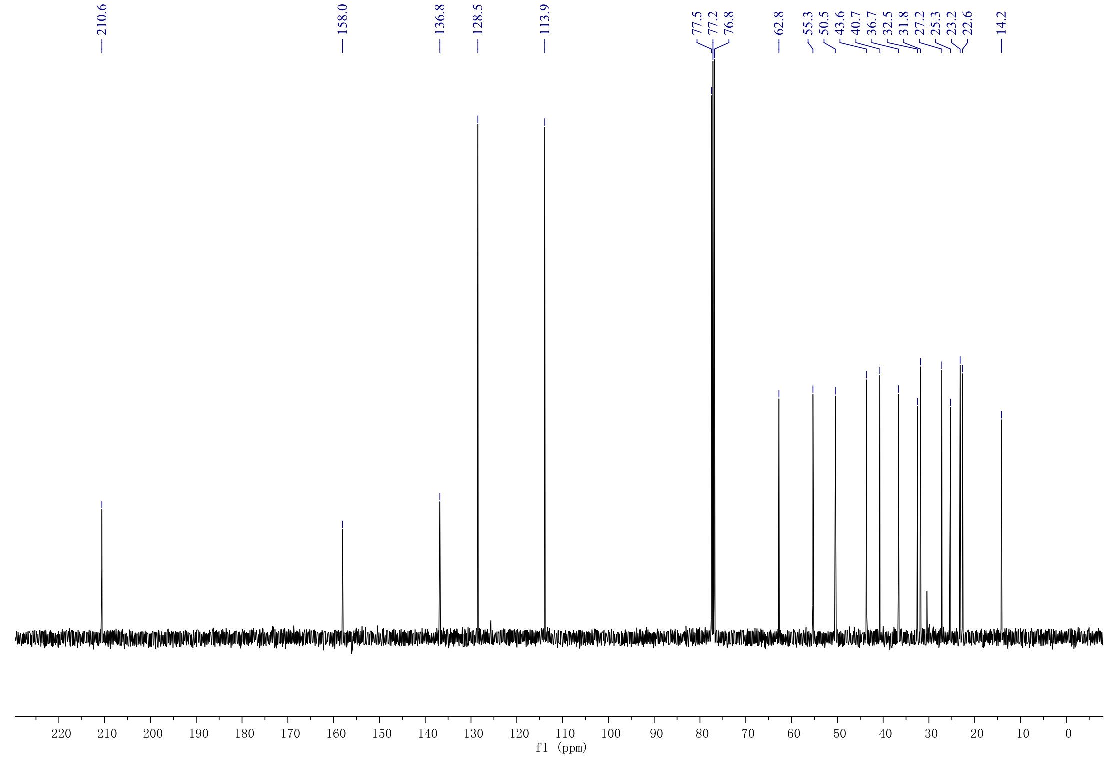

(*S*)-7-(2-Methoxyphenyl)-1-phenyldodecan-5-one (4b)

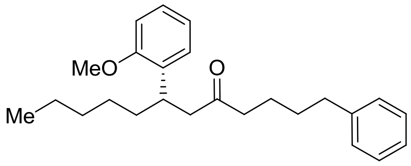

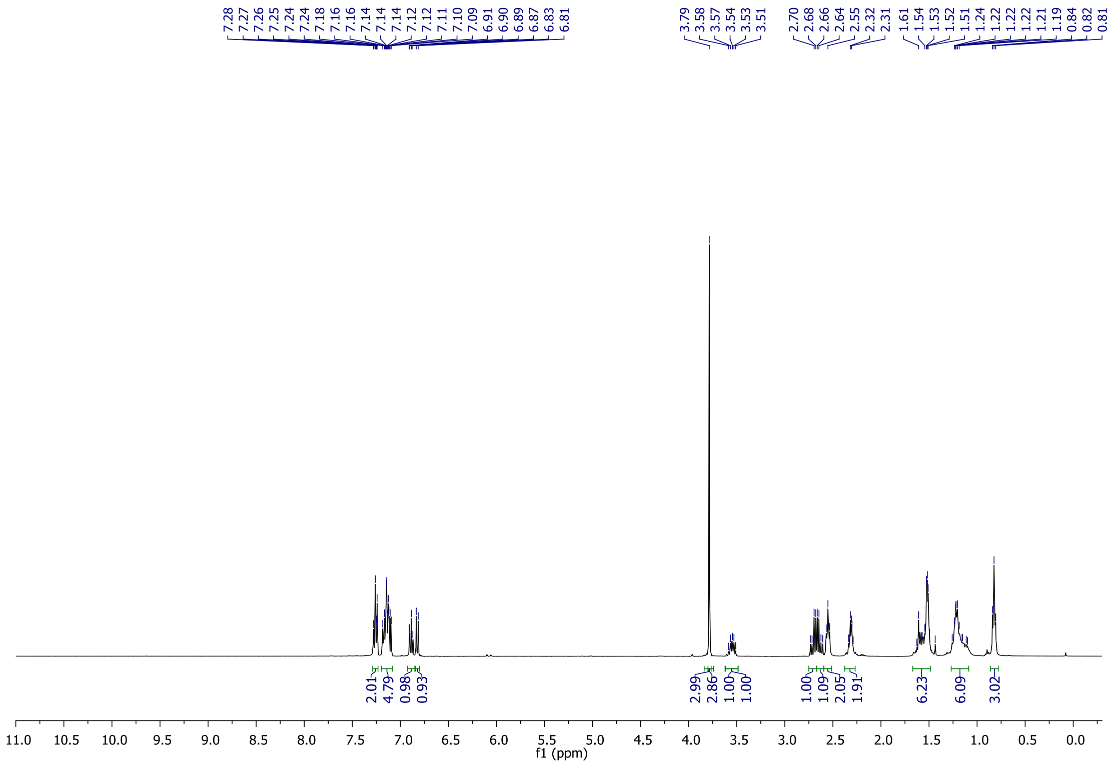

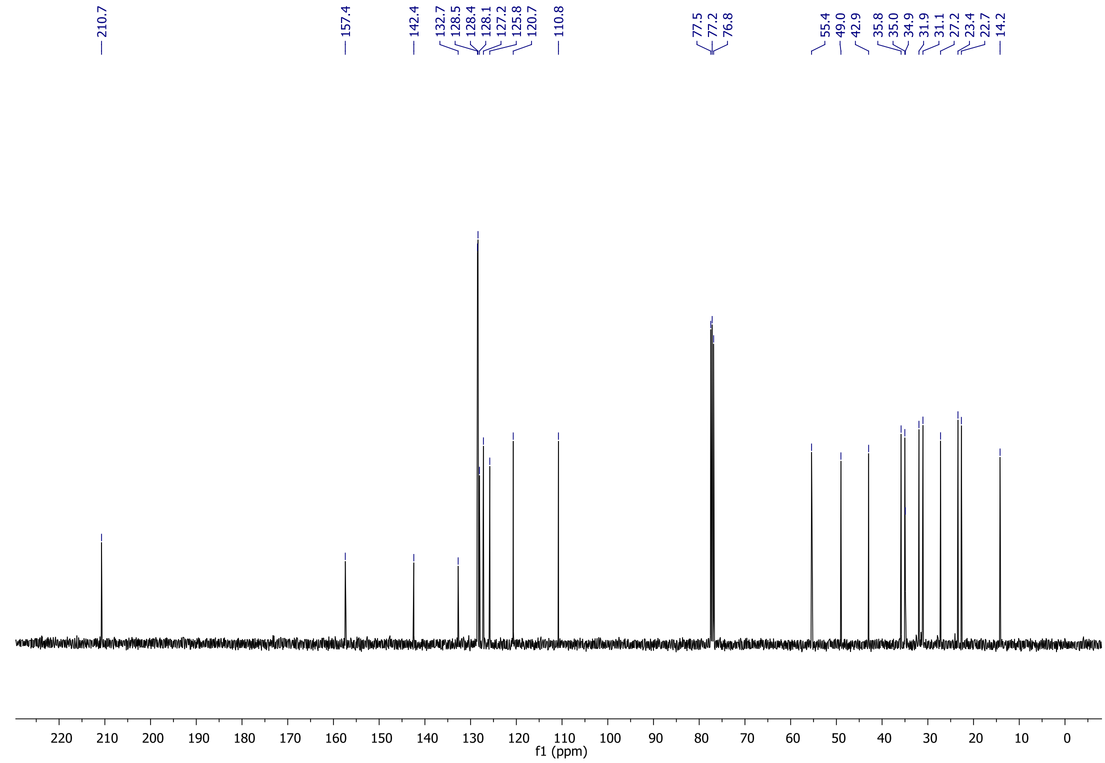

(*S*)-6-(*p*-Tolyl)hexadecan-8-one (4c)

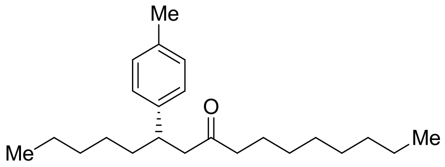

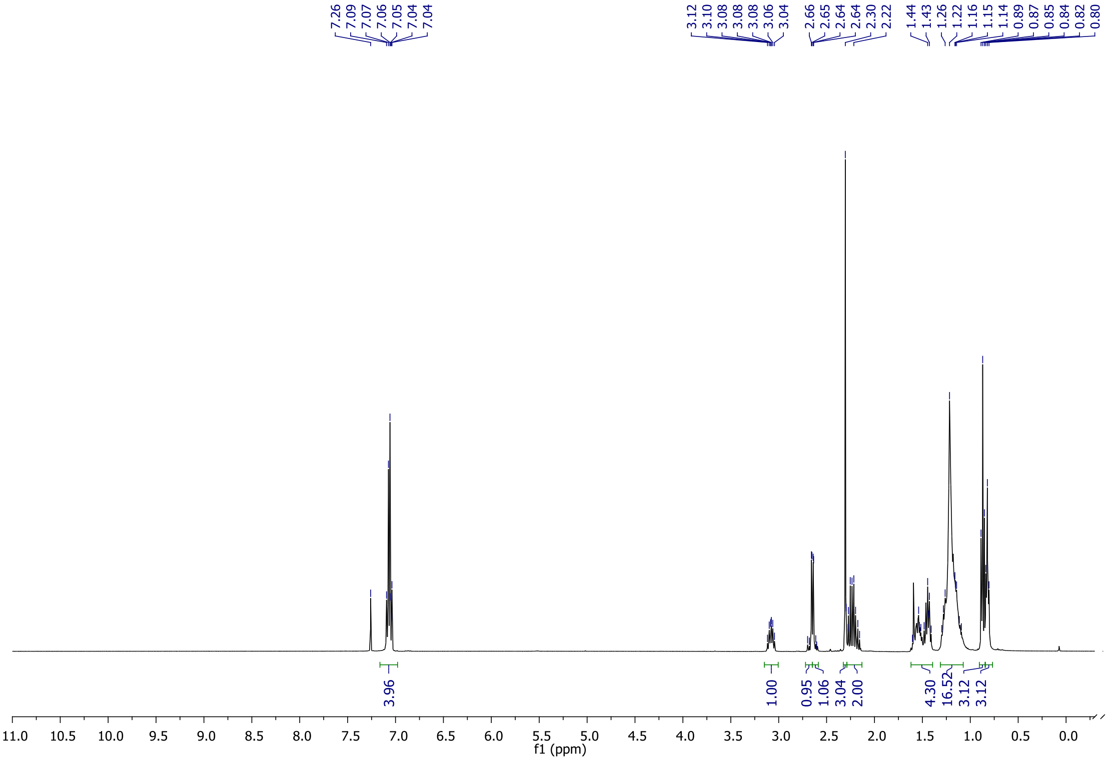

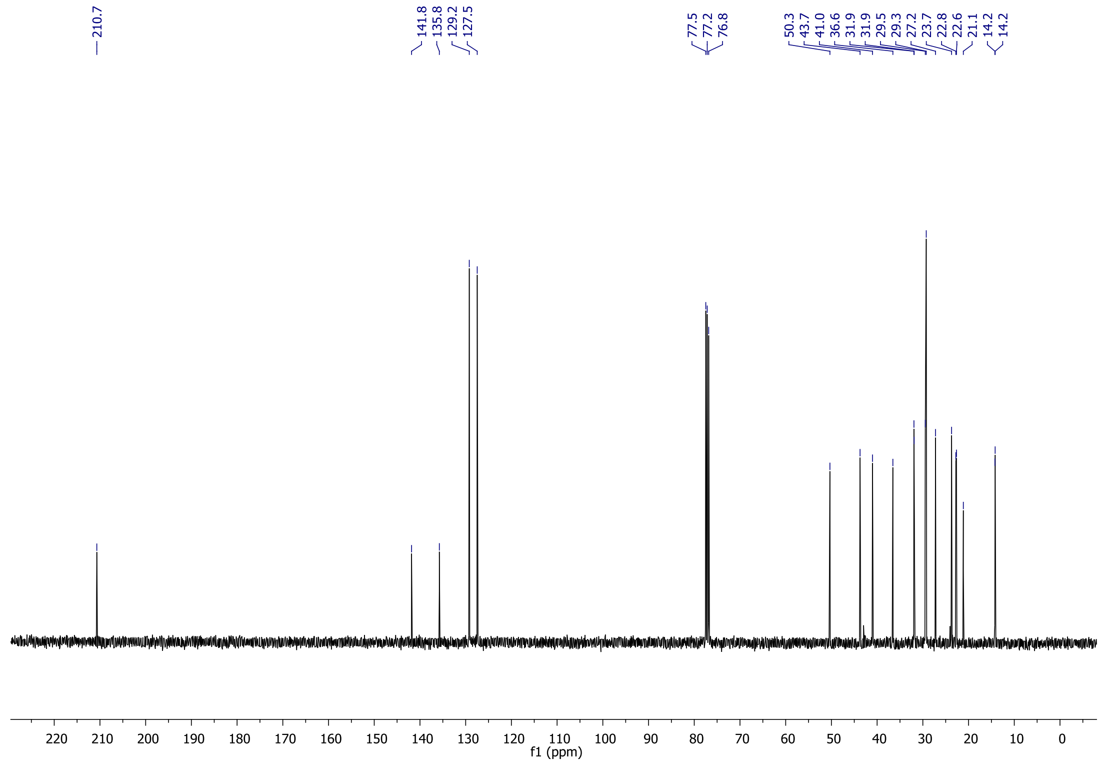

(*S*)-7-[4-(*tert*-Butyl)phenyl]-1-phenyldodecan-5-one (4d)

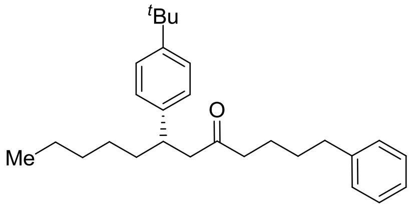

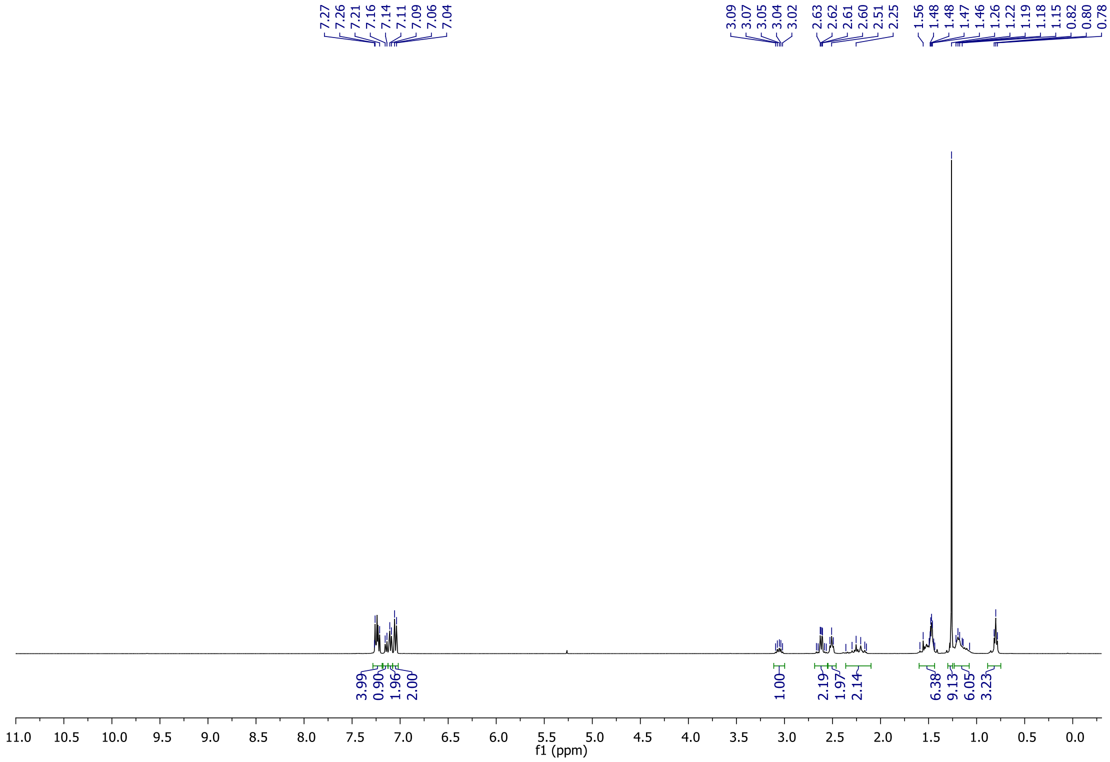

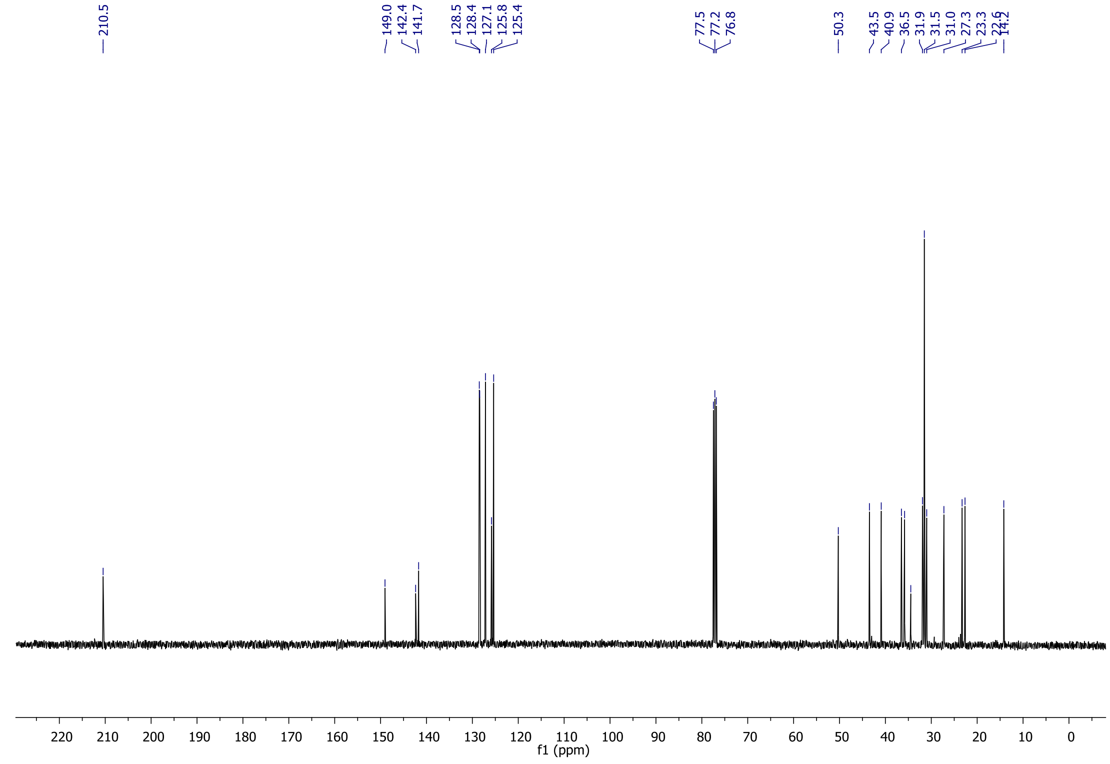

(*S*)-1,7-Diphenyldodecan-5-one (4e)

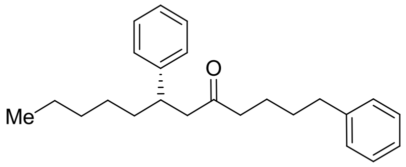

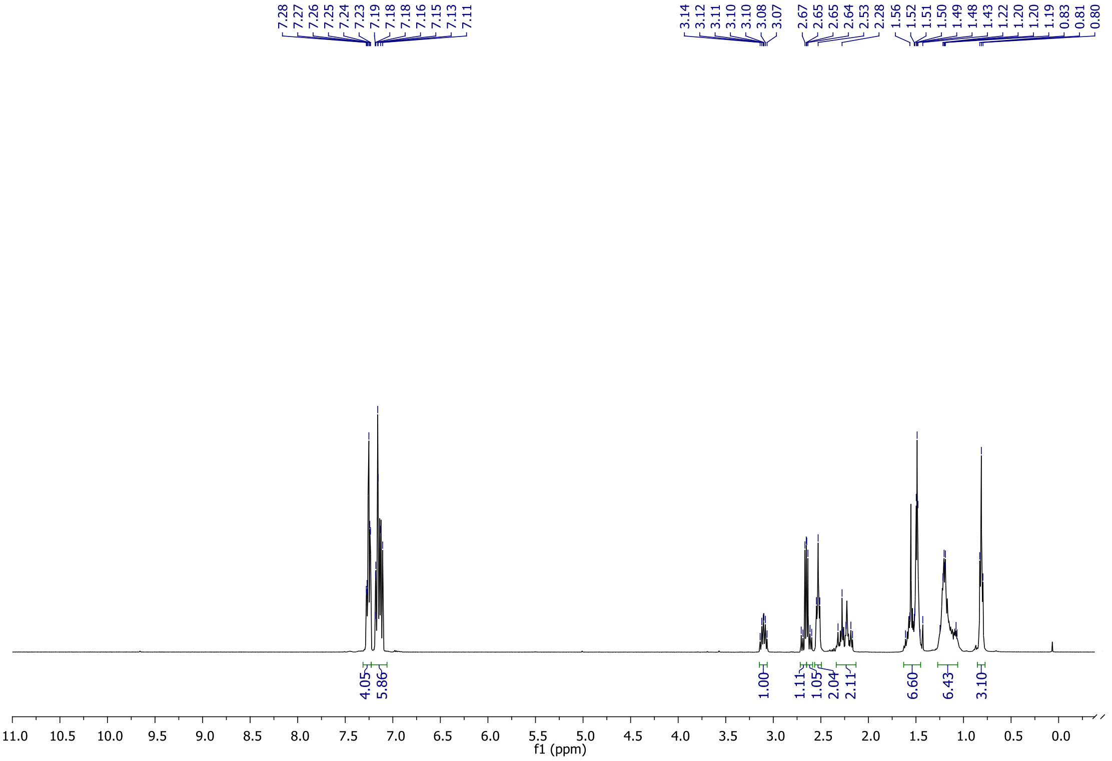

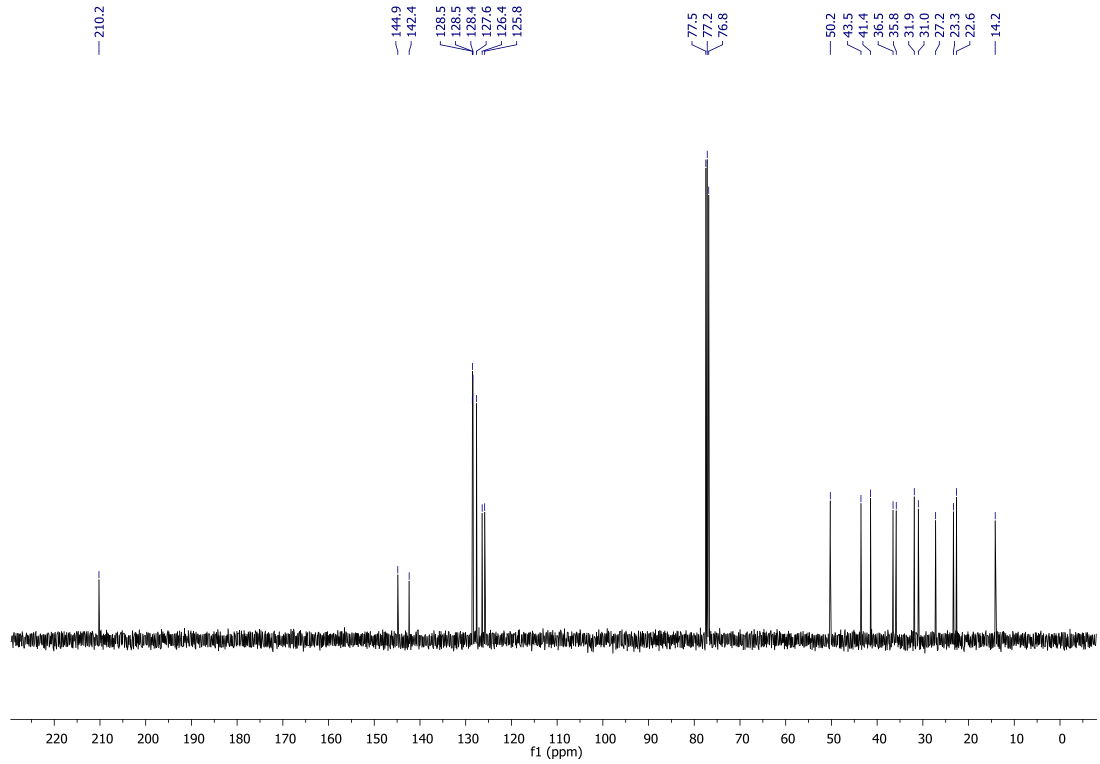

(*S*)-7-(Naphthalen-2-yl)-1-phenyldodecan-5-one (4f)

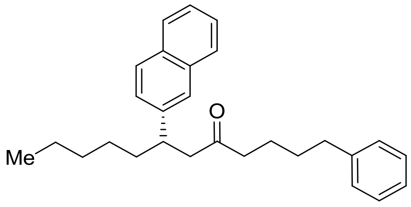

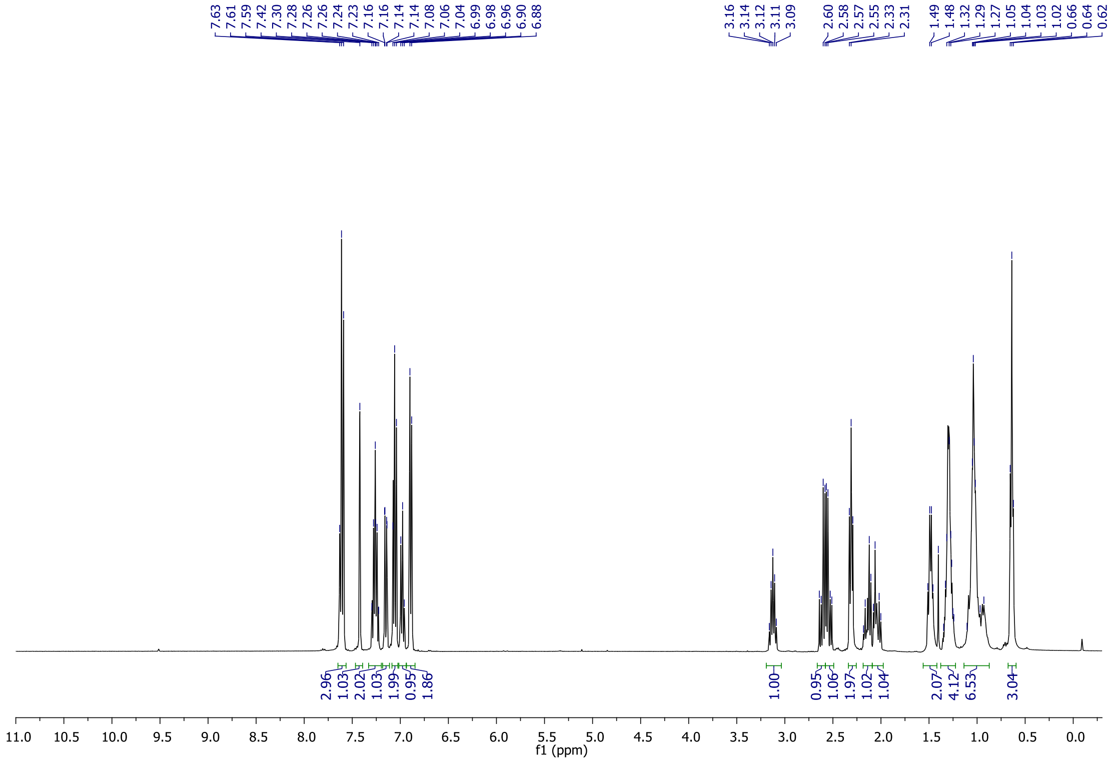

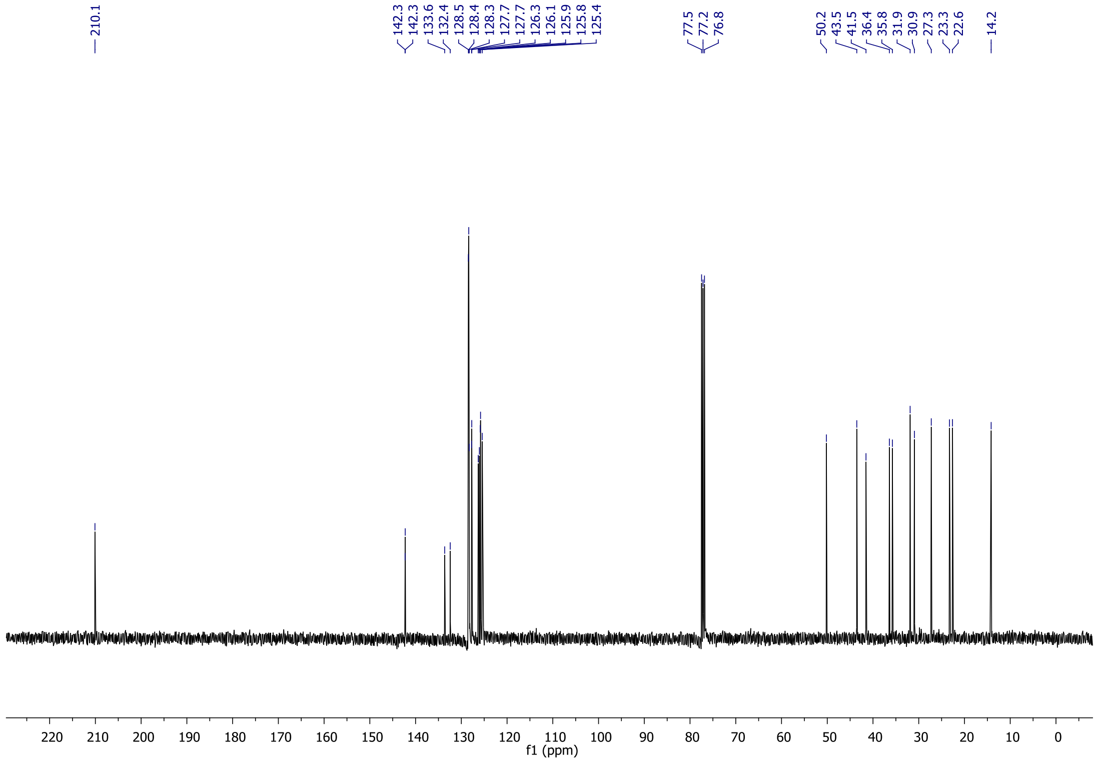

(*S*)-7-(3-Hydroxyphenyl)-1-phenyldodecan-5-one (4g)

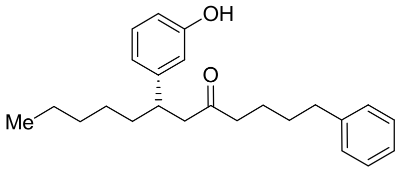

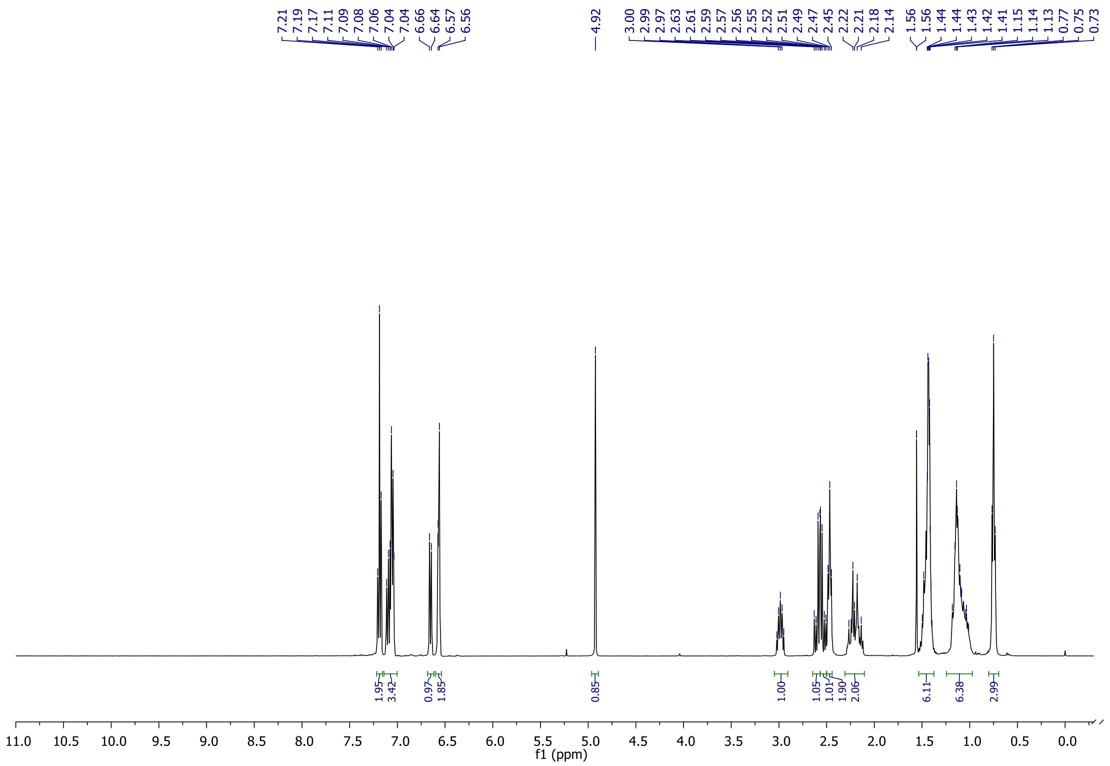

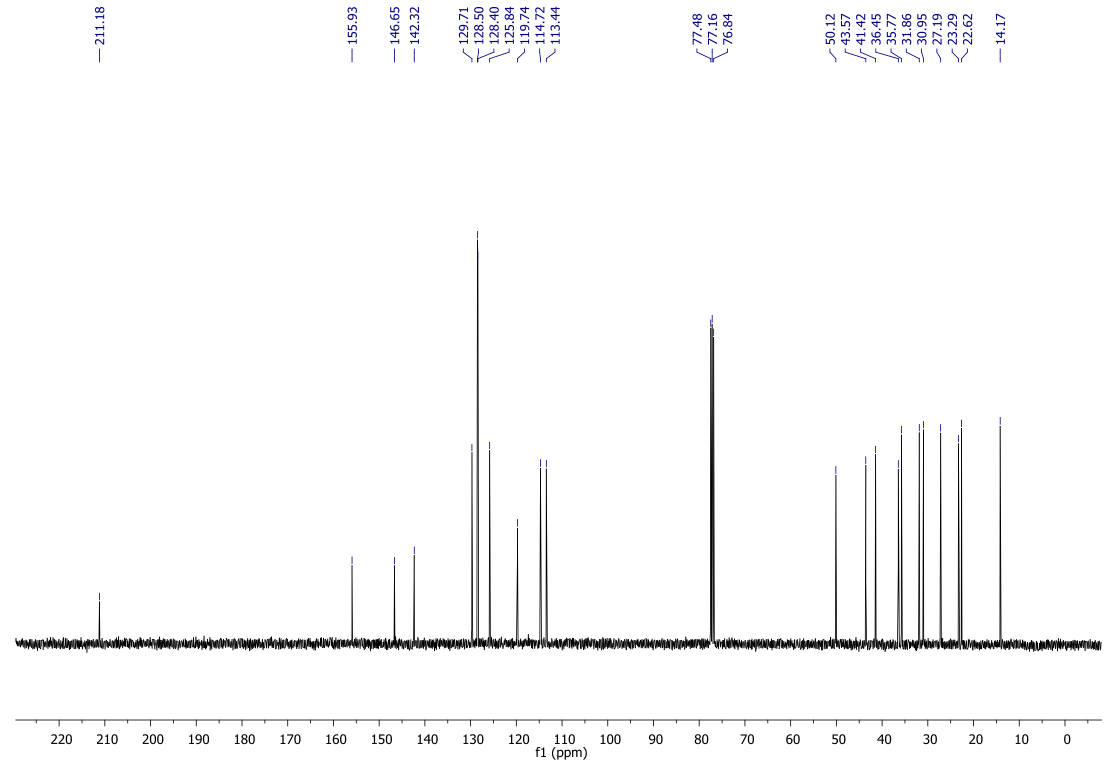

(*S*)-7-[4-(Dimethylamino)phenyl]-1-phenyldodecan-5-one (4h)

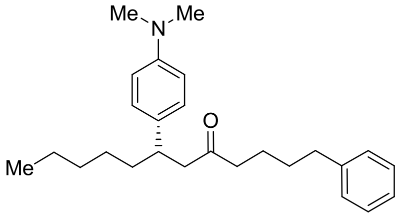

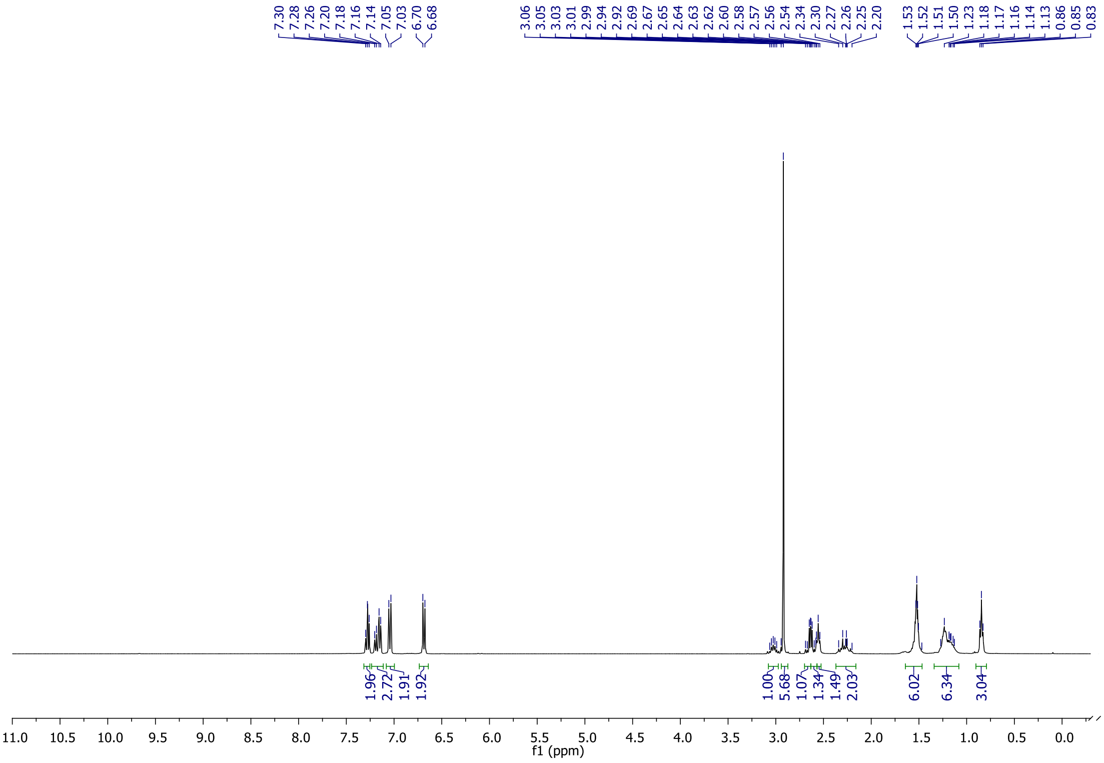

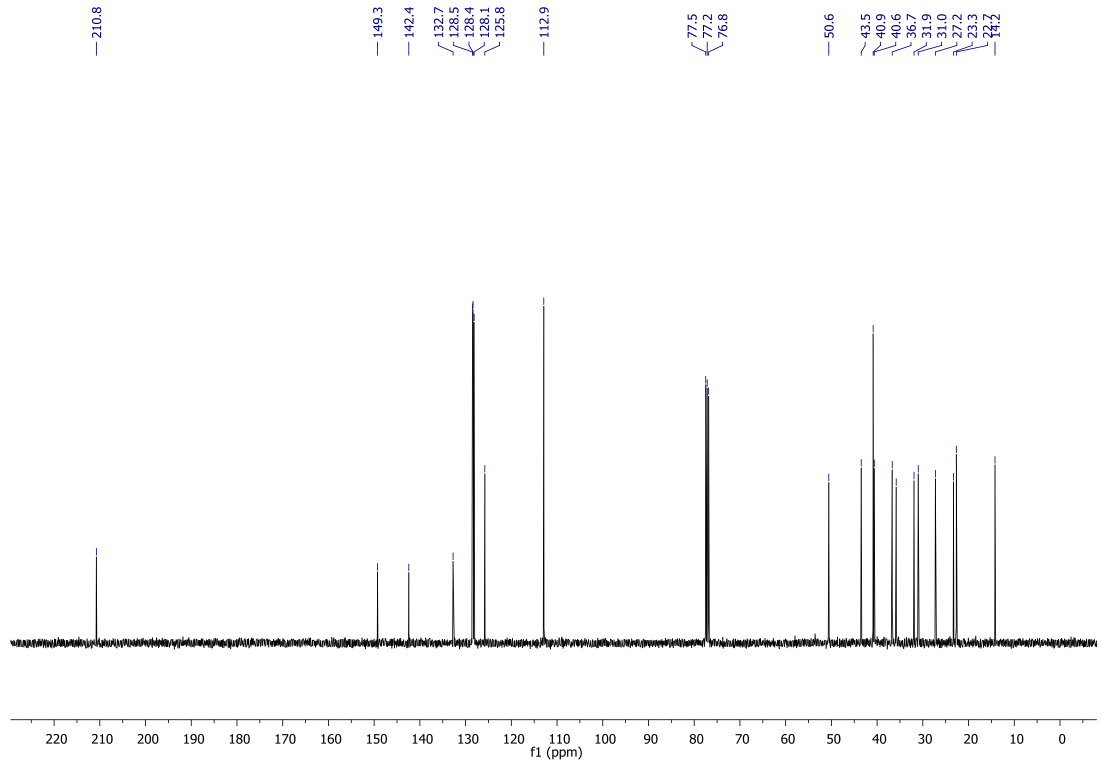

(*S*)-6-(4-Hydroxyphenyl)hexadecan-8-one (4i)

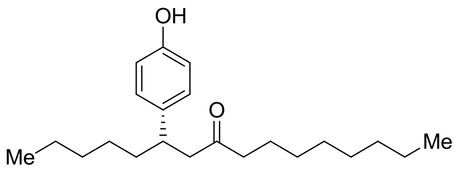

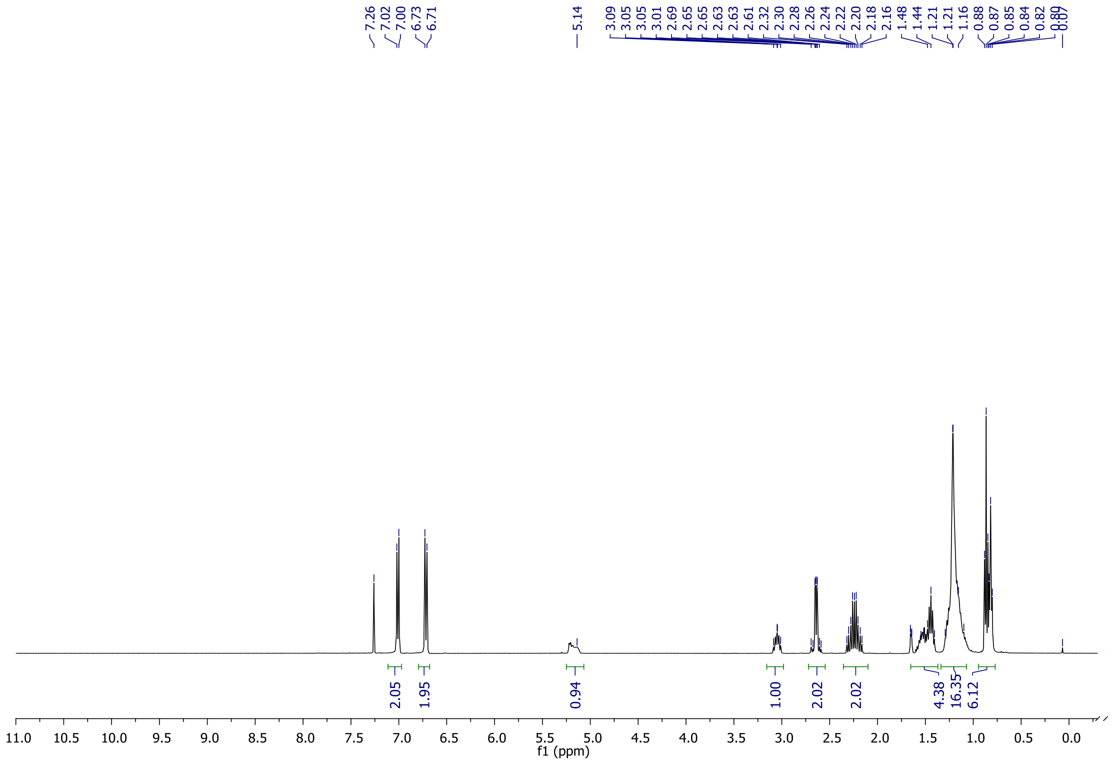

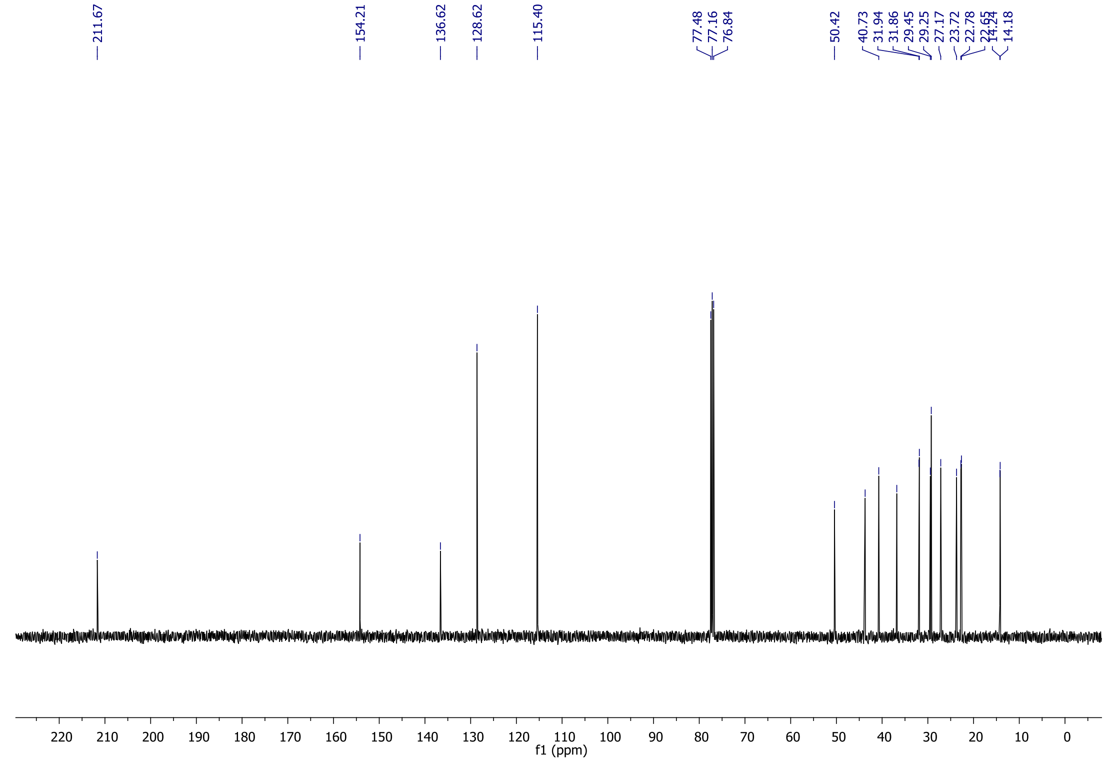

(*S*)-6-(4-Bromophenyl)hexadecan-8-one (4j)

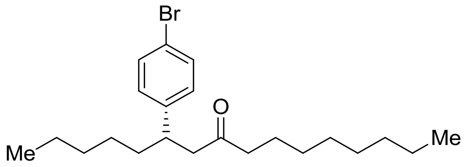

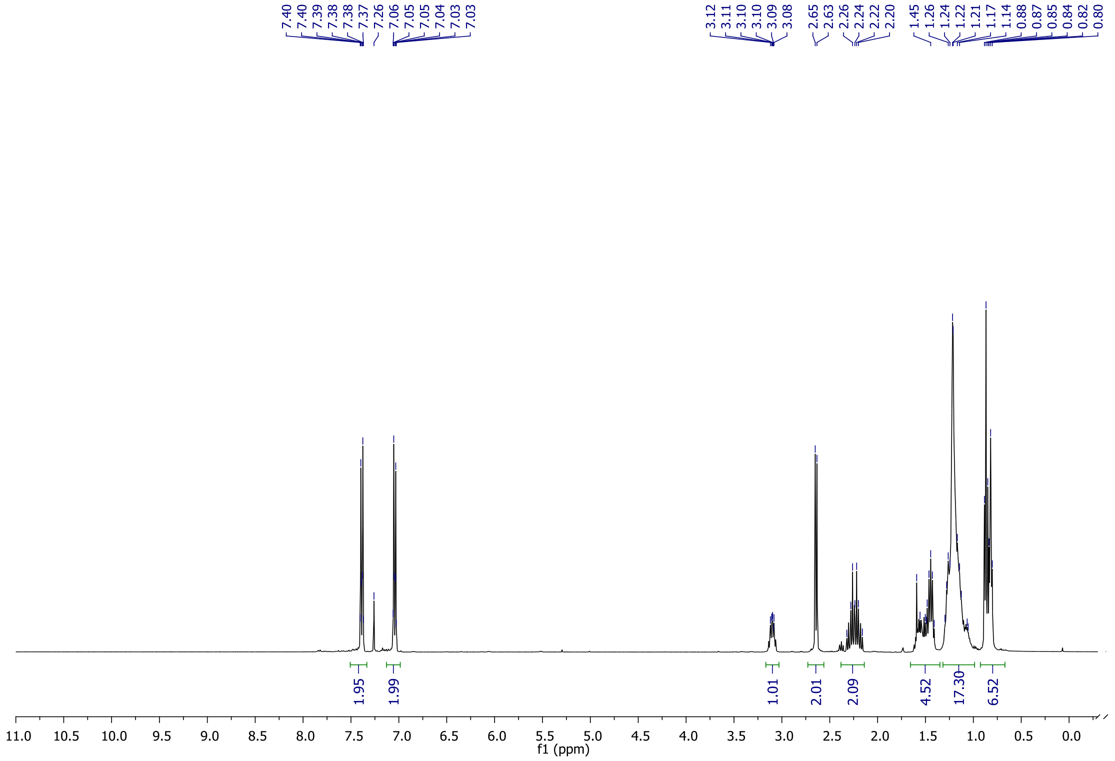

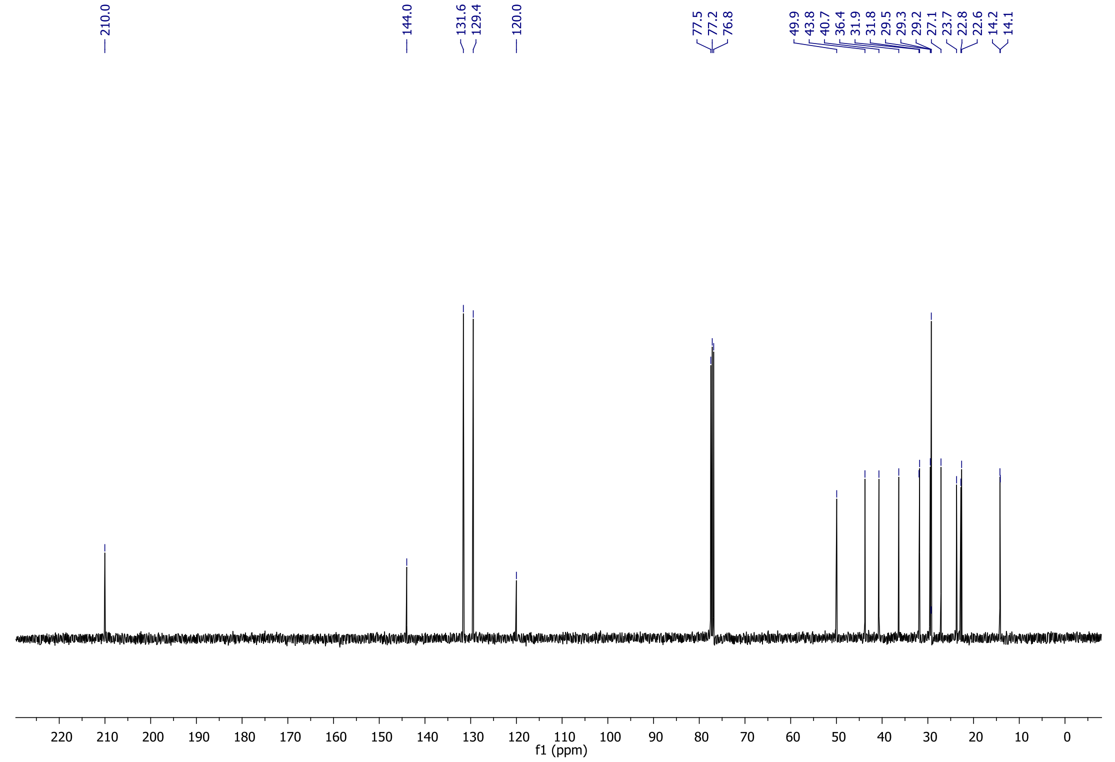

(*S*)-Methyl 4-(8-oxohexadecan-6-yl)benzoate (4k)

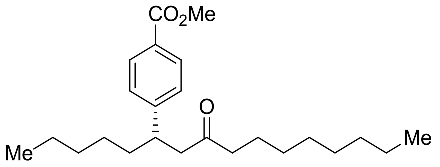

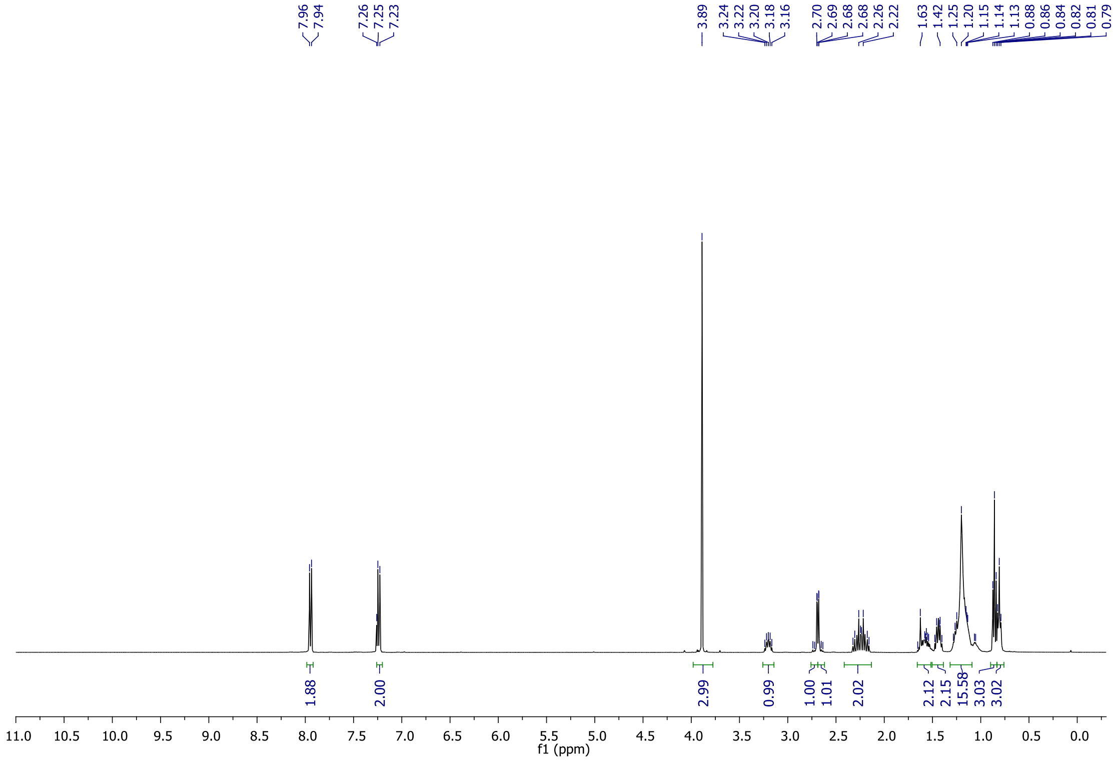

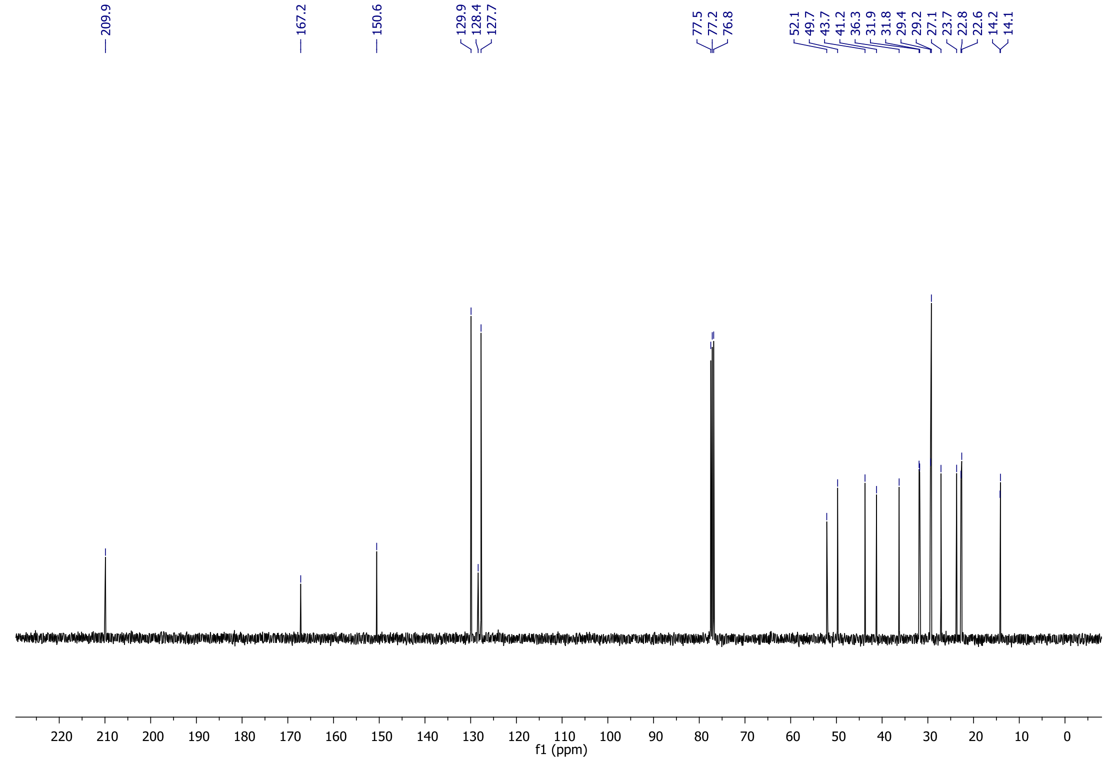

(*S*)-4-(8-Oxohexadecan-6-yl)benzonitrile (4l)

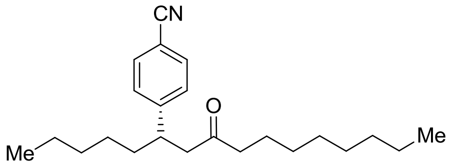

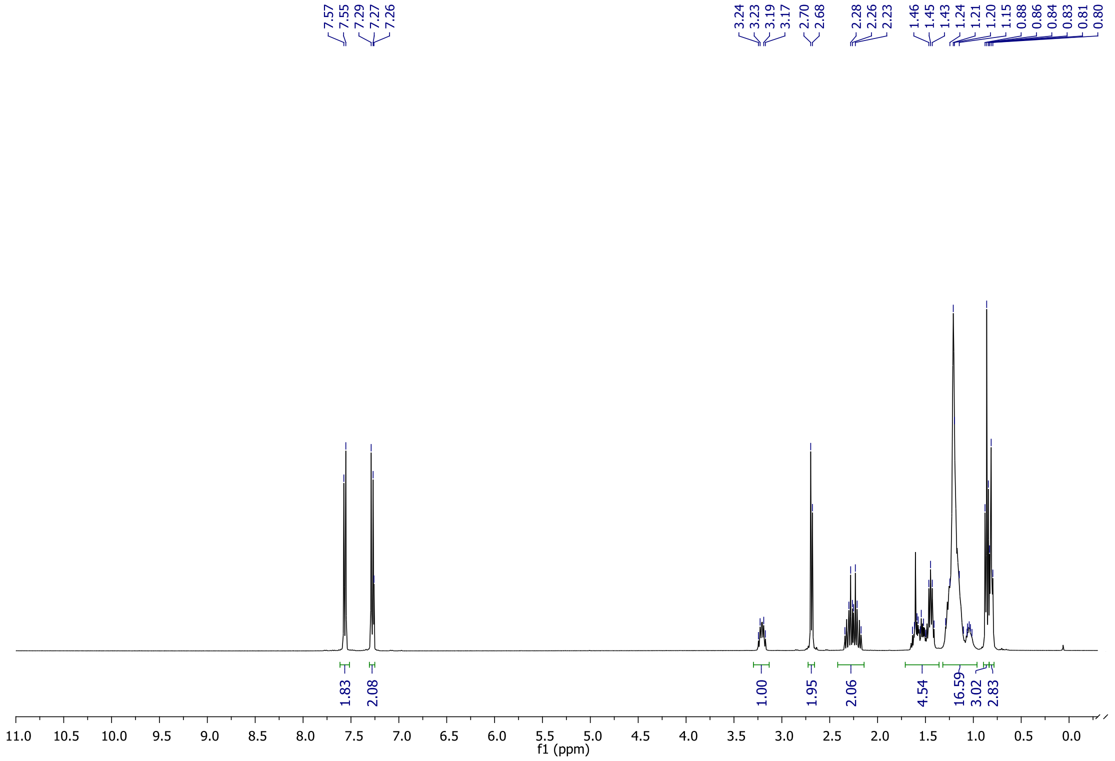

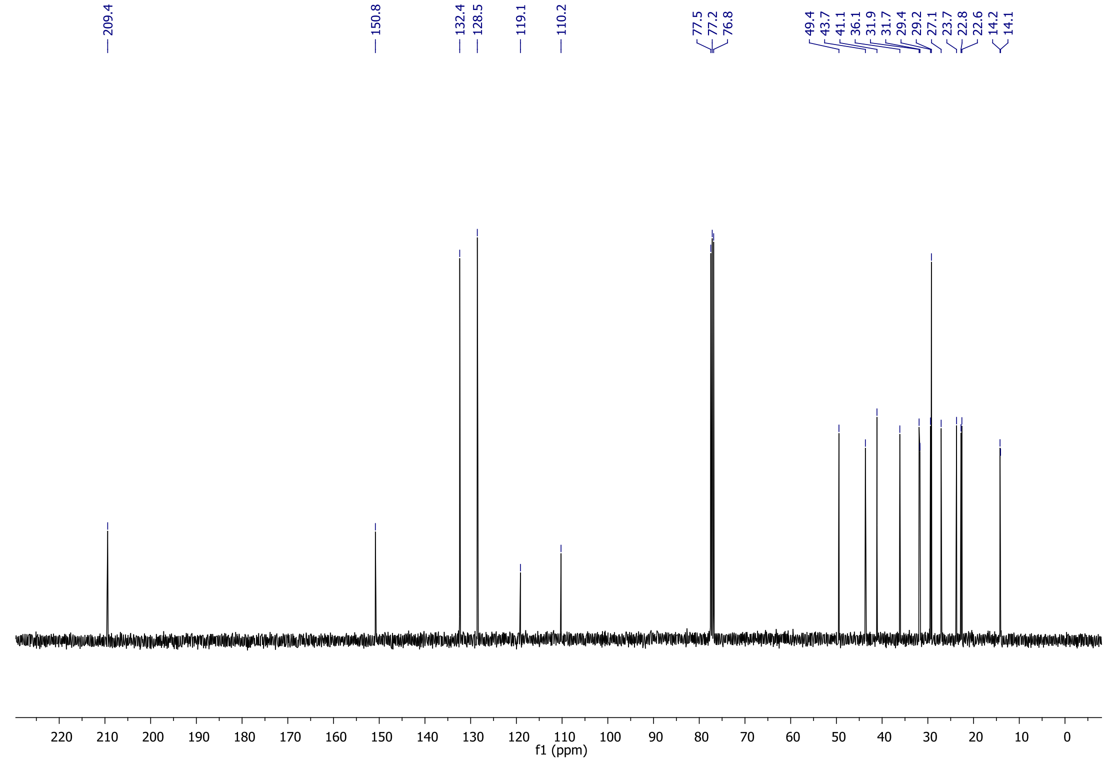

(*S*)-7-(2,3-Dihydrobenzo[b][1,4]dioxin-6-yl)-1-phenyldodecan-5-one (4m)

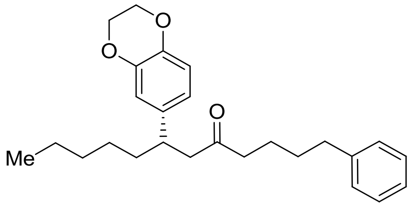

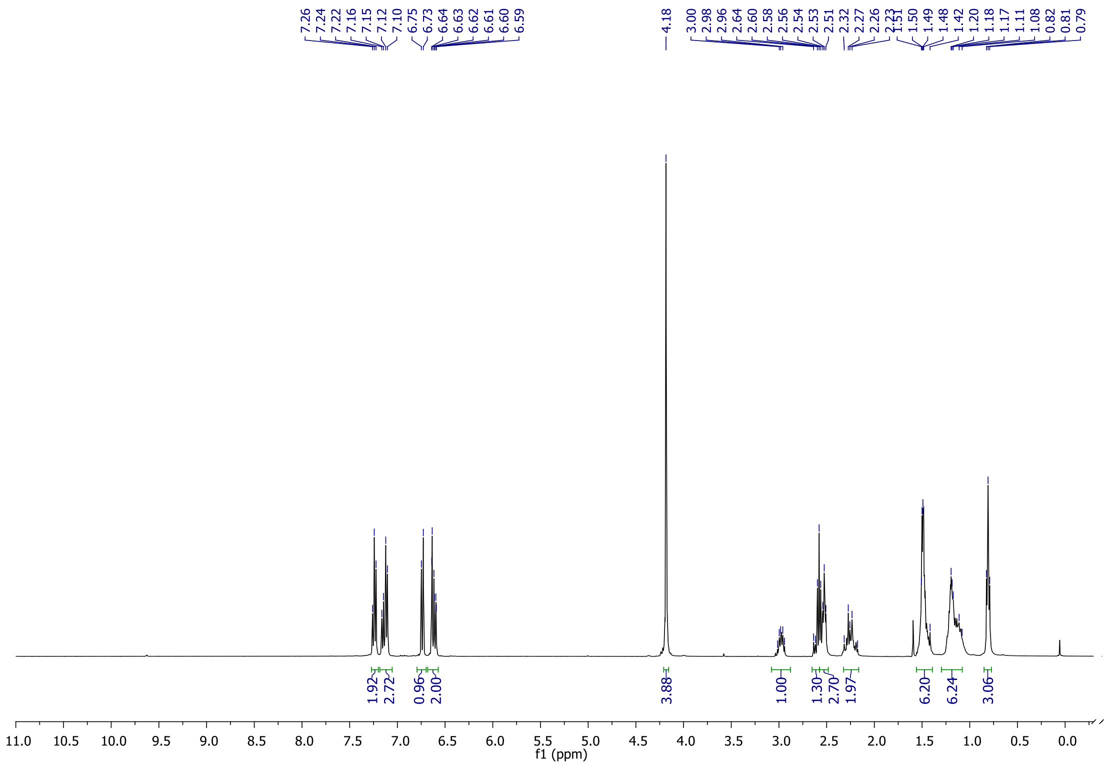

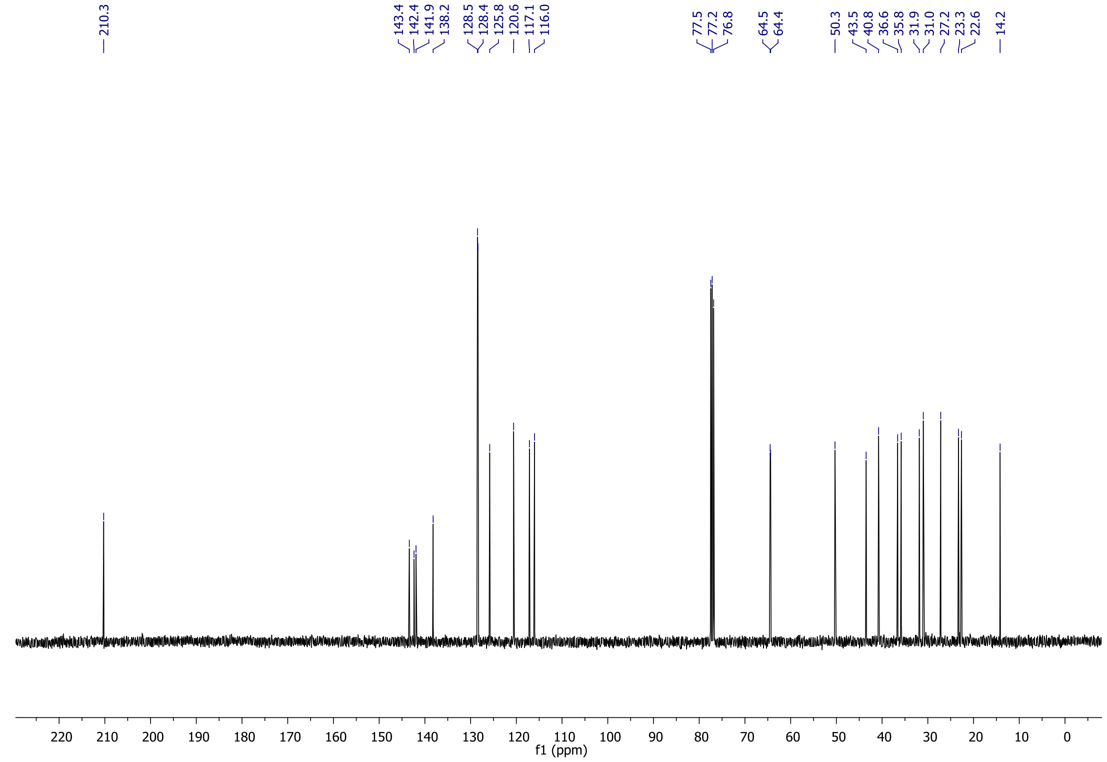

(*S*)-1-Phenyl-7-(thiophen-3-yl)dodecan-5-one (4n)

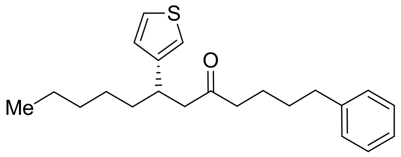

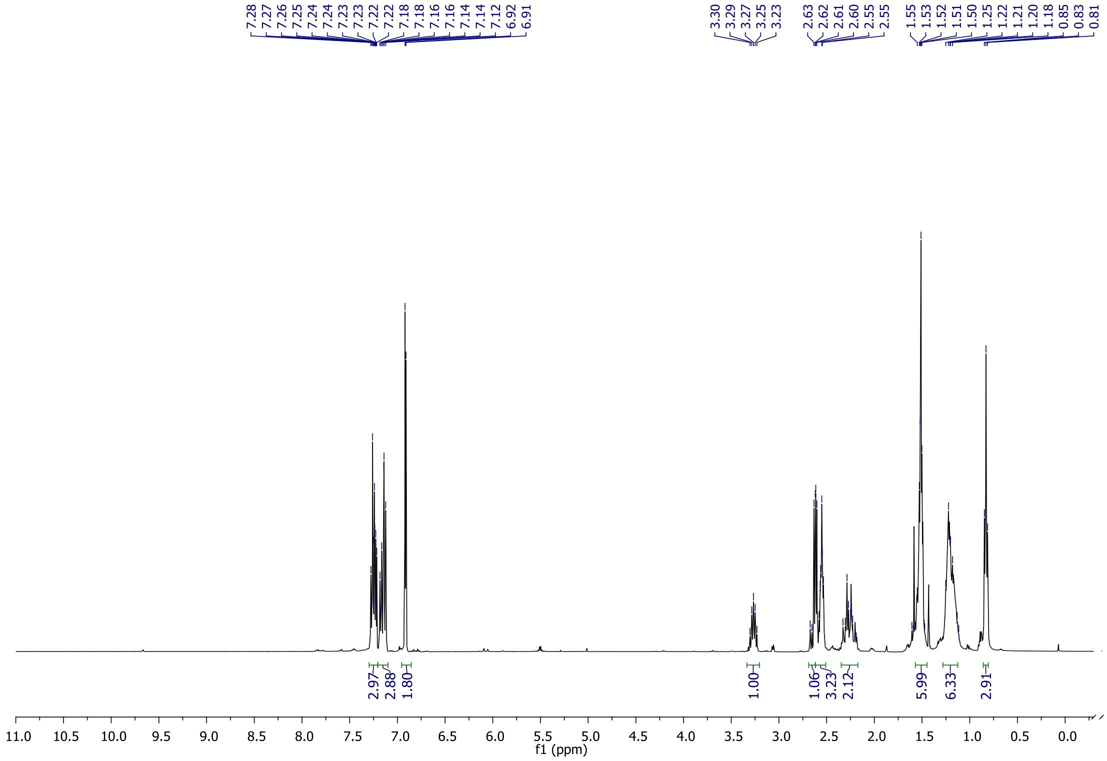

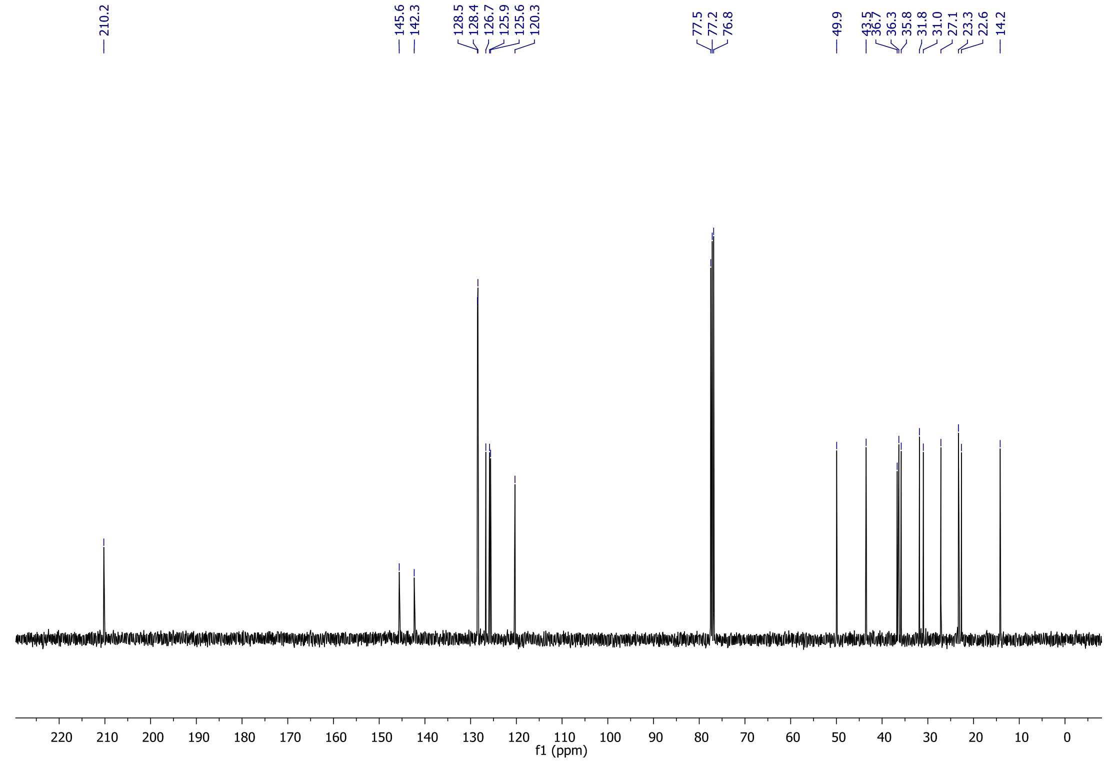

(*S*)-2-[8-(Cyclohex-1-en-1-yl)-6-oxotridecyl]isoindoline-1,3-dione (4o)

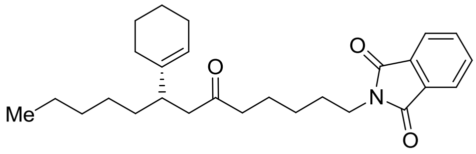

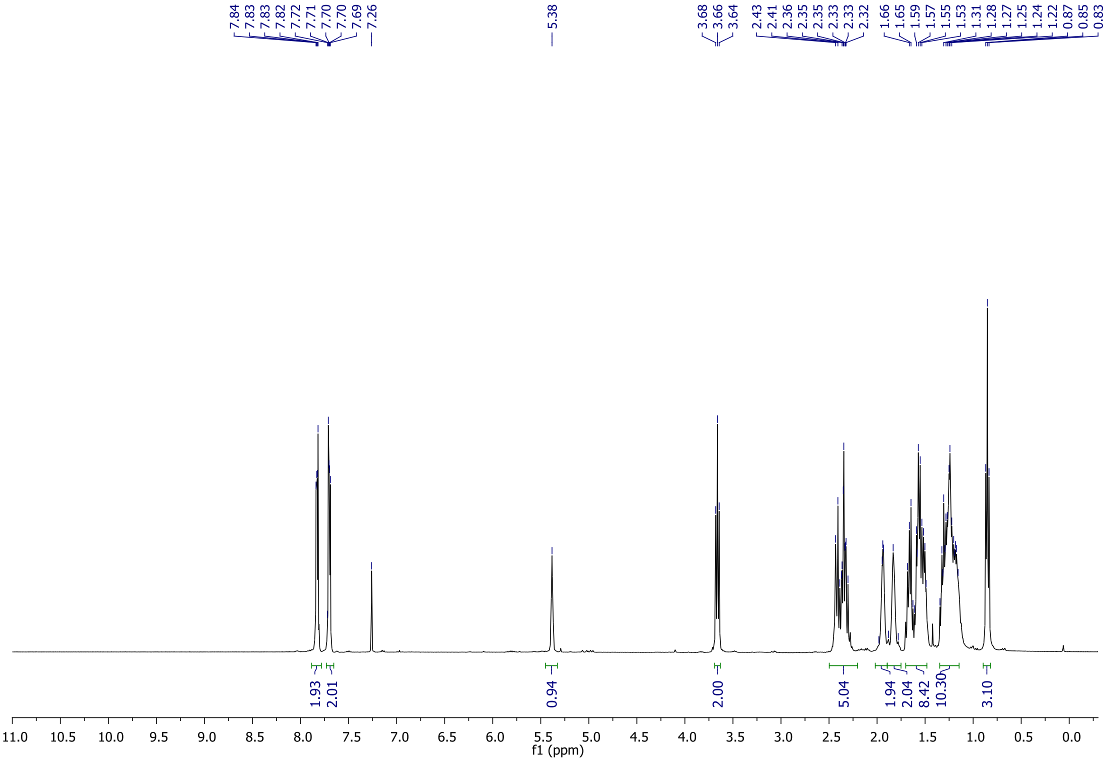

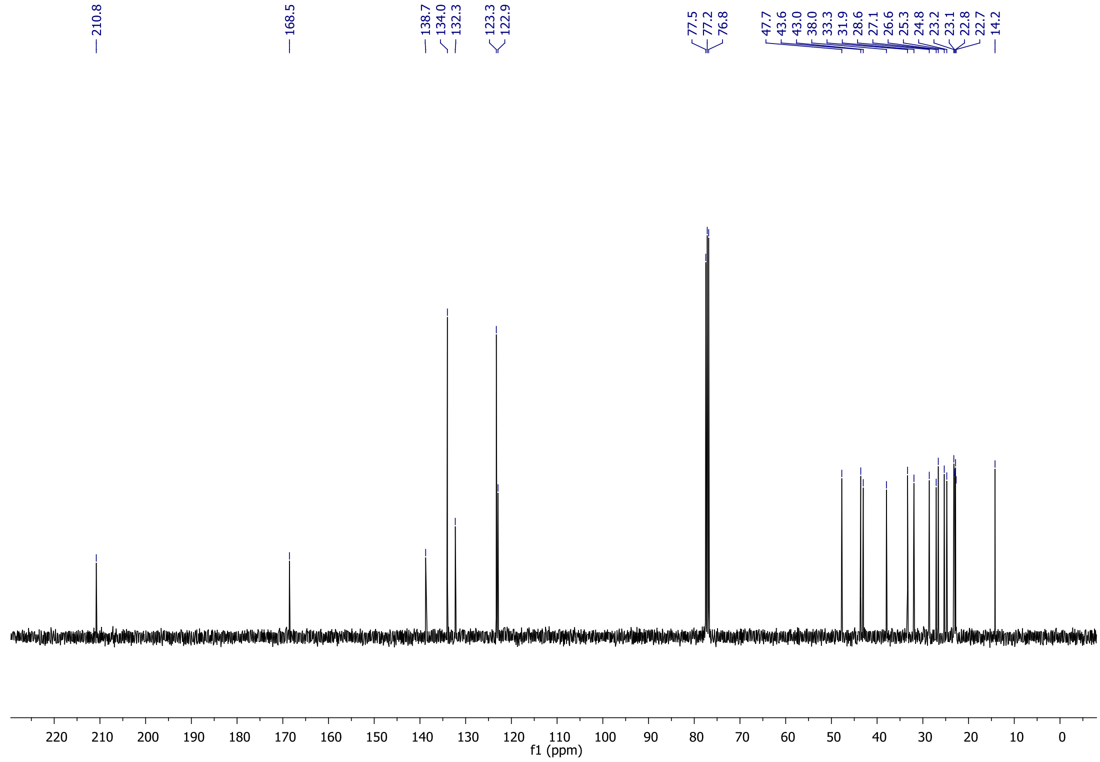

(*S*)-1-Cyclopentyl-6-(4-methoxyphenyl)undecan-4-one (4p)

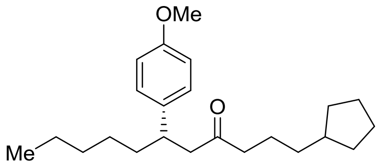

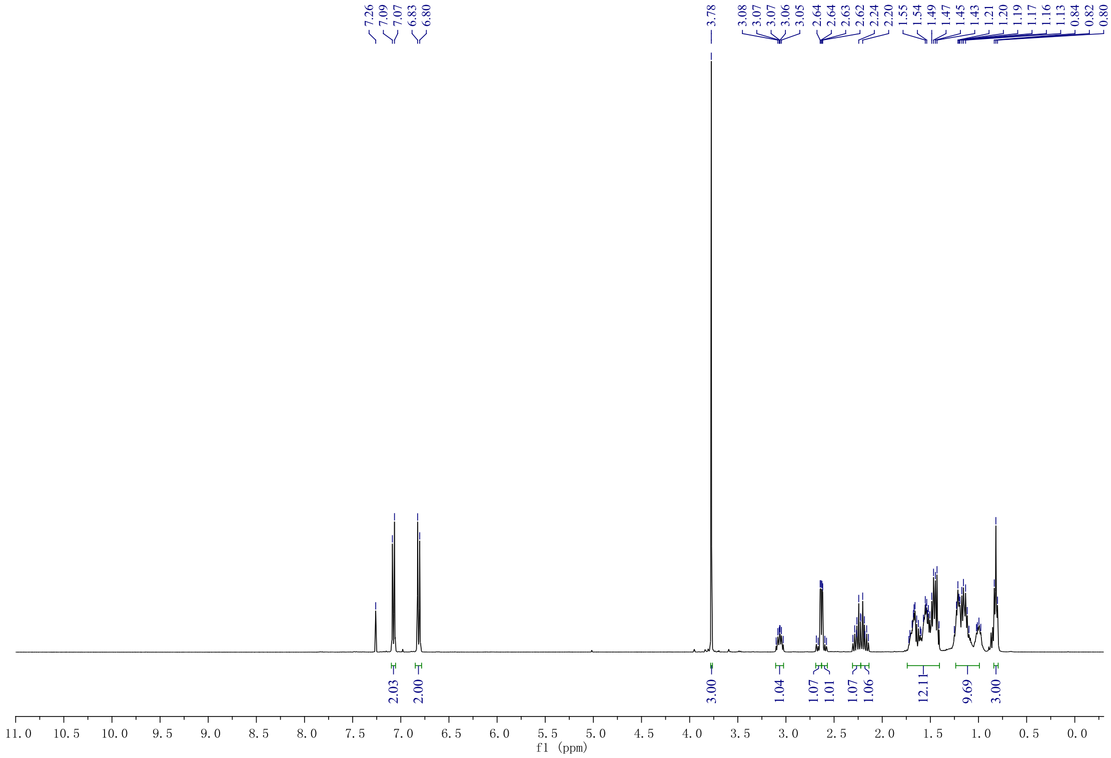

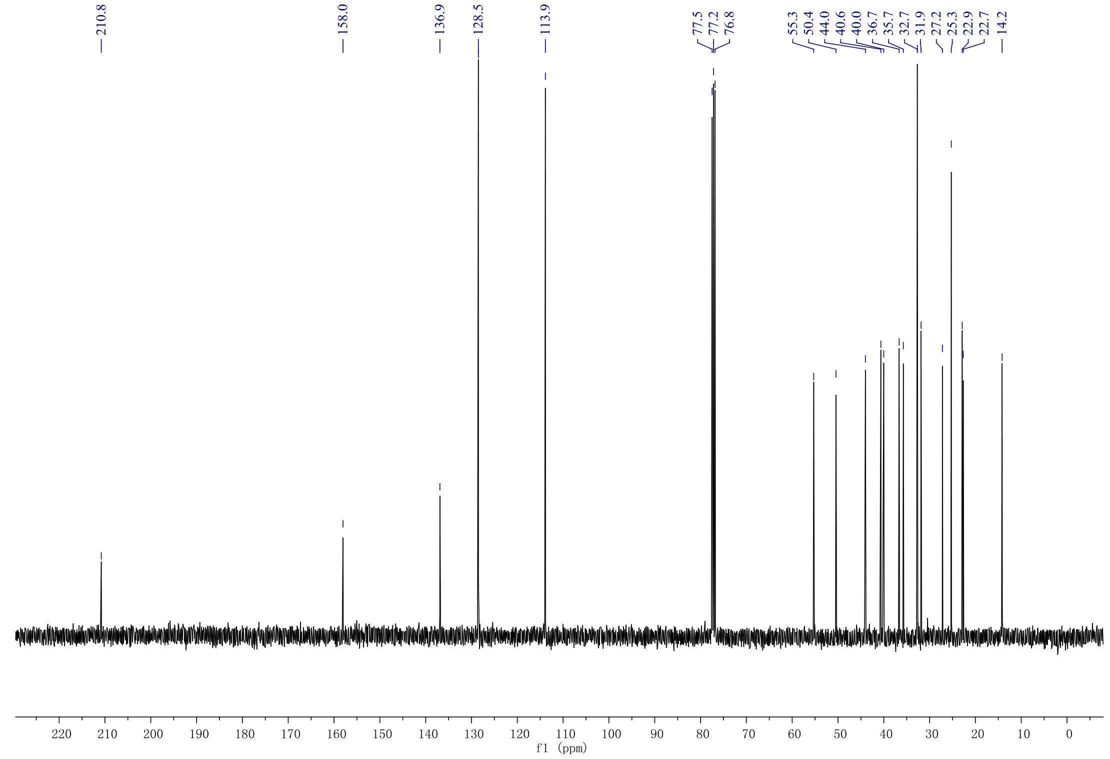

(*S*)-7-(4-Methoxyphenyl)-1-phenyldodecan-5-one (4q)

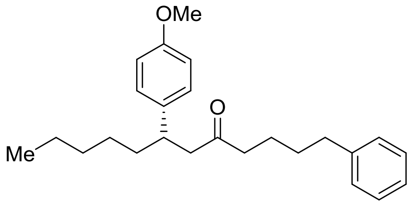

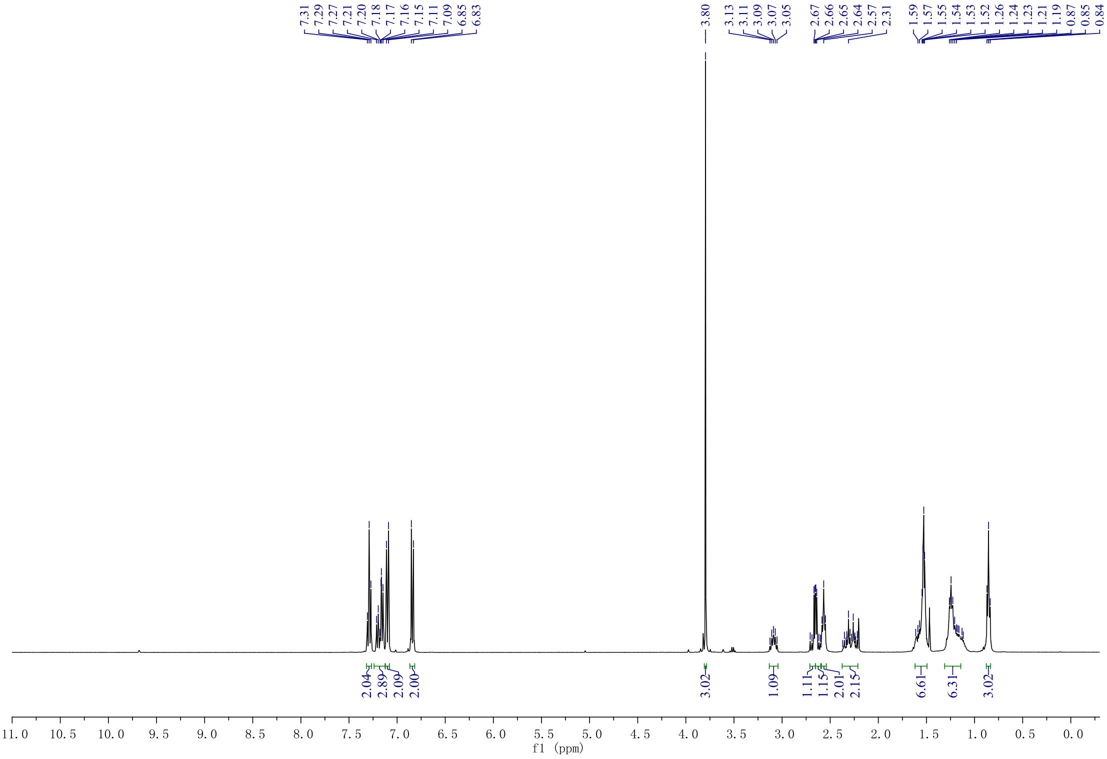

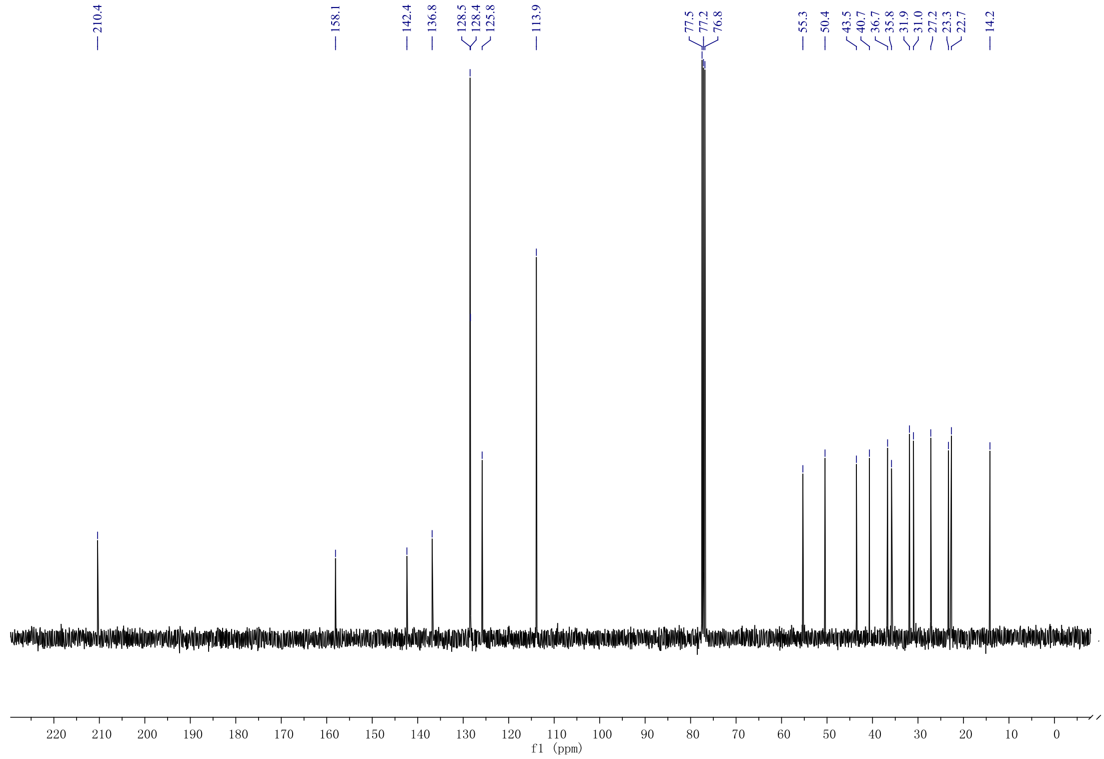

(*S*)-9-(4-Methoxyphenyl)tetradecane-2,7-dione (4r)

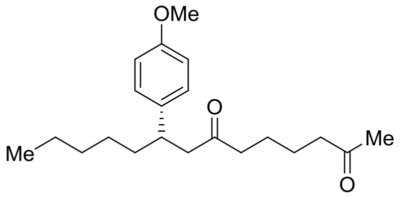

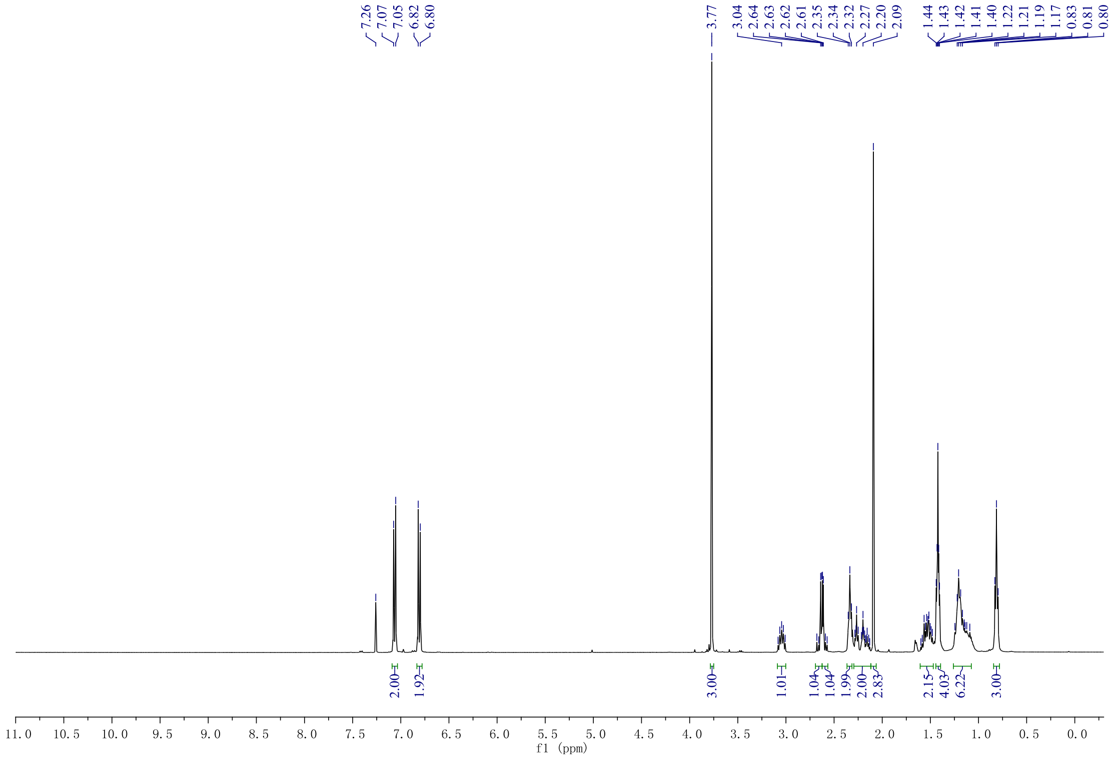

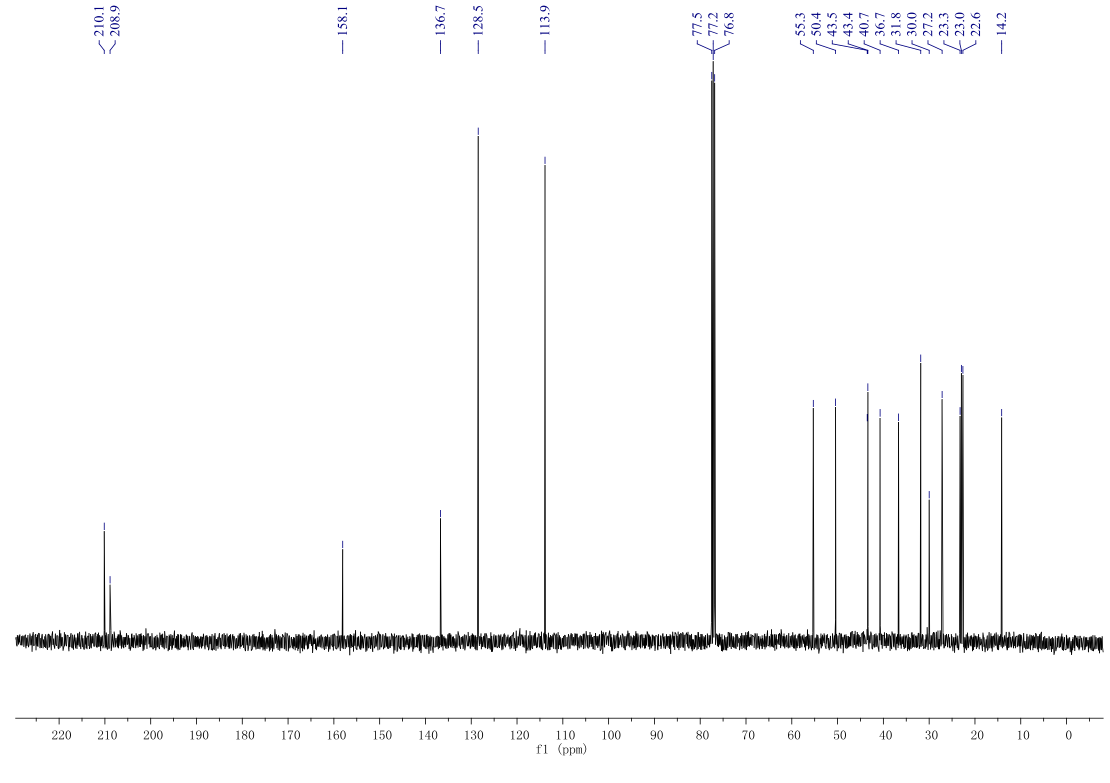

(*S*)-Diethyl 2-[6-(4-methoxyphenyl)-4-oxoundecyl]malonate (4s)

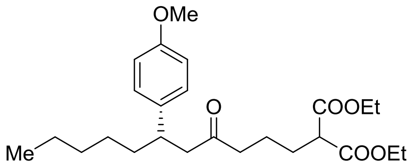

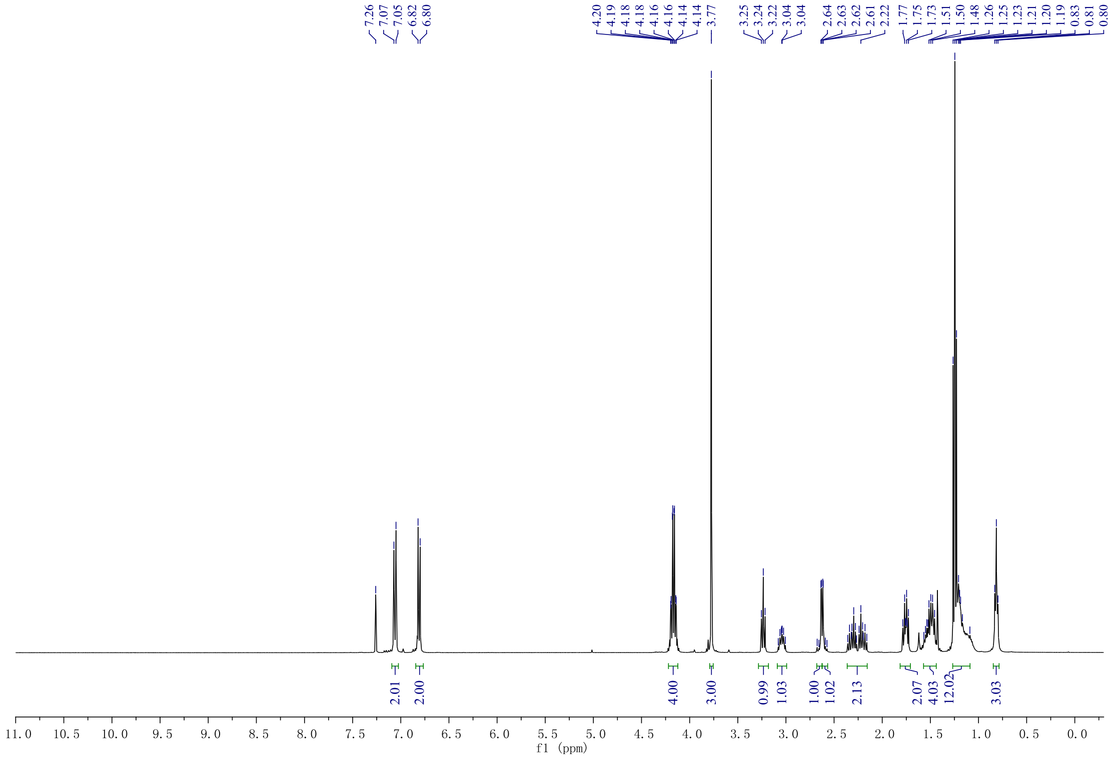

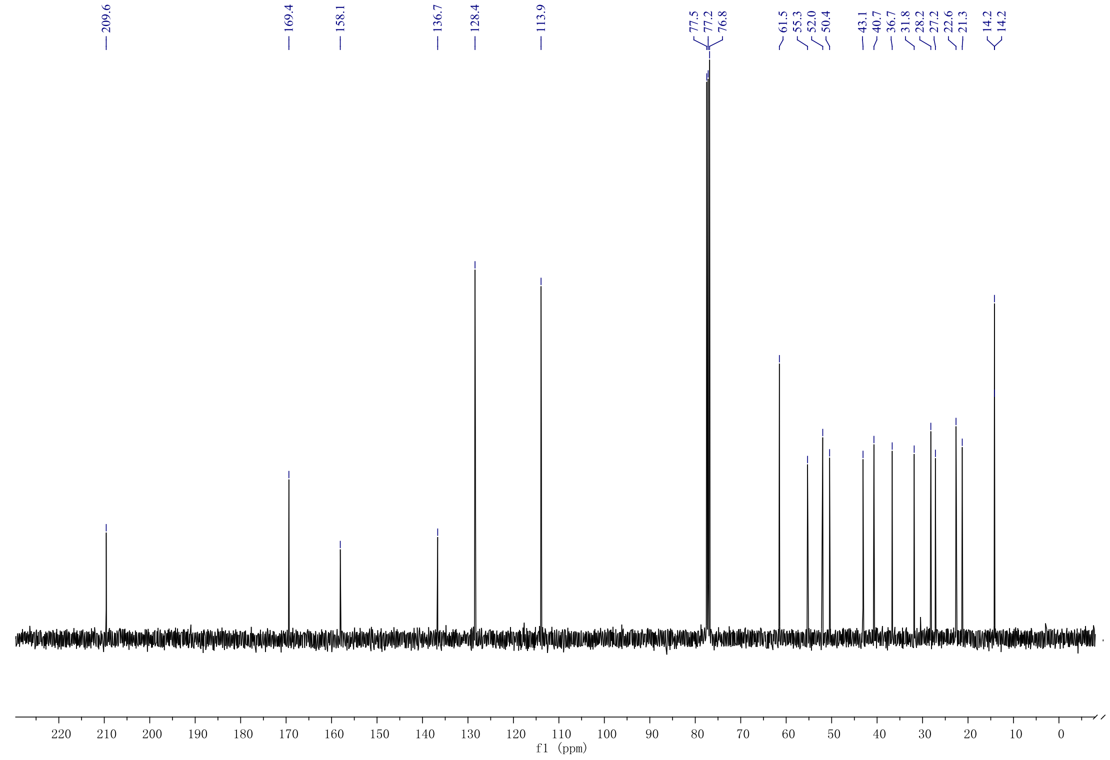

(*S*)-1-Bromo-9-(4-methoxyphenyl)tetradecan-7-one (4t)

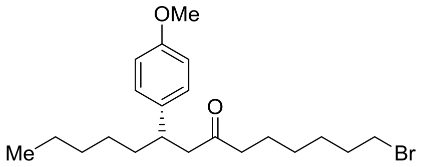

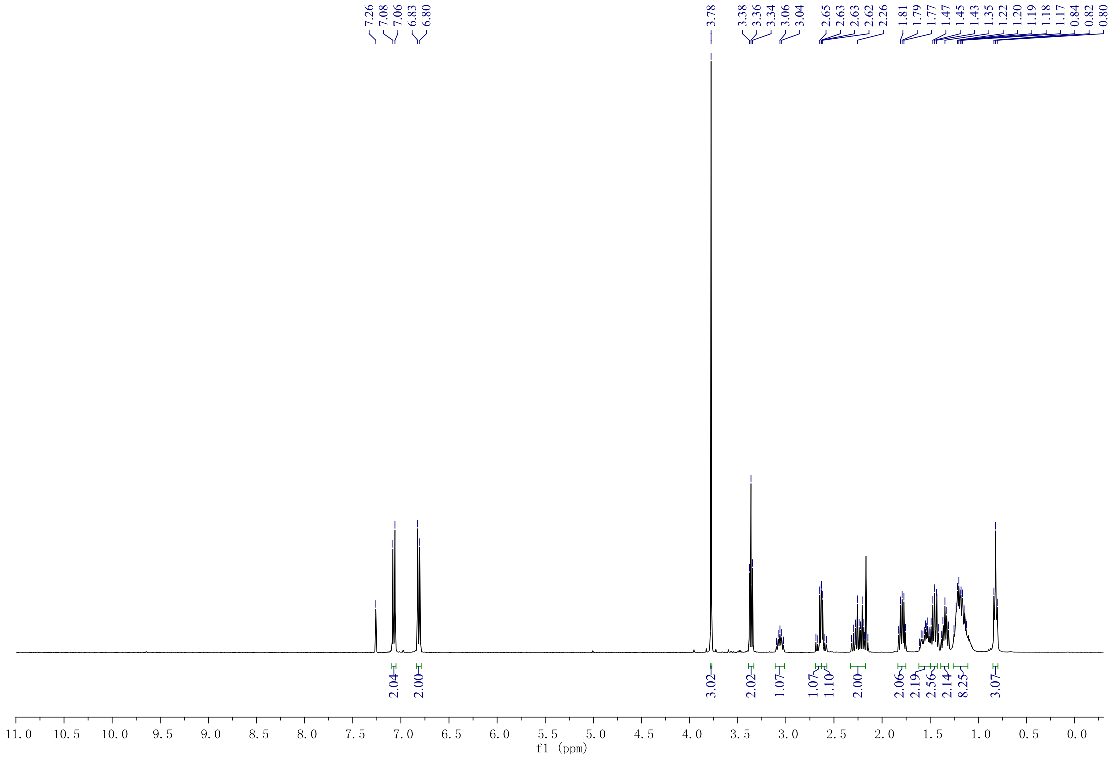

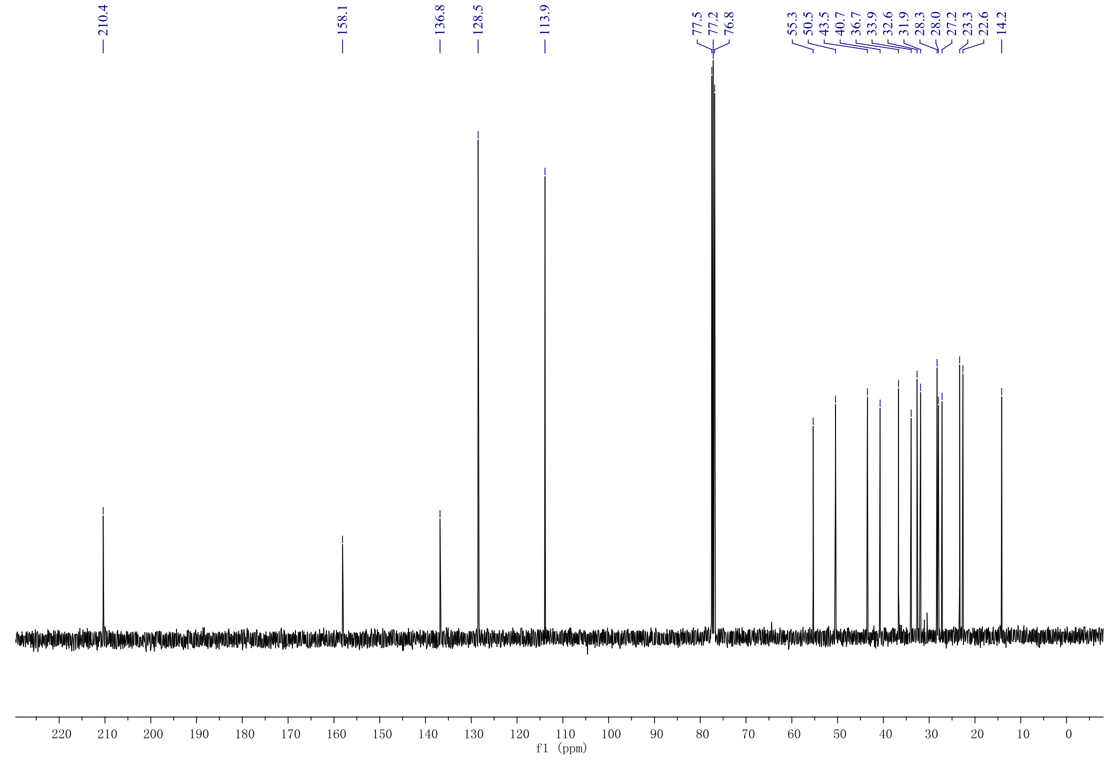

(*S*)-1,1-Diethoxy-6-(4-methoxyphenyl)undecan-4-one (4u)

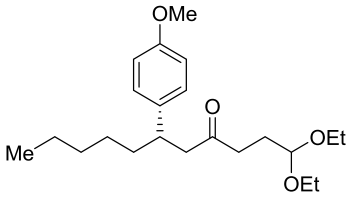

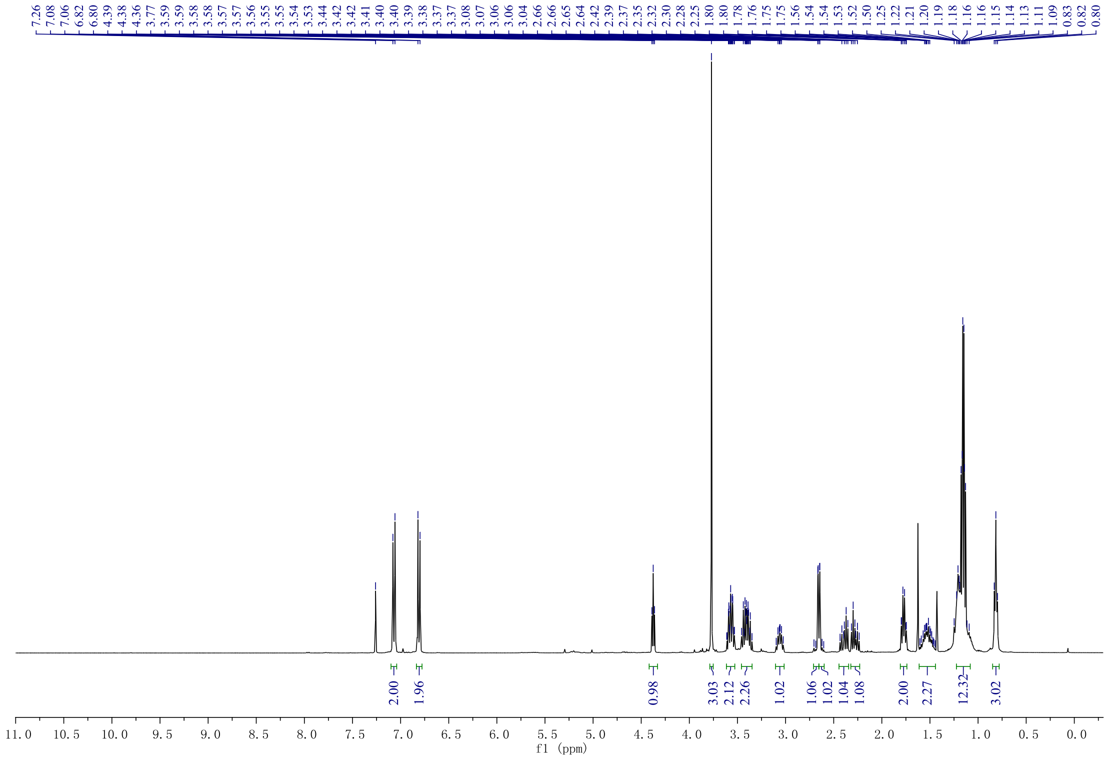

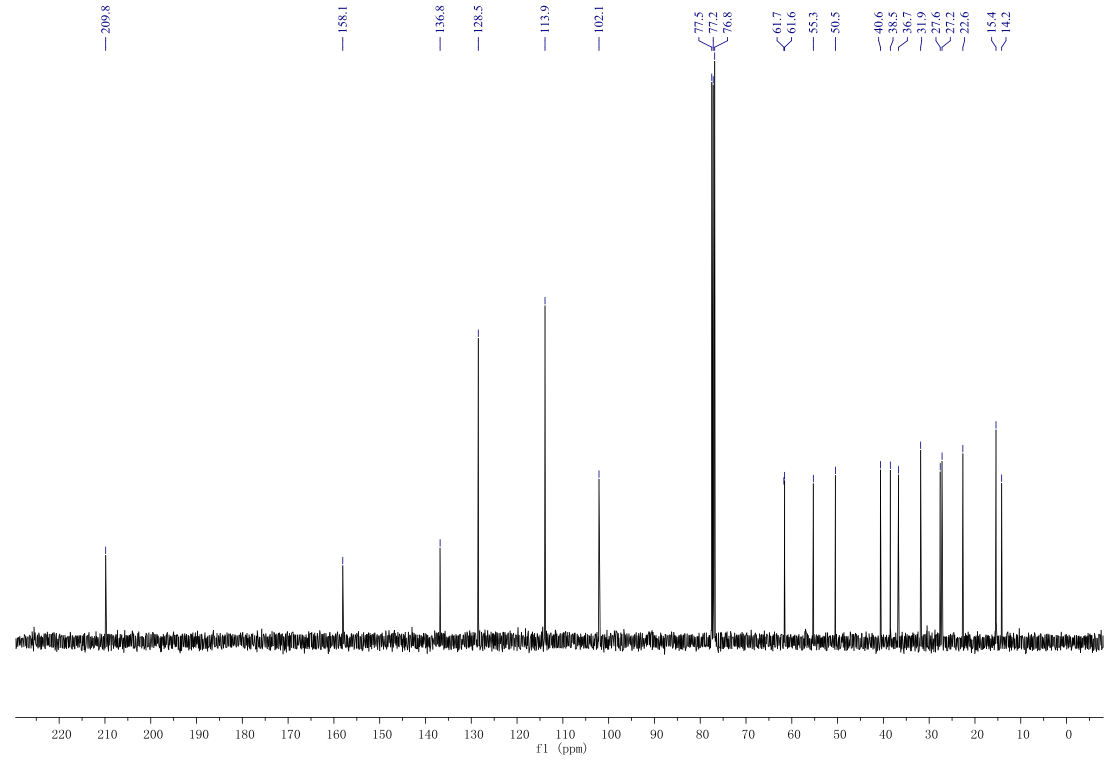

(*S*)-*N*,*N*-Di-Boc-1-amino-7-(4-methoxyphenyl)dodecan-5-one (4w)

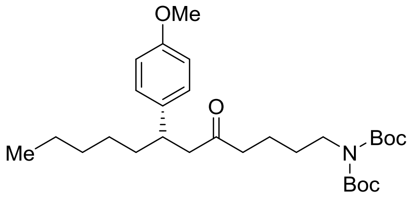

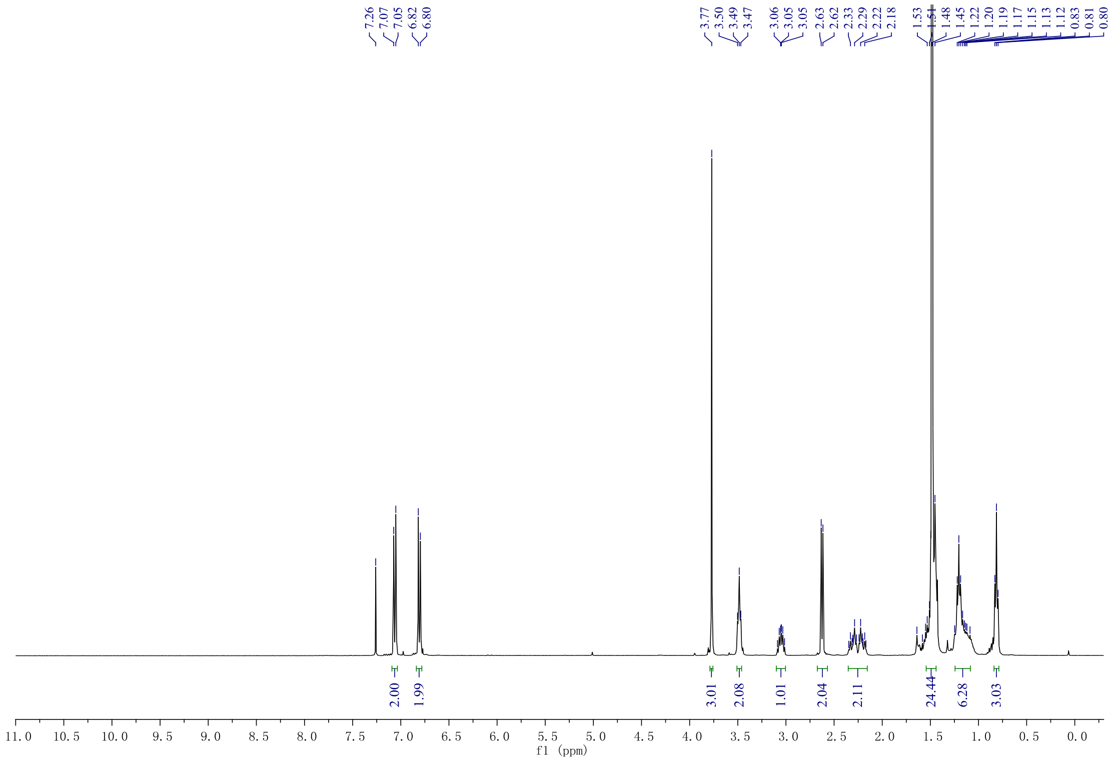

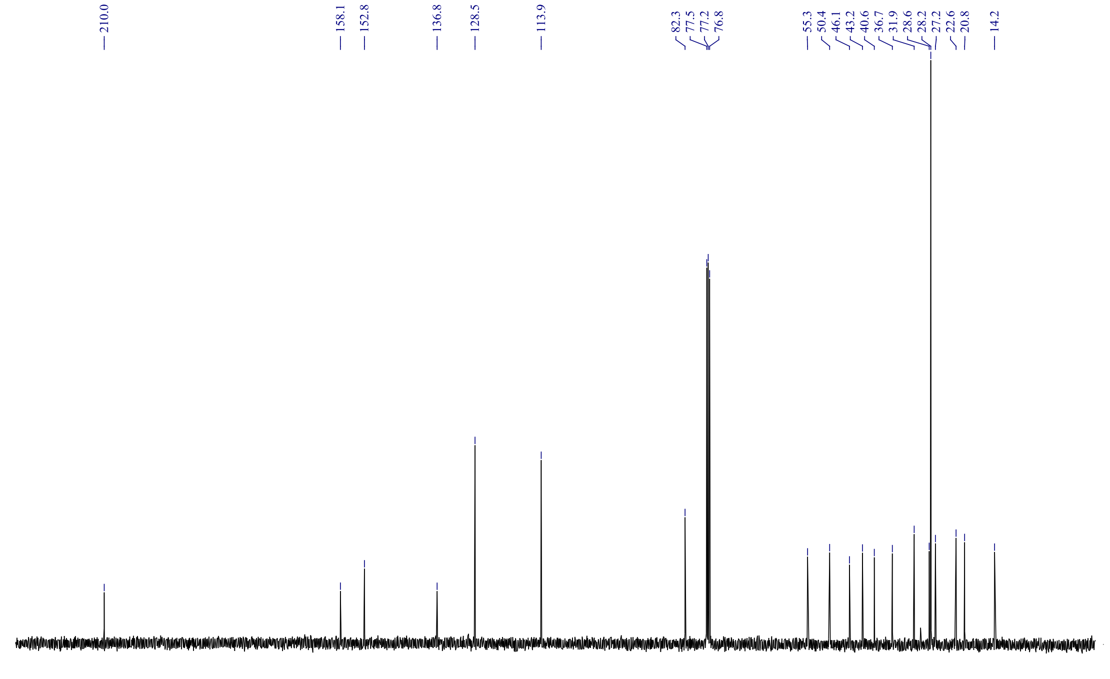

(*S*)-9-(4-Methoxyphenyl)-7-oxotetradecyl acetate (4x)

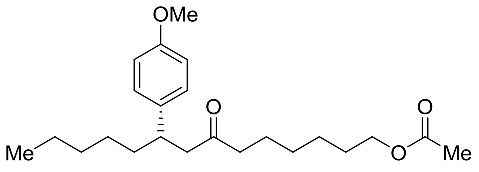

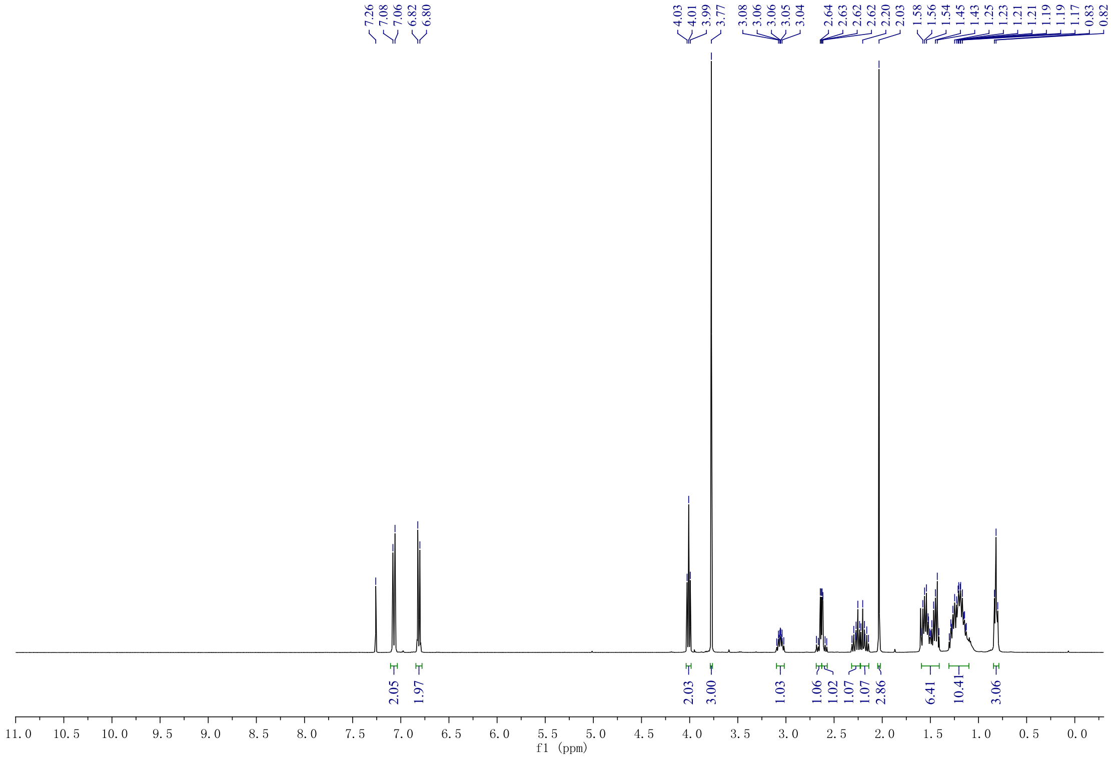

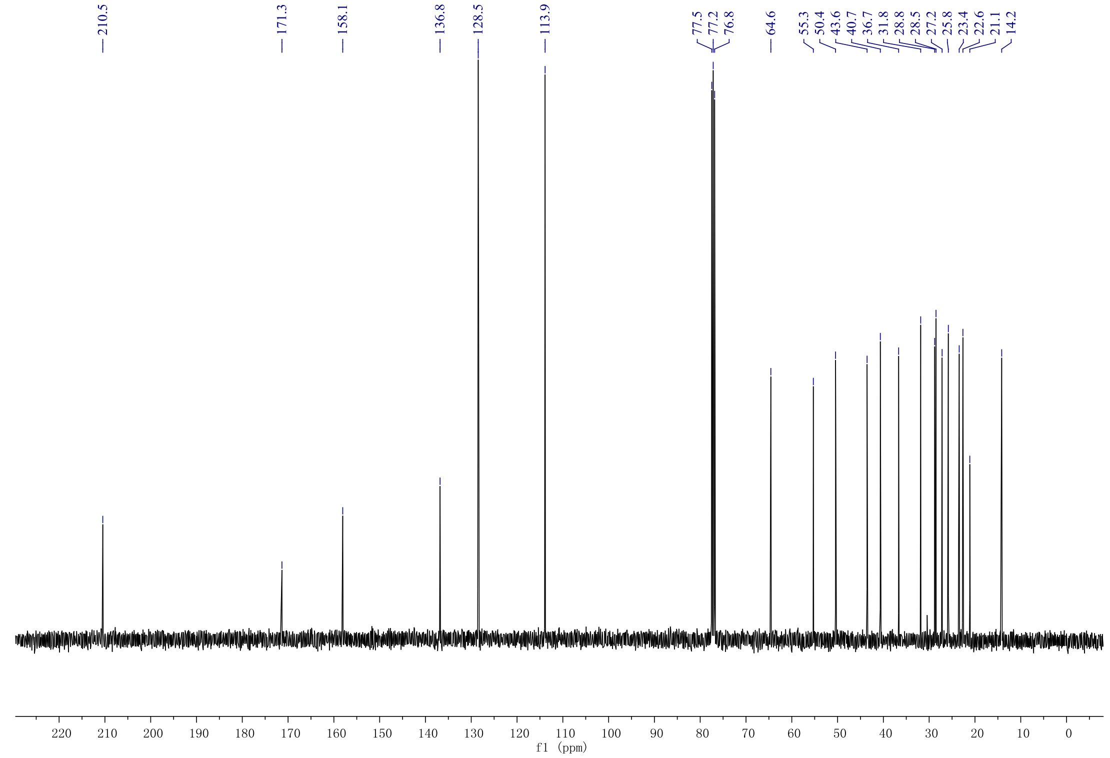

(*S*)-2-[8-(4-Methoxyphenyl)-6-oxotridecyl]isoindoline-1,3-dione (4y)

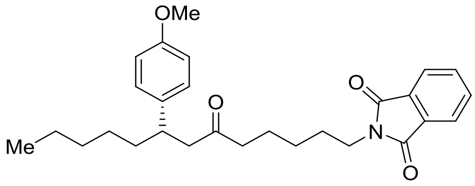

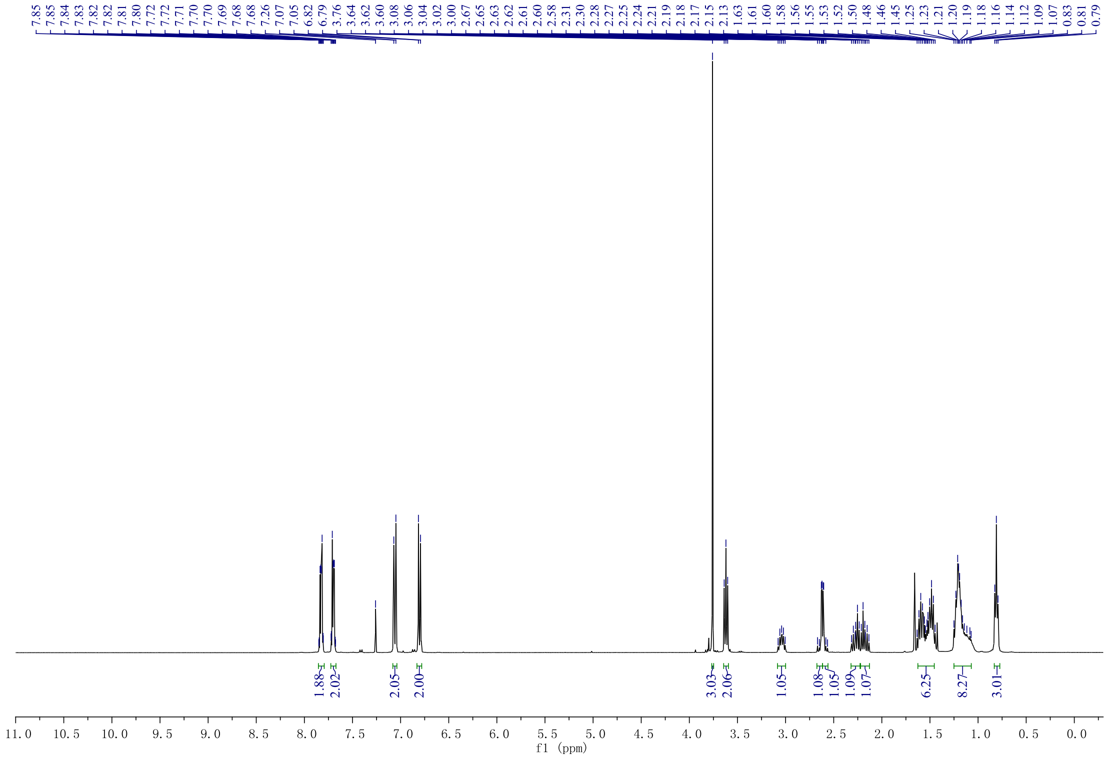

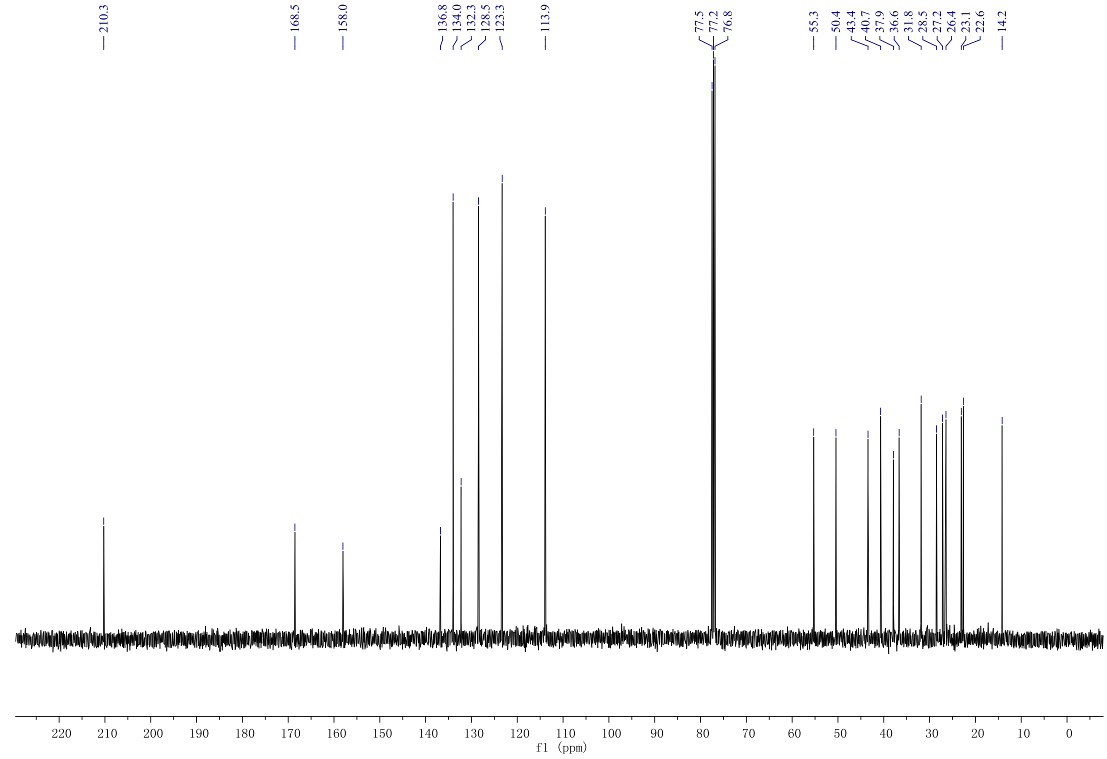

(*R*)-7-(4-Methoxyphenyl)-8-methyl-1-phenylnonan-5-one (4aa)

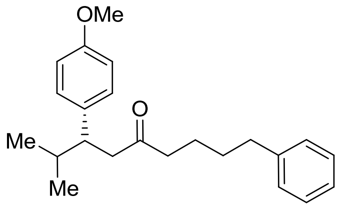

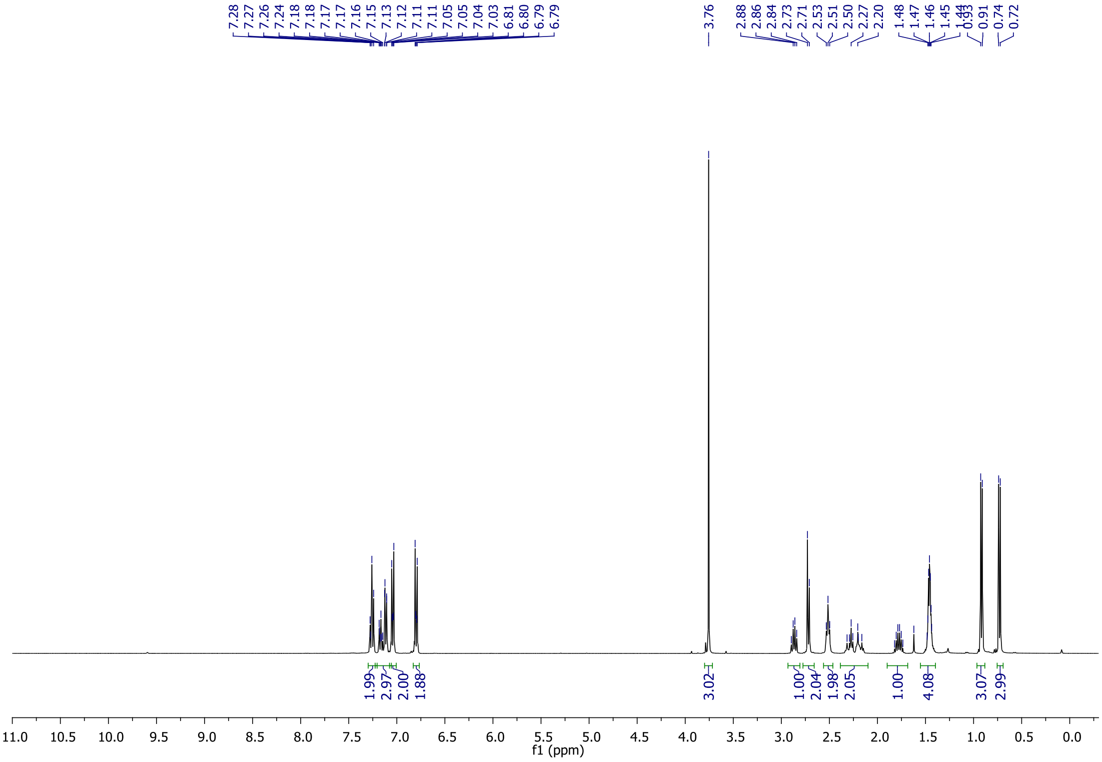

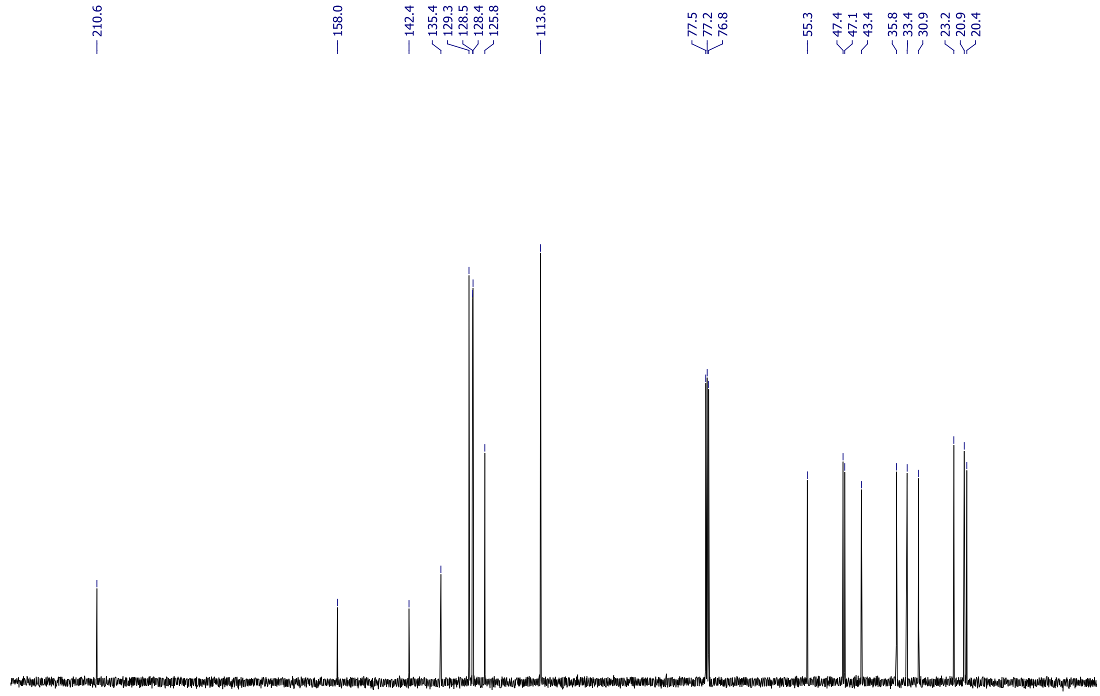

(*S*)-9-(4-Methoxyphenyl)-11,11-dimethyldodecane-2,7-dione (4ab)

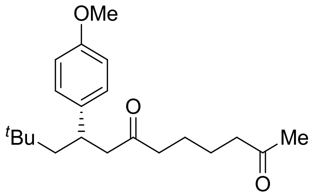

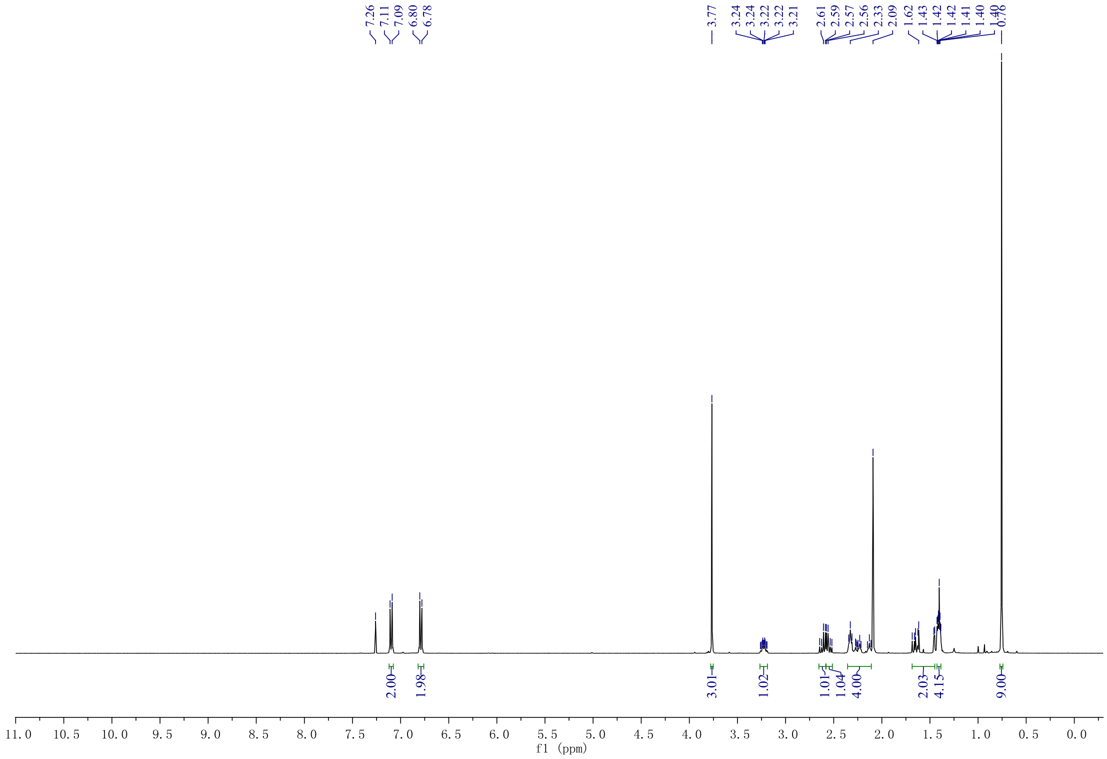

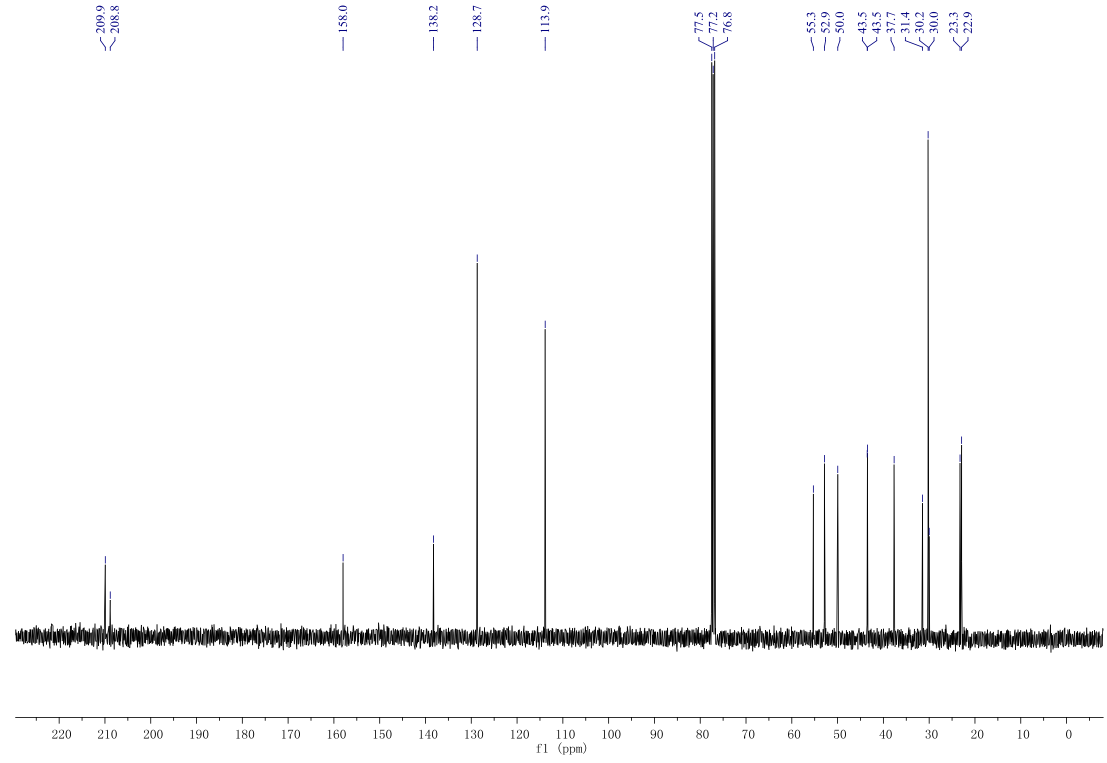

(*R*)-1-Cyclohexyl-1-(4-methoxyphenyl)-7-phenylheptan-3-one (4ac)

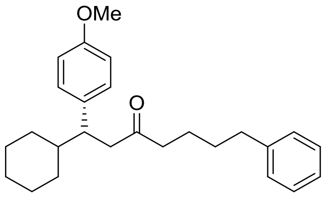

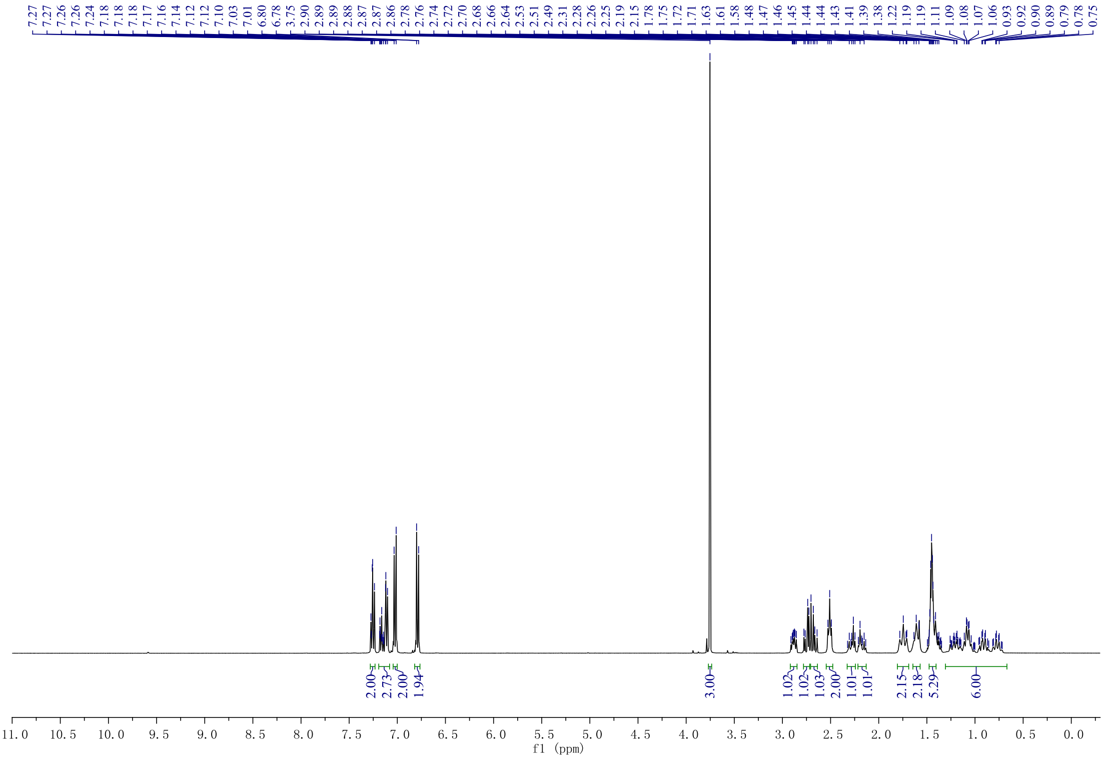

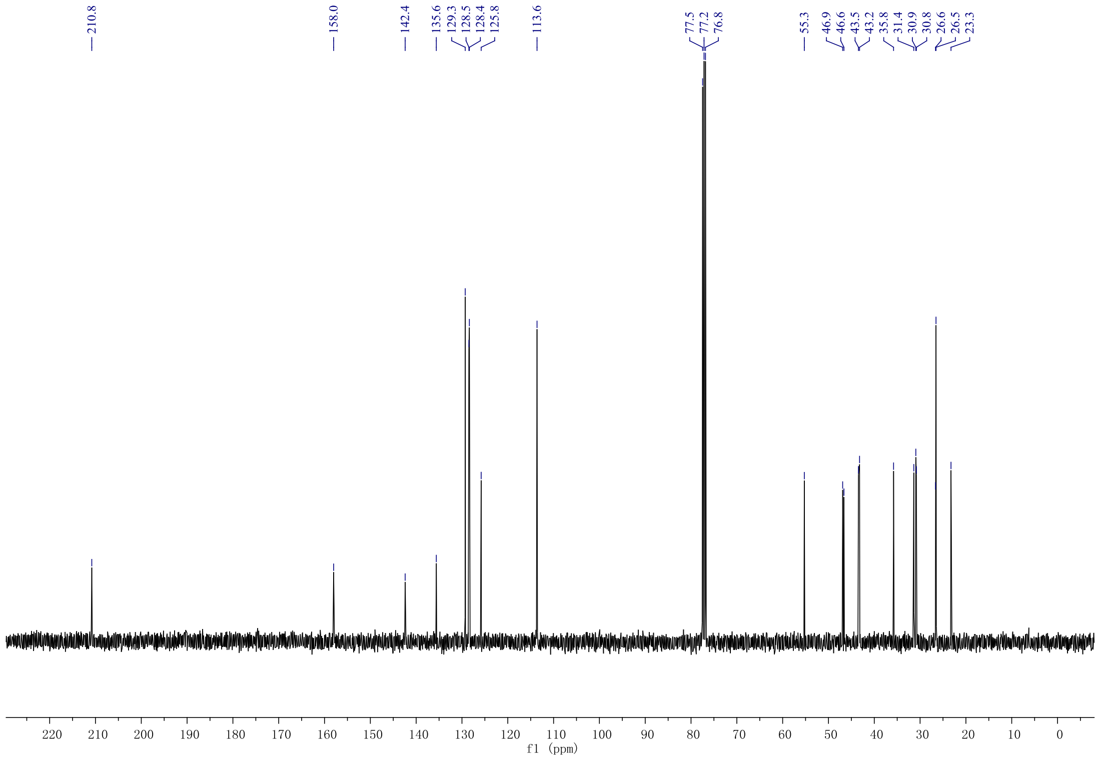

(*R*)-1-Cyclopropyl-1-(4-methoxyphenyl)-7-phenylheptan-3-one (4ad)

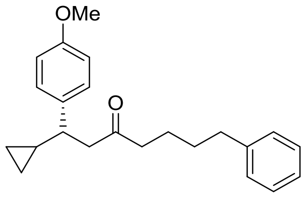

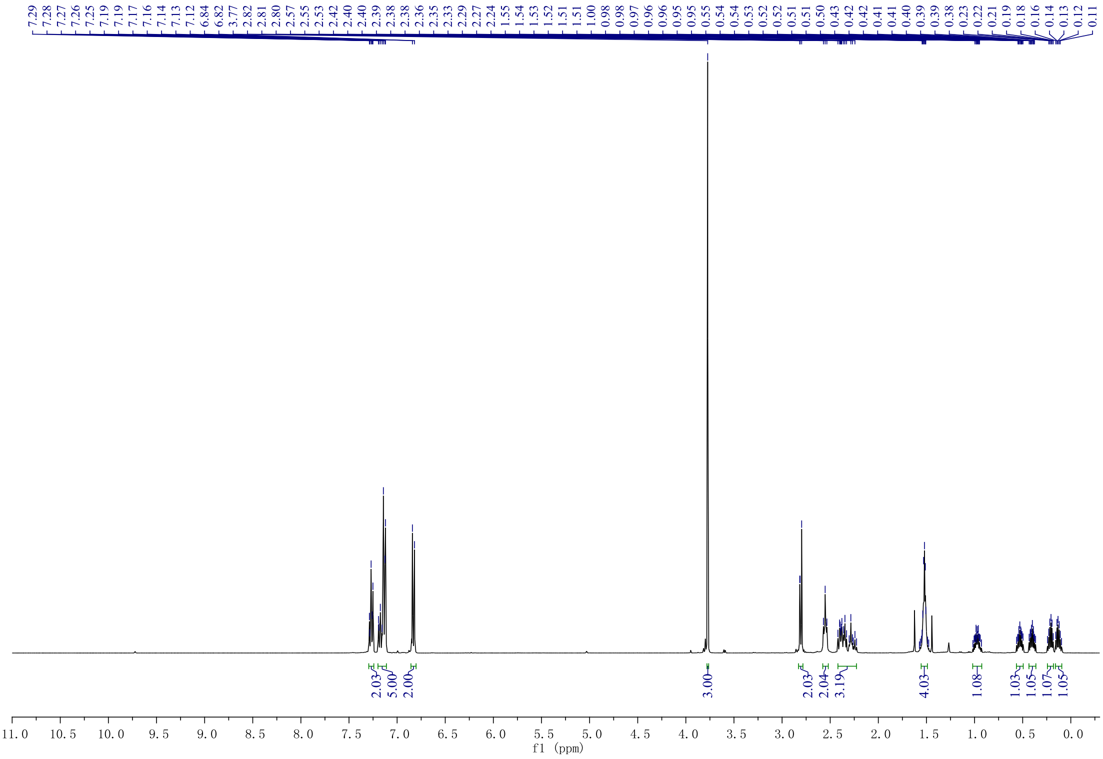

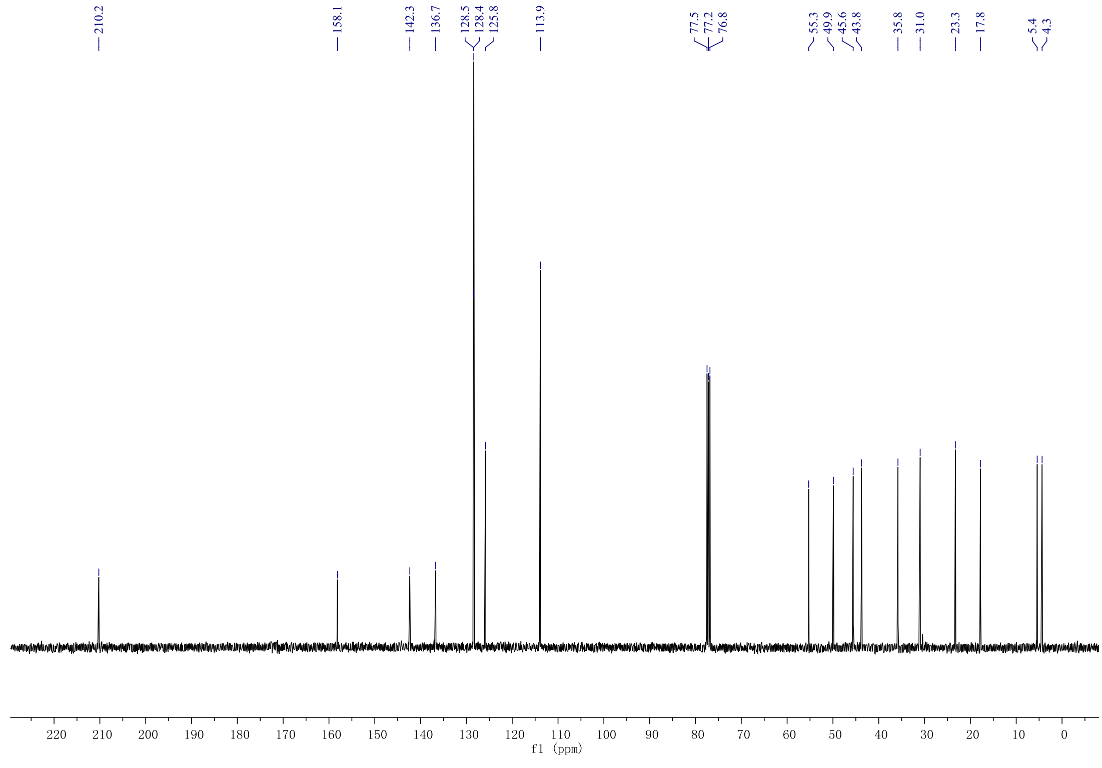

(*S*)-3-(4-Methoxyphenyl)-1,9-diphenylnonan-5-one (4ae)

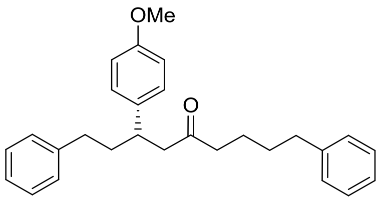

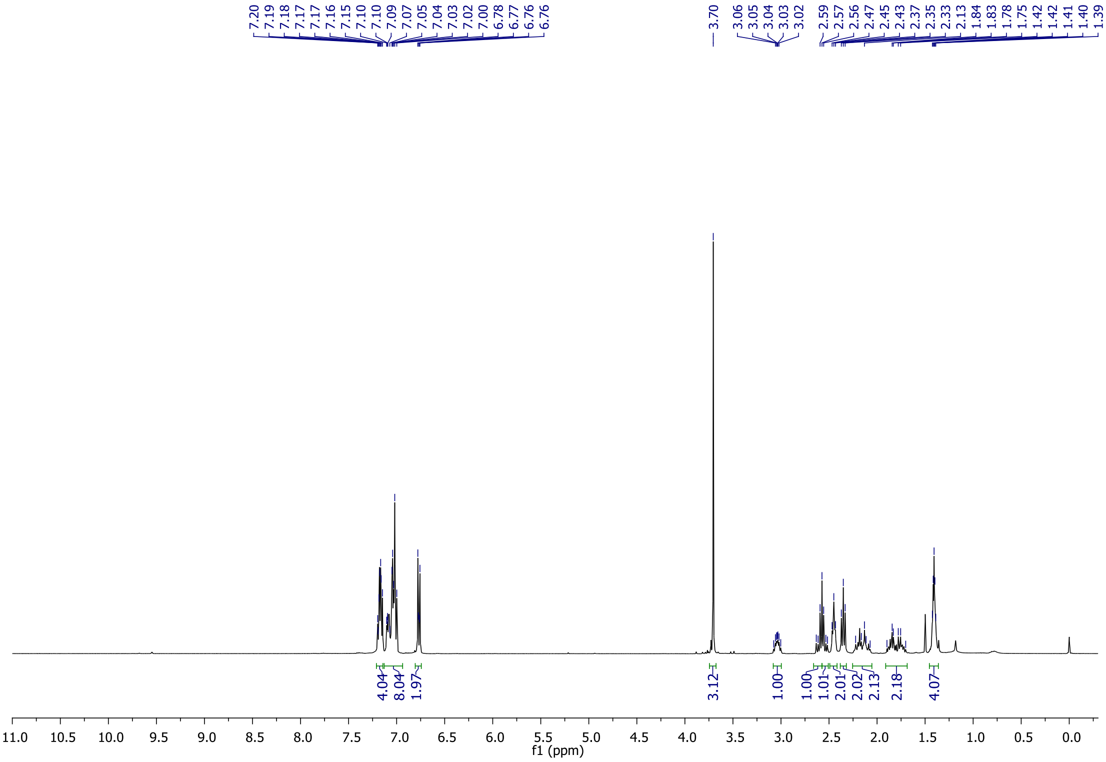

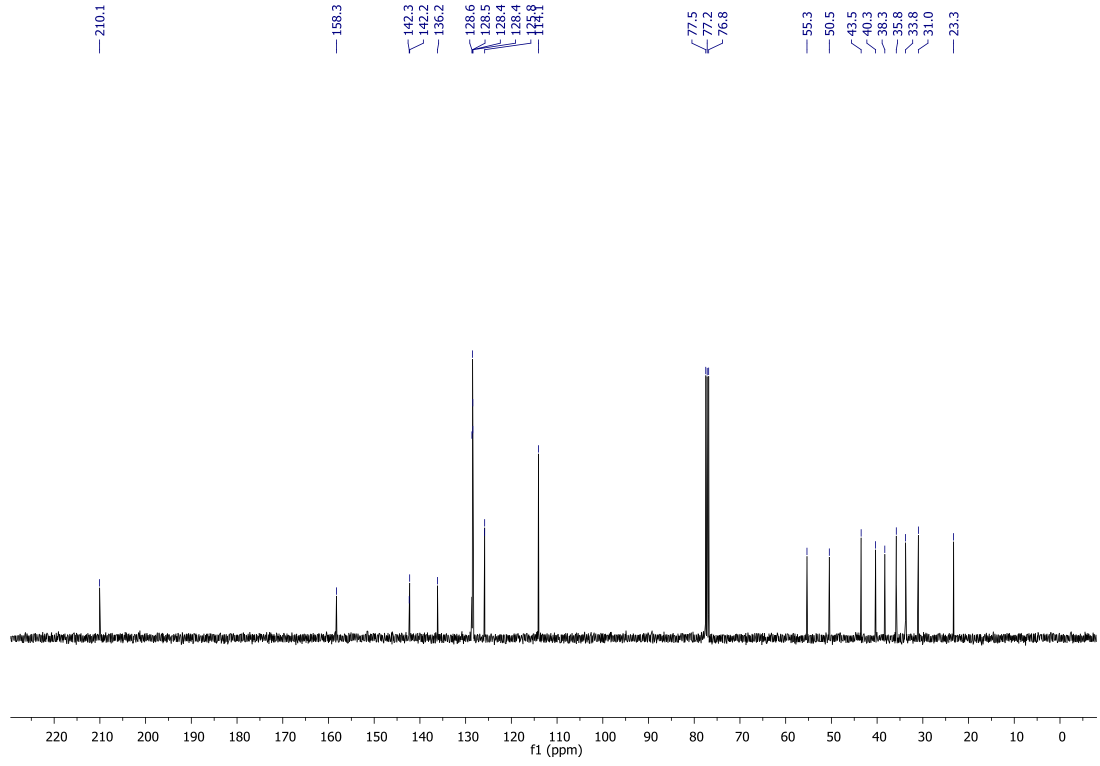

(*R*)-1-(4-Methoxyphenyl)-1,7-diphenylheptan-3-one (4af)

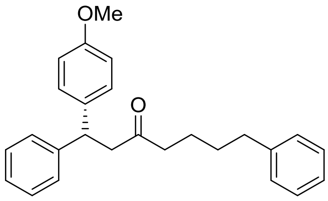

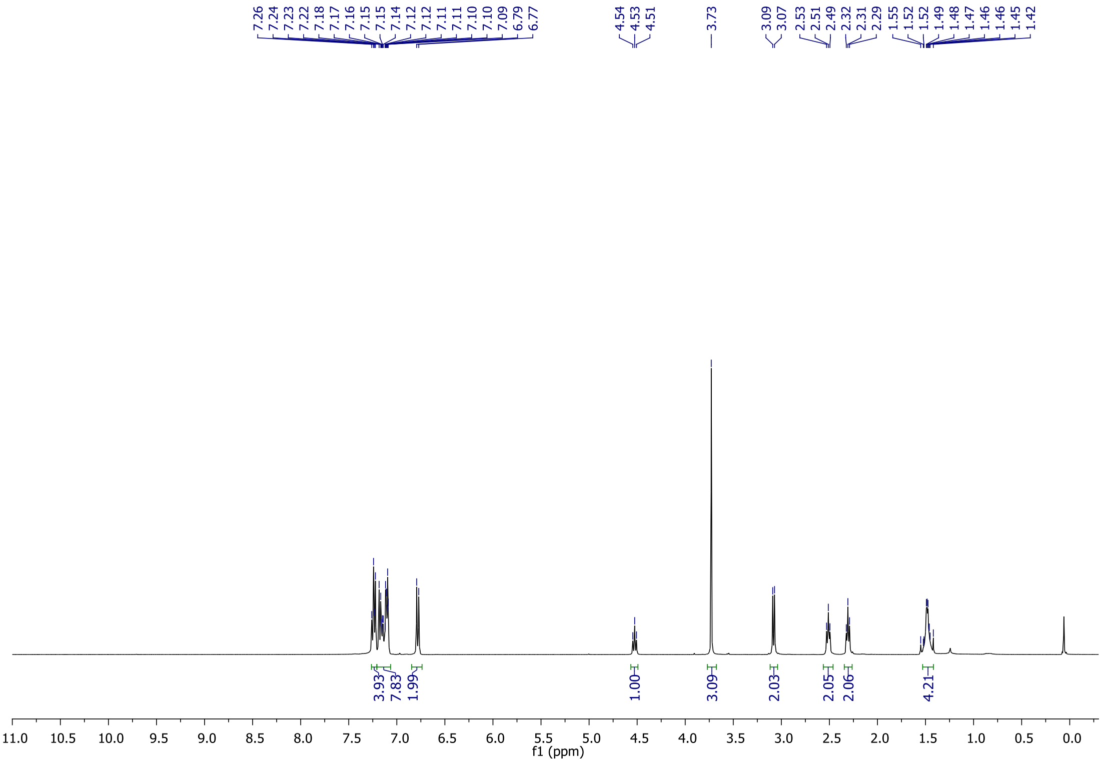

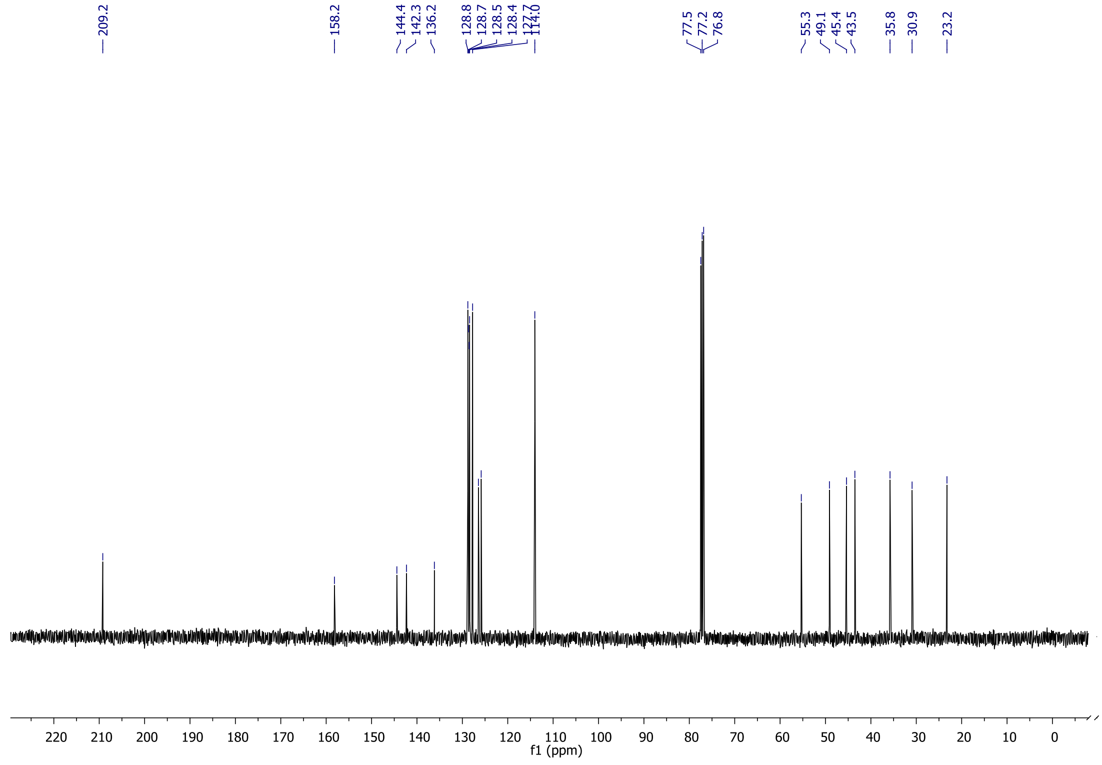

(*S*)-1-(4-Bromophenyl)-1-(4-methoxyphenyl)-7-phenylheptan-3-one (4ag)

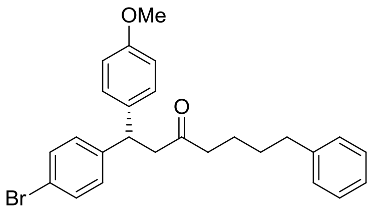

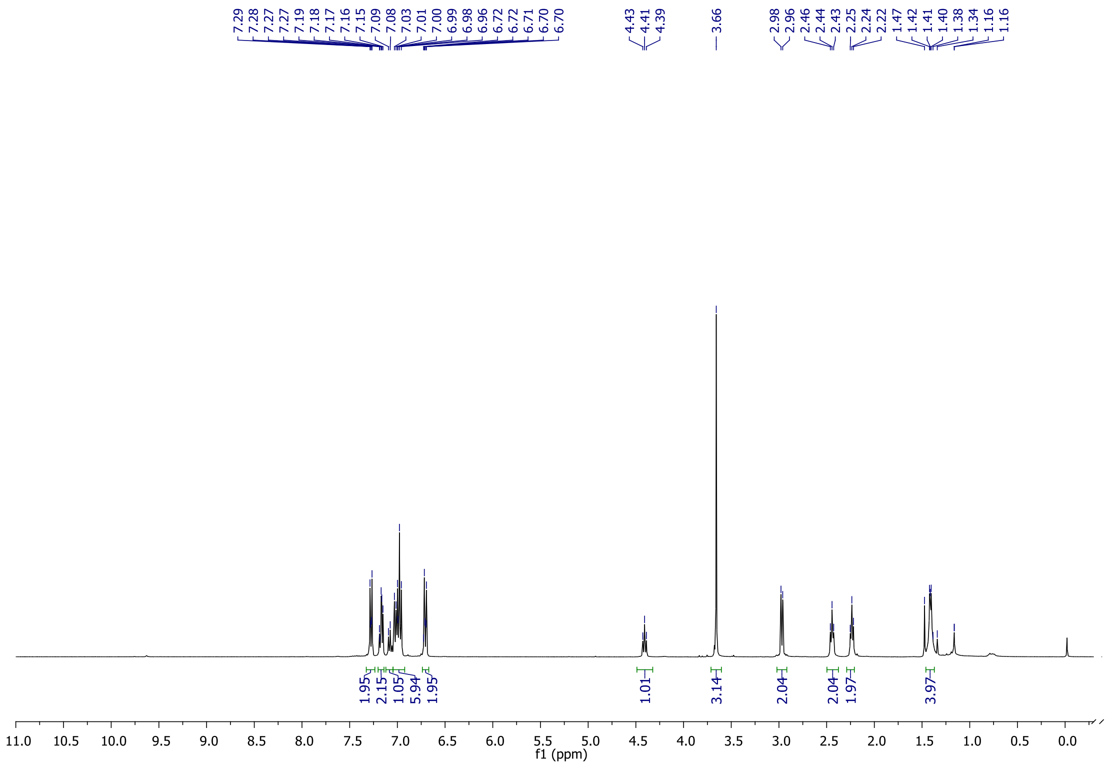

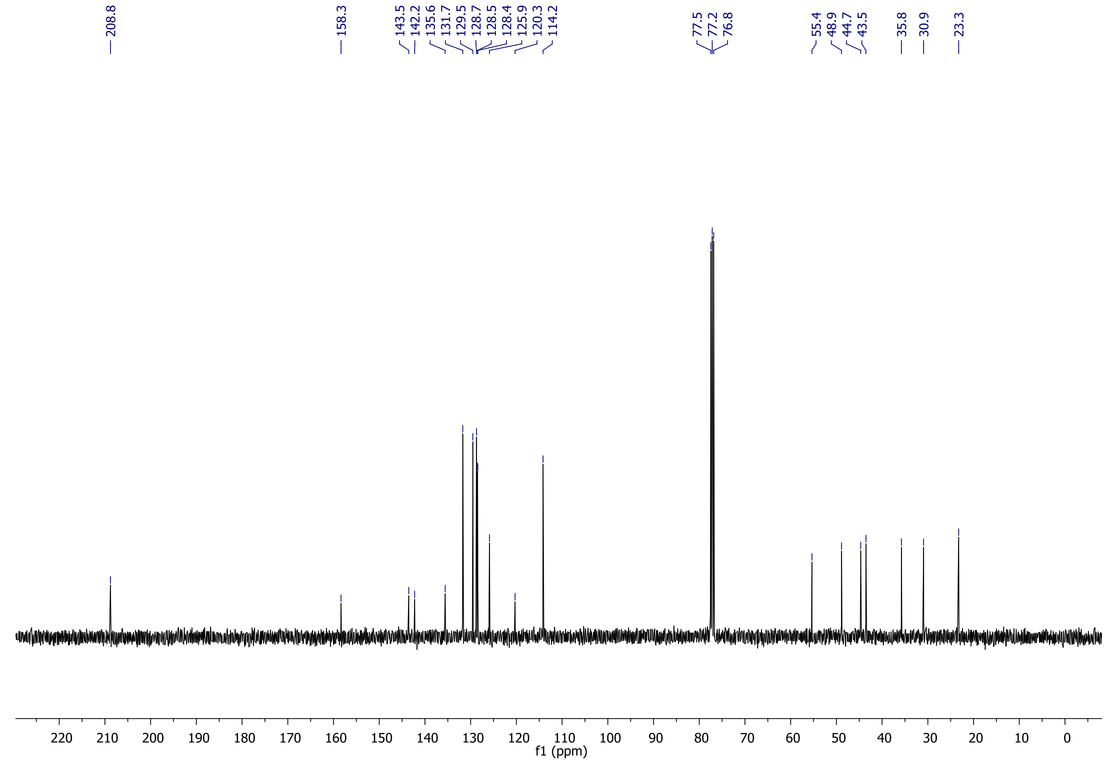

(*R*)-1-(4-Methoxyphenyl)-1-(4-nitrophenyl)-7-phenylheptan-3-one (4ah)

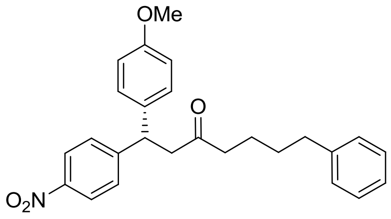

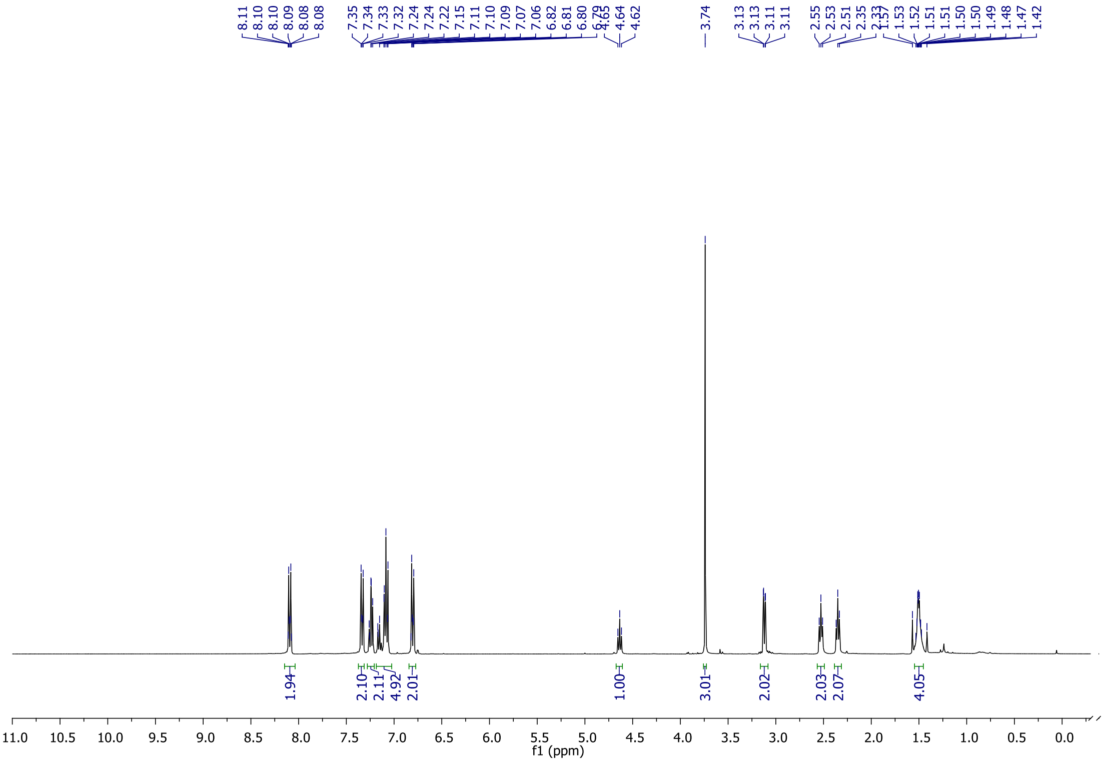

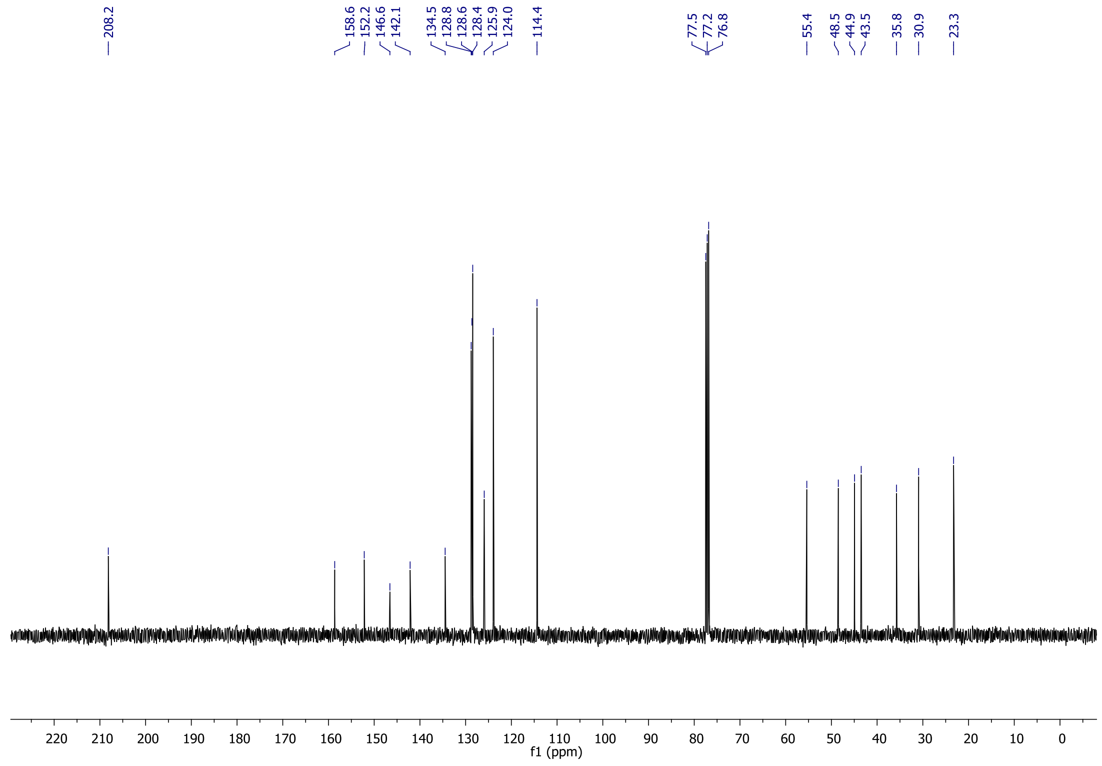

(*S*)-1-(Benzyloxy)-3-(4-methoxyphenyl)-9-phenylnonan-5-one (4ai)

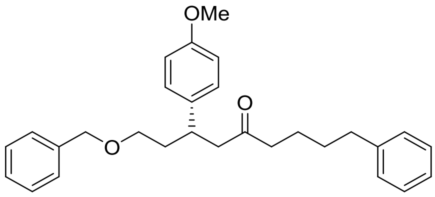

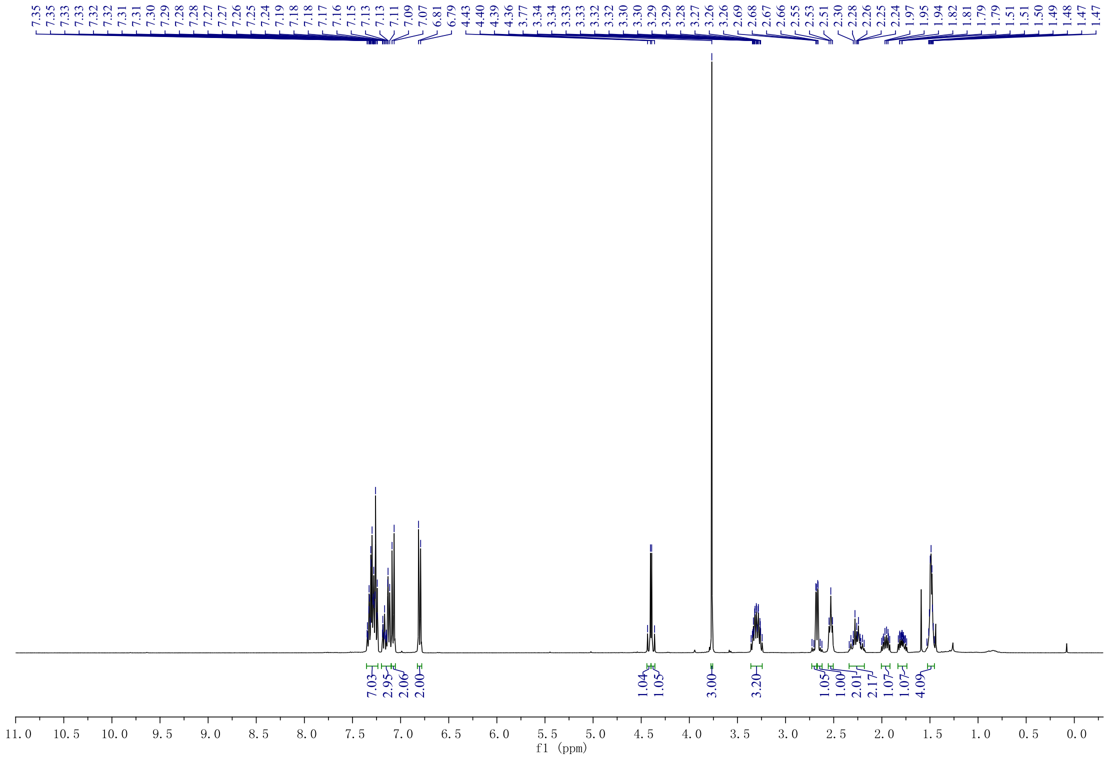

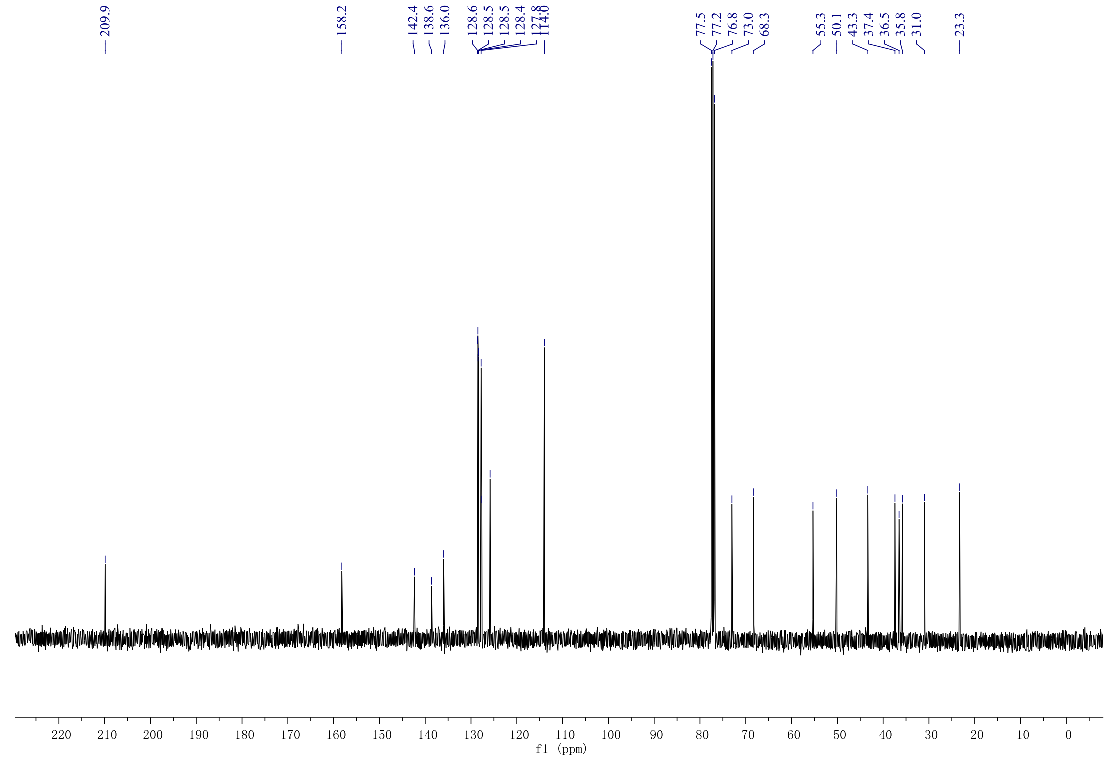

(*S*)-5-(4-Methoxyphenyl)-7-oxo-11-phenylundecyl acetate (4aj)

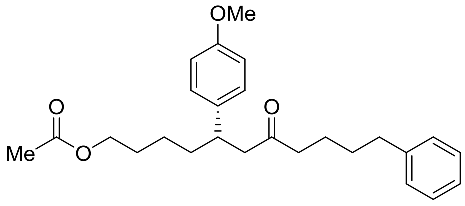

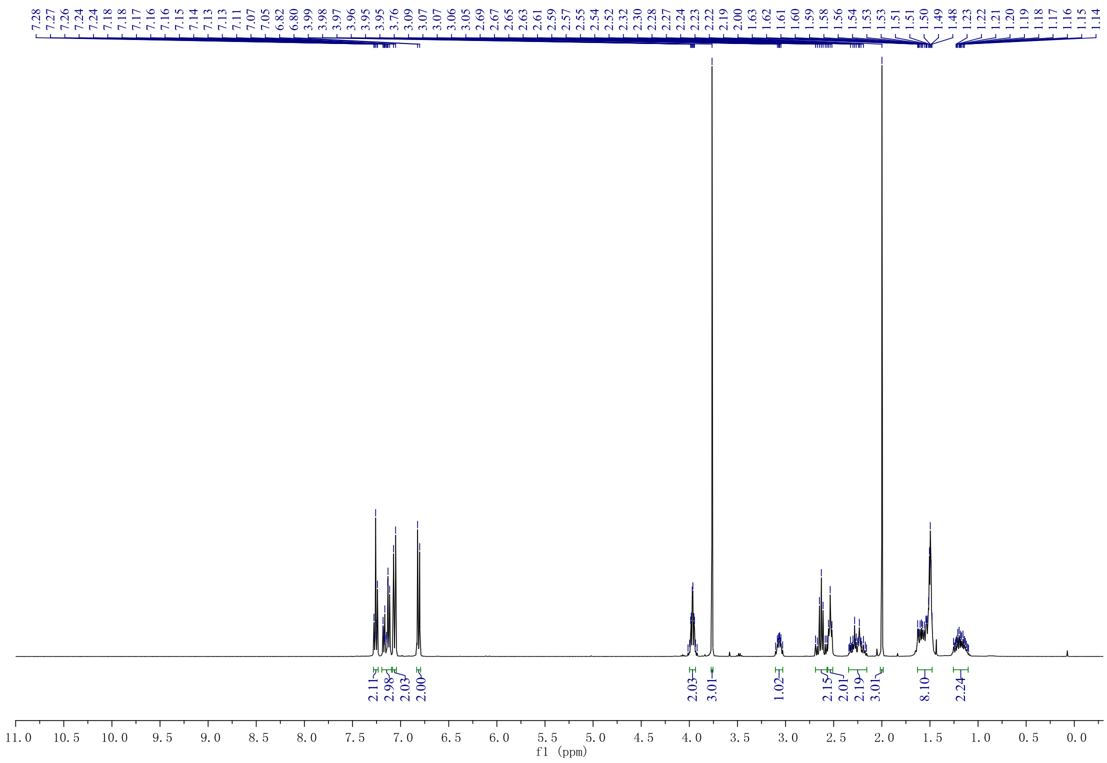

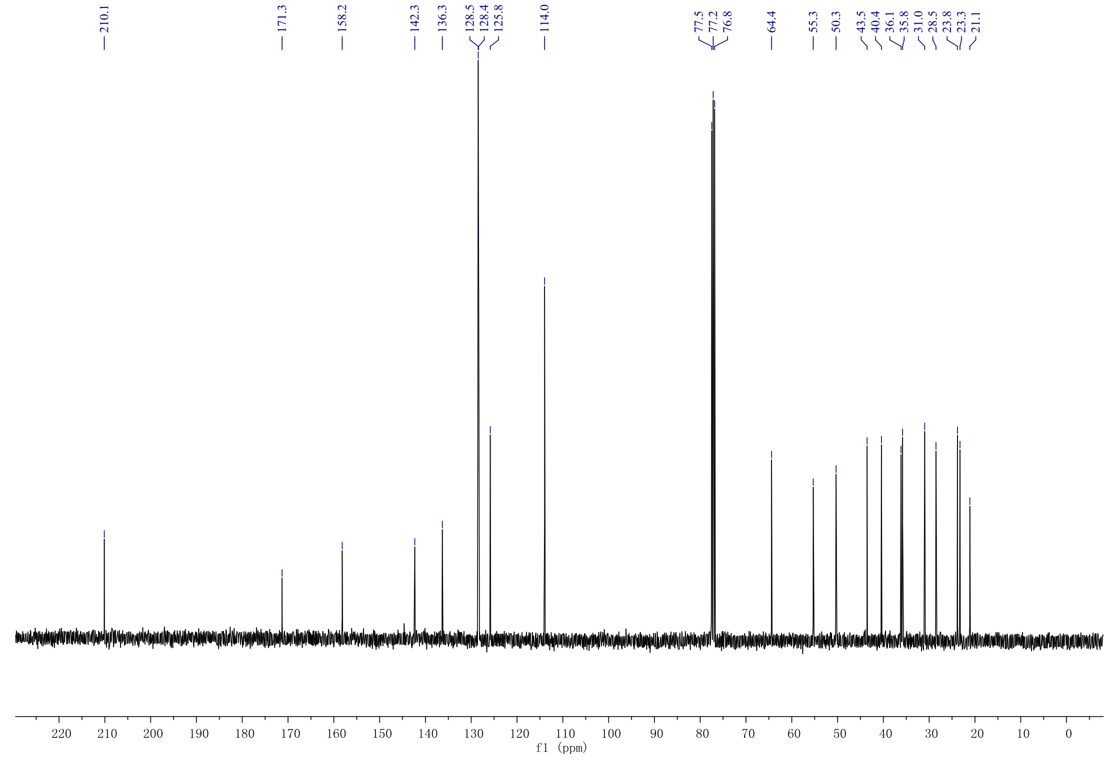

(*S*)-2-[4-(4-Methoxyphenyl)-6-oxo-10-phenyldecyl]isoindoline-1,3-dione (4ak)

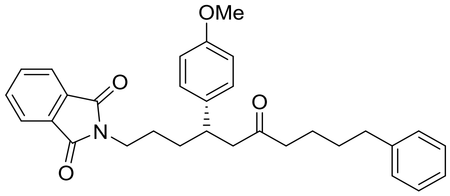

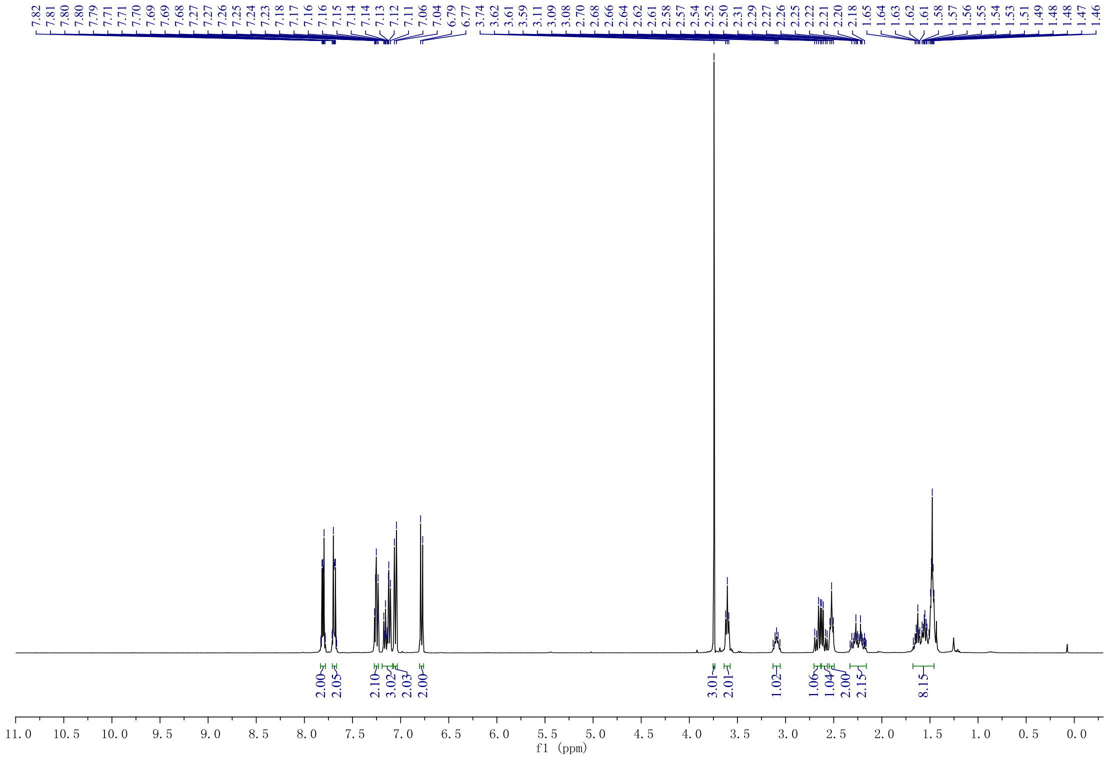

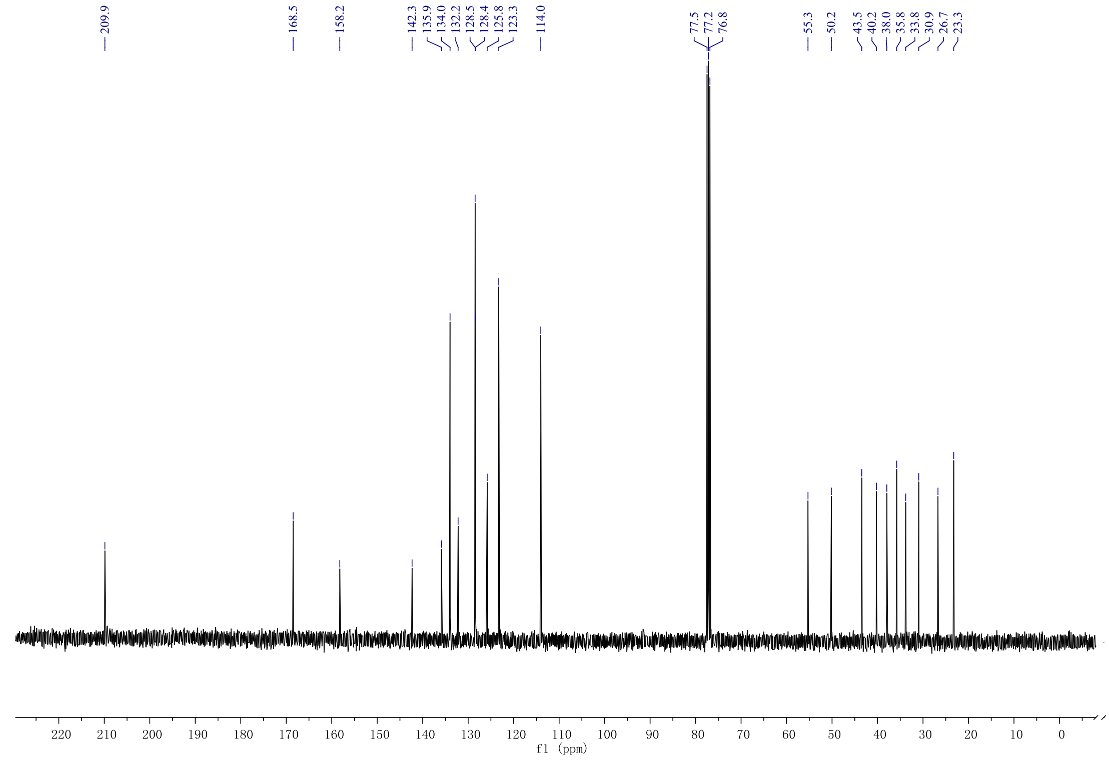

Methyl 4-[(7*S*,9*S*)-9,13-dimethyl-5-oxo-1-phenyltetradec-12-en-7-yl]benzoate (4al)

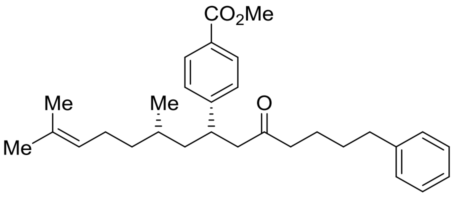

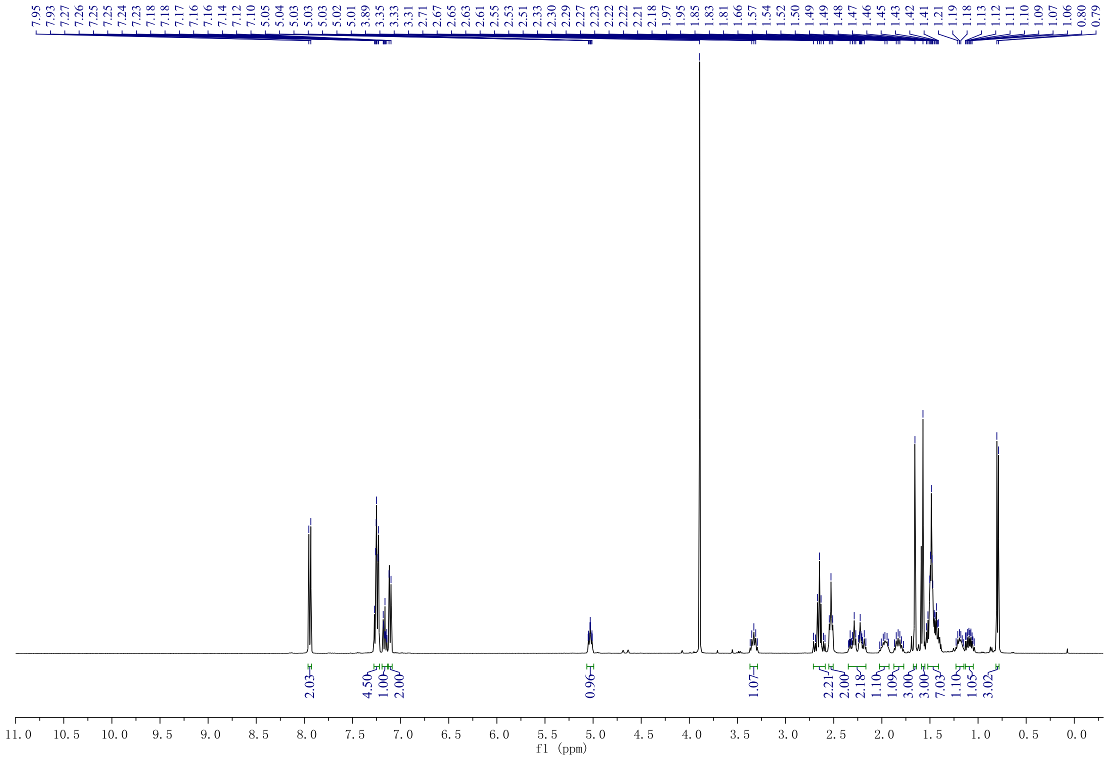

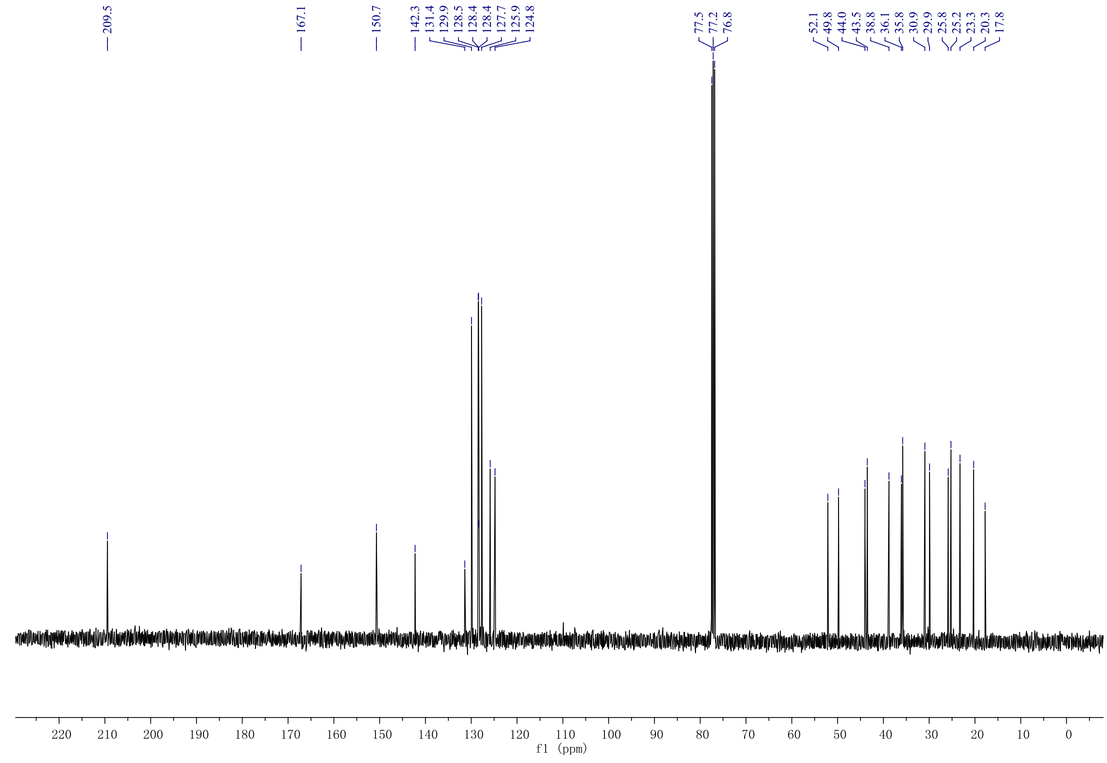

Methyl 4-[(7*R*,9*S*)-9,13-dimethyl-5-oxo-1-phenyltetradec-12-en-7-yl]benzoate (4am)

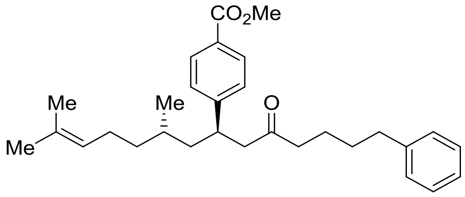

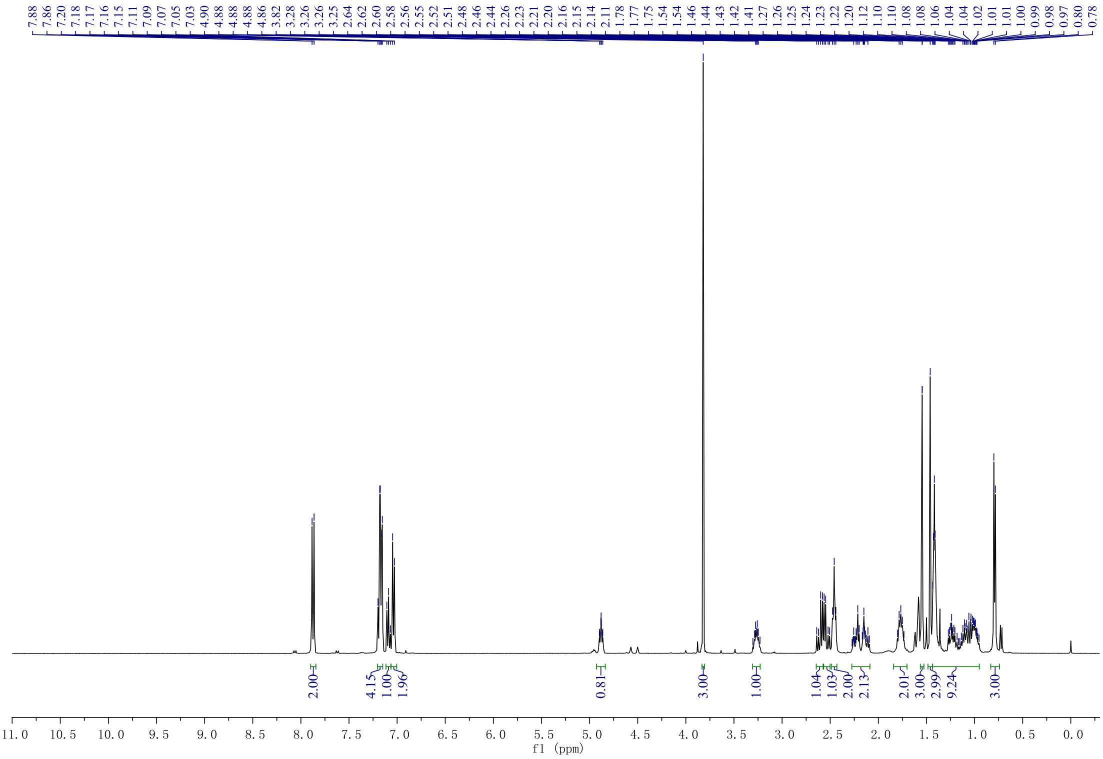

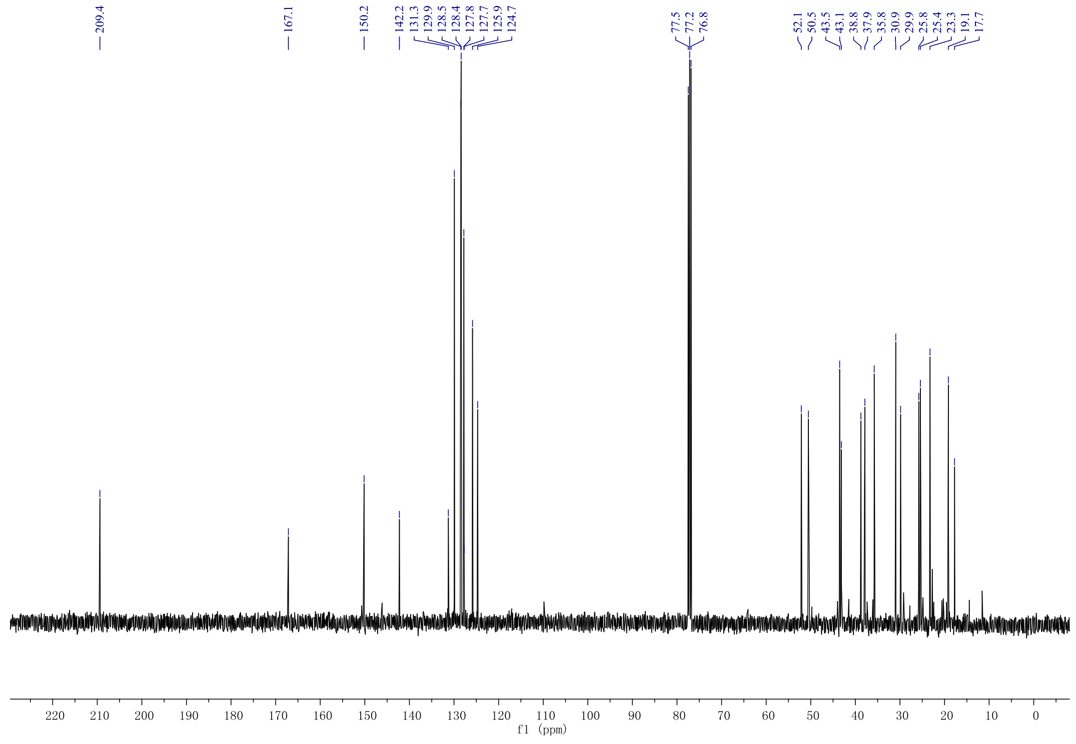

(*S*,*E*)-7-(4-Methoxyphenyl)-2,2-dimethyldodec-3-en-5-one (5a)

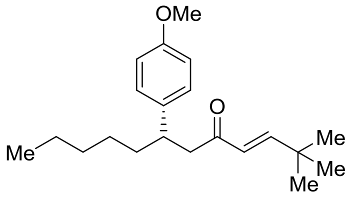

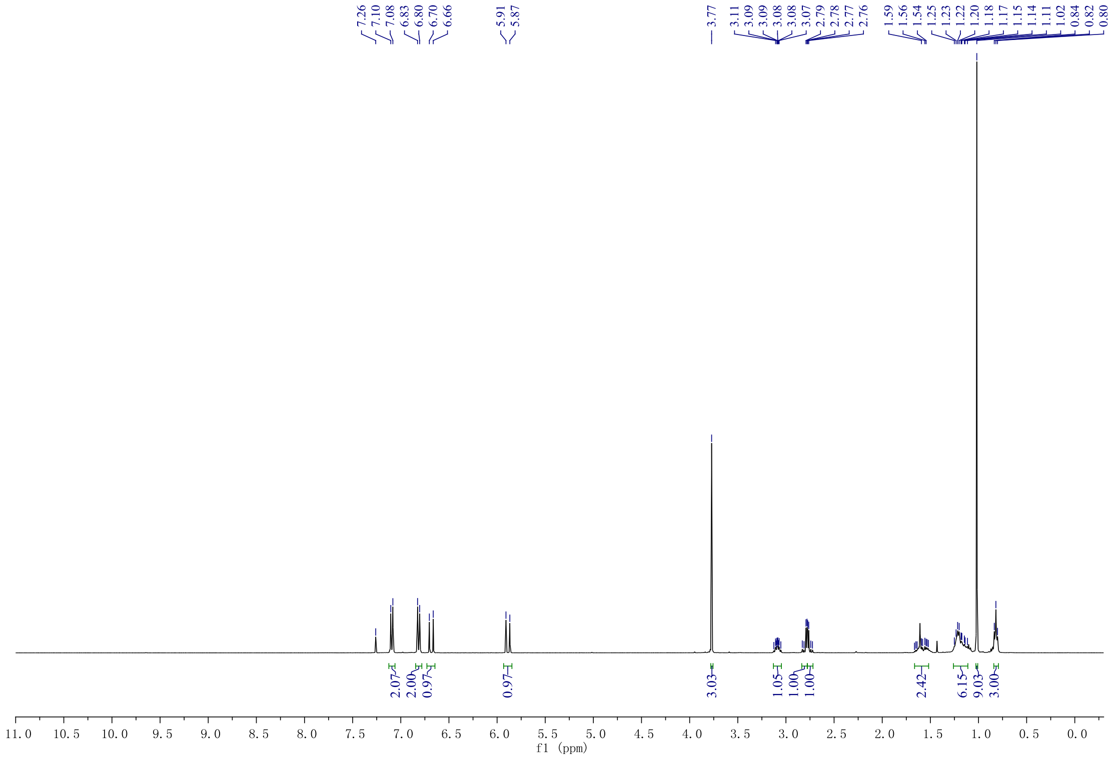

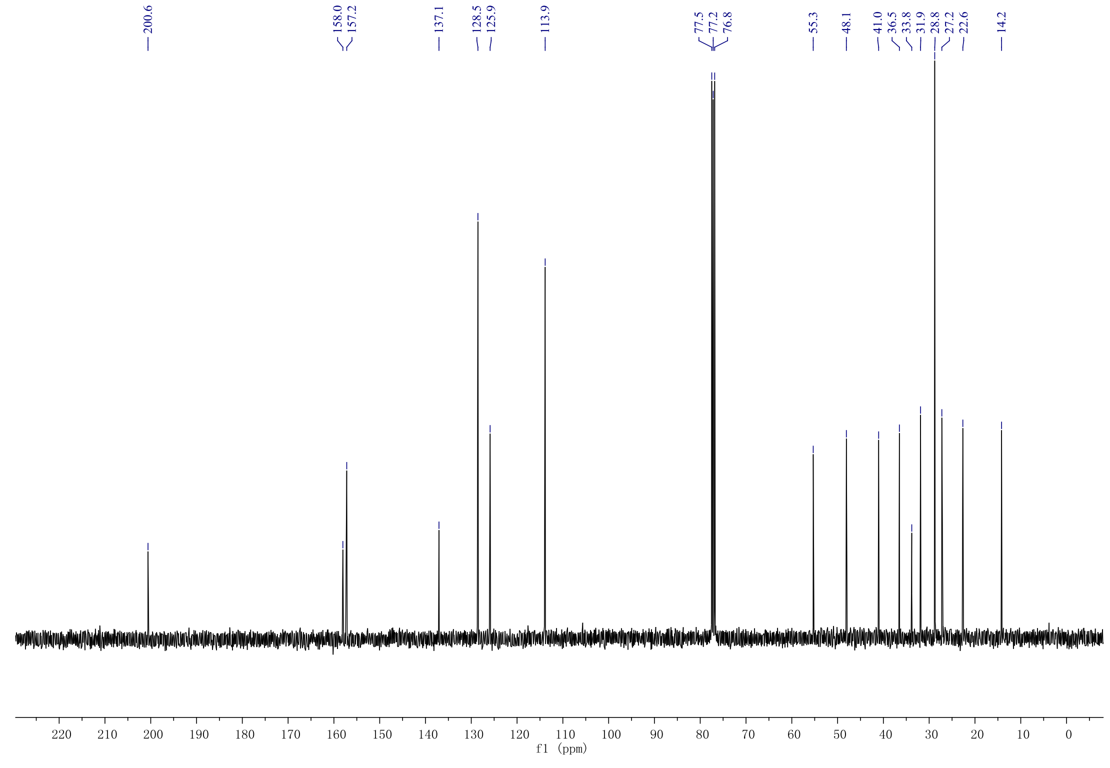

(*S*,*E*)-8-(4-Methoxyphenyl)-5-propyltridec-4-en-6-one (5b)

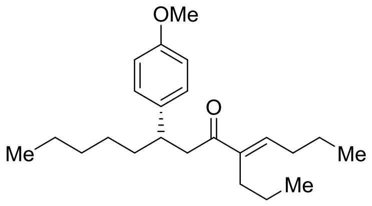

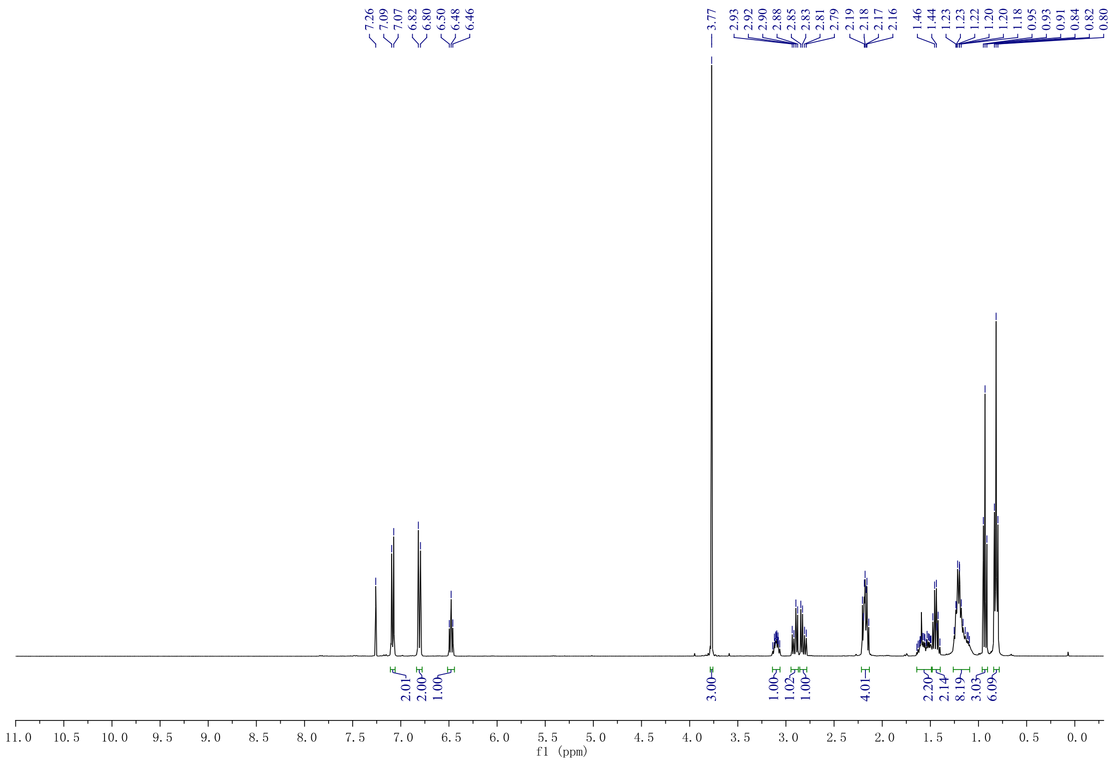

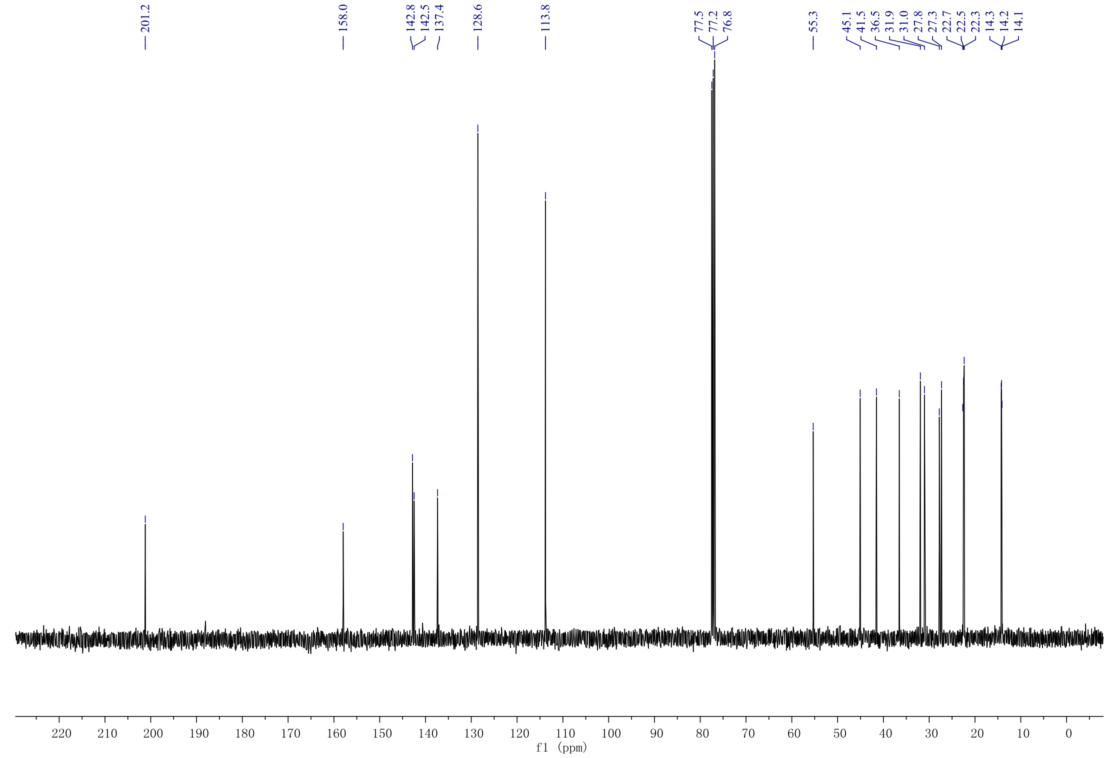

Dimethyl 4,4'-[(1*R*,5*S*)-1-cyclohexyl-3-oxodecane-1,5-diyl]dibenzoate (5c)

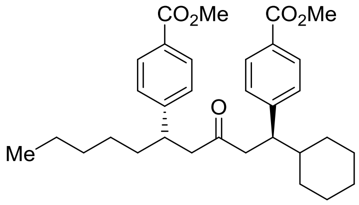

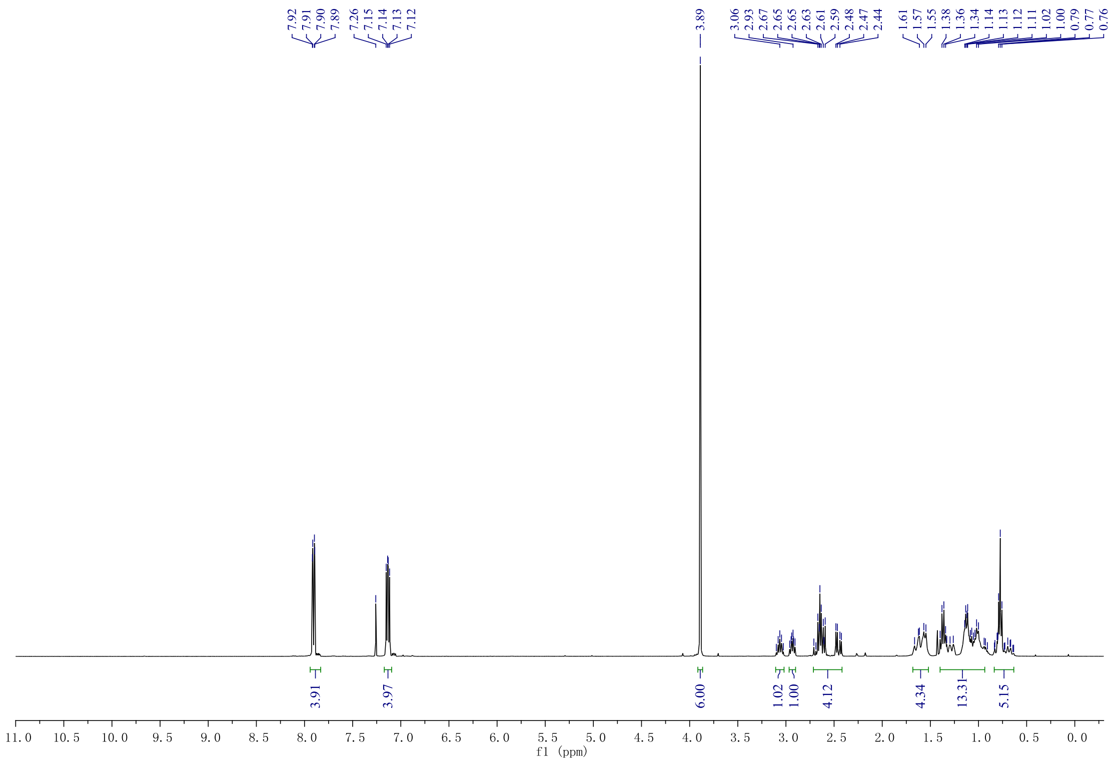

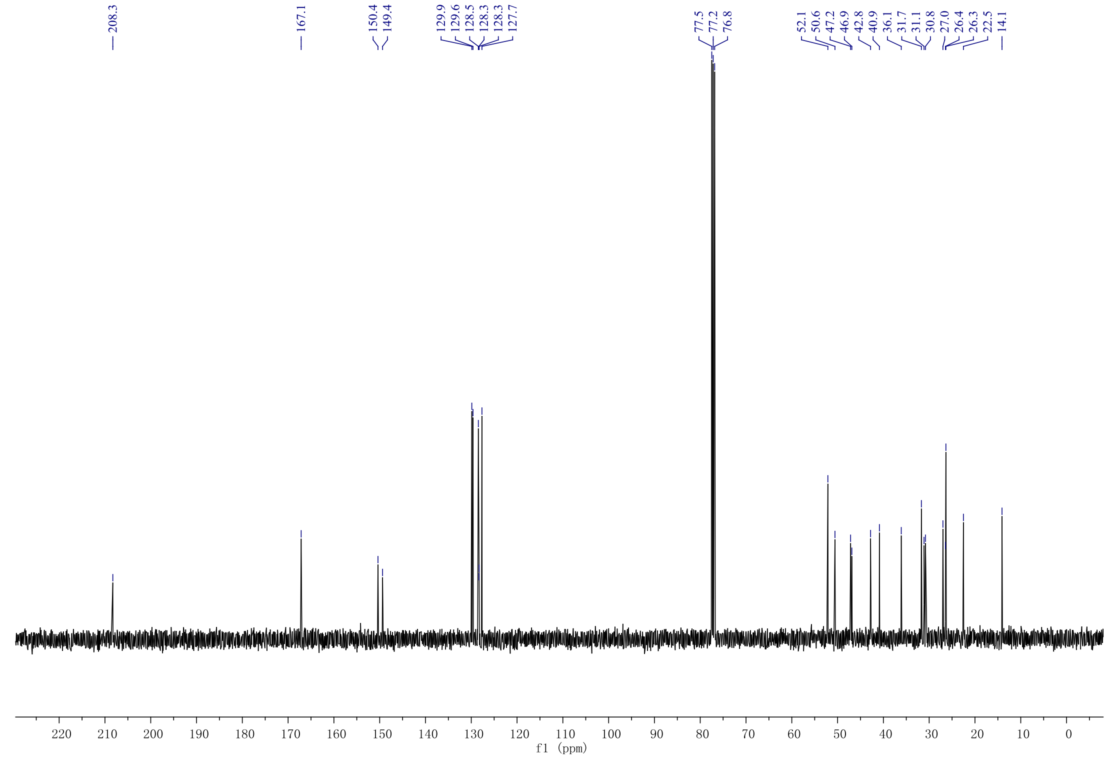

(*S*)-6-[2-(4-Methoxyphenyl)heptyl]-2,3,4,5-tetrahydropyridine (6a)

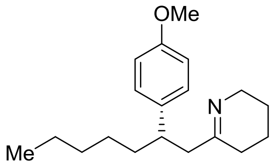

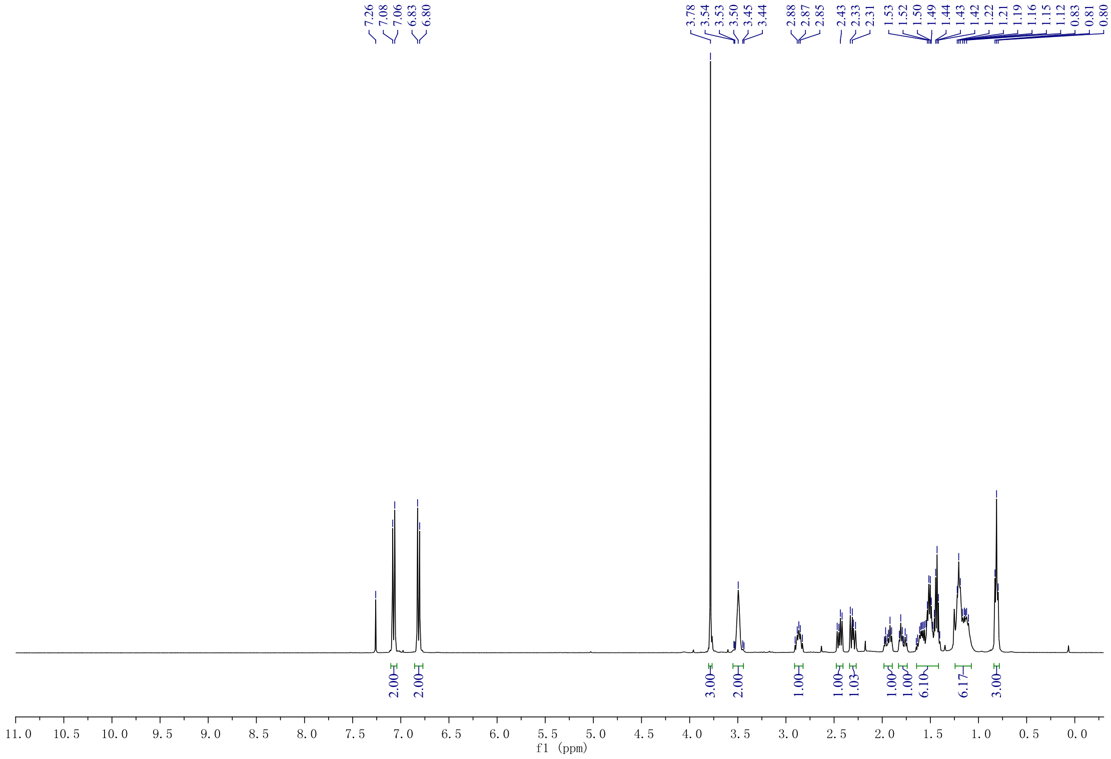

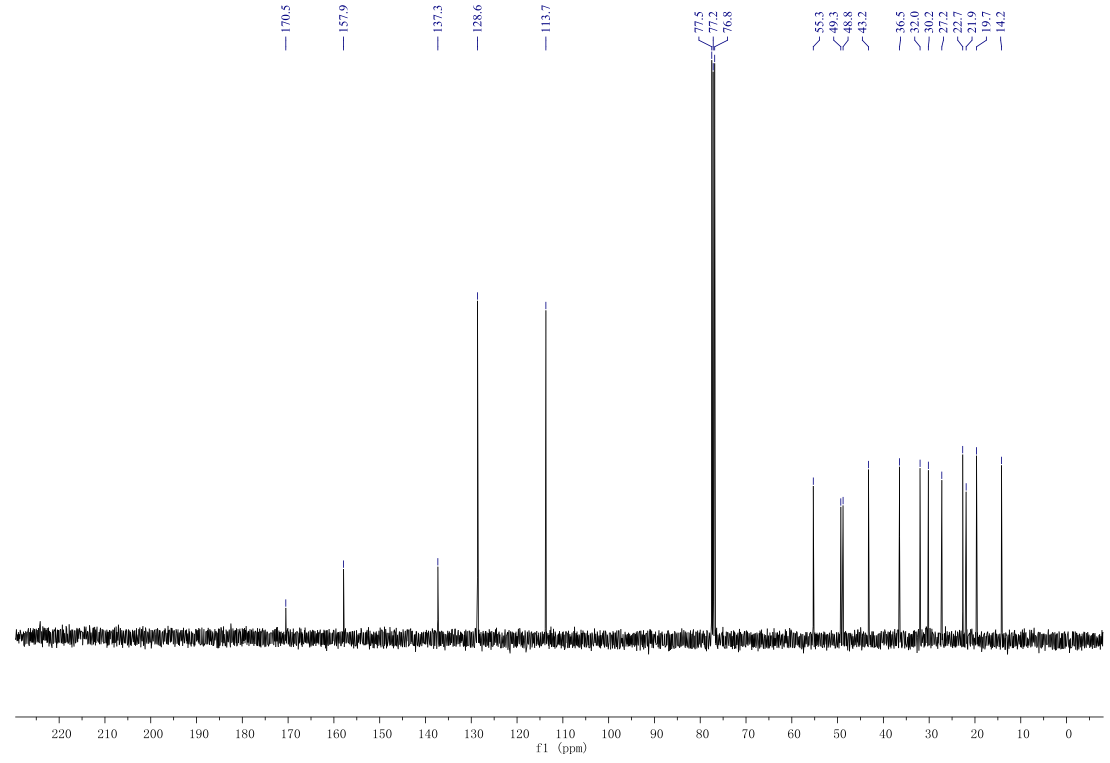

(*S*)-3-(4-Methoxyphenyl)-5-[(*S*)-2-(4-methoxyphenyl)heptyl]-3,4-dihydro-2*H*-pyrrole (6b)

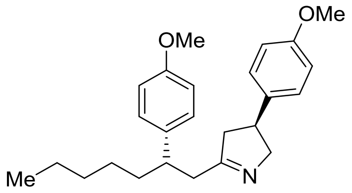

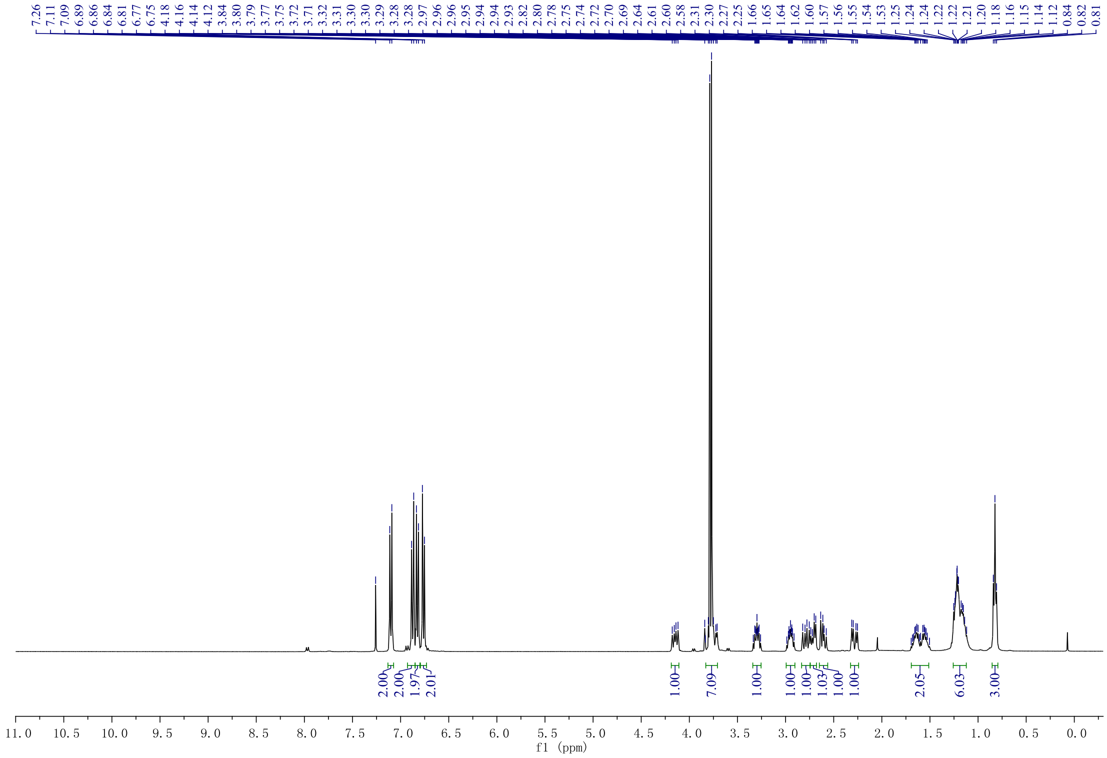

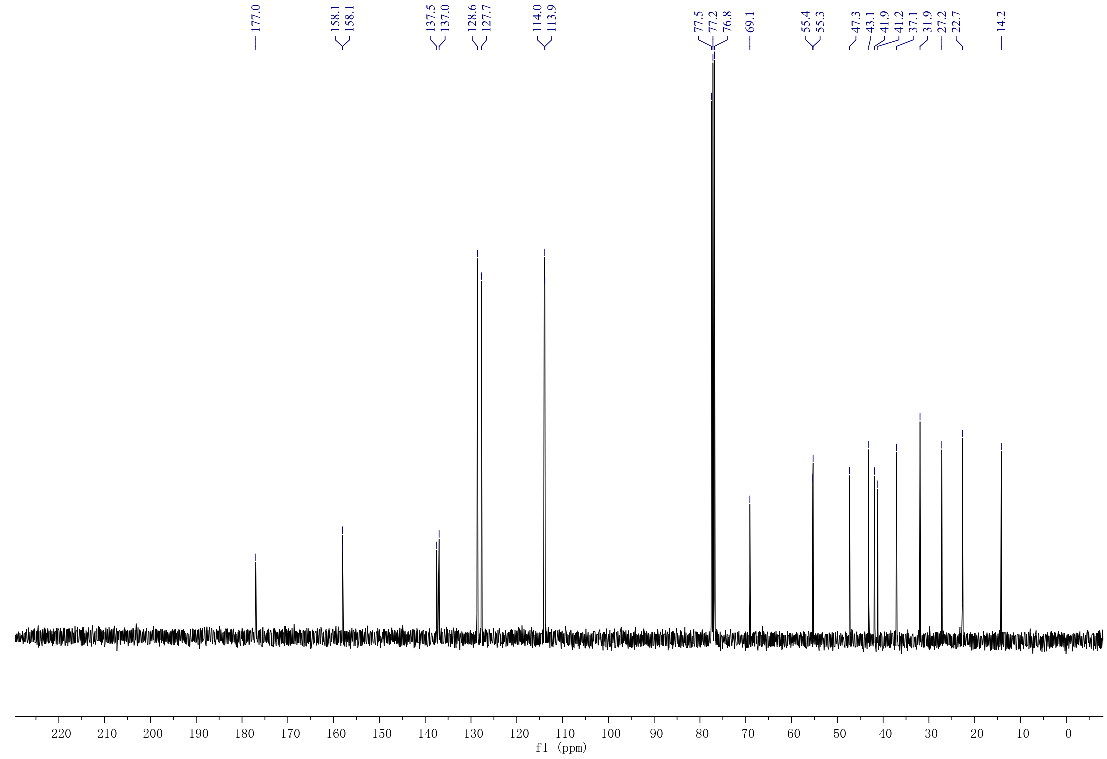

(4*S*)-2-Methoxy-4-pentyl-2-(4-phenylbutyl)chroman (6c)

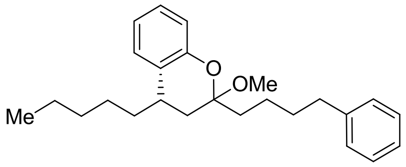

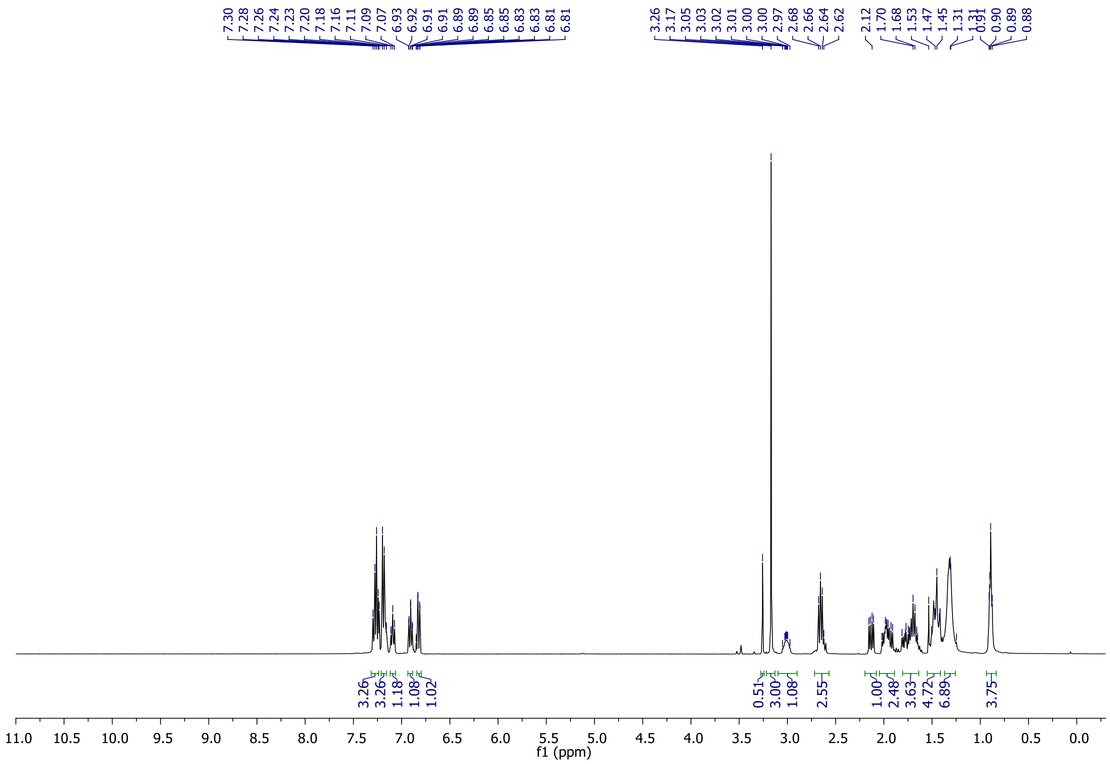

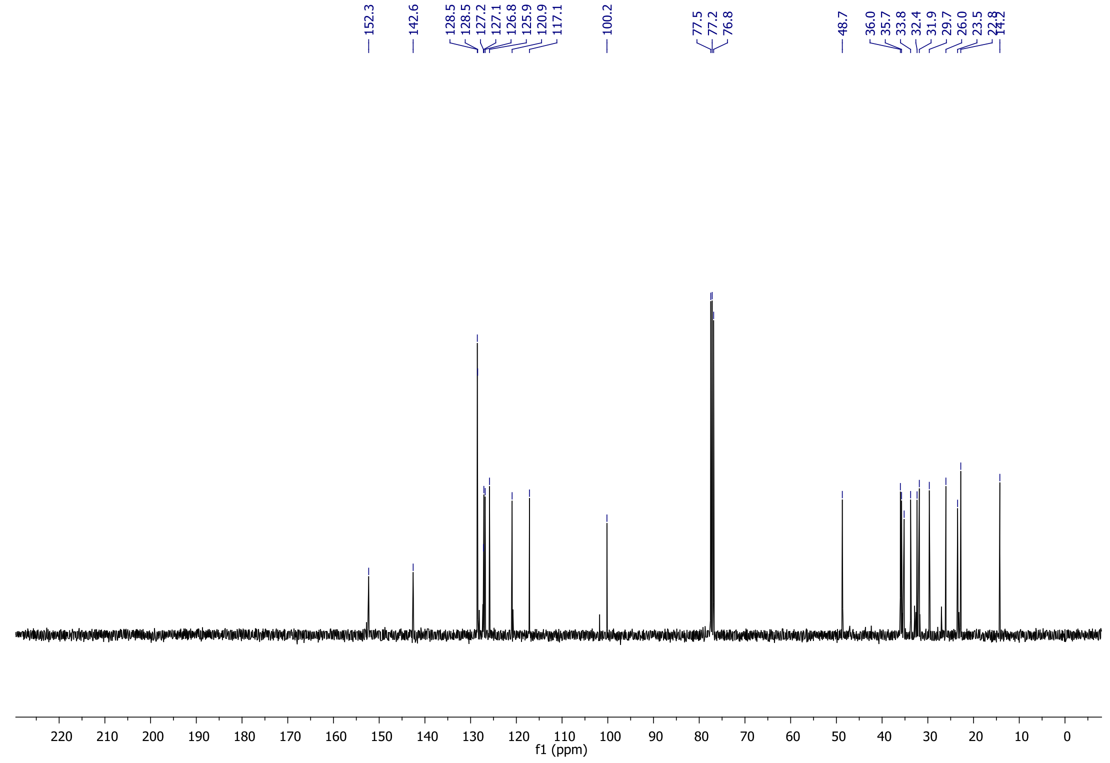

6-[(4*S*)-2-Methoxy-4-pentylchroman-2-yl]hexan-2-one (6d)

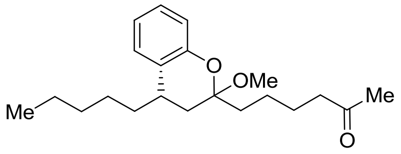

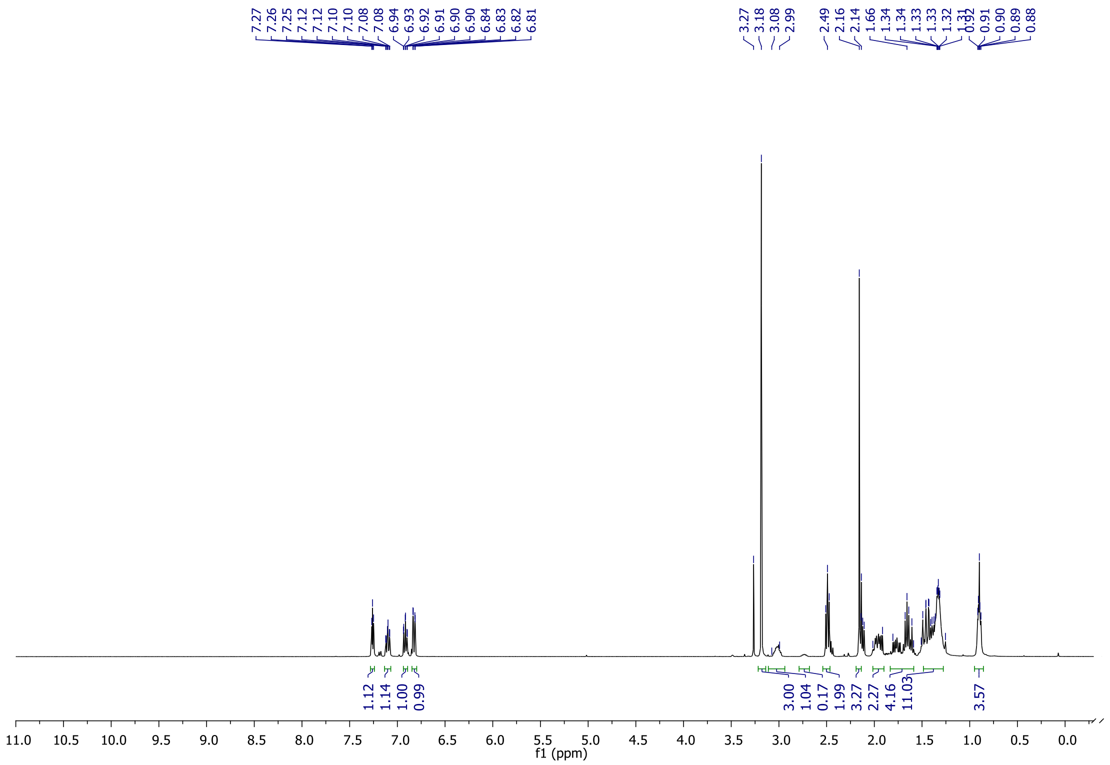

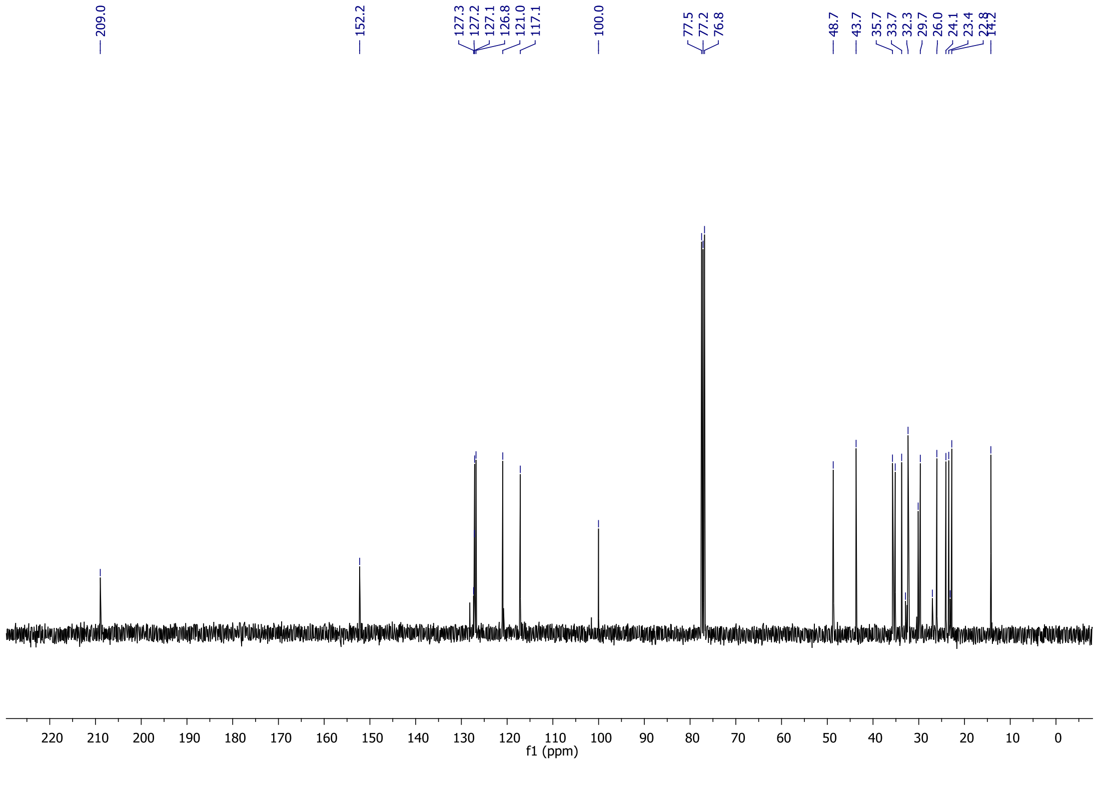

(2*R*,4*S*)-4-Pentyl-2-(4-phenylbutyl)chroman (6e)

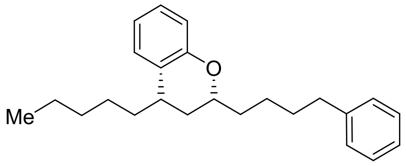

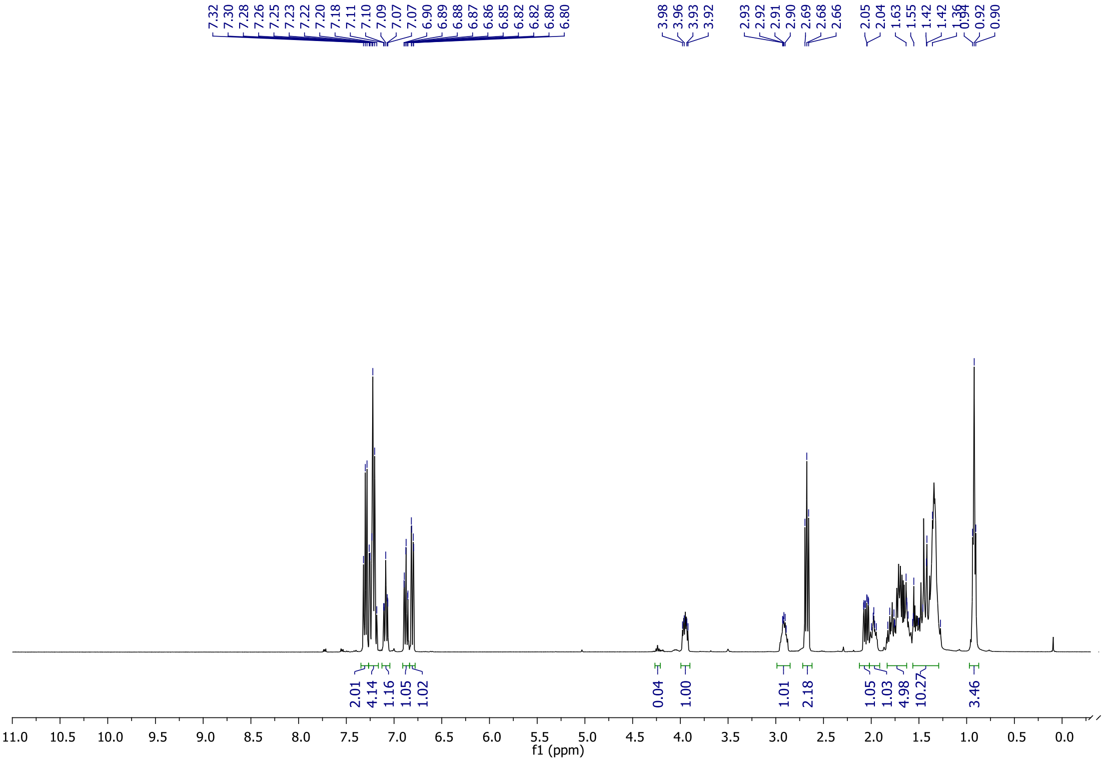

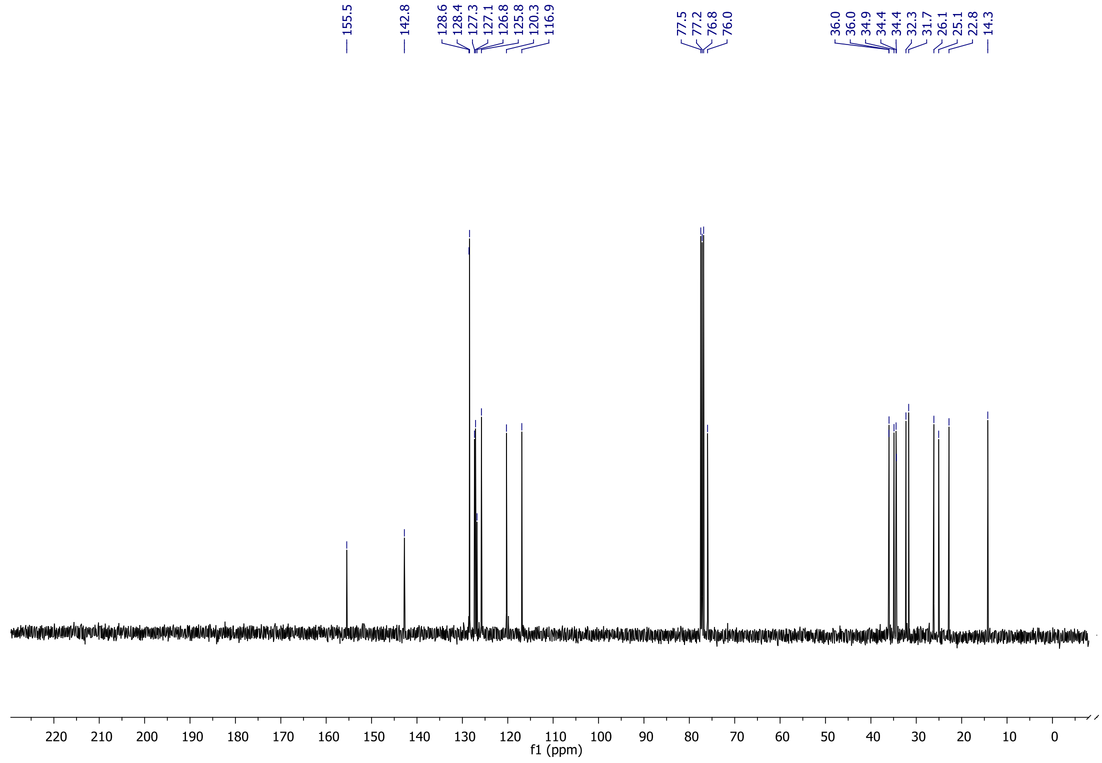

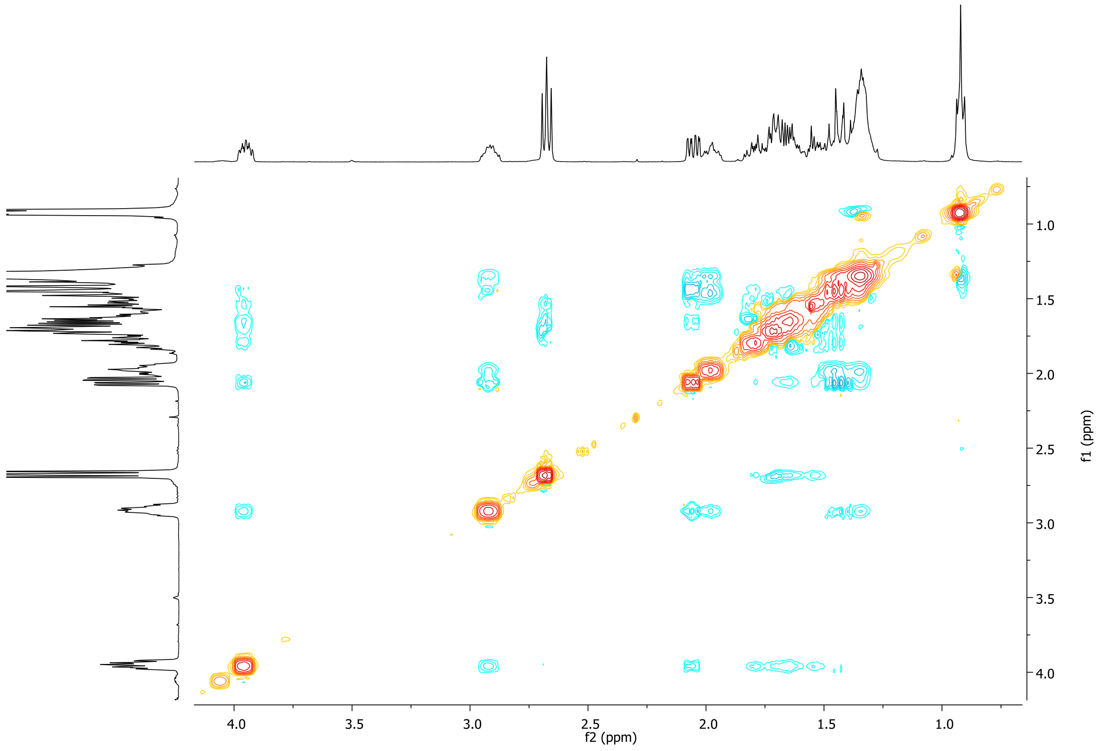

4.0
3.5
3.0
2.5
2.0
1.5
1.0
f2 (ppm)
f1 (ppm)

8. Chiral HPLC chromatography

(*S*,*E*)-5,9-Dimethyldeca-2,8-dienal (SM2d)

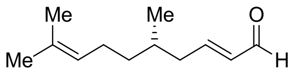

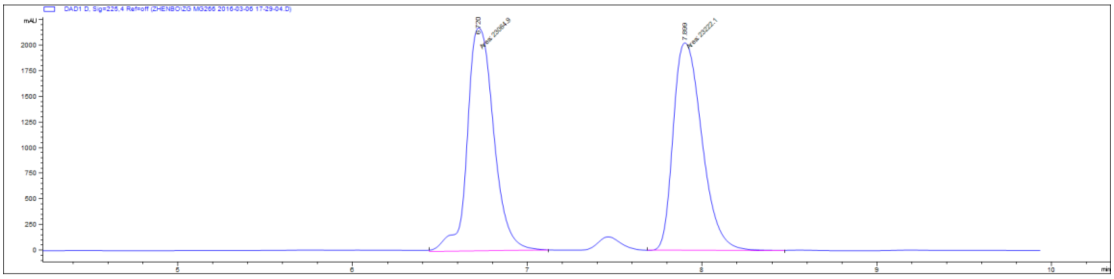

#	Time	Area	Height	Width	Area%	Symmetry
1	6.72	23064.9	2173.1	0.1769	49.830	0.76
2	7.899	23222.1	2021.4	0.1915	50.170	0.662

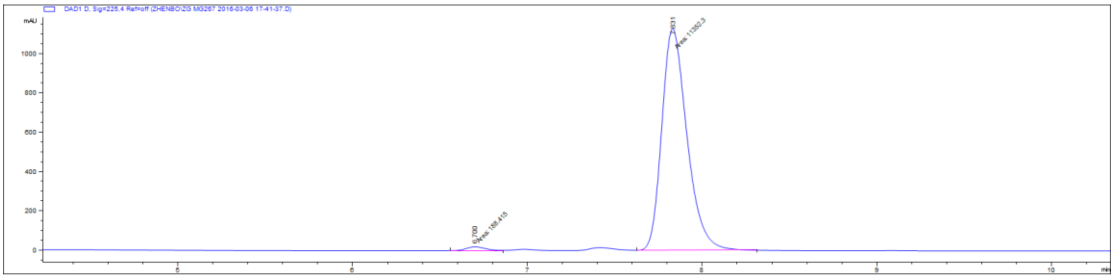

#	Time	Area	Height	Width	Area%	Symmetry
1	6.7	188.4	21.7	0.1449	1.633	0.794
2	7.831	11352.3	1129.6	0.1675	98.367	0.728

(*S*)-6-(4-methoxyphenyl)hexadecan-8-one (4a)

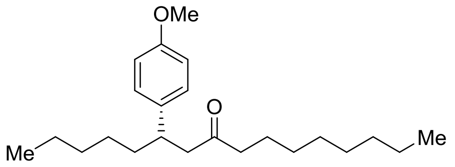

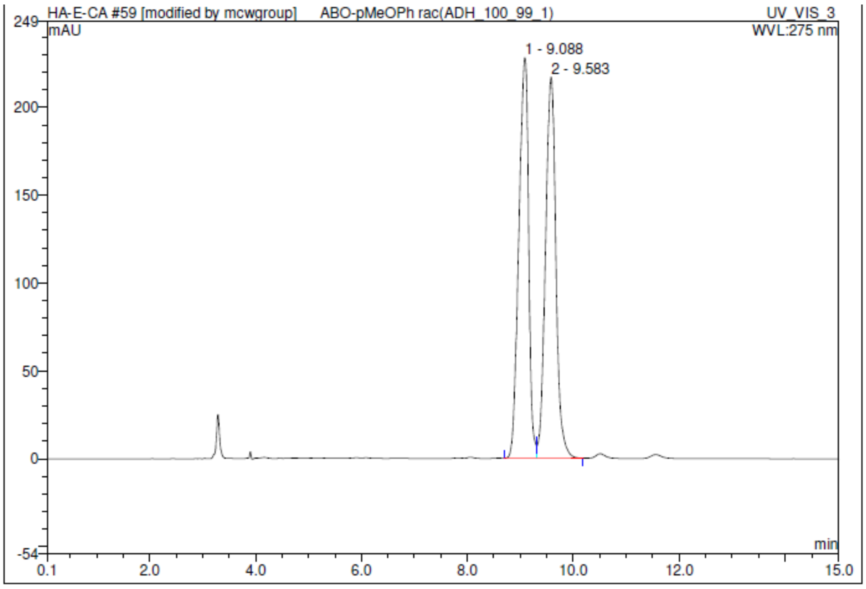

No.	Ret.Time min	Peak Name	Height mAU	Area mAU*min	Rel.Area %	Amount	Type
1	9.09	n.a.	227.918	48.941	49.69	n.a.	BM *
2	9.58	n.a.	216.837	49.555	50.31	n.a.	MB*
Total:			444.755	98.496	100.00	0.000	

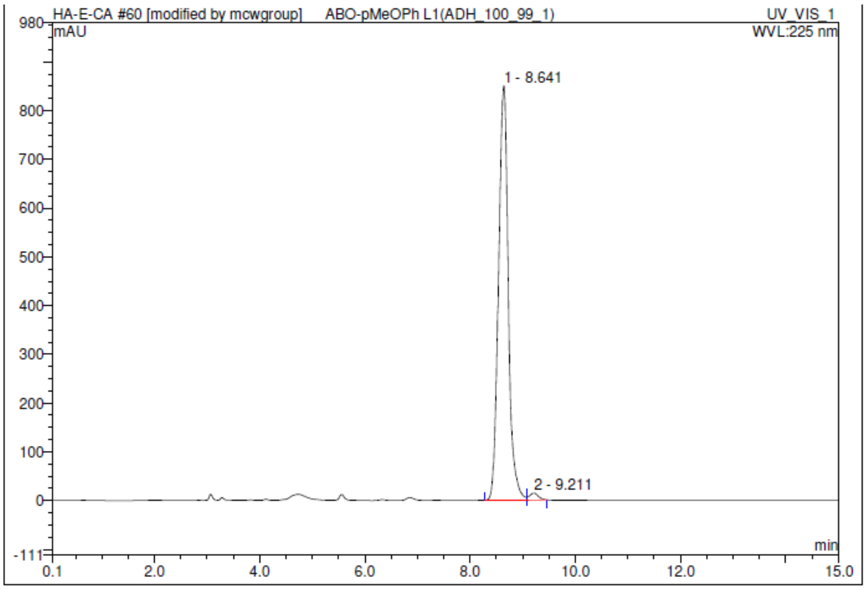

No.	Ret.Time min	Peak Name	Height mAU	Area mAU*min	Rel.Area %	Amount	Type
1	8.64	n.a.	850.493	183.035	98.53	n.a.	BM *
2	9.21	n.a.	14.238	2.733	1.47	n.a.	MB*
Total:			864.731	185.768	100.00	0.000	

(*S*)-1-hydroxy-8-(4-methoxyphenyl)tridecan-6-one (4v)

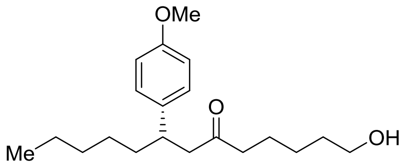

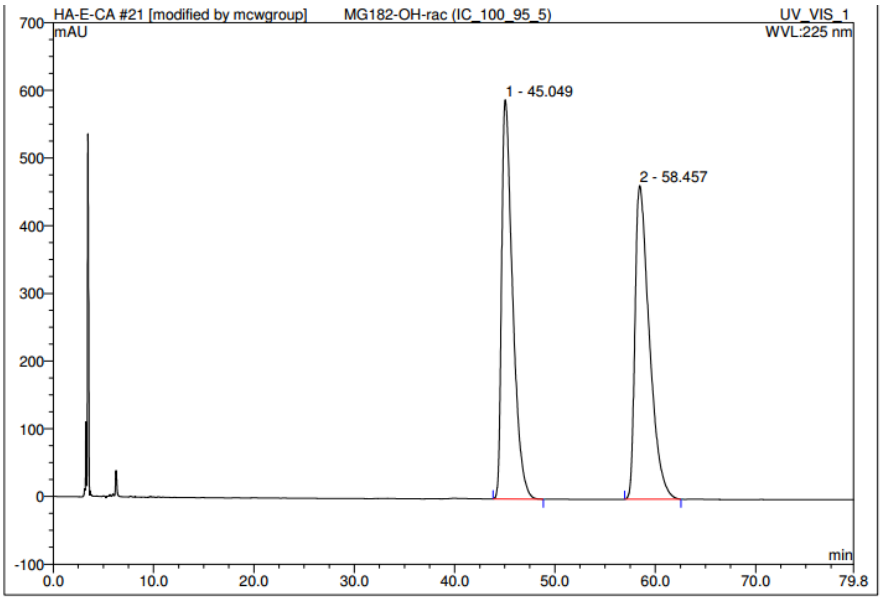

No.	Ret.Time min	Peak Name	Height mAU	Area mAU*min	Rel.Area %	Amount	Type
1	45.05	n.a.	589.404	748.431	49.38	n.a.	BMB*
2	58.46	n.a.	463.205	767.266	50.62	n.a.	BMB*
Total:			1052.609	1515.698	100.00	0.000	

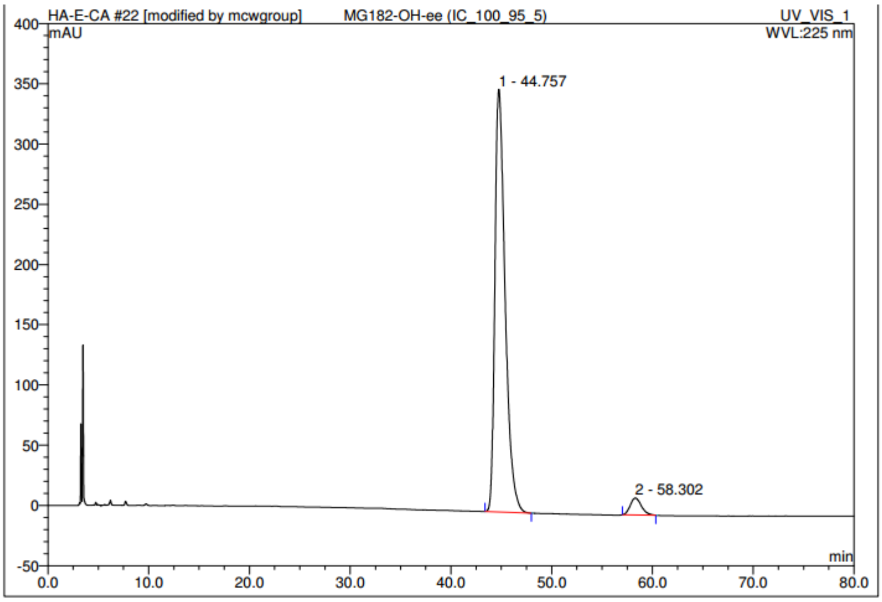

No.	Ret.Time min	Peak Name	Height mAU	Area mAU*min	Rel.Area %	Amount	Type
1	44.76	n.a.	350.883	412.574	95.80	n.a.	BMB*
2	58.30	n.a.	14.077	18.082	4.20	n.a.	BMB*
Total:			364.961	430.657	100.00	0.000	

(*S*)-7-(2-Methoxyphenyl)-1-phenyldodecan-5-one (4b)

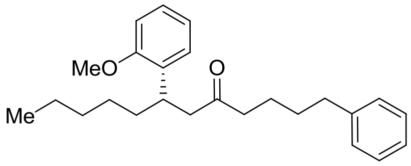

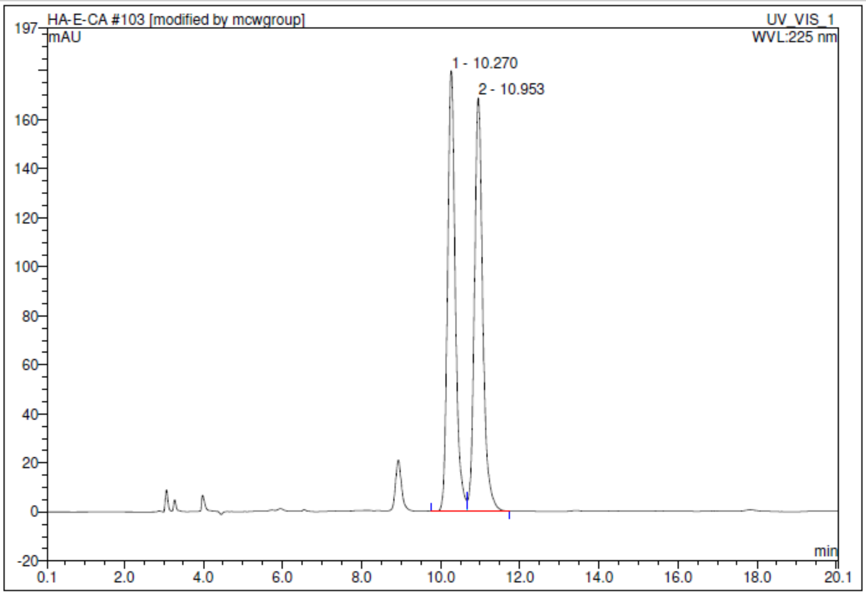

No.	Ret.Time min	Peak Name	Height mAU	Area mAU*min	Rel.Area %	Amount	Type
1	10.27	n.a.	179.510	41.023	49.88	n.a.	BM *
2	10.95	n.a.	168.535	41.223	50.12	n.a.	MB*
Total:			348.044	82.246	100.00	0.000	

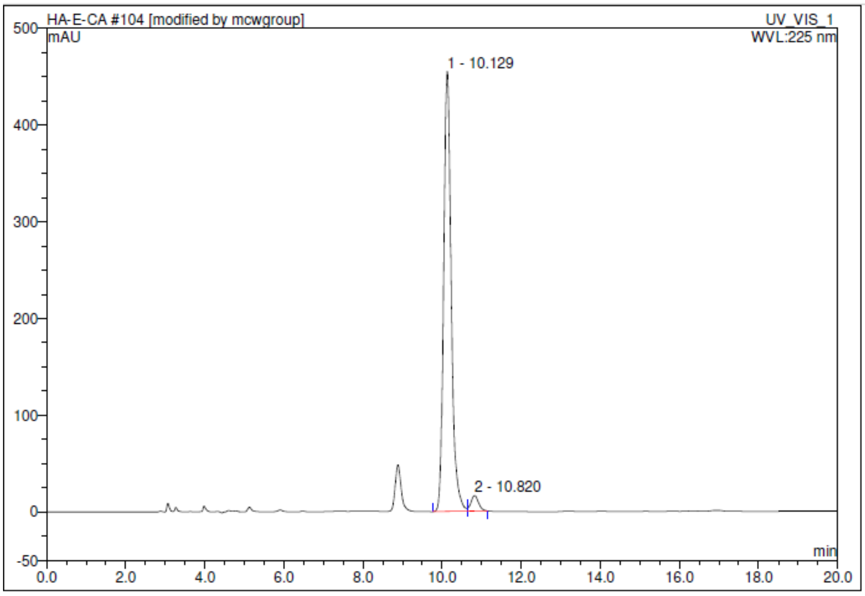

No.	Ret.Time min	Peak Name	Height mAU	Area mAU*min	Rel.Area %	Amount	Type
1	10.13	n.a.	454.957	100.757	96.57	n.a.	BM *
2	10.82	n.a.	15.979	3.576	3.43	n.a.	MB*
Total:			470.936	104.333	100.00	0.000	

(*S*)-6-(*p*-Tolyl)hexadecan-8-one 1b (4c)

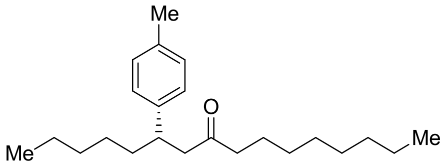

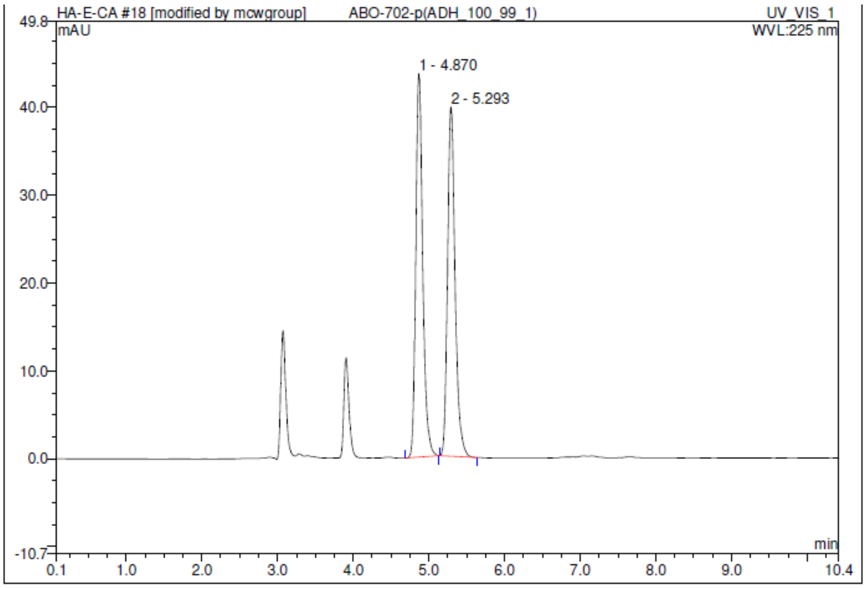

No.	Ret.Time min	Peak Name	Height mAU	Area mAU*min	Rel.Area %	Amount	Type
1	4.87	n.a.	43.655	4.633	50.26	n.a.	BMB*
2	5.29	n.a.	39.755	4.585	49.74	n.a.	BMB*
Total:			83.410	9.218	100.00	0.000	

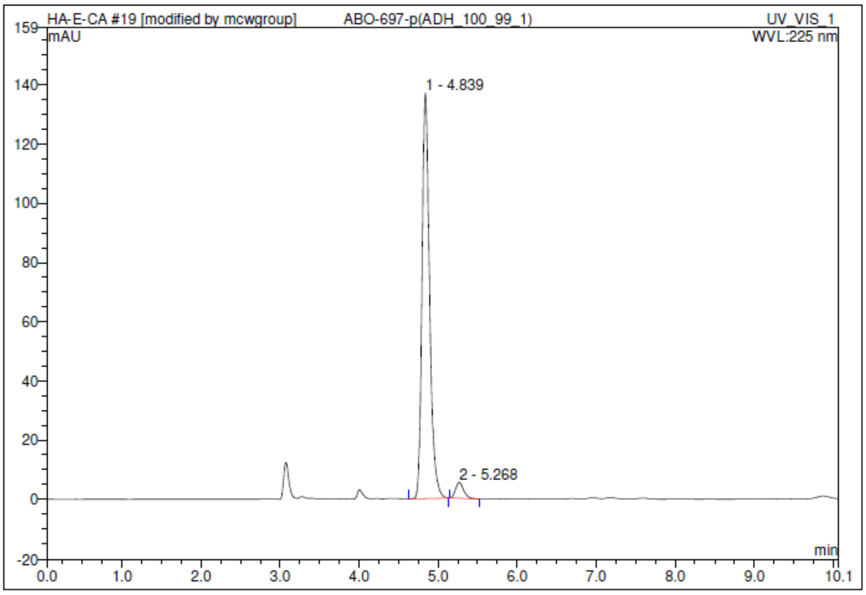

No.	Ret.Time min	Peak Name	Height mAU	Area mAU*min	Rel.Area %	Amount	Type
1	4.84	n.a.	136.867	15.171	95.97	n.a.	BMB*
2	5.27	n.a.	5.359	0.638	4.03	n.a.	BMB*
Total:			142.225	15.808	100.00	0.000	

(*S*)-7-[4-(*tert*-Butyl)phenyl]-1-phenyldodecan-5-one (4d)

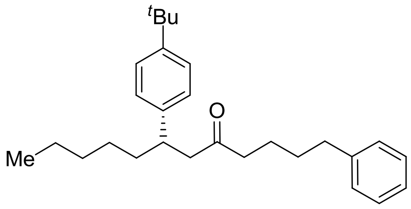

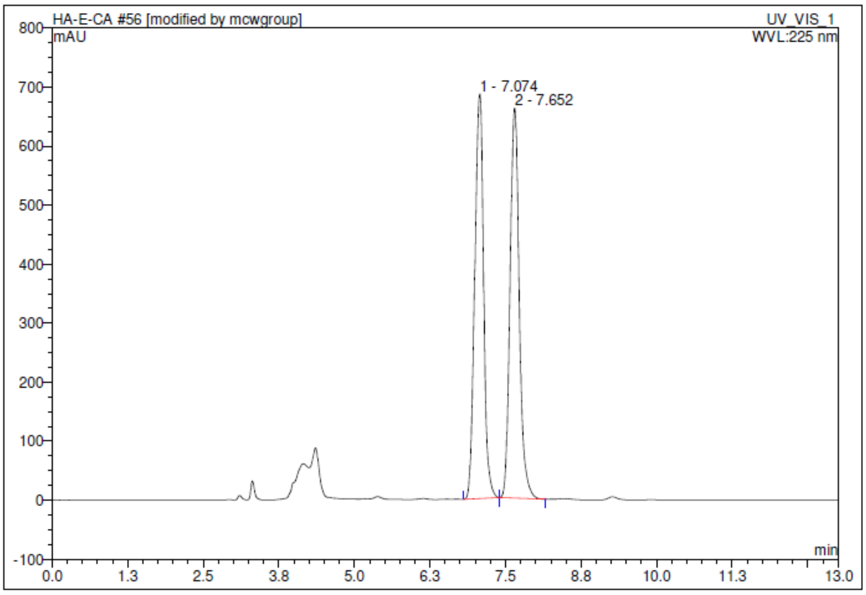

No.	Ret.Time min	Peak Name	Height mAU	Area mAU*min	Rel.Area %	Amount	Type
1	7.07	n.a.	685.643	116.353	49.92	n.a.	BMB*
2	7.65	n.a.	660.815	116.730	50.08	n.a.	BMB*
Total:			1346.459	233.083	100.00	0.000	

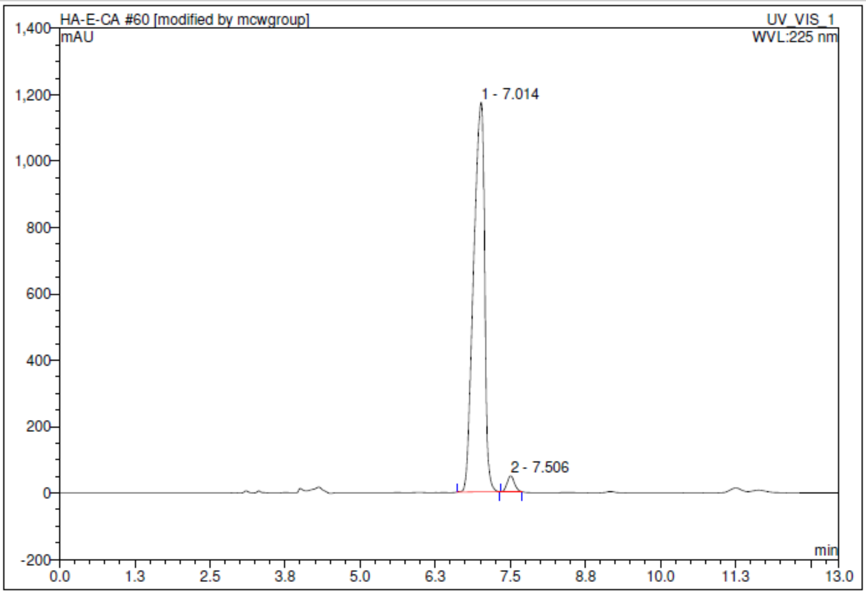

No.	Ret.Time min	Peak Name	Height mAU	Area mAU*min	Rel.Area %	Amount	Type
1	7.01	n.a.	1173.358	248.127	97.35	n.a.	BMB*
2	7.51	n.a.	47.688	6.763	2.65	n.a.	BMB*
Total:			1221.046	254.891	100.00	0.000	

(*S*)-1,7-Diphenyldodecan-5-one (4e)

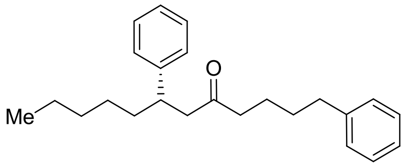

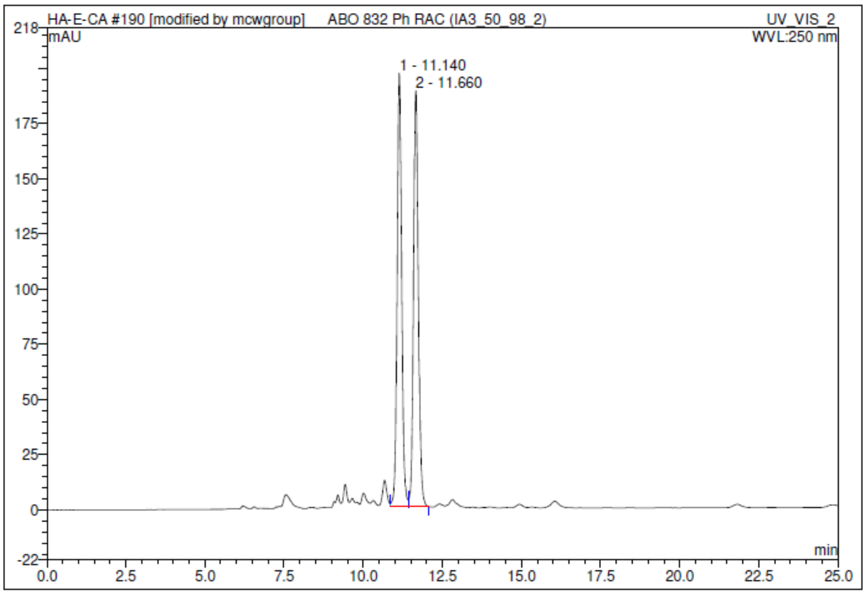

No.	Ret.Time min	Peak Name	Height mAU	Area mAU*min	Rel.Area %	Amount	Type
1	11.14	n.a.	196.227	32.974	49.69	n.a.	M *
2	11.66	n.a.	188.302	33.382	50.31	n.a.	MB*
Total:			384.529	66.355	100.00	0.000	

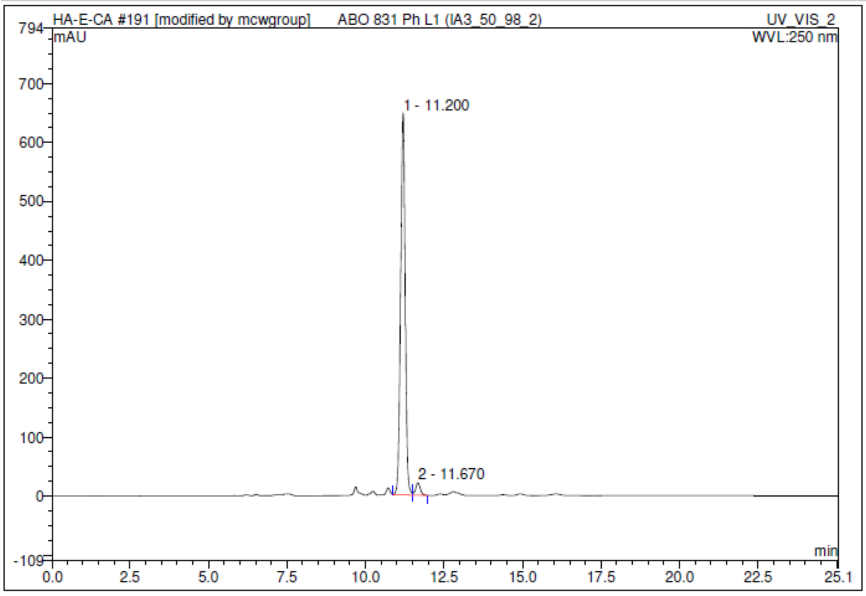

No.	Ret.Time min	Peak Name	Height mAU	Area mAU*min	Rel.Area %	Amount	Type
1	11.20	n.a.	648.200	109.871	96.81	n.a.	BM *
2	11.67	n.a.	21.160	3.618	3.19	n.a.	MB*
Total:			669.360	113.489	100.00	0.000	

(*S*)-7-(Naphthalen-2-yl)-1-phenyldodecan-5-one (4f)

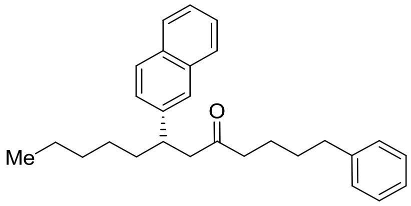

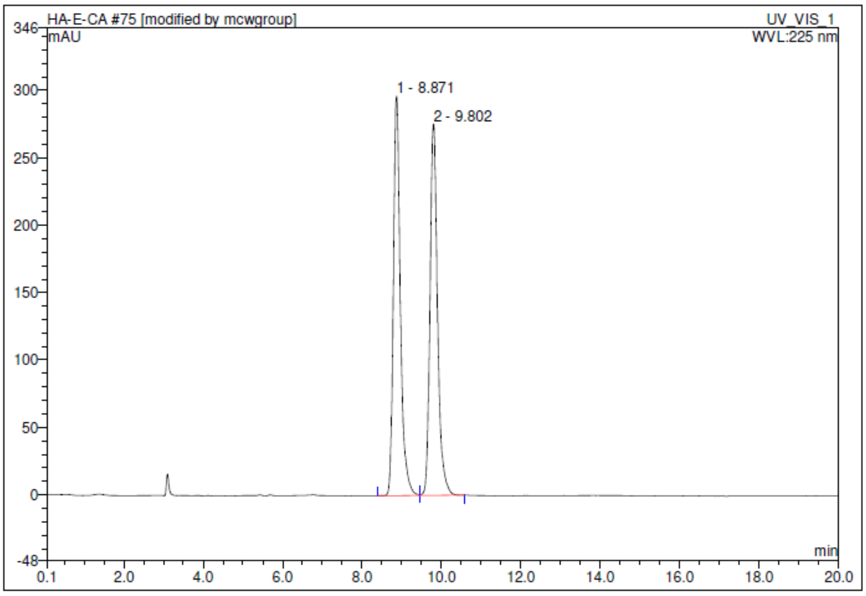

No.	Ret.Time min	Peak Name	Height mAU	Area mAU*min	Rel.Area %	Amount	Type
1	8.87	n.a.	296.130	60.488	50.05	n.a.	BM *
2	9.80	n.a.	275.331	60.369	49.95	n.a.	MB*
Total:			571.461	120.857	100.00	0.000	

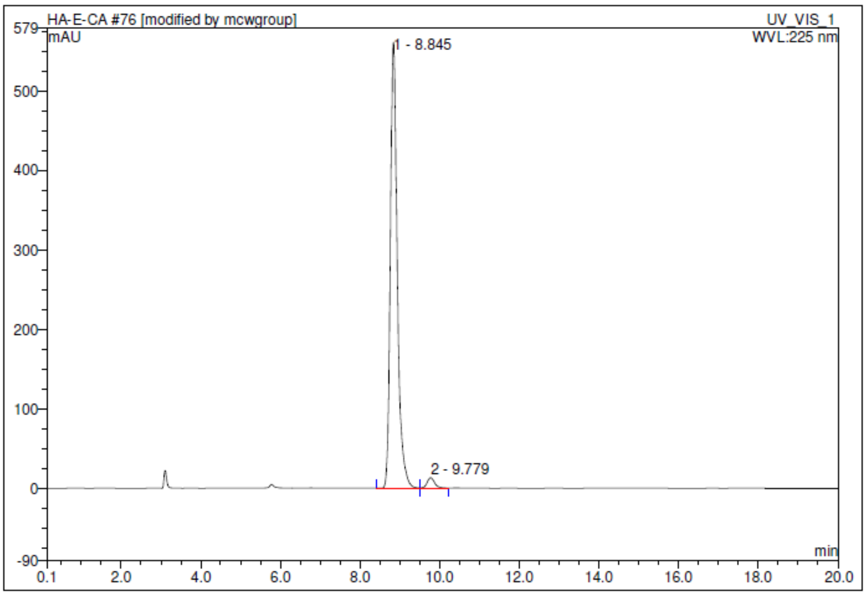

No.	Ret.Time min	Peak Name	Height mAU	Area mAU*min	Rel.Area %	Amount	Type
1	8.84	n.a.	561.235	113.842	97.52	n.a.	BM *
2	9.78	n.a.	13.143	2.899	2.48	n.a.	MB*
Total:			574.378	116.742	100.00	0.000	

(*S*)-7-(3-Hydroxyphenyl)-1-phenyldodecan-5-one (4g)

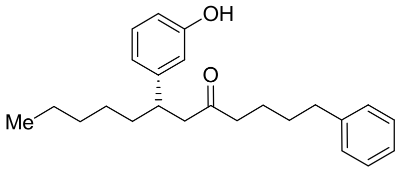

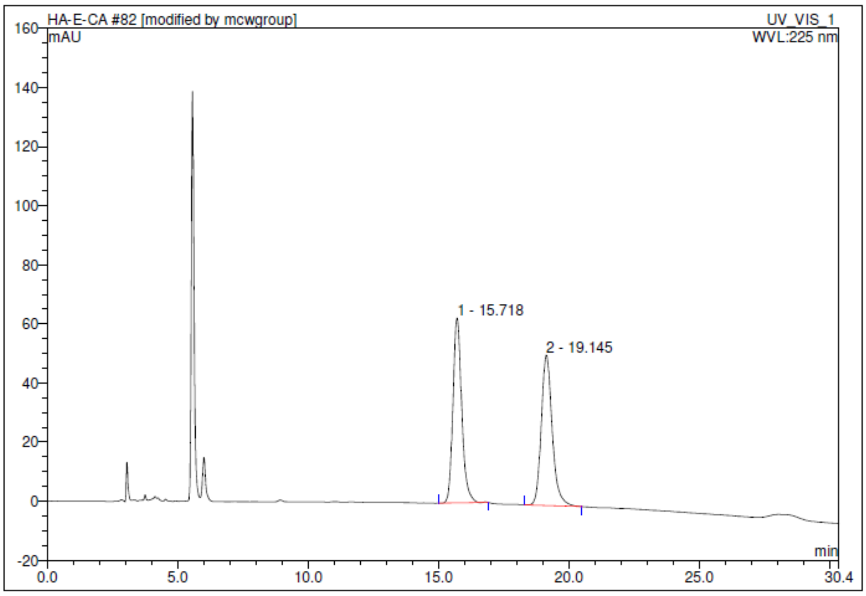

No.	Ret.Time min	Peak Name	Height mAU	Area mAU*min	Rel.Area %	Amount	Type
1	15.72	n.a.	62.506	24.455	49.66	n.a.	BMB*
2	19.15	n.a.	50.895	24.785	50.34	n.a.	BMB
Total:			113.401	49.240	100.00	0.000	

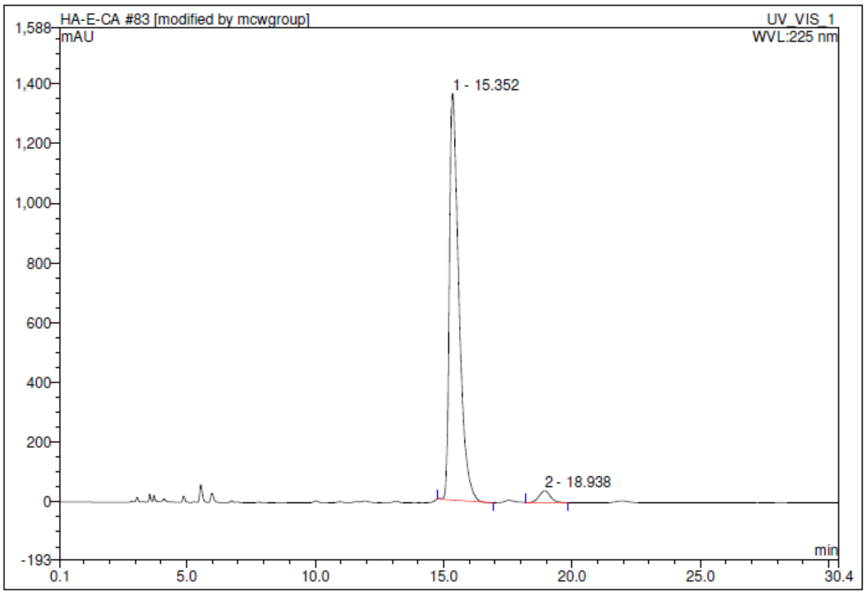

No.	Ret.Time min	Peak Name	Height mAU	Area mAU*min	Rel.Area %	Amount	Type
1	15.35	n.a.	1362.005	584.863	96.69	n.a.	BMB*
2	18.94	n.a.	39.327	20.018	3.31	n.a.	BMB*
Total:			1401.332	604.881	100.00	0.000	

(*S*)-7-[4-(Dimethylamino)phenyl]-1-phenyldodecan-5-one (4h)

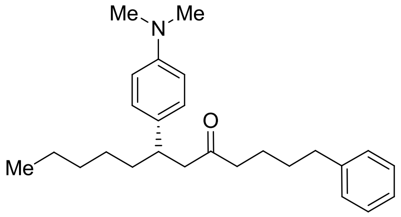

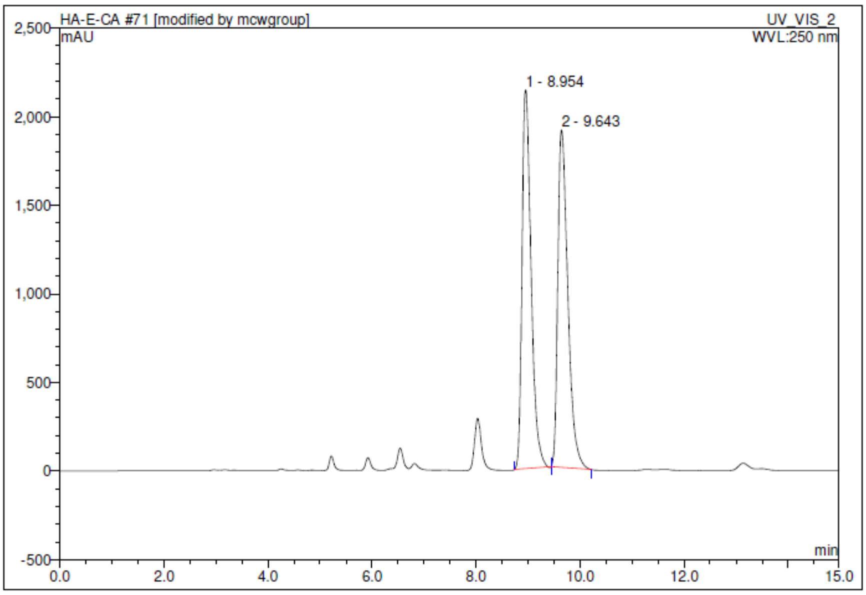

No.	Ret.Time min	Peak Name	Height mAU	Area mAU*min	Rel.Area %	Amount	Type
1	8.95	n.a.	2138.372	430.698	50.32	n.a.	BMb*
2	9.64	n.a.	1906.609	425.298	49.68	n.a.	bMB*
Total:			4044.981	855.996	100.00	0.000	

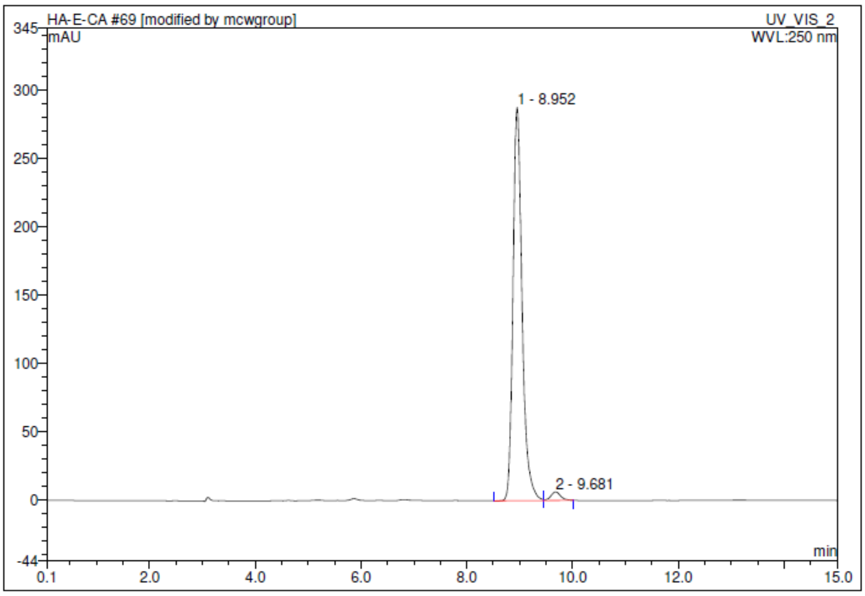

No.	Ret.Time min	Peak Name	Height mAU	Area mAU*min	Rel.Area %	Amount	Type
1	8.95	n.a.	287.399	57.504	97.76	n.a.	BM *
2	9.68	n.a.	6.211	1.317	2.24	n.a.	MB*
Total:			293.610	58.822	100.00	0.000	

(*S*)-6-(4-Hydroxyphenyl)hexadecan-8-one (4i)

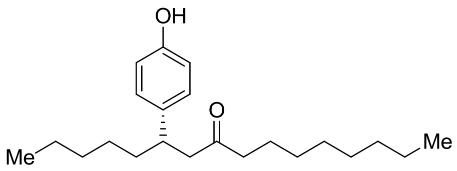

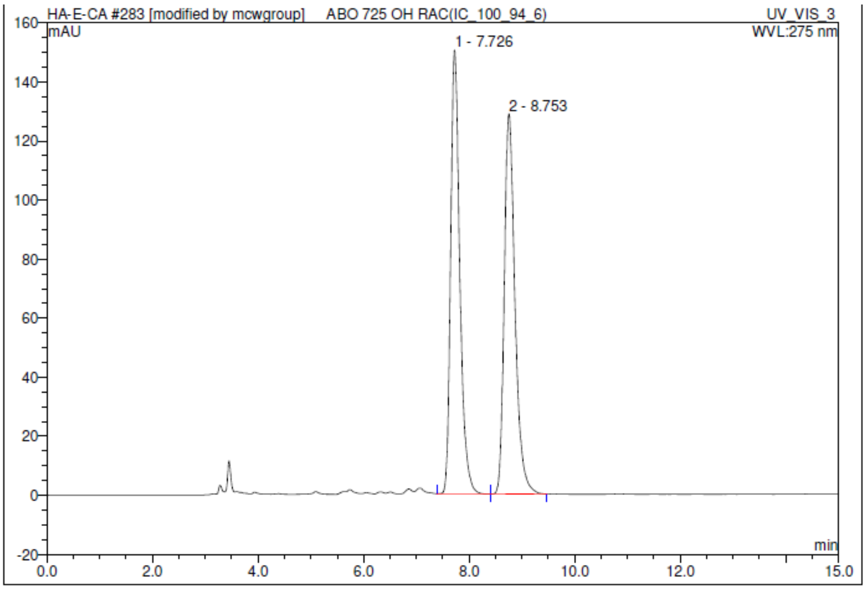

No.	Ret.Time min	Peak Name	Height mAU	Area mAU*min	Rel.Area %	Amount	Type
1	7.73	n.a.	150.201	30.250	49.94	n.a.	BM *
2	8.75	n.a.	128.794	30.320	50.06	n.a.	MB*
Total:			278.994	60.570	100.00	0.000	

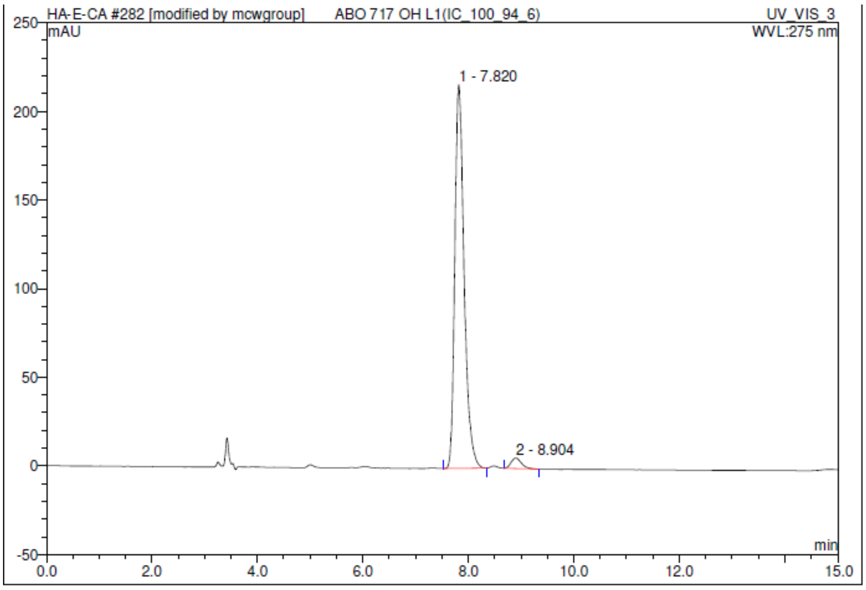

No.	Ret.Time min	Peak Name	Height mAU	Area mAU*min	Rel.Area %	Amount	Type
1	7.82	n.a.	216.529	44.119	97.04	n.a.	BMB*
2	8.90	n.a.	5.913	1.347	2.96	n.a.	BMB*
Total:			222.441	45.467	100.00	0.000	

(*S*)-6-(4-Bromophenyl)hexadecan-8-one (4j)

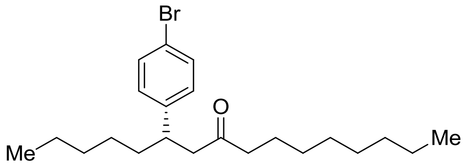

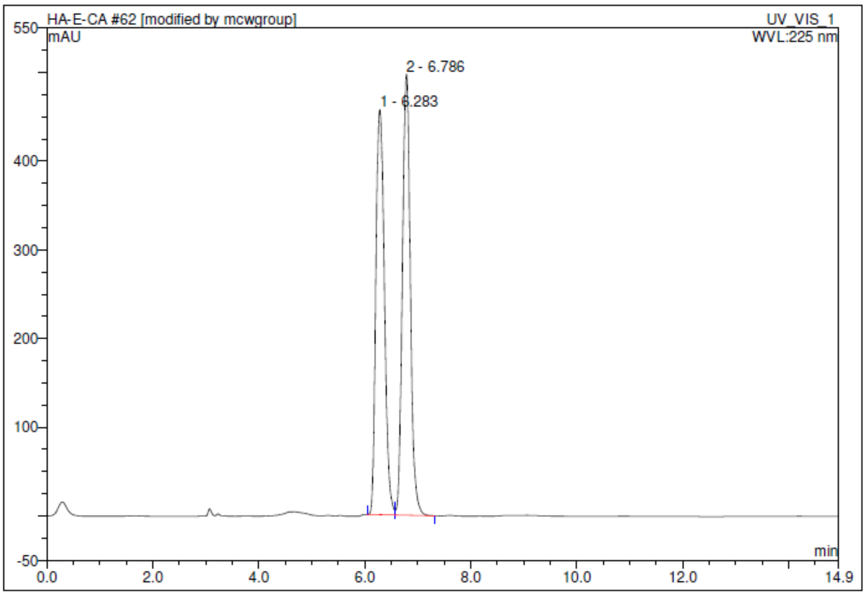

No.	Ret.Time min	Peak Name	Height mAU	Area mAU*min	Rel.Area %	Amount	Type
1	6.28	n.a.	456.646	85.469	49.73	n.a.	BM *
2	6.79	n.a.	497.271	86.396	50.27	n.a.	MB*
Total:			953.917	171.865	100.00	0.000	

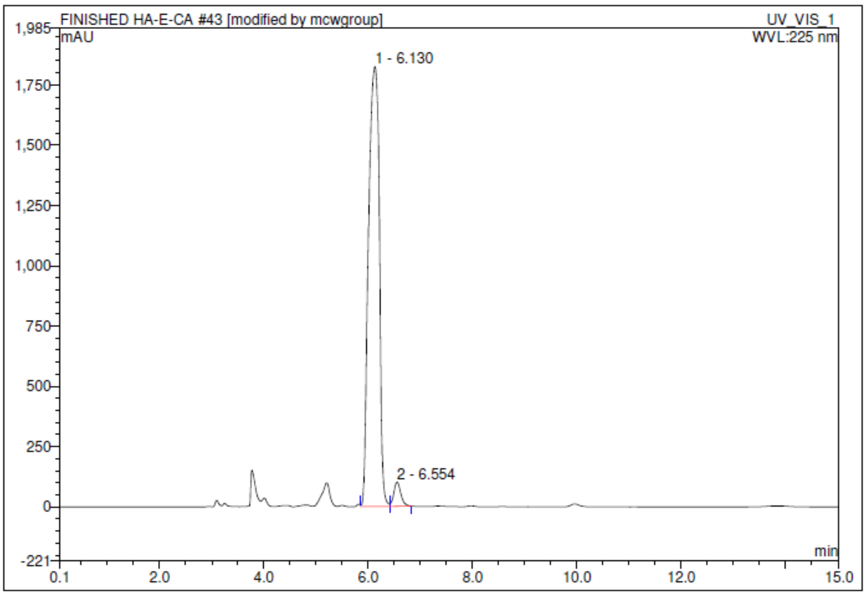

No.	Ret.Time min	Peak Name	Height mAU	Area mAU*min	Rel.Area %	Amount	Type
1	6.13	n.a.	1823.824	434.110	96.51	n.a.	M *
2	6.55	n.a.	100.503	15.709	3.49	n.a.	MB*
Total:			1924.327	449.819	100.00	0.000	

(*S*)-Methyl 4-(8-oxohexadecan-6-yl)benzoate (4k)

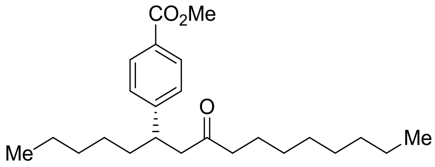

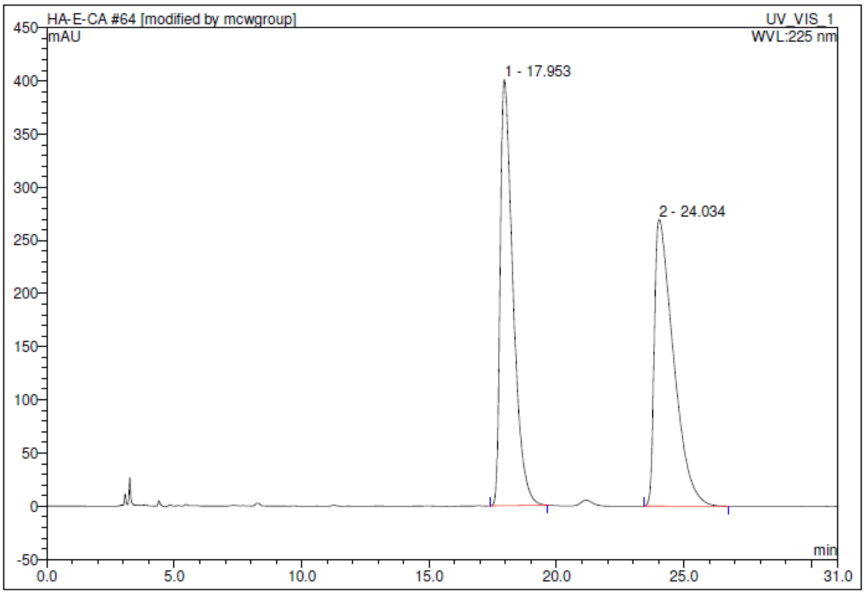

No.	Ret.Time min	Peak Name	Height mAU	Area mAU*min	Rel.Area %	Amount	Type
1	17.95	n.a.	400.722	232.265	49.70	n.a.	BMB*
2	24.03	n.a.	269.696	235.116	50.30	n.a.	BMB*
Total:			670.418	467.380	100.00	0.000	

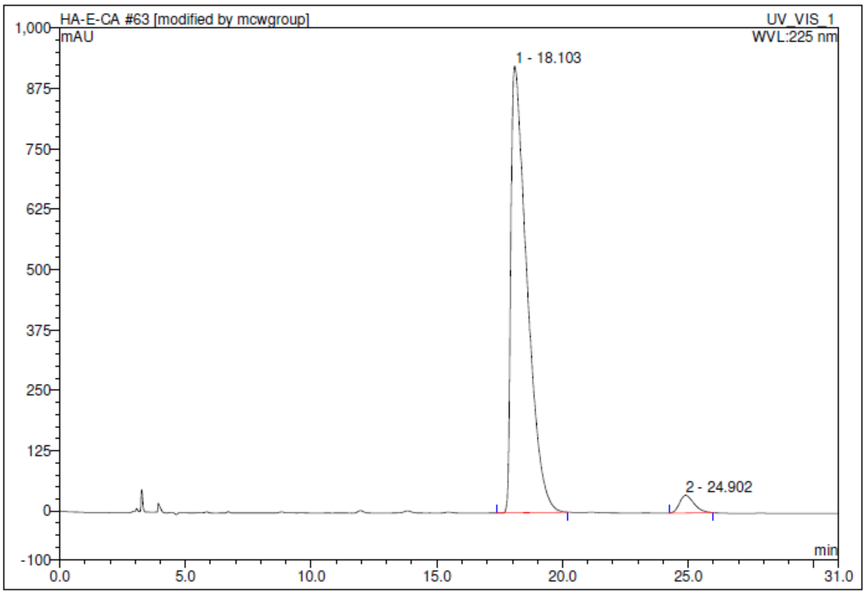

No.	Ret.Time min	Peak Name	Height mAU	Area mAU*min	Rel.Area %	Amount	Type
1	18.10	n.a.	924.740	686.273	96.55	n.a.	BMB*
2	24.90	n.a.	36.507	24.498	3.45	n.a.	BMB*
Total:			961.247	710.770	100.00	0.000	

(*S*)-4-(8-Oxohexadecan-6-yl)benzonitrile 1d (4l)

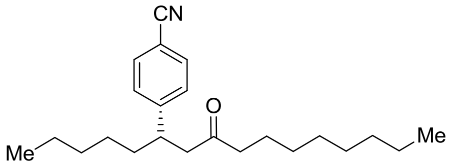

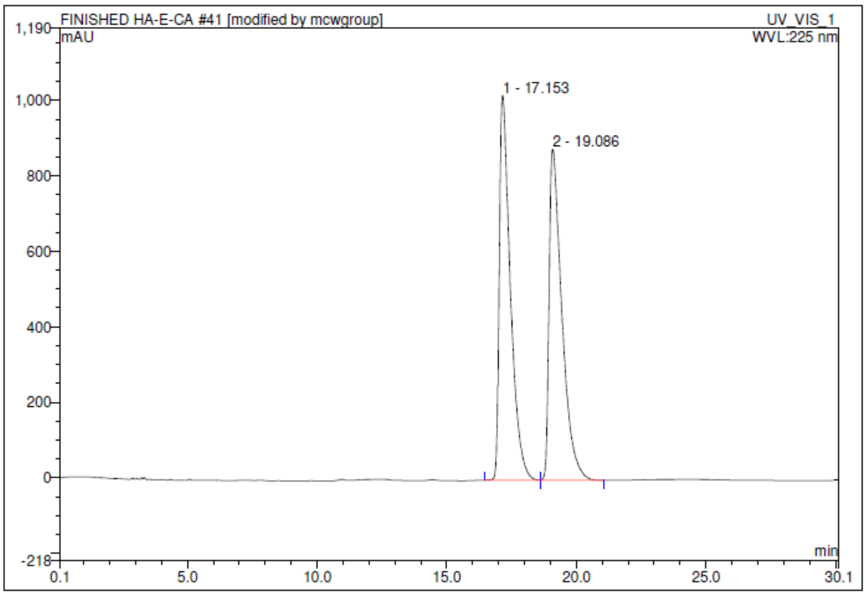

No.	Ret.Time min		Peak Name	Height mAU	Area mAU*min	Rel.Area %	Amount	Type
1	17.15	n.a.		1018.366	502.184	50.02	n.a.	BM *
2	19.09	n.a.		876.706	501.830	49.98	n.a.	MB*
Total:				1895.071	1004.013	100.00	0.000	

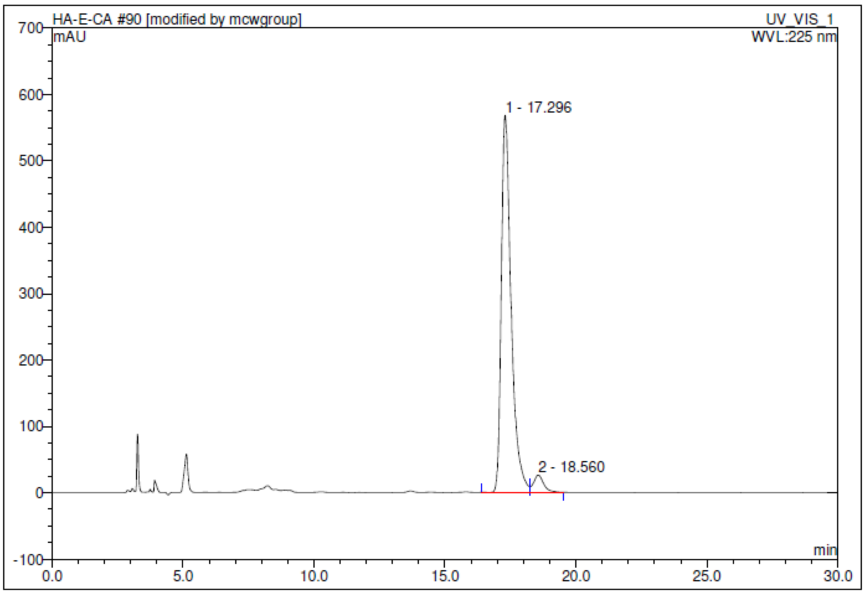

No.	Ret.Time min		Peak Name	Height mAU	Area mAU*min	Rel.Area %	Amount	Type
1	17.30	n.a.		567.860	247.442	95.41	n.a.	BM *
2	18.56	n.a.		25.968	11.898	4.59	n.a.	MB*
Total:				593.828	259.340	100.00	0.000	

(*S*)-7-(2,3-Dihydrobenzo[b][1,4]dioxin-6-yl)-1-phenyldodecan-5-one (4m)

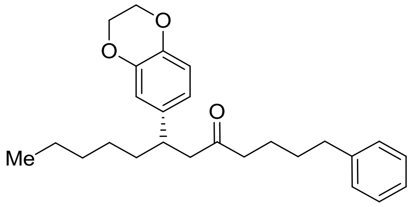

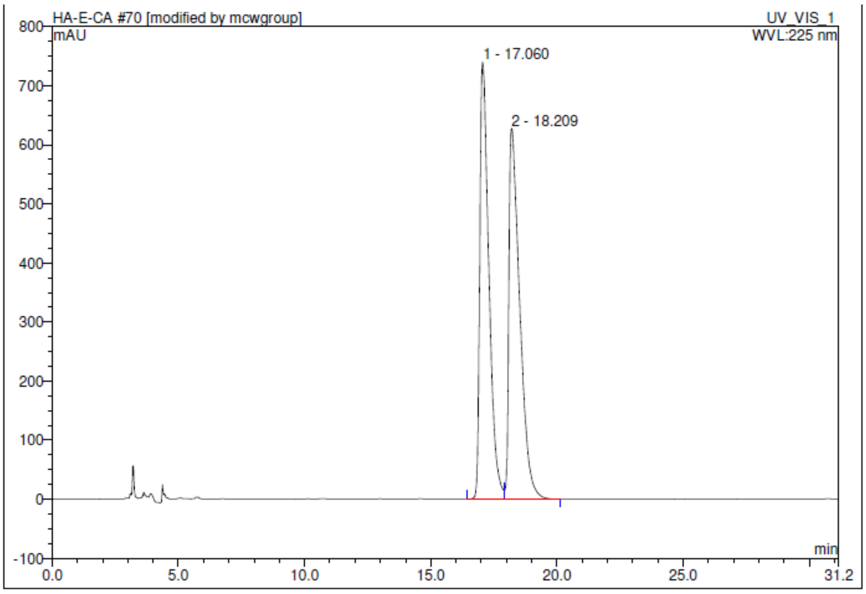

No.	Ret.Time min	Peak Name	Height mAU	Area mAU*min	Rel.Area %	Amount	Type
1	17.06	n.a.	739.272	309.326	49.69	n.a.	BM *
2	18.21	n.a.	627.116	313.203	50.31	n.a.	MB*
Total:			1366.388	622.529	100.00	0.000	

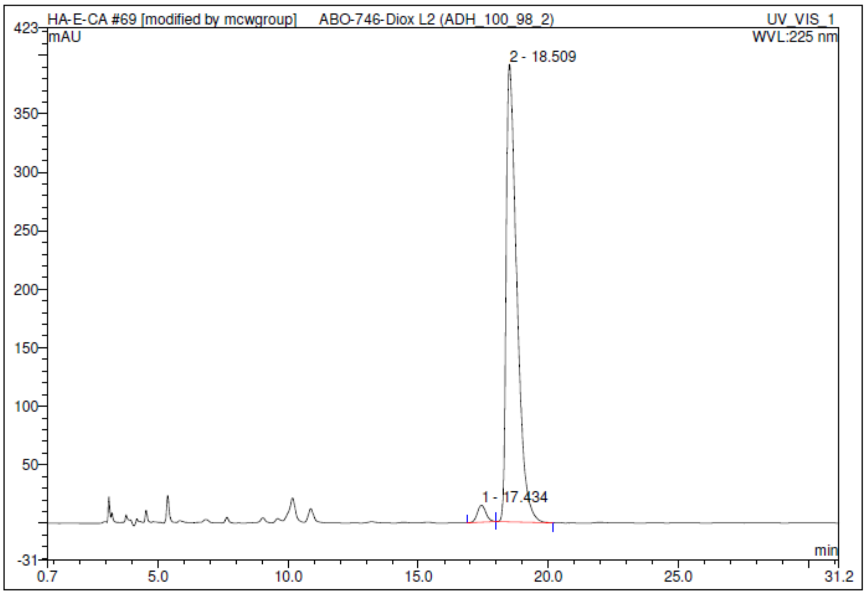

No.	Ret.Time min	Peak Name	Height mAU	Area mAU*min	Rel.Area %	Amount	Type
1	17.43	n.a.	14.590	5.534	2.89	n.a.	BMb*
2	18.51	n.a.	391.081	185.653	97.11	n.a.	bMB*
Total:			405.670	191.187	100.00	0.000	

(*S*)-1-Phenyl-7-(thiophen-3-yl)dodecan-5-one (4n)

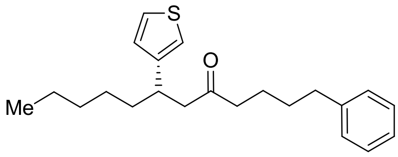

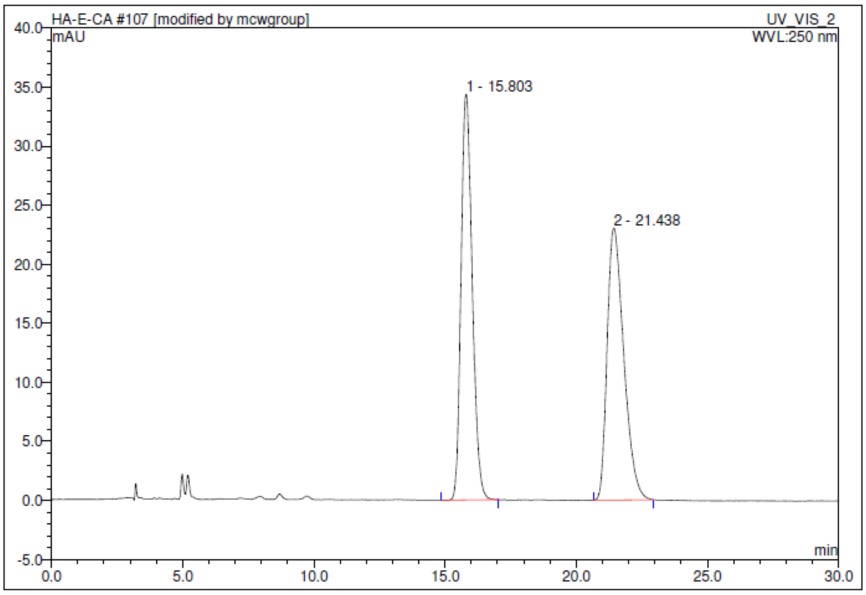

No.	Ret.Time min	Peak Name	Height mAU	Area mAU*min	Rel.Area %	Amount	Type
1	15.80	n.a.	34.368	16.965	50.09	n.a.	BMB*
2	21.44	n.a.	23.031	16.901	49.91	n.a.	BMB*
Total:			57.399	33.866	100.00	0.000	

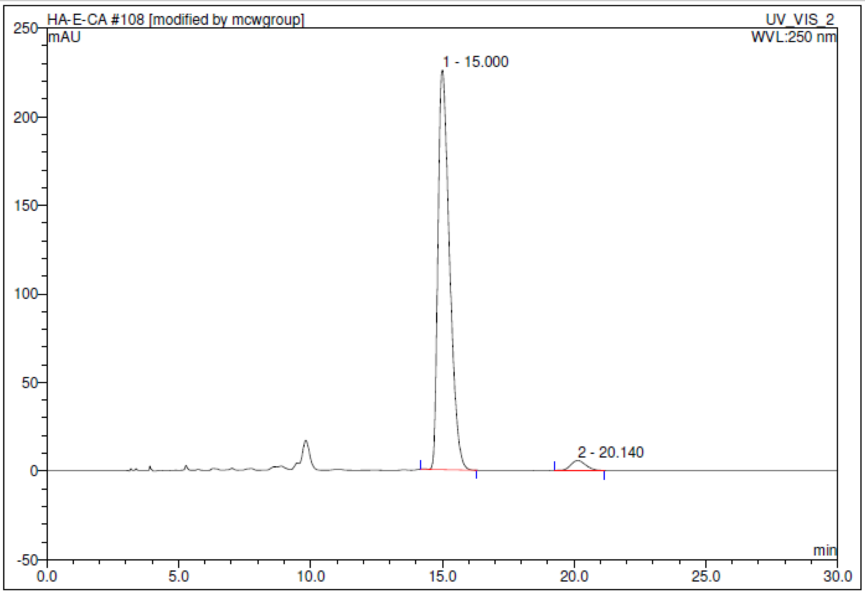

No.	Ret.Time min	Peak Name	Height mAU	Area mAU*min	Rel.Area %	Amount	Type
1	15.00	n.a.	225.710	114.561	96.84	n.a.	BMB*
2	20.14	n.a.	5.664	3.742	3.16	n.a.	BMB*
Total:			231.374	118.302	100.00	0.000	

(*S*)-2-[8-(Cyclohex-1-en-1-yl)-6-oxotridecyl]isoindoline-1,3-dione (4o)

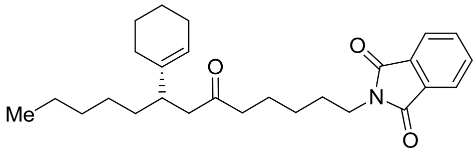

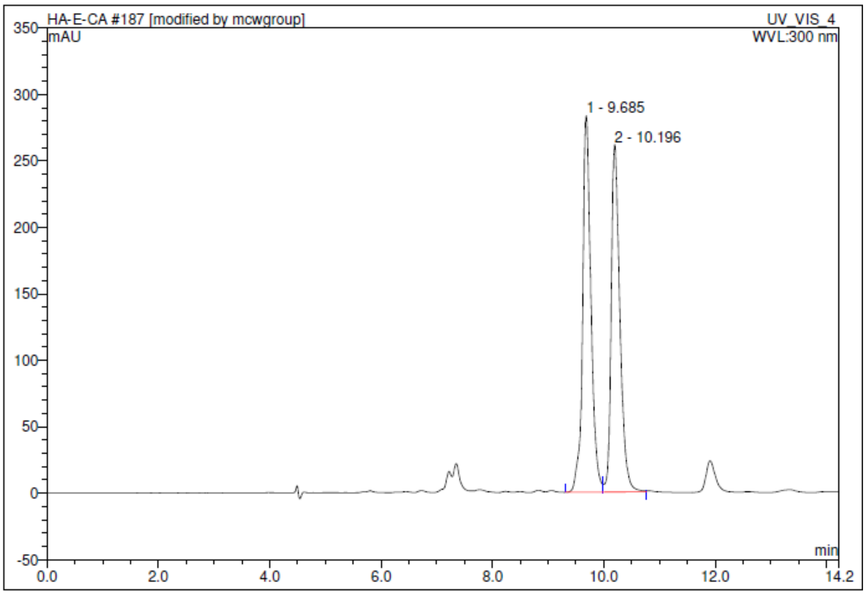

No.	Ret.Time min	Peak Name	Height mAU	Area mAU*min	Rel.Area %	Amount	Type
1	9.68	n.a.	283.349	49.383	51.08	n.a.	BM *
2	10.20	n.a.	261.498	47.304	48.92	n.a.	M *
Total:			544.847	96.688	100.00	0.000	

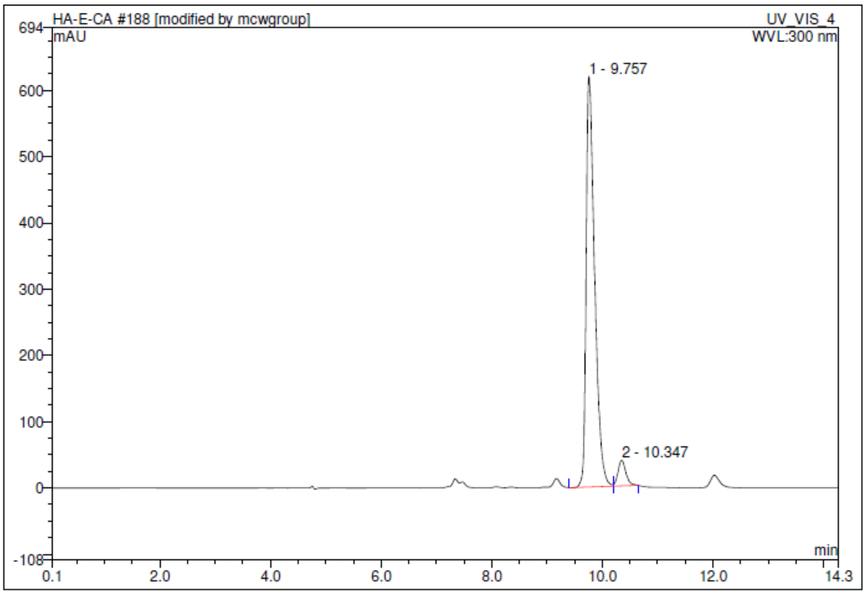

No.	Ret.Time min	Peak Name	Height mAU	Area mAU*min	Rel.Area %	Amount	Type
1	9.76	n.a.	619.543	110.431	94.53	n.a.	BM *
2	10.35	n.a.	38.313	6.385	5.47	n.a.	MB*
Total:			657.856	116.815	100.00	0.000	

(*S*)-1-Cyclopentyl-6-(4-methoxyphenyl)undecan-4-one (4p)

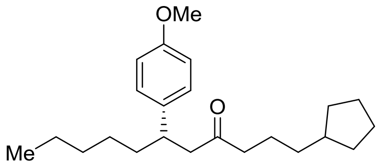

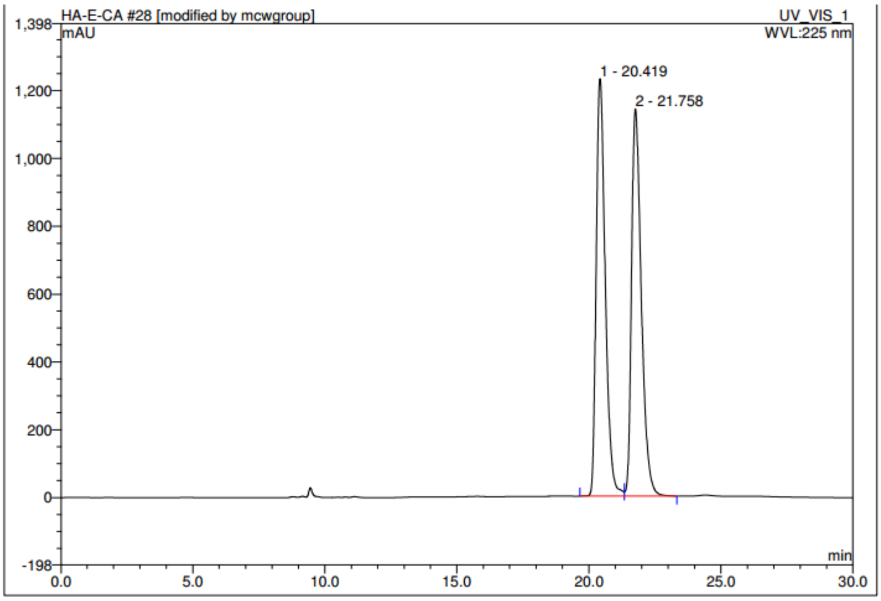

No.	Ret.Time min	Peak Name	Height mAU	Area mAU*min	Rel.Area %	Amount	Type
1	20.42	n.a.	1230.262	490.240	50.00	n.a.	BM *
2	21.76	n.a.	1142.061	490.177	50.00	n.a.	MB*
Total:			2372.323	980.417	100.00	0.000	

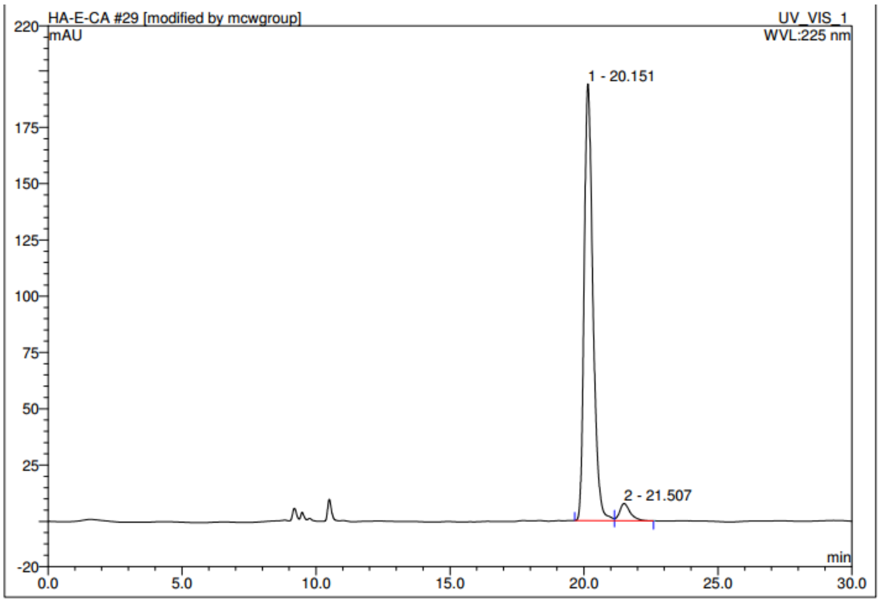

No.	Ret.Time min	Peak Name	Height mAU	Area mAU*min	Rel.Area %	Amount	Type
1	20.15	n.a.	193.934	73.393	95.43	n.a.	BM *
2	21.51	n.a.	7.687	3.518	4.57	n.a.	MB*
Total:			201.621	76.911	100.00	0.000	

(*S*)-7-(4-Methoxyphenyl)-1-phenyldodecan-5-one (4q)

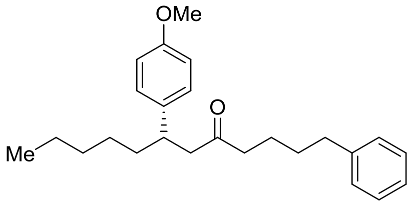

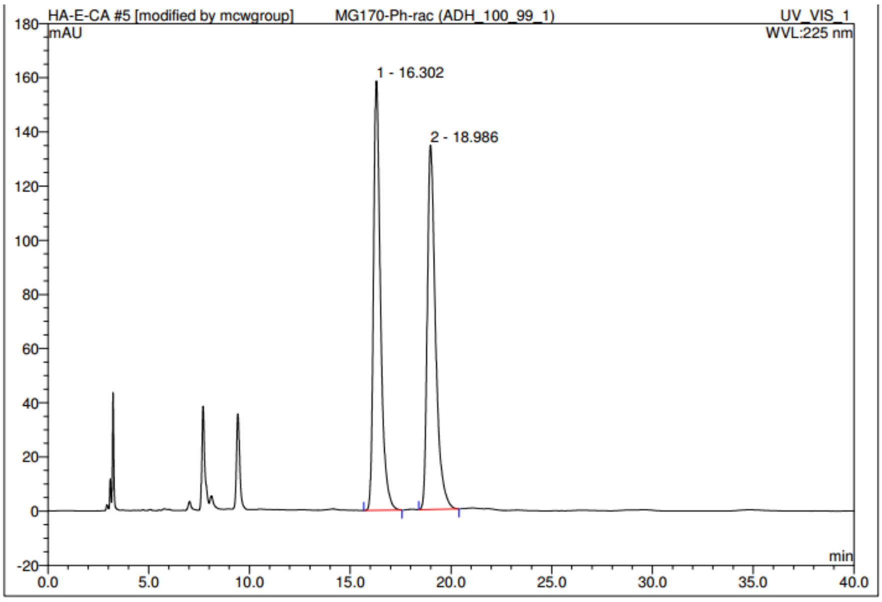

No.	Ret.Time min		Peak Name	Height mAU	Area mAU*min	Rel.Area %	Amount	Type
1	16.30	n.a.		158.549	64.041	50.06	n.a.	BMB
2	18.99	n.a.		134.523	63.875	49.94	n.a.	BMB*
Total:				293.072	127.916	100.00	0.000	

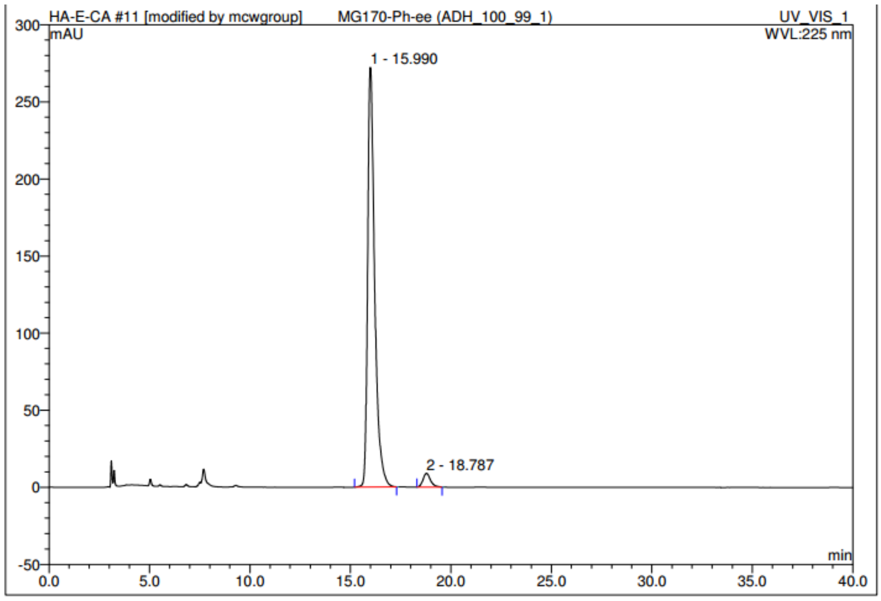

No.	Ret.Time min		Peak Name	Height mAU	Area mAU*min	Rel.Area %	Amount	Type
1	15.99	n.a.		272.307	110.013	96.65	n.a.	BMB*
2	18.79	n.a.		8.847	3.808	3.35	n.a.	BMB*
Total:				281.154	113.821	100.00	0.000	

(*S*)-9-(4-Methoxyphenyl)tetradecane-2,7-dione (4r)

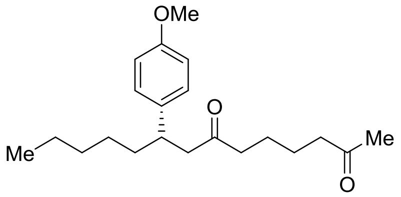

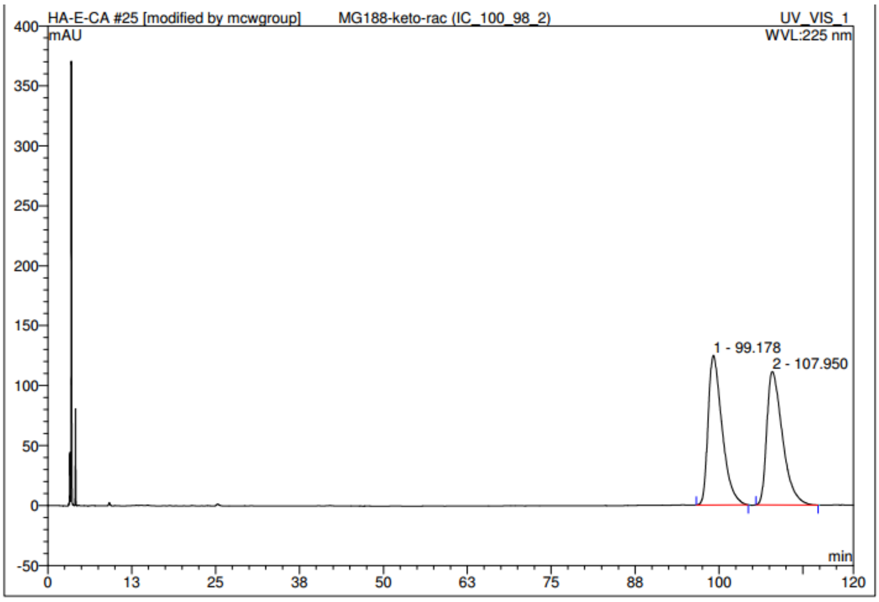

No.	Ret.Time min	Peak Name	Height mAU	Area mAU*min	Rel.Area %	Amount	Type
1	99.18	n.a.	124.746	301.173	50.00	n.a.	BMB*
2	107.95	n.a.	111.096	301.176	50.00	n.a.	BMB*
Total:			235.842	602.349	100.00	0.000	

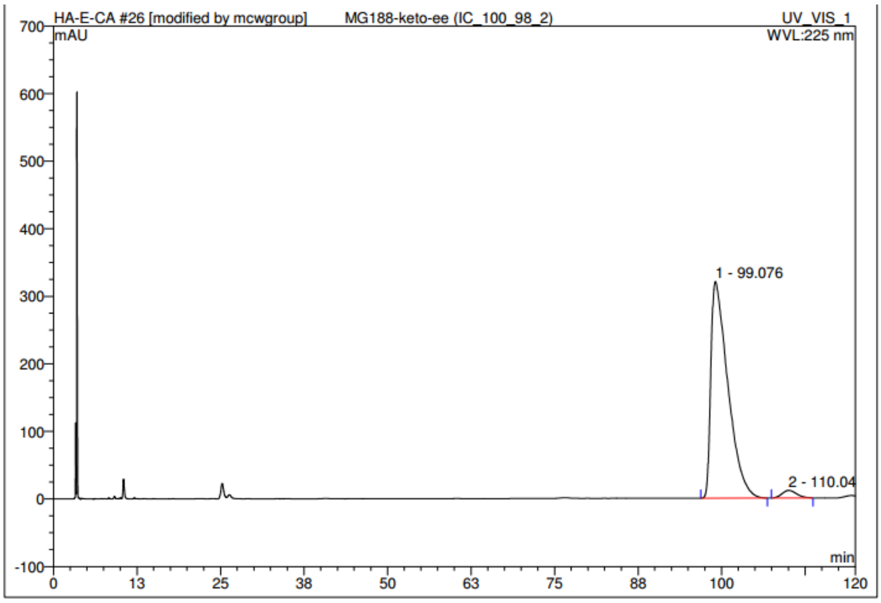

No.	Ret.Time min	Peak Name	Height mAU	Area mAU*min	Rel.Area %	Amount	Type
1	99.08	n.a.	320.794	946.003	97.13	n.a.	BMB*
2	110.04	n.a.	11.043	27.958	2.87	n.a.	BMB*
Total:			331.837	973.961	100.00	0.000	

(*S*)-Diethyl 2-[6-(4-methoxyphenyl)-4-oxoundecyl]malonate (4s)

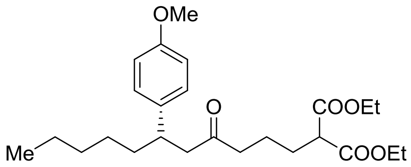

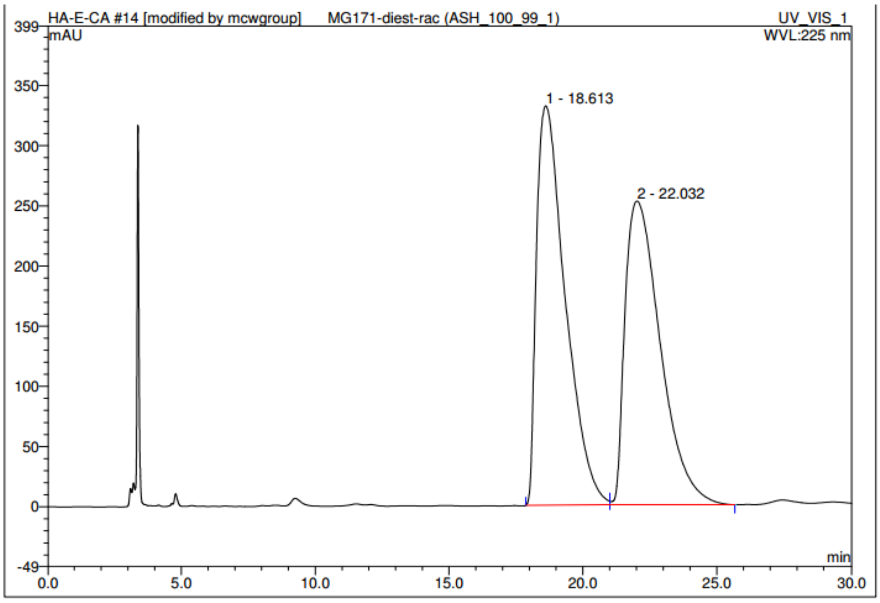

No.	Ret.Time min	Peak Name	Height mAU	Area mAU*min	Rel.Area %	Amount	Type
1	18.61	n.a.	331.715	412.847	51.66	n.a.	BM *
2	22.03	n.a.	252.220	386.255	48.34	n.a.	MB*
Total:			583.935	799.102	100.00	0.000	

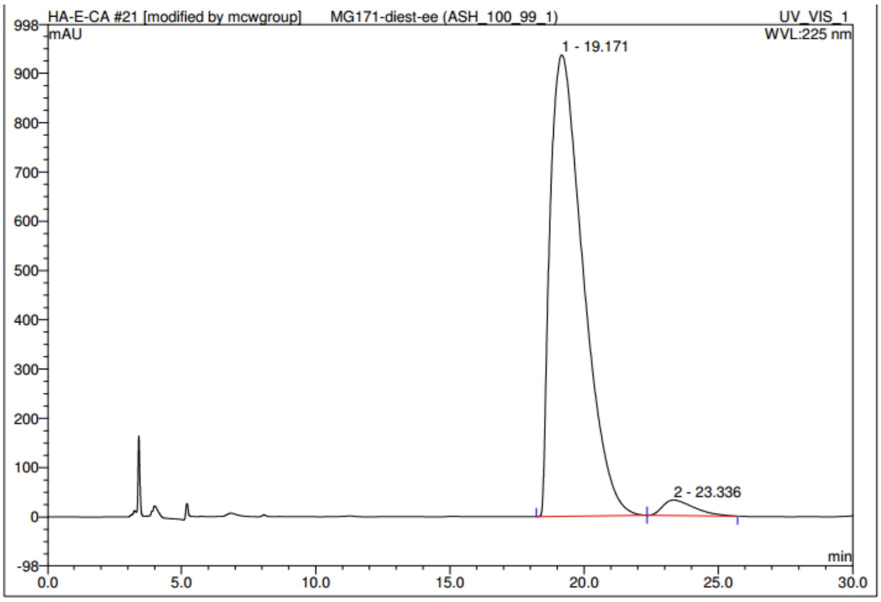

No.	Ret.Time min	Peak Name	Height mAU	Area mAU*min	Rel.Area %	Amount	Type
1	19.17	n.a.	935.594	1327.983	96.91	n.a.	BMb*
2	23.34	n.a.	31.418	42.353	3.09	n.a.	bMB*
Total:			967.012	1370.336	100.00	0.000	

(*S*)-1-Bromo-9-(4-methoxyphenyl)tetradecan-7-one (4t)

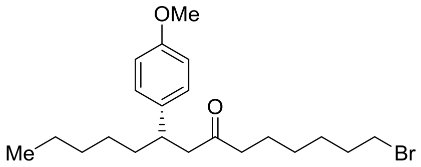

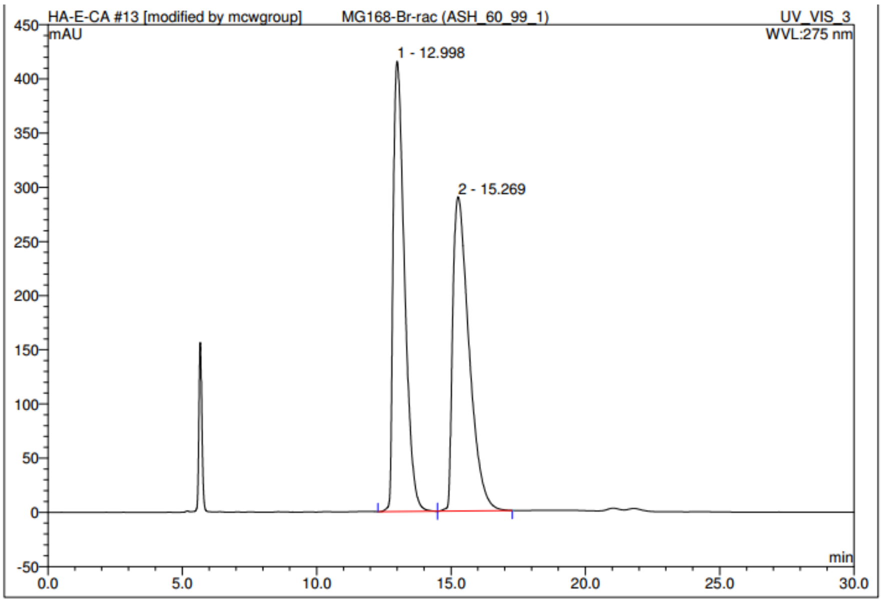

No.	Ret.Time min	Peak Name	Height mAU	Area mAU*min	Rel.Area %	Amount	Type
1	13.00	n.a.	415.582	203.710	50.00	n.a.	BM *
2	15.27	n.a.	290.045	203.730	50.00	n.a.	MB*
Total:			705.627	407.440	100.00	0.000	

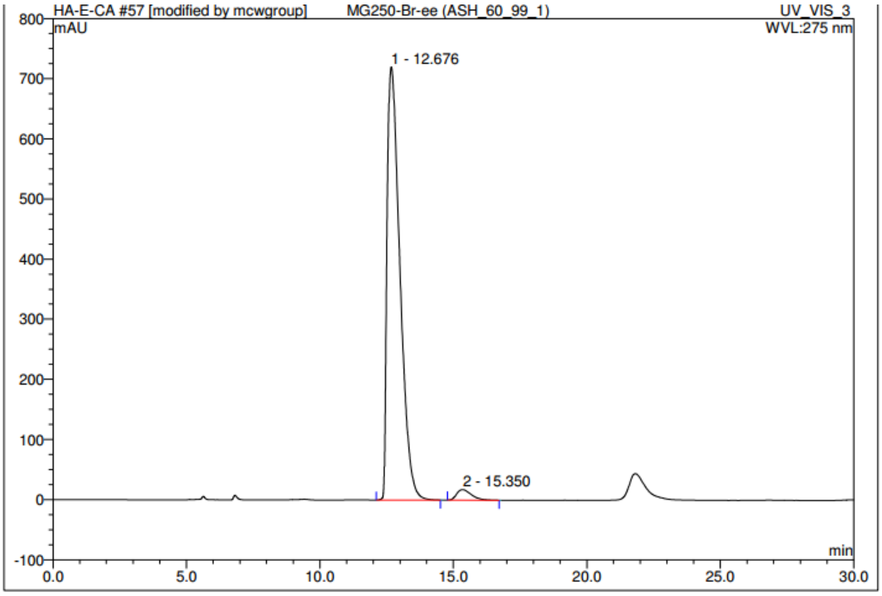

No.	Ret.Time min	Peak Name	Height mAU	Area mAU*min	Rel.Area %	Amount	Type
1	12.68	n.a.	720.752	403.034	97.33	n.a.	BMB*
2	15.35	n.a.	17.490	11.077	2.67	n.a.	BMB*
Total:			738.242	414.112	100.00	0.000	

(*S*)-1,1-Diethoxy-6-(4-methoxyphenyl)undecan-4-one (4u)

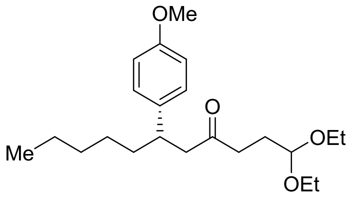

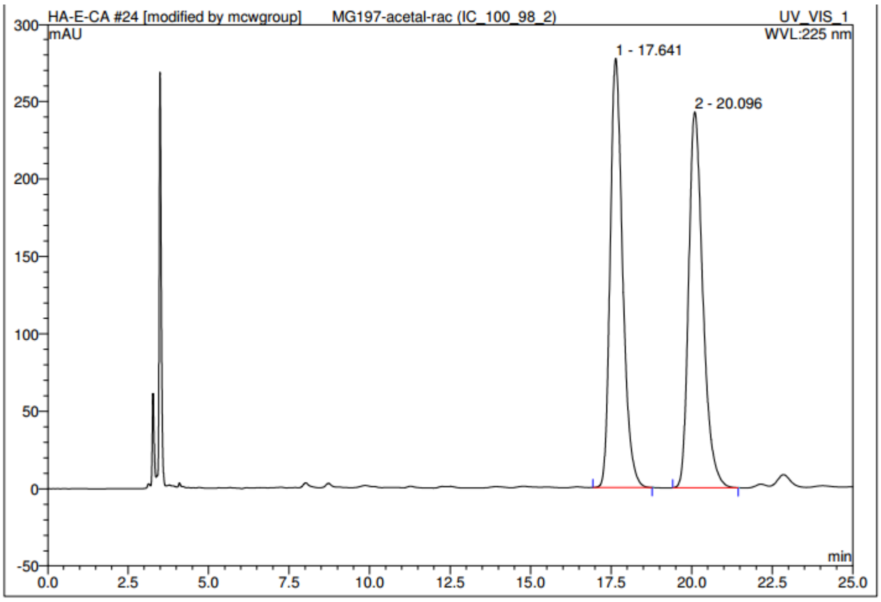

No.	Ret.Time min	Peak Name	Height mAU	Area mAU*min	Rel.Area %	Amount	Type
1	17.64	n.a.	276.966	123.960	49.91	n.a.	BMB*
2	20.10	n.a.	242.706	124.395	50.09	n.a.	BMB*
Total:			519.673	248.355	100.00	0.000	

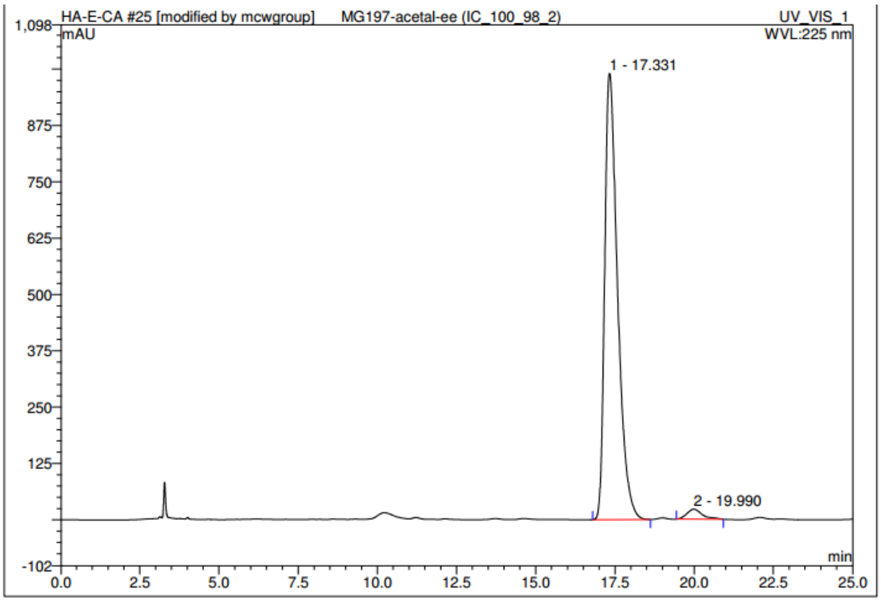

No.	Ret.Time min	Peak Name	Height mAU	Area mAU*min	Rel.Area %	Amount	Type
1	17.33	n.a.	989.937	467.002	97.41	n.a.	BMB*
2	19.99	n.a.	22.097	12.411	2.59	n.a.	BMB*
Total:			1012.034	479.413	100.00	0.000	

(*S*)-*N*,*N*-Di-Boc-1-amino-7-(4-methoxyphenyl)dodecan-5-one (4w)

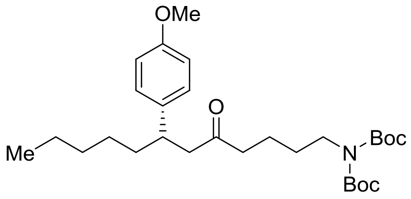

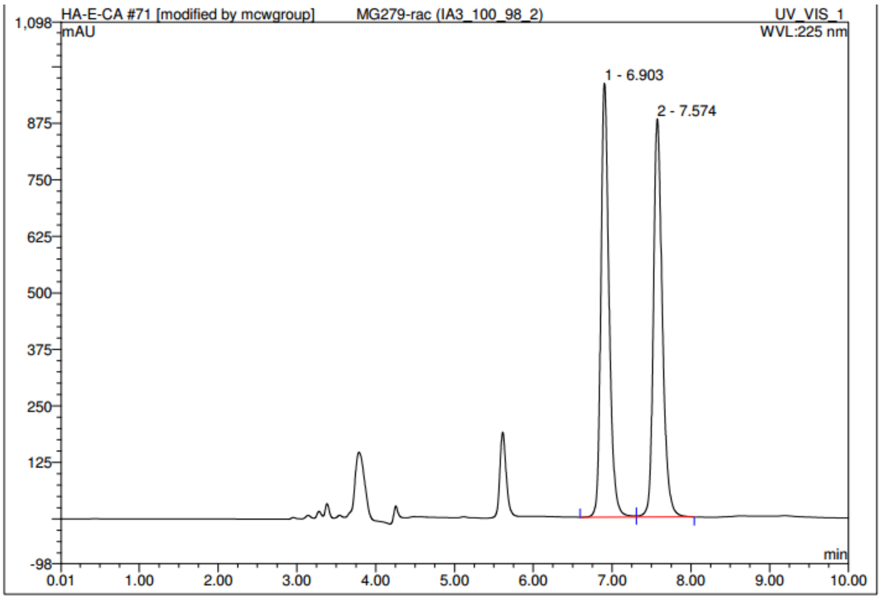

No.	Ret.Time min	Peak Name	Height mAU	Area mAU*min	Rel.Area %	Amount	Type
1	6.90	n.a.	959.372	115.157	49.78	n.a.	BM *
2	7.57	n.a.	880.562	116.164	50.22	n.a.	MB*
Total:			1839.934	231.320	100.00	0.000	

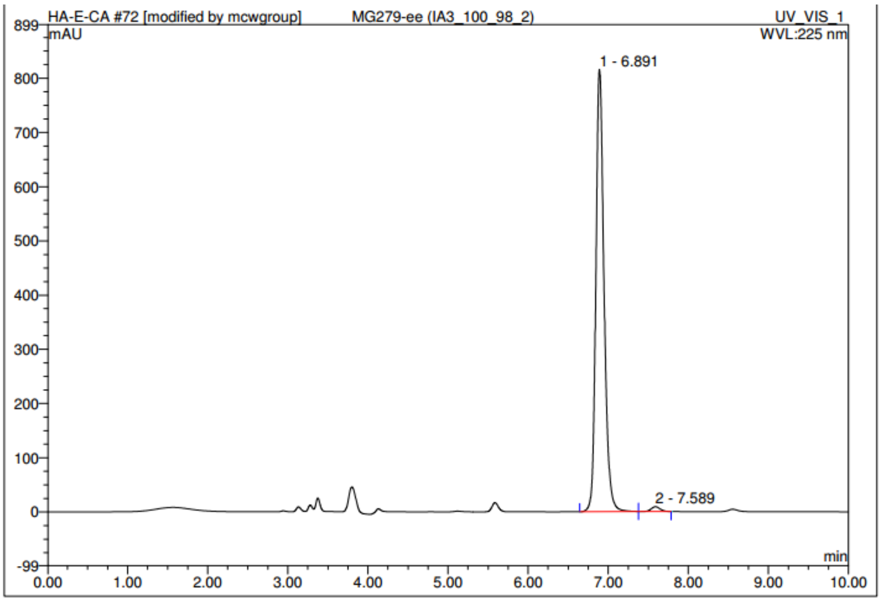

No.	Ret.Time min	Peak Name	Height mAU	Area mAU*min	Rel.Area %	Amount	Type
1	6.89	n.a.	815.626	96.268	98.86	n.a.	BMb*
2	7.59	n.a.	8.829	1.109	1.14	n.a.	bMB*
Total:			824.455	97.378	100.00	0.000	

(*S*)-9-(4-Methoxyphenyl)-7-oxotetradecyl acetate (4x)

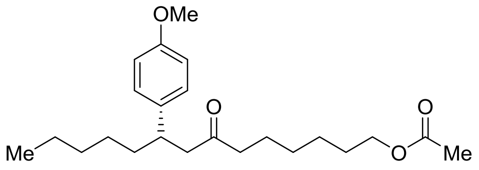

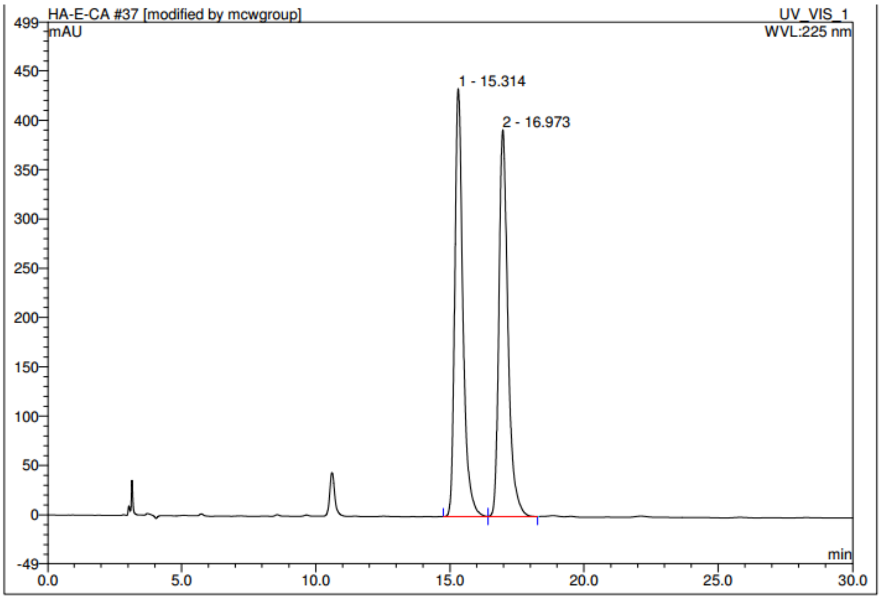

No.	Ret.Time min	Peak Name	Height mAU	Area mAU*min	Rel.Area %	Amount	Type
1	15.31	n.a.	433.674	153.848	50.09	n.a.	BM *
2	16.97	n.a.	391.849	153.266	49.91	n.a.	MB*
Total:			825.523	307.114	100.00	0.000	

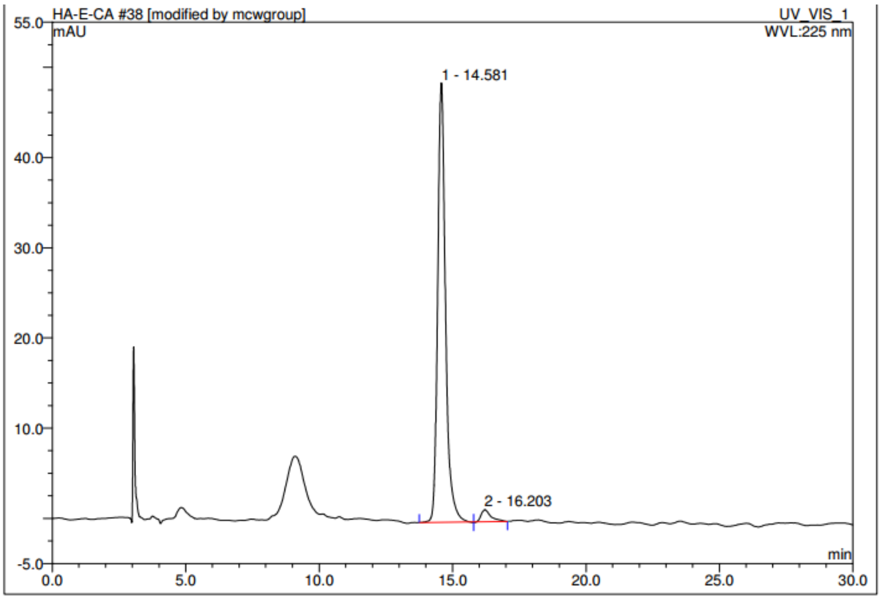

No.	Ret.Time min	Peak Name	Height mAU	Area mAU*min	Rel.Area %	Amount	Type
1	14.58	n.a.	48.721	16.644	96.67	n.a.	BM *
2	16.20	n.a.	1.348	0.573	3.33	n.a.	MB*
Total:			50.069	17.217	100.00	0.000	

(*S*)-2-[8-(4-Methoxyphenyl)-6-oxotridecyl]isoindoline-1,3-dione (4y)

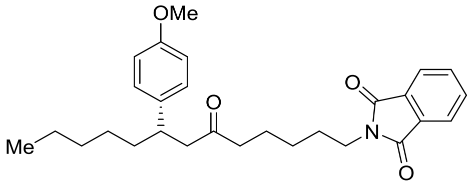

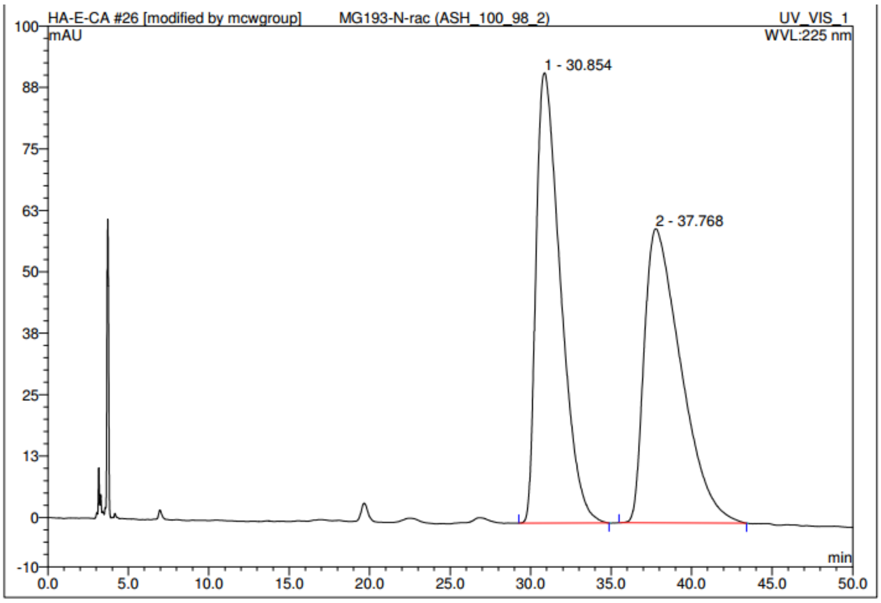

No.	Ret.Time min	Peak Name	Height mAU	Area mAU*min	Rel.Area %	Amount	Type
1	30.85	n.a.	91.591	161.065	50.49	n.a.	BMB*
2	37.77	n.a.	59.855	157.953	49.51	n.a.	BMB*
Total:			151.446	319.018	100.00	0.000	

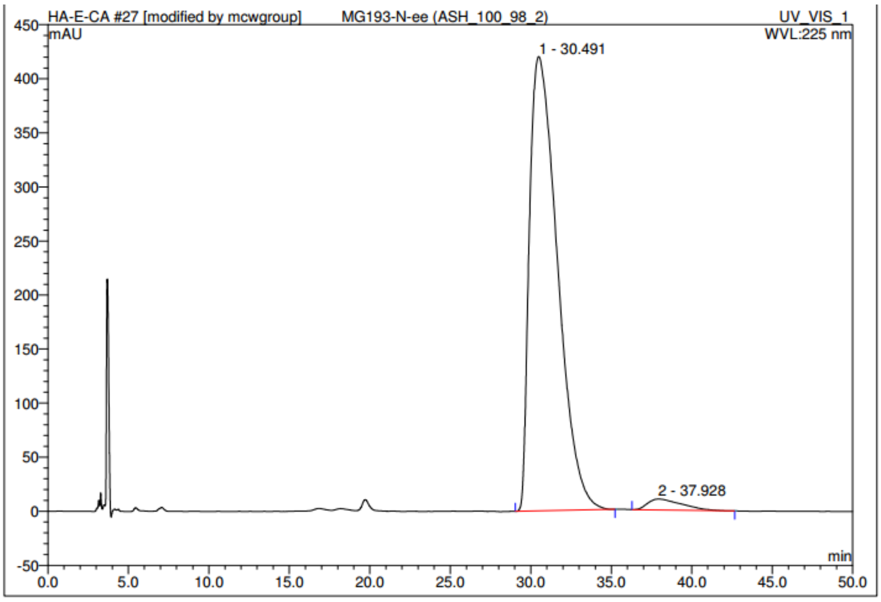

No.	Ret.Time min	Peak Name	Height mAU	Area mAU*min	Rel.Area %	Amount	Type
1	30.49	n.a.	420.202	845.069	97.16	n.a.	BMB*
2	37.93	n.a.	10.040	24.683	2.84	n.a.	BMB*
Total:			430.242	869.753	100.00	0.000	

(*R*)-7-(4-Methoxyphenyl)-8-methyl-1-phenylnonan-5-one (4aa)

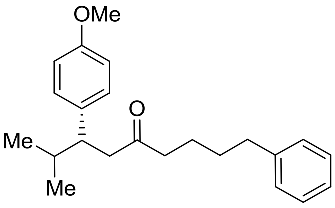

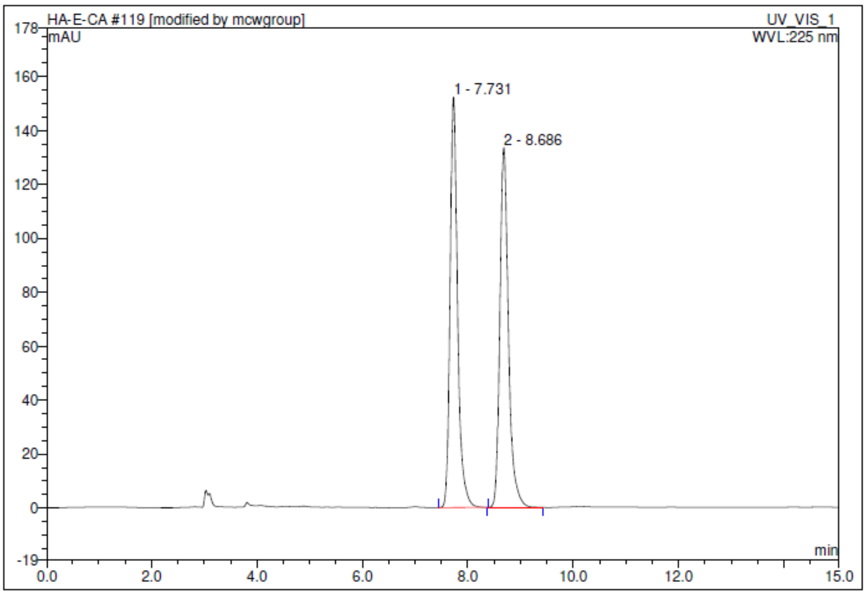

No.	Ret.Time min	Peak Name	Height mAU	Area mAU*min	Rel.Area %	Amount	Type
1	7.73	n.a.	152.178	25.275	50.07	n.a.	BMB
2	8.69	n.a.	133.453	25.208	49.93	n.a.	BMB
Total:			285.631	50.483	100.00	0.000	

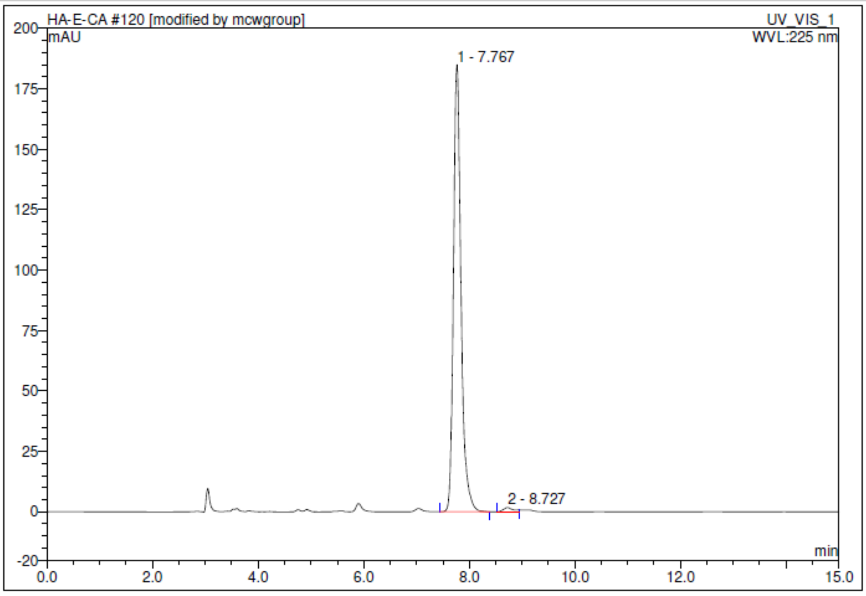

No.	Ret.Time min	Peak Name	Height mAU	Area mAU*min	Rel.Area %	Amount	Type
1	7.77	n.a.	184.914	30.648	98.80	n.a.	BMB*
2	8.73	n.a.	1.723	0.372	1.20	n.a.	BM *
Total:			186.637	31.020	100.00	0.000	

(*S*)-9-(4-Methoxyphenyl)-11,11-dimethyldodecane-2,7-dione (4ab)

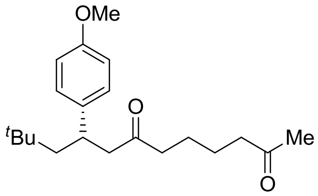

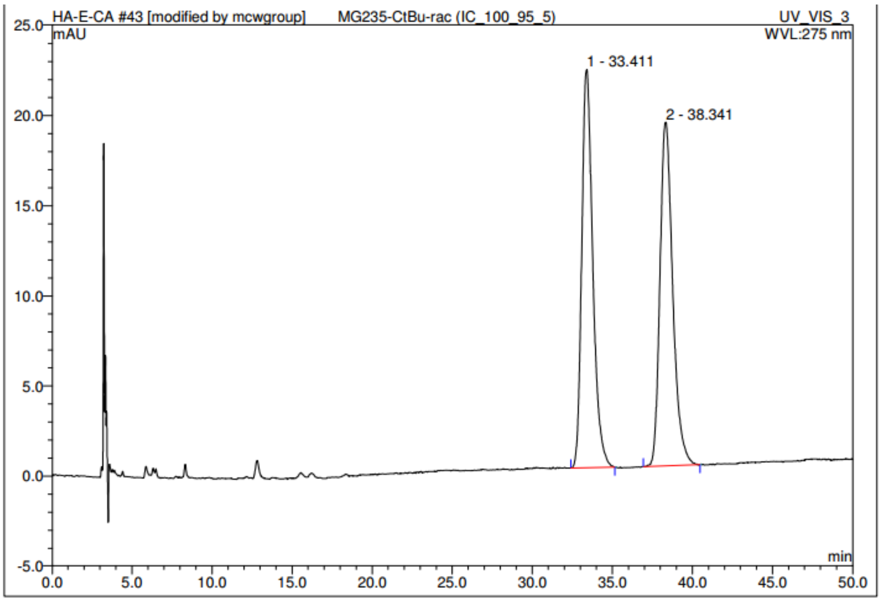

No.	Ret.Time min	Peak Name	Height mAU	Area mAU*min	Rel.Area %	Amount	Type
1	33.41	n.a.	22.086	17.560	49.94	n.a.	BMB*
2	38.34	n.a.	19.057	17.603	50.06	n.a.	BMB*
Total:			41.142	35.163	100.00	0.000	

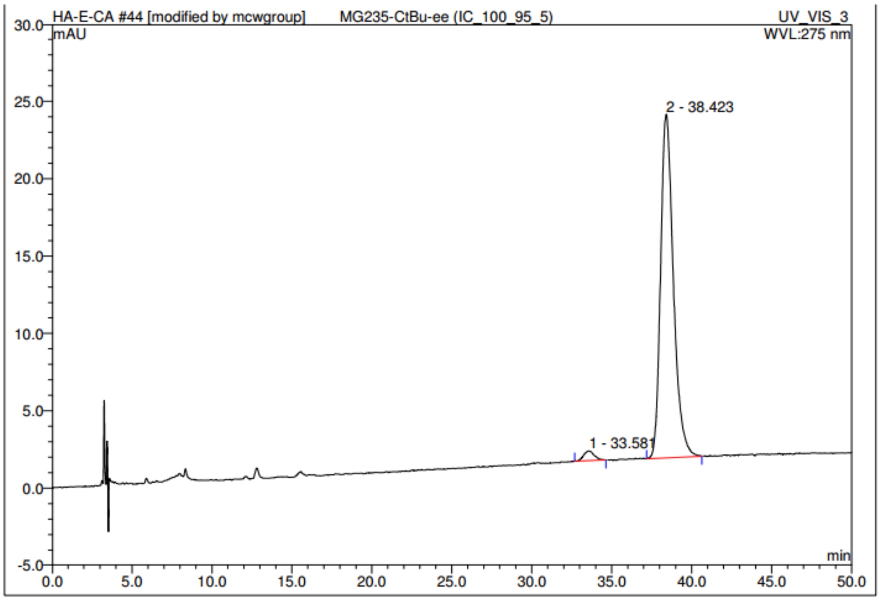

No.	Ret.Time min	Peak Name	Height mAU	Area mAU*min	Rel.Area %	Amount	Type
1	33.58	n.a.	0.621	0.480	2.28	n.a.	BMB*
2	38.42	n.a.	22.206	20.577	97.72	n.a.	BMB*
Total:			22.827	21.057	100.00	0.000	

(*R*)-1-Cyclohexyl-1-(4-methoxyphenyl)-7-phenylheptan-3-one (4ac)

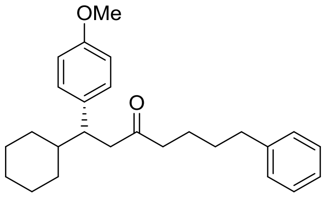

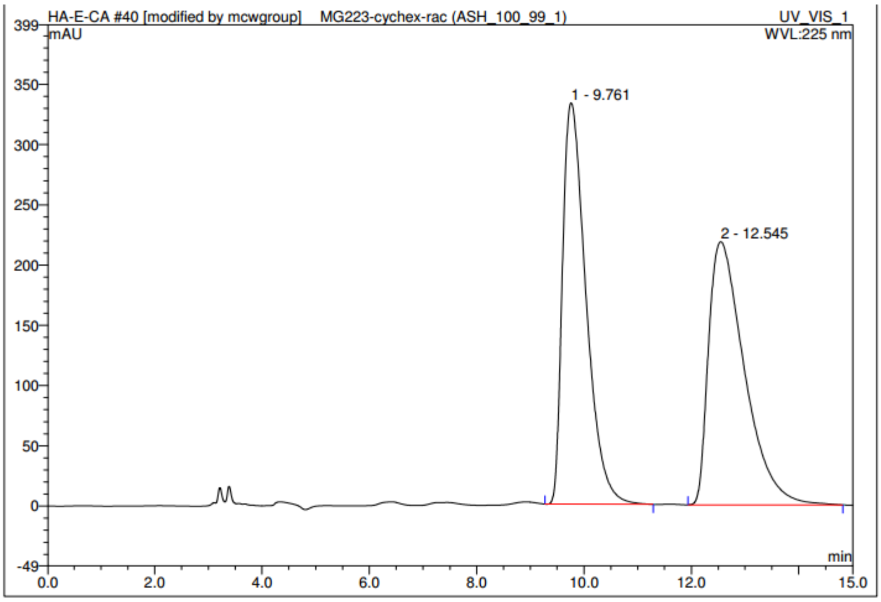

No.	Ret.Time min	Peak Name	Height mAU	Area mAU*min	Rel.Area %	Amount	Type
1	9.76	n.a.	332.949	168.600	49.92	n.a.	BMB*
2	12.54	n.a.	218.396	169.162	50.08	n.a.	BMB*
Total:			551.346	337.762	100.00	0.000	

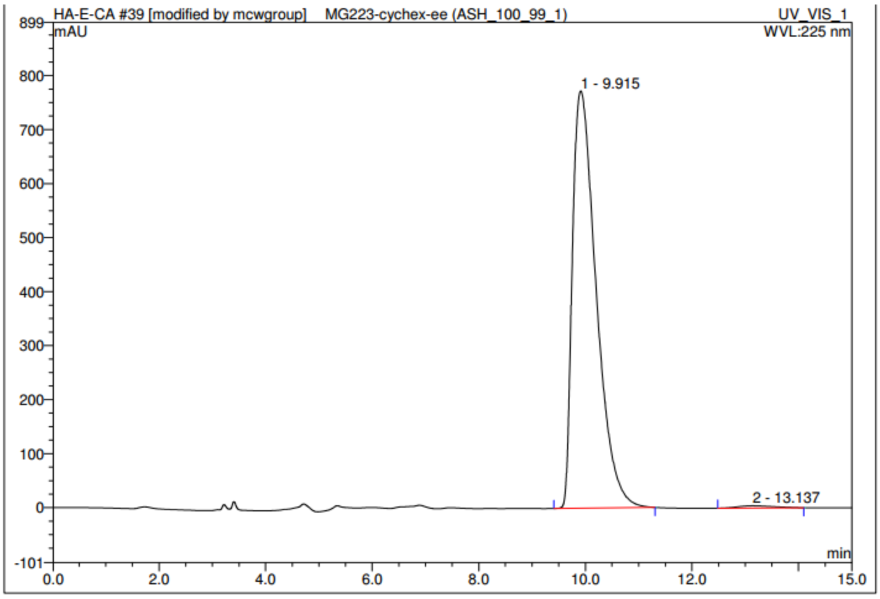

No.	Ret.Time min	Peak Name	Height mAU	Area mAU*min	Rel.Area %	Amount	Type
1	9.92	n.a.	772.329	398.603	99.23	n.a.	BMB*
2	13.14	n.a.	4.050	3.112	0.77	n.a.	BMB*
Total:			776.380	401.715	100.00	0.000	

(*R*)-1-Cyclopropyl-1-(4-methoxyphenyl)-7-phenylheptan-3-one (4ad)

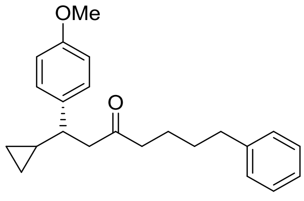

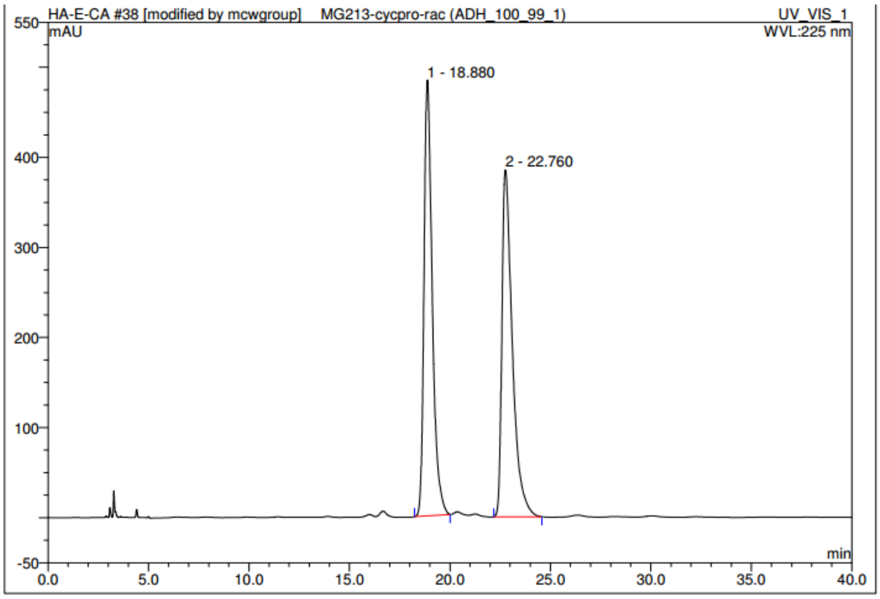

No.	Ret.Time min	Peak Name	Height mAU	Area mAU*min	Rel.Area %	Amount	Type
1	18.88	n.a.	483.876	230.500	49.93	n.a.	BMB*
2	22.76	n.a.	385.082	231.144	50.07	n.a.	BMB*
Total:			868.958	461.644	100.00	0.000	

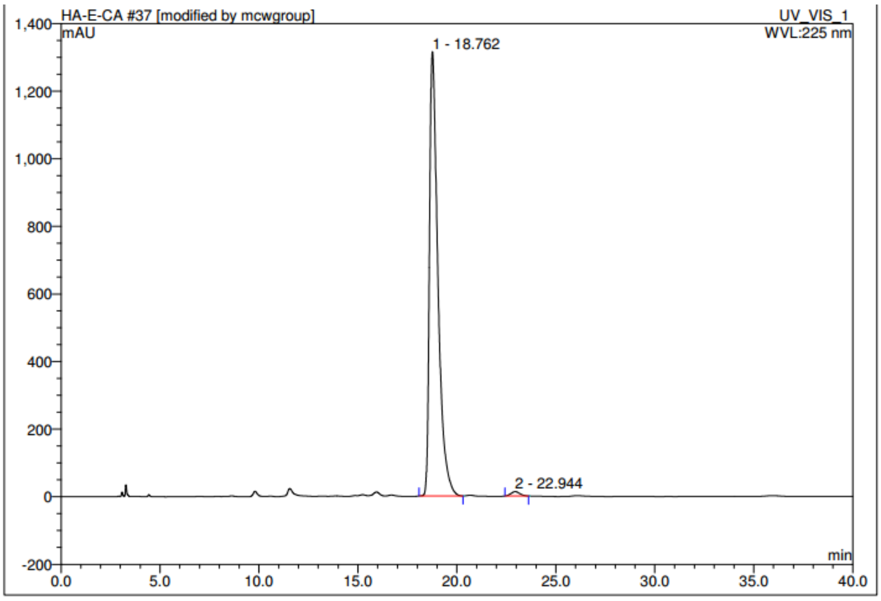

No.	Ret.Time min	Peak Name	Height mAU	Area mAU*min	Rel.Area %	Amount	Type
1	18.76	n.a.	1314.530	650.440	98.98	n.a.	BMB*
2	22.94	n.a.	12.933	6.719	1.02	n.a.	BMB*
Total:			1327.464	657.158	100.00	0.000	

(*S*)-3-(4-Methoxyphenyl)-1,9-diphenylnonan-5-one (4ae)

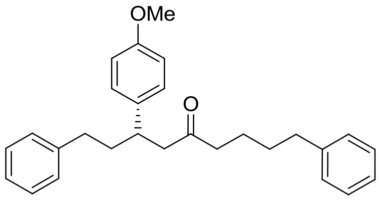

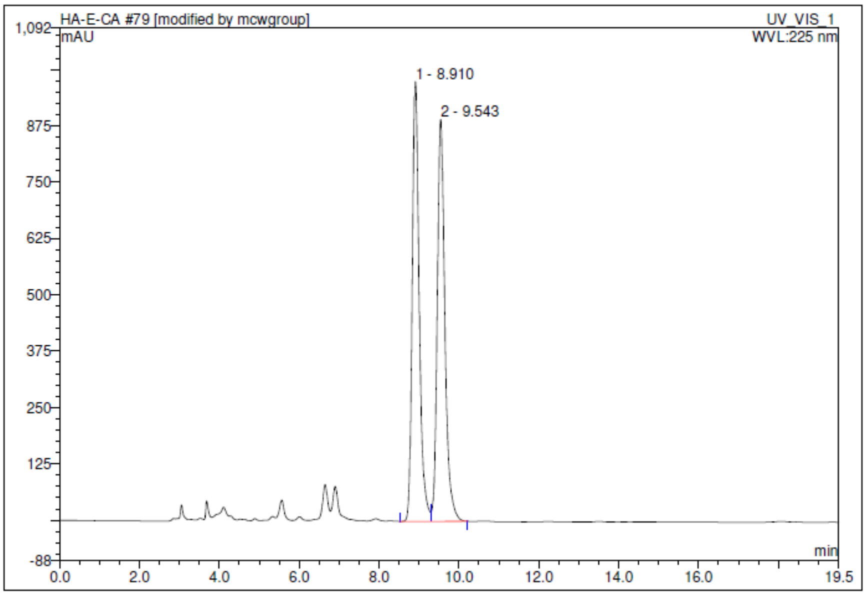

No.	Ret.Time min	Peak Name	Height mAU	Area mAU*min	Rel.Area %	Amount	Type
1	8.91	n.a.	975.757	198.272	50.00	n.a.	BM *
2	9.54	n.a.	891.455	198.263	50.00	n.a.	MB*
Total:			1867.212	396.535	100.00	0.000	

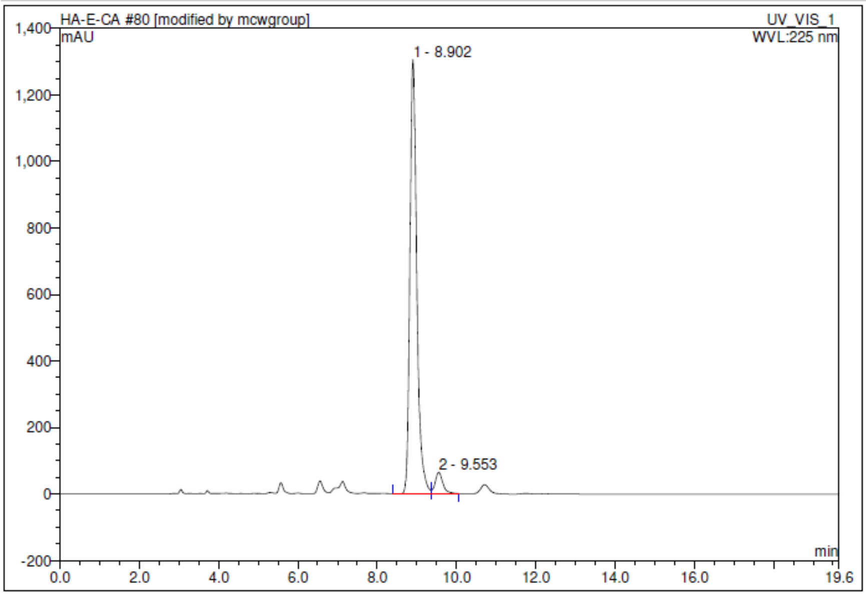

No.	Ret.Time min	Peak Name	Height mAU	Area mAU*min	Rel.Area %	Amount	Type
1	8.90	n.a.	1304.844	263.594	94.80	n.a.	BM *
2	9.55	n.a.	64.303	14.445	5.20	n.a.	MB*
Total:			1369.147	278.039	100.00	0.000	

(*R*)-1-(4-Methoxyphenyl)-1,7-diphenylheptan-3-one (4af)

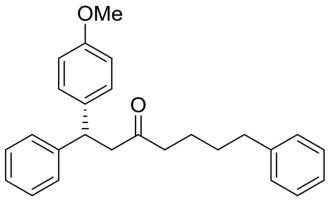

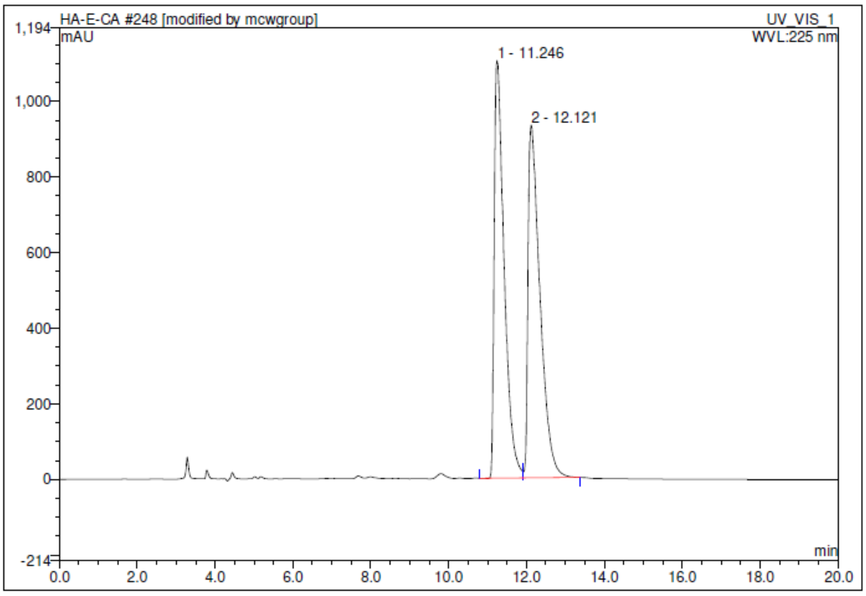

No.	Ret.Time min	Peak Name	Height mAU	Area mAU*min	Rel.Area %	Amount	Type
1	11.25	n.a.	1105.316	326.430	49.50	n.a.	BM *
2	12.12	n.a.	933.358	332.974	50.50	n.a.	MB*
Total:			2038.674	659.404	100.00	0.000	

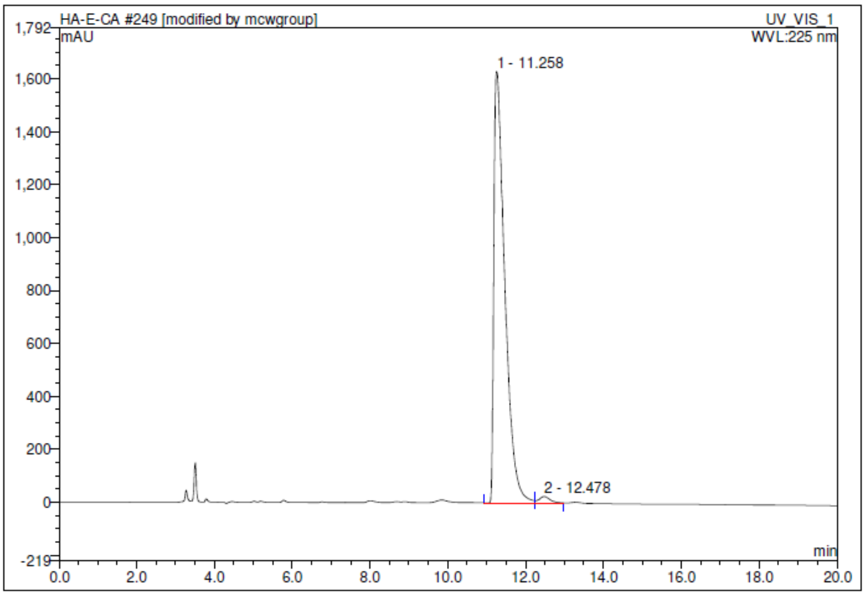

No.	Ret.Time min	Peak Name	Height mAU	Area mAU*min	Rel.Area %	Amount	Type
1	11.26	n.a.	1630.666	532.843	98.57	n.a.	BM *
2	12.48	n.a.	24.118	7.722	1.43	n.a.	MB*
Total:			1654.785	540.565	100.00	0.000	

(*S*)-1-(4-Bromophenyl)-1-(4-methoxyphenyl)-7-phenylheptan-3-one (4ag)

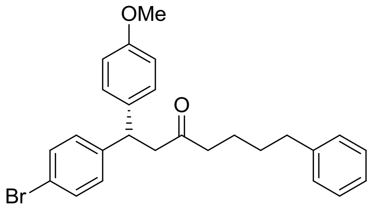

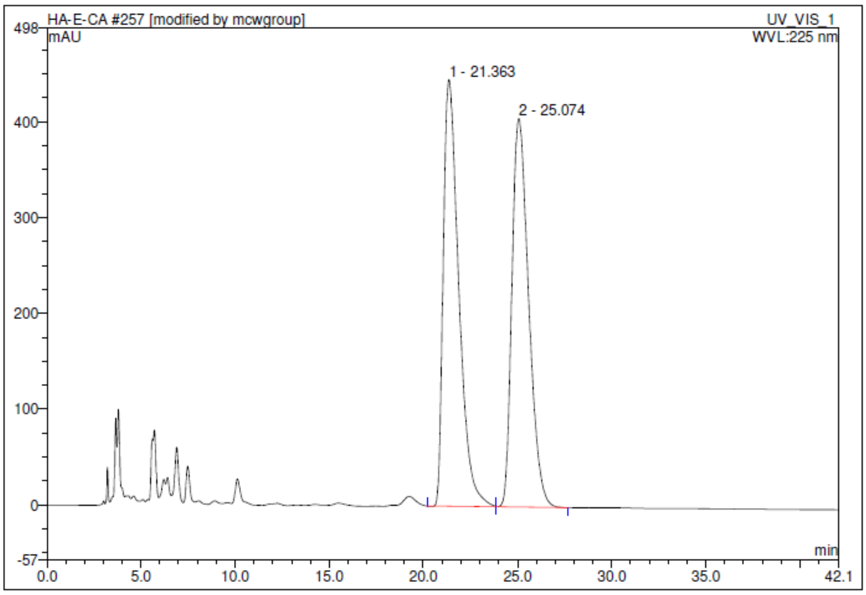

No.	Ret.Time min	Peak Name	Height mAU	Area mAU*min	Rel.Area %	Amount	Type
1	21.36	n.a.	445.586	424.192	50.39	n.a.	BM *
2	25.07	n.a.	405.706	417.655	49.61	n.a.	MB*
Total:			851.292	841.847	100.00	0.000	

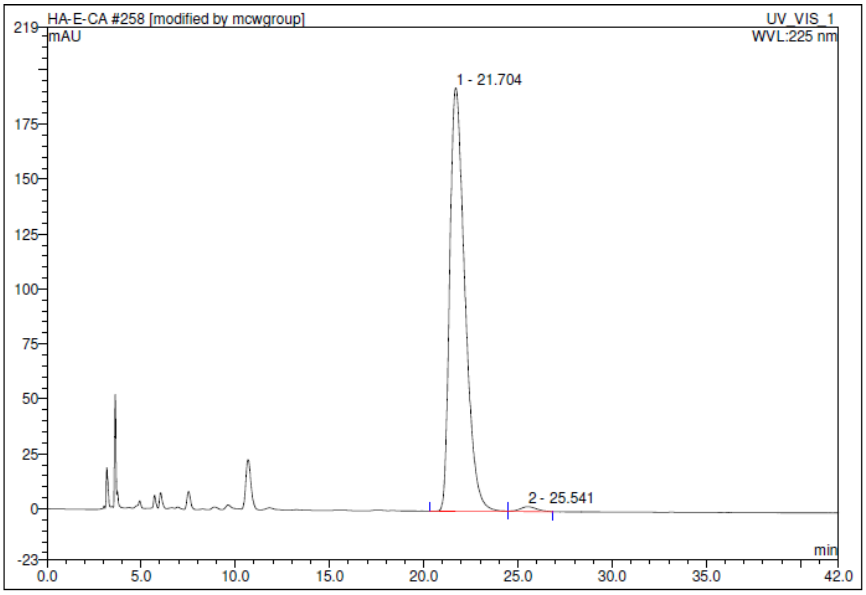

No.	Ret.Time min	Peak Name	Height mAU	Area mAU*min	Rel.Area %	Amount	Type
1	21.70	n.a.	192.681	179.804	98.78	n.a.	BM *
2	25.54	n.a.	2.177	2.228	1.22	n.a.	MB*
Total:			194.858	182.032	100.00	0.000	

(*R*)-1-(4-Methoxyphenyl)-1-(4-nitrophenyl)-7-phenylheptan-3-one (4ah)

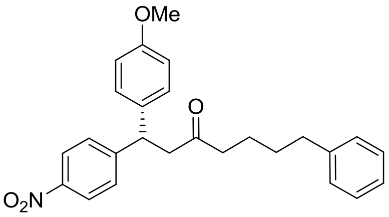

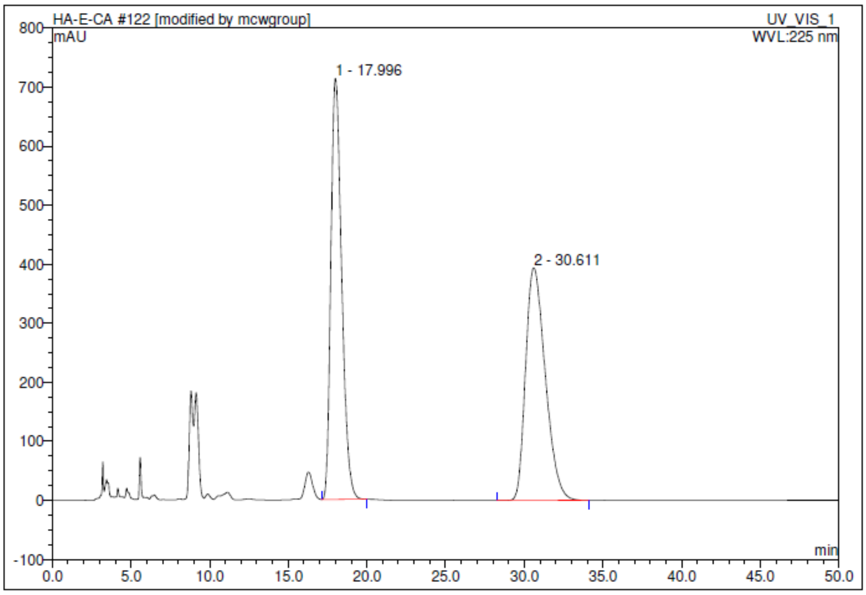

No.	Ret.Time min	Peak Name	Height mAU	Area mAU*min	Rel.Area %	Amount	Type
1	18.00	n.a.	712.985	568.408	49.89	n.a.	BMB*
2	30.61	n.a.	394.002	570.856	50.11	n.a.	BMB*
Total:			1106.987	1139.264	100.00	0.000	

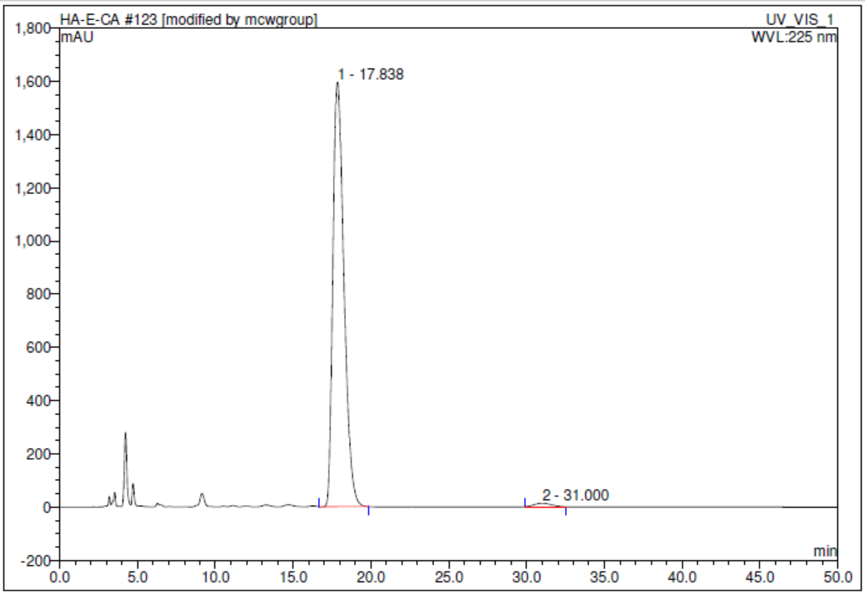

No.	Ret.Time min	Peak Name	Height mAU	Area mAU*min	Rel.Area %	Amount	Type
1	17.84	n.a.	1596.283	1315.789	98.74	n.a.	BMB*
2	31.00	n.a.	13.141	16.797	1.26	n.a.	BMB*
Total:			1609.424	1332.585	100.00	0.000	

(*S*)-1-(Benzyloxy)-3-(4-methoxyphenyl)-9-phenylnonan-5-one (4ai)

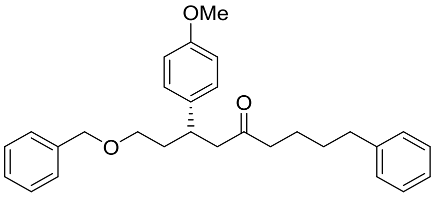

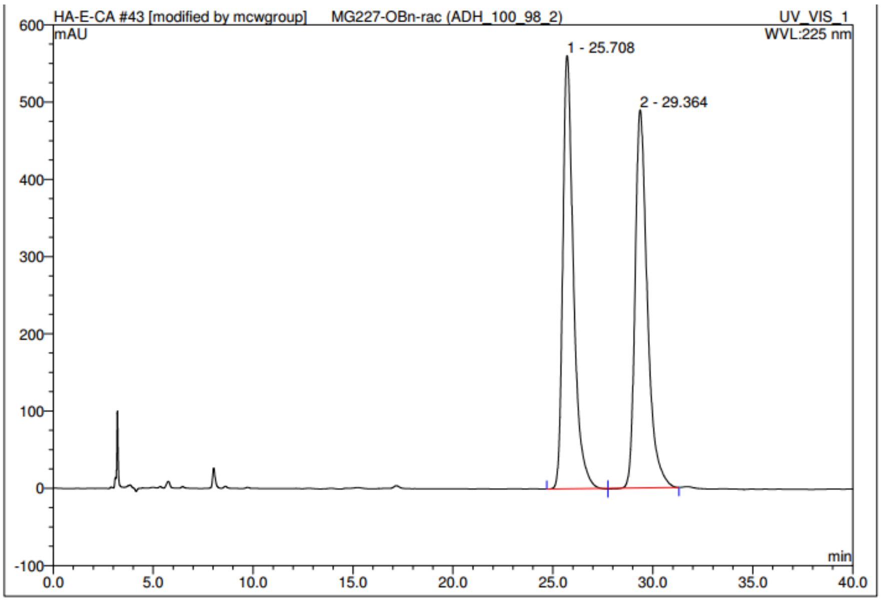

No.	Ret.Time min	Peak Name	Height mAU	Area mAU*min	Rel.Area %	Amount	Type
1	25.71	n.a.	560.682	347.303	50.00	n.a.	BM *
2	29.36	n.a.	489.659	347.248	50.00	n.a.	MB*
Total:			1050.340	694.550	100.00	0.000	

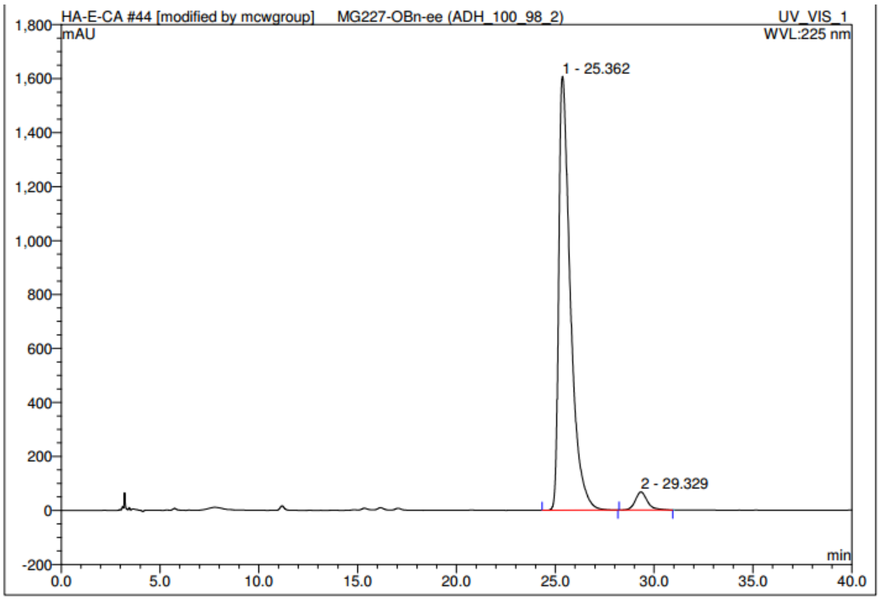

No.	Ret.Time min	Peak Name	Height mAU	Area mAU*min	Rel.Area %	Amount	Type
1	25.36	n.a.	1607.972	1096.317	95.83	n.a.	BMB*
2	29.33	n.a.	66.766	47.760	4.17	n.a.	BMB*
Total:			1674.738	1144.077	100.00	0.000	

(*S*)-5-(4-Methoxyphenyl)-7-oxo-11-phenylundecyl acetate (4aj)

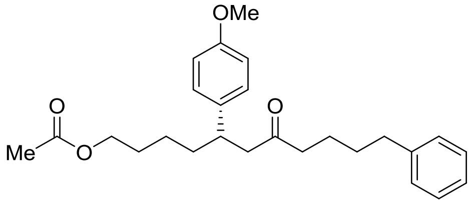

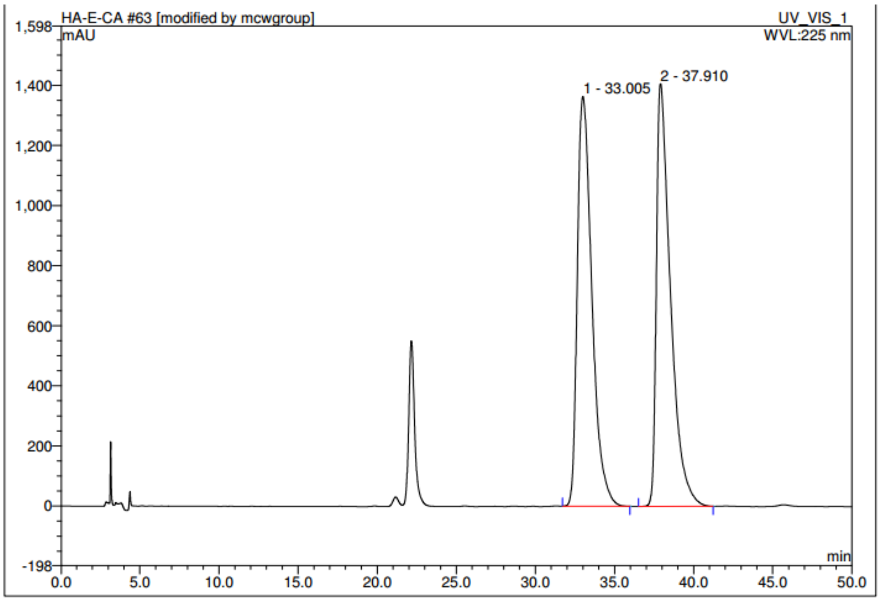

No.	Ret.Time min	Peak Name	Height mAU	Area mAU*min	Rel.Area %	Amount	Type
1	33.01	n.a.	1363.065	1454.878	50.00	n.a.	BMB*
2	37.91	n.a.	1406.537	1454.913	50.00	n.a.	BMB*
Total:			2769.602	2909.791	100.00	0.000	

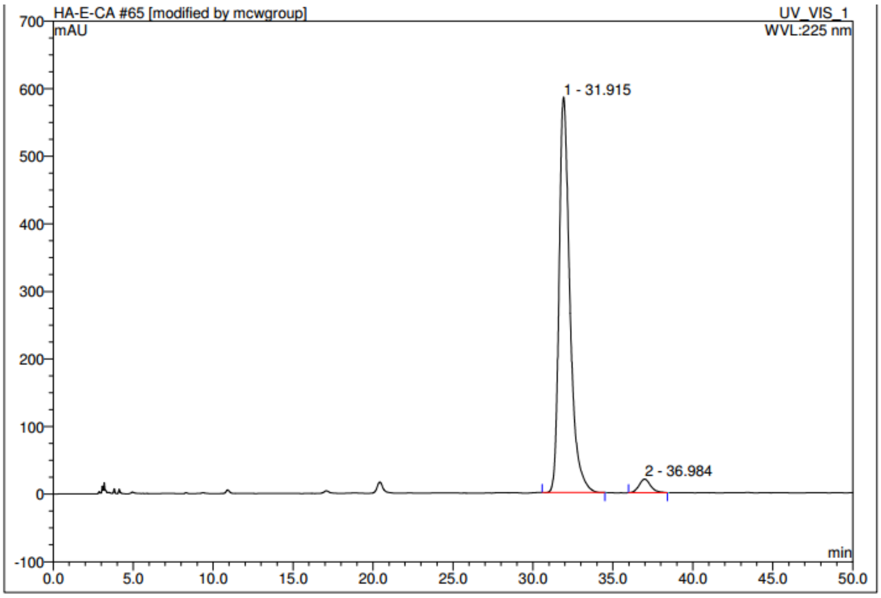

No.	Ret.Time min	Peak Name	Height mAU	Area mAU*min	Rel.Area %	Amount	Type
1	31.92	n.a.	585.143	458.487	96.45	n.a.	BMB*
2	36.98	n.a.	20.112	16.877	3.55	n.a.	BMB*
Total:			605.254	475.364	100.00	0.000	

(*S*)-2-[4-(4-Methoxyphenyl)-6-oxo-10-phenyldecyl]isoindoline-1,3-dione (4ak)

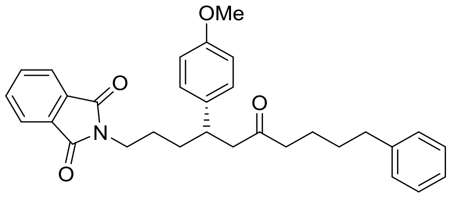

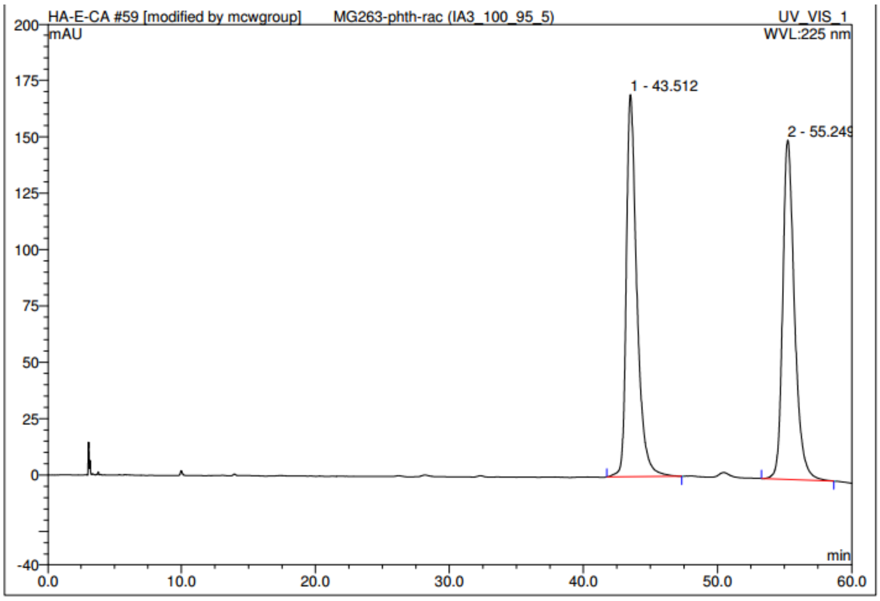

No.	Ret.Time min	Peak Name	Height mAU	Area mAU*min	Rel.Area %	Amount	Type
1	43.51	n.a.	169.478	154.902	50.17	n.a.	BMB*
2	55.25	n.a.	150.468	153.832	49.83	n.a.	BMB*
Total:			319.946	308.734	100.00	0.000	

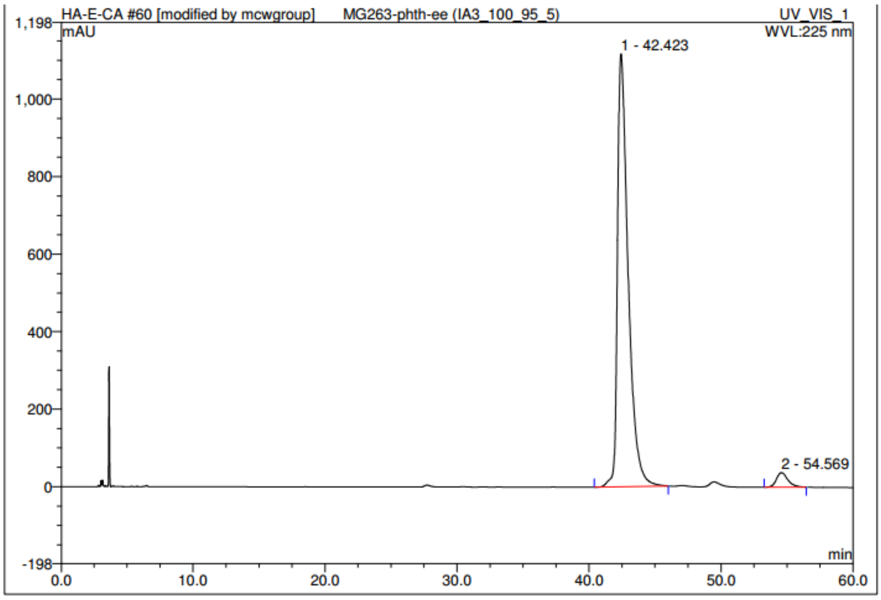

No.	Ret.Time min	Peak Name	Height mAU	Area mAU*min	Rel.Area %	Amount	Type
1	42.42	n.a.	1116.867	1047.424	96.53	n.a.	BMB*
2	54.57	n.a.	37.588	37.667	3.47	n.a.	BMB*
Total:			1154.456	1085.090	100.00	0.000	

Methyl 4-[(7*S*,9*S*)-9,13-dimethyl-5-oxo-1-phenyltetradec-12-en-7-yl]benzoate (4al)

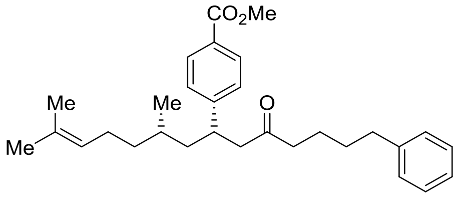

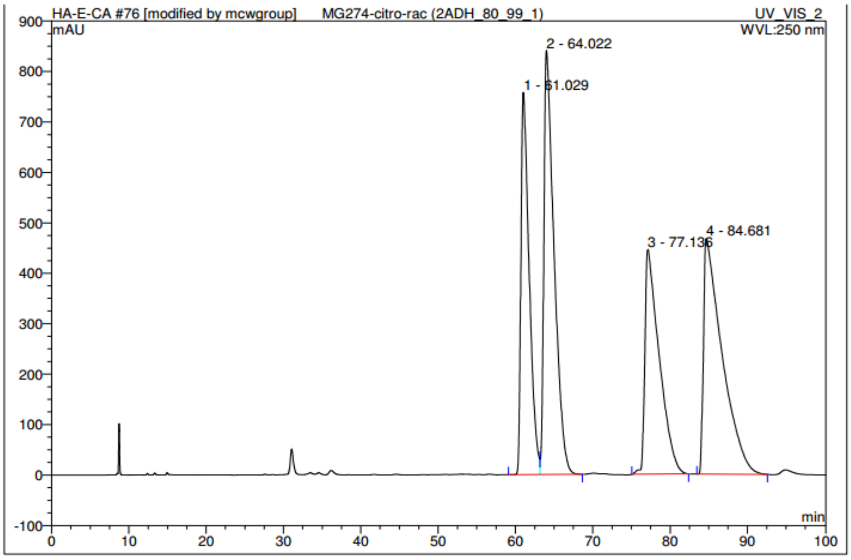

No.	Ret.Time min	Peak Name	Height mAU	Area mAU*min	Rel.Area %	Amount	Type
1	61.03	n.a.	757.950	939.148	20.96	n.a.	BM *
2	64.02	n.a.	840.029	1284.433	28.67	n.a.	MB*
3	77.14	n.a.	445.621	956.157	21.34	n.a.	BMB*
4	84.68	n.a.	467.339	1300.434	29.03	n.a.	BMB*
Total:			2510.938	4480.171	100.00	0.000	

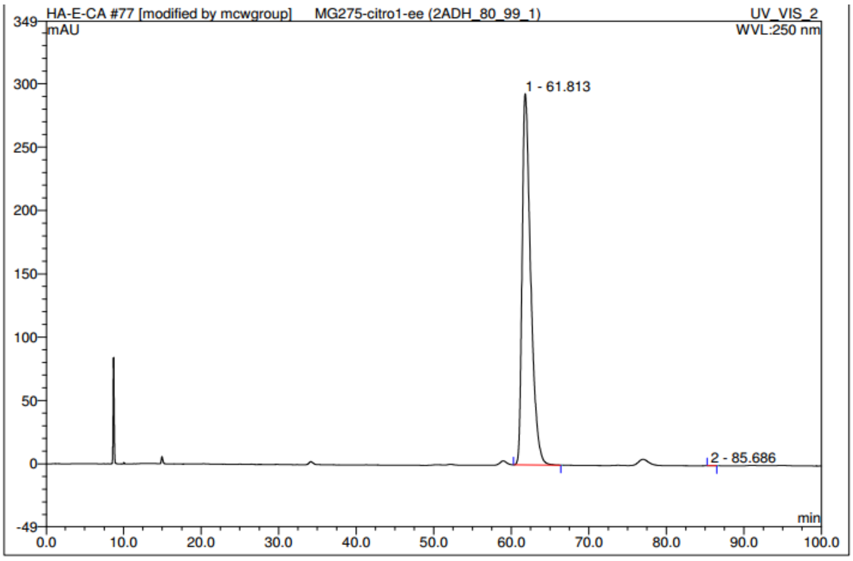

No.	Ret.Time min	Peak Name	Height mAU	Area mAU*min	Rel.Area %	Amount	Type
1	61.81	n.a.	293.030	375.878	99.99	n.a.	BMB*
2	85.69	n.a.	0.077	0.051	0.01	n.a.	BMB*
Total:			293.106	375.930	100.00	0.000	

Methyl 4-[(7*R*,9*S*)-9,13-dimethyl-5-oxo-1-phenyltetradec-12-en-7-yl]benzoate (4am)

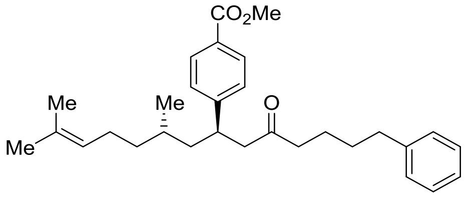

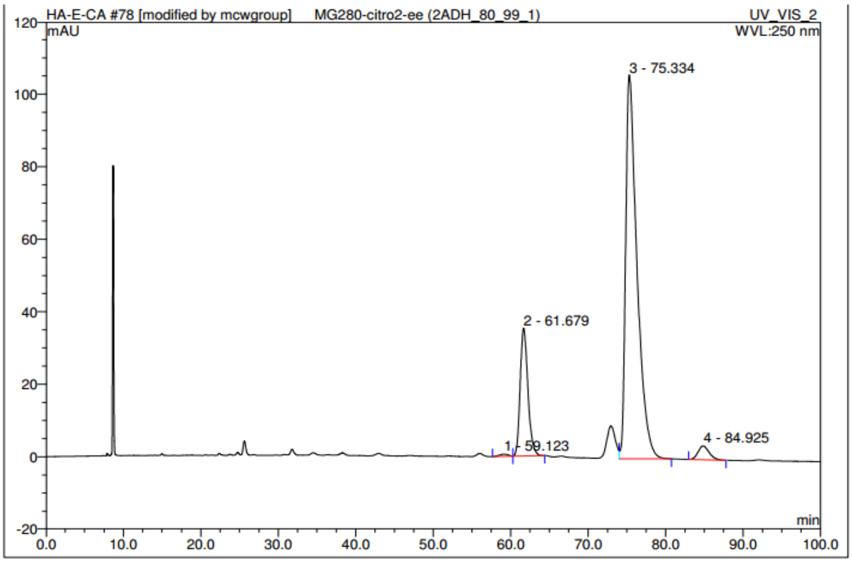

No.	Ret.Time min	Peak Name	Height mAU	Area mAU*min	Rel.Area %	Amount	Type
1	59.12	n.a.	0.600	0.805	0.35	n.a.	BMb*
2	61.68	n.a.	35.236	40.635	17.90	n.a.	bMB*
3	75.33	n.a.	105.817	179.260	78.96	n.a.	MB*
4	84.92	n.a.	3.794	6.314	2.78	n.a.	BMB*
Total:			145.448	227.014	100.00	0.000	

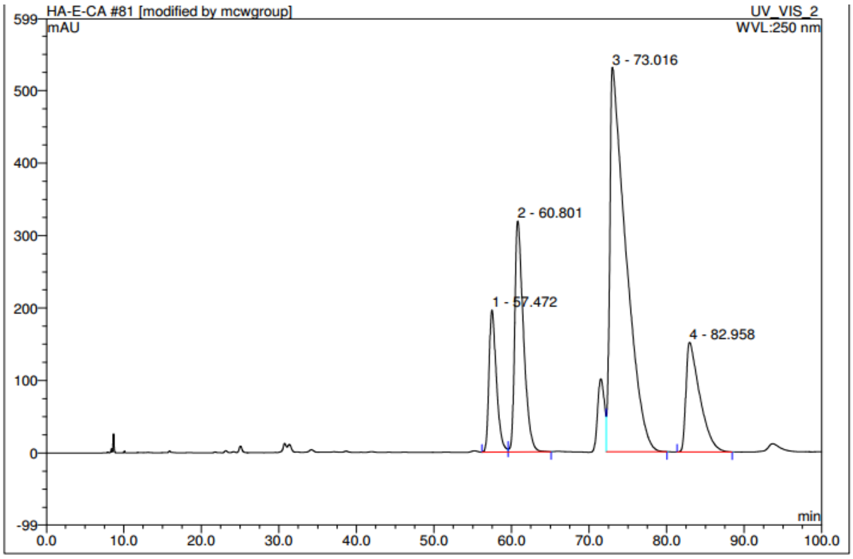

No.	Ret.Time min	Peak Name	Height mAU	Area mAU*min	Rel.Area %	Amount	Type
1	57.47	n.a.	196.020	218.182	9.79	n.a.	BM *
2	60.80	n.a.	318.688	415.366	18.63	n.a.	MB*
3	73.02	n.a.	530.533	1282.684	57.54	n.a.	MB*
4	82.96	n.a.	151.186	312.851	14.03	n.a.	BMB*
Total:			1196.427	2229.084	100.00	0.000	

(co-injection)

(*S*,*E*)-7-(4-Methoxyphenyl)-2,2-dimethyldodec-3-en-5-one (5a)

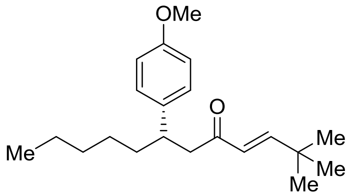

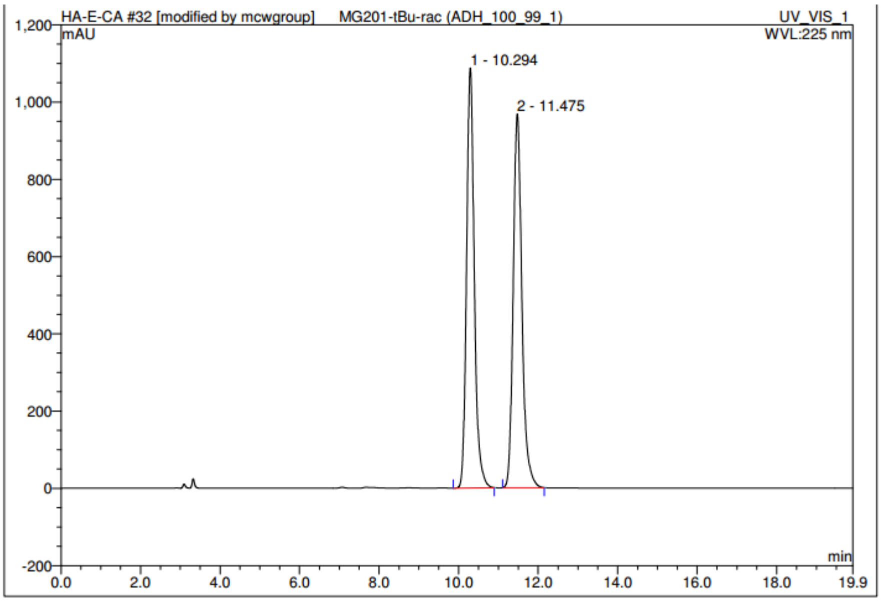

No.	Ret.Time min	Peak Name	Height mAU	Area mAU*min	Rel.Area %	Amount	Type
1	10.29	n.a.	1087.713	244.837	50.00	n.a.	BMB*
2	11.47	n.a.	967.634	244.866	50.00	n.a.	BMB*
Total:			2055.347	489.703	100.00	0.000	

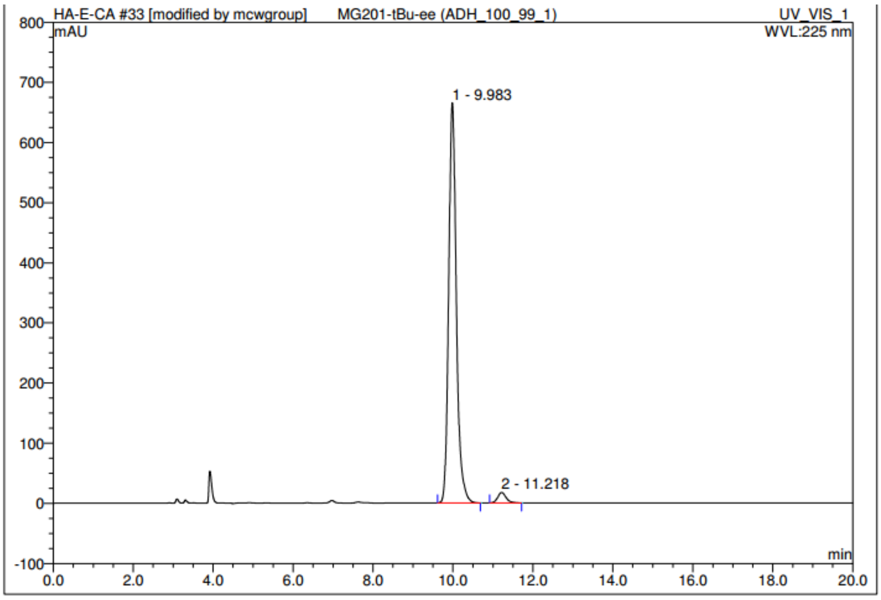

No.	Ret.Time min	Peak Name	Height mAU	Area mAU*min	Rel.Area %	Amount	Type
1	9.98	n.a.	665.682	147.569	97.21	n.a.	BMB*
2	11.22	n.a.	17.463	4.241	2.79	n.a.	BMB*
Total:			683.145	151.810	100.00	0.000	

(*S*,*E*)-8-(4-Methoxyphenyl)-5-propyltridec-4-en-6-one (5b)

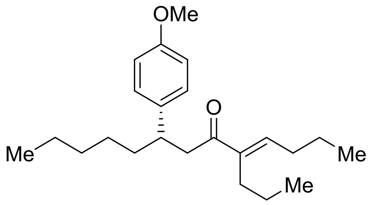

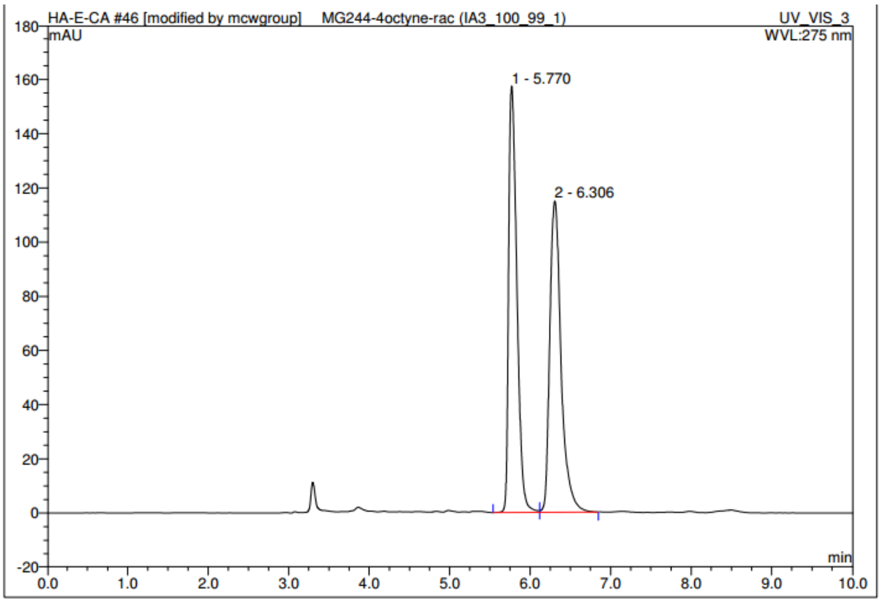

No.	Ret.Time min	Peak Name	Height mAU	Area mAU*min	Rel.Area %	Amount	Type
1	5.77	n.a.	157.314	18.708	49.99	n.a.	BM *
2	6.31	n.a.	114.835	18.718	50.01	n.a.	MB*
Total:			272.150	37.426	100.00	0.000	

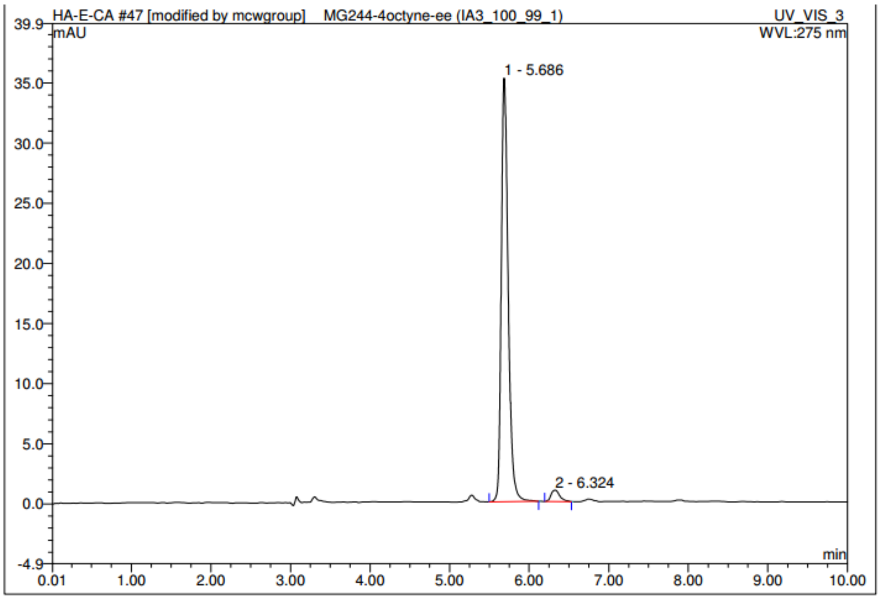

No.	Ret.Time min	Peak Name	Height mAU	Area mAU*min	Rel.Area %	Amount	Type
1	5.69	n.a.	35.201	3.537	96.72	n.a.	BMB*
2	6.32	n.a.	0.937	0.120	3.28	n.a.	BMB*
Total:			36.137	3.657	100.00	0.000	

Dimethyl 4,4'-[(1*R*,5*S*)-1-cyclohexyl-3-oxodecane-1,5-diyl]dibenzoate (5c)

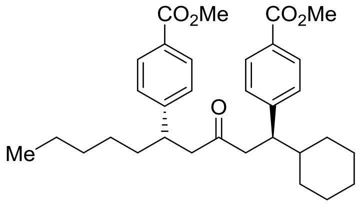

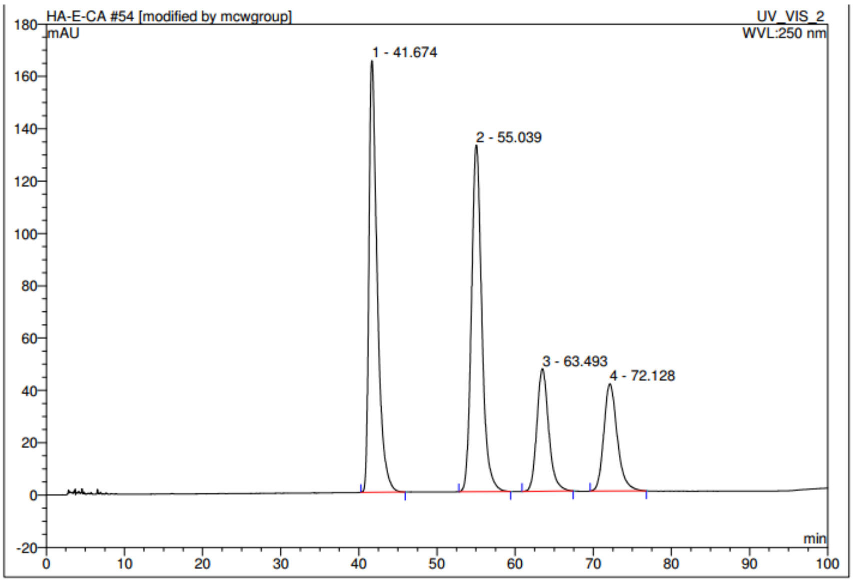

No.	Ret.Time min	Peak Name	Height mAU	Area mAU*min	Rel.Area %	Amount	Type
1	41.67	n.a.	165.019	203.294	35.65	n.a.	BMB*
2	55.04	n.a.	132.428	203.198	35.64	n.a.	BMB*
3	63.49	n.a.	46.840	82.053	14.39	n.a.	BMB*
4	72.13	n.a.	40.934	81.671	14.32	n.a.	BMB*
Total:			385.222	570.216	100.00	0.000	

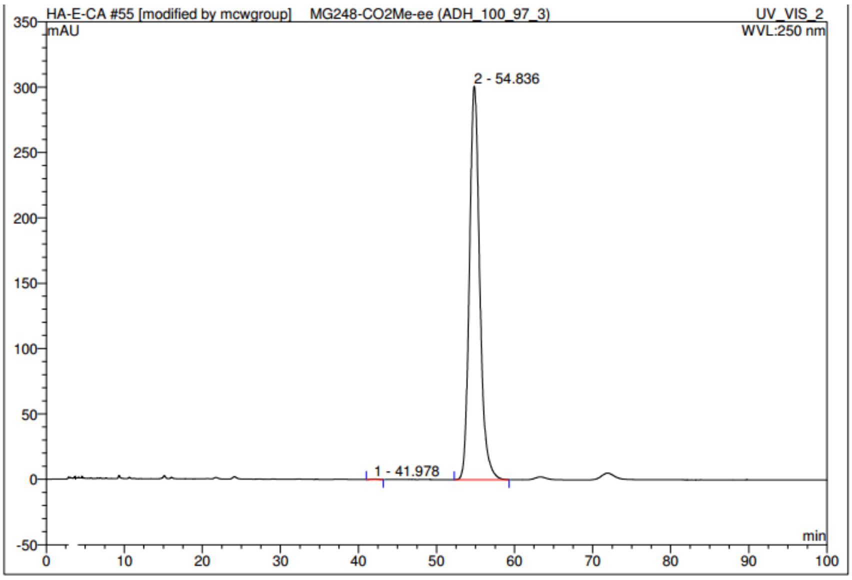

No.	Ret.Time min	Peak Name	Height mAU	Area mAU*min	Rel.Area %	Amount	Type
1	41.98	n.a.	0.265	0.273	0.06	n.a.	BMB*
2	54.84	n.a.	301.031	460.866	99.94	n.a.	BMB*
Total:			301.296	461.139	100.00	0.000	

(*S*)-6-[2-(4-Methoxyphenyl)heptyl]-2,3,4,5-tetrahydropyridine (6a)

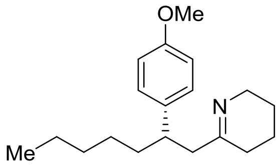

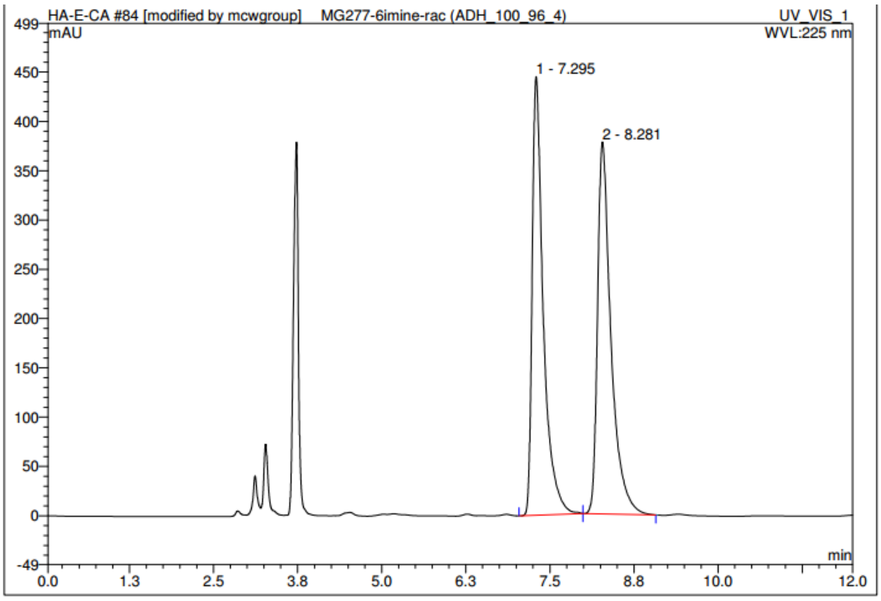

No.	Ret.Time min	Peak Name	Height mAU	Area mAU*min	Rel.Area %	Amount	Type
1	7.30	n.a.	444.914	85.202	49.37	n.a.	BMb*
2	8.28	n.a.	377.345	87.388	50.63	n.a.	bMB*
Total:			822.259	172.590	100.00	0.000	

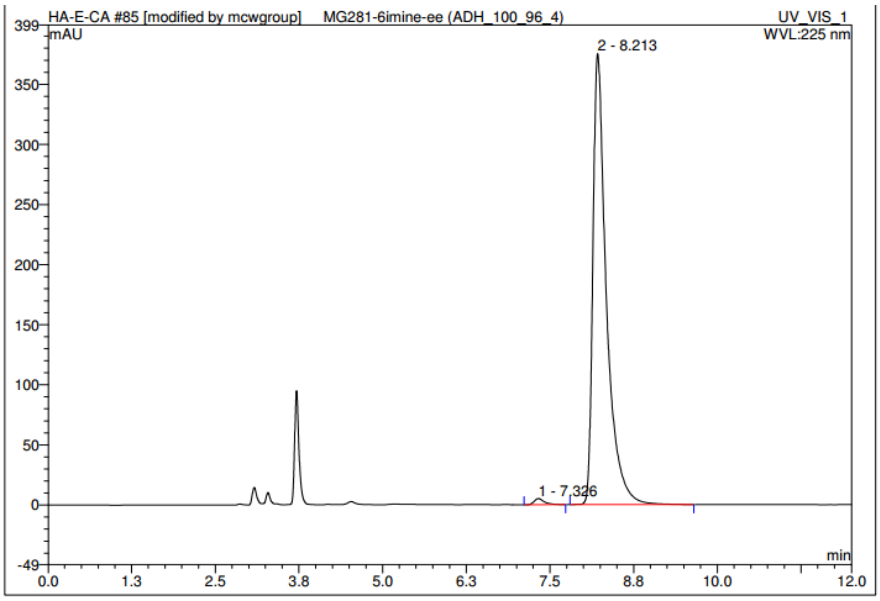

No.	Ret.Time min	Peak Name	Height mAU	Area mAU*min	Rel.Area %	Amount	Type
1	7.33	n.a.	5.144	0.922	1.04	n.a.	BMB*
2	8.21	n.a.	375.128	87.835	98.96	n.a.	BMB*
Total:			380.271	88.757	100.00	0.000	

(*S*)-3-(4-Methoxyphenyl)-5-[(*S*)-2-(4-methoxyphenyl)heptyl]-3,4-dihydro-2*H*-pyrrole (6b)

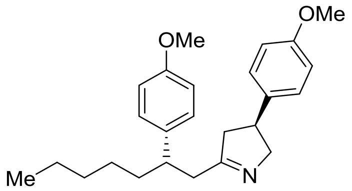

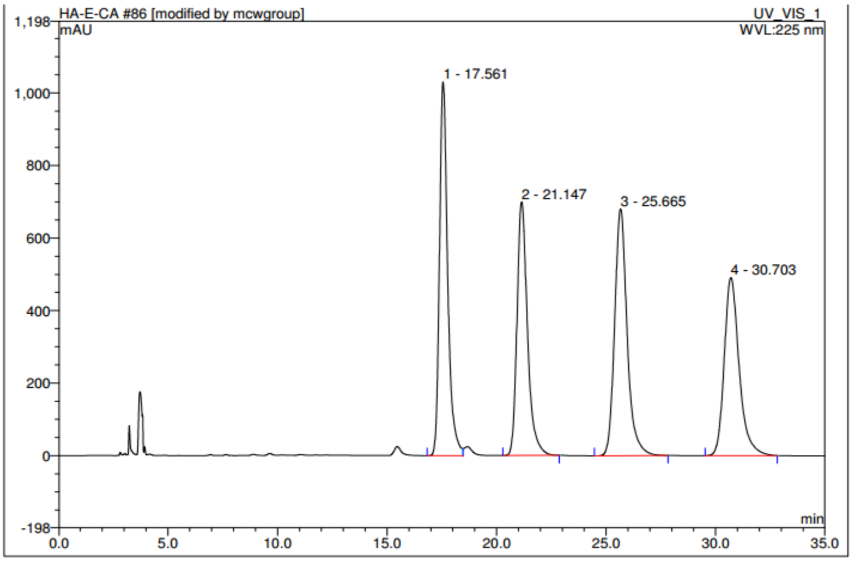

No.	Ret.Time min	Peak Name	Height mAU	Area mAU*min	Rel.Area %	Amount	Type
1	17.56	n.a.	1030.978	448.917	26.83	n.a.	BM *
2	21.15	n.a.	699.532	386.605	23.11	n.a.	BMB*
3	25.67	n.a.	680.634	450.044	26.90	n.a.	BMB*
4	30.70	n.a.	491.355	387.515	23.16	n.a.	BMB*
Total:			2902.498	1673.082	100.00	0.000	

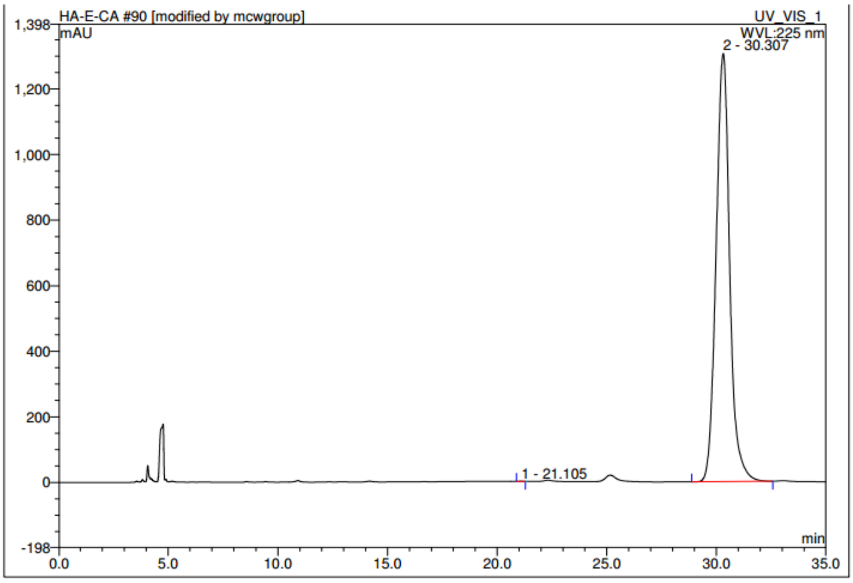

No.	Ret.Time min	Peak Name	Height mAU	Area mAU*min	Rel.Area %	Amount	Type
1	21.11	n.a.	0.258	0.067	0.01	n.a.	BMB*
2	30.31	n.a.	1305.632	939.715	99.99	n.a.	BMB*
Total:			1305.890	939.782	100.00	0.000	

6-[(4*S*)-2-Methoxy-4-pentylchroman-2-yl]hexan-2-one (6d)

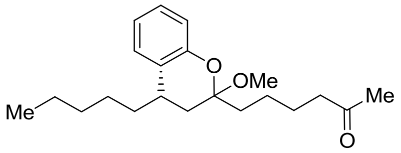

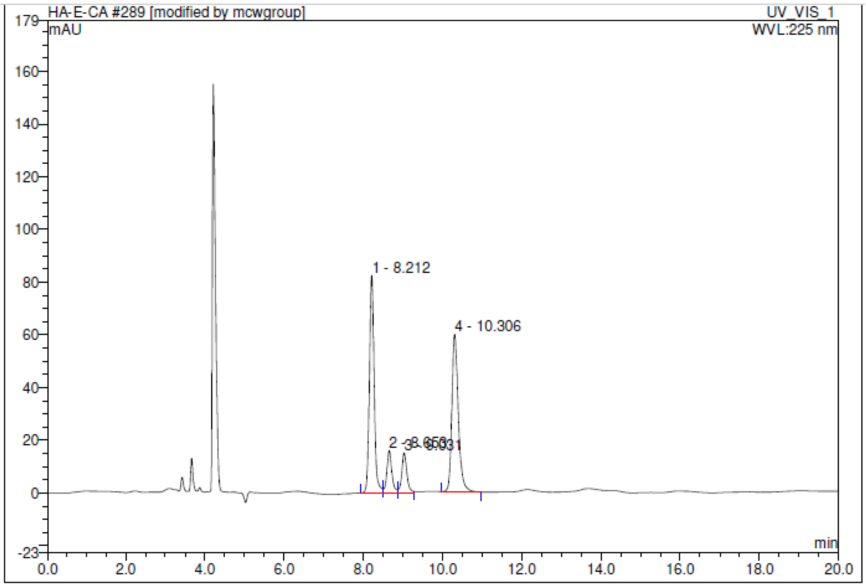

No.	Ret.Time min	Peak Name	Height mAU	Area mAU*min	Rel.Area %	Amount	Type
1	8.21	n.a.	82.500	11.442	41.57	n.a.	BM *
2	8.65	n.a.	16.129	2.358	8.57	n.a.	M *
3	9.03	n.a.	15.251	2.323	8.44	n.a.	M *
4	10.31	n.a.	59.857	11.401	41.42	n.a.	MB*

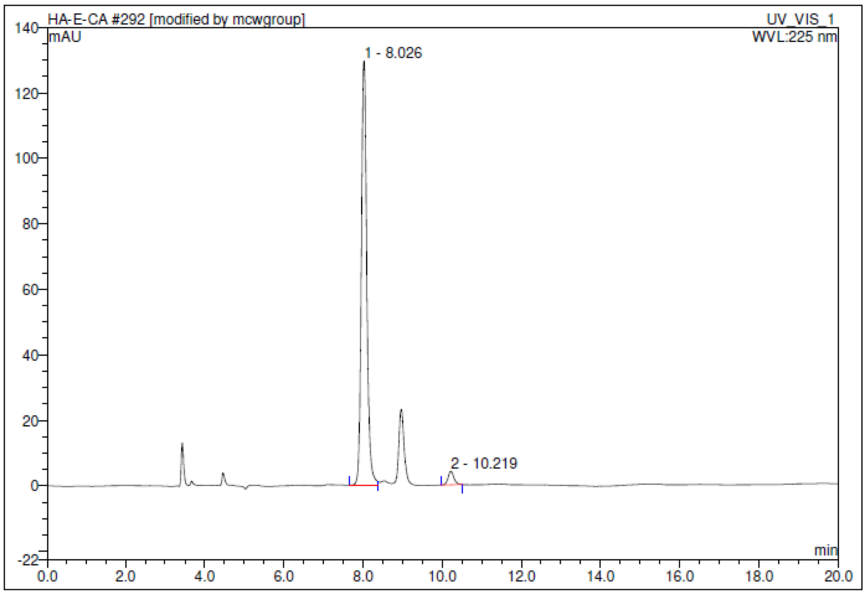

No.	Ret.Time min	Peak Name	Height mAU	Area mAU*min	Rel.Area %	Amount	Type
1	8.03	n.a.	129.468	20.630	96.70	n.a.	BM *
2	10.22	n.a.	4.120	0.704	3.30	n.a.	BMB*
Total:			133.587	21.334	100.00	0.000	

(2*R*,4*S*)-4-Pentyl-2-(4-phenylbutyl)chroman (6e)

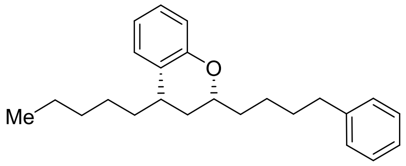

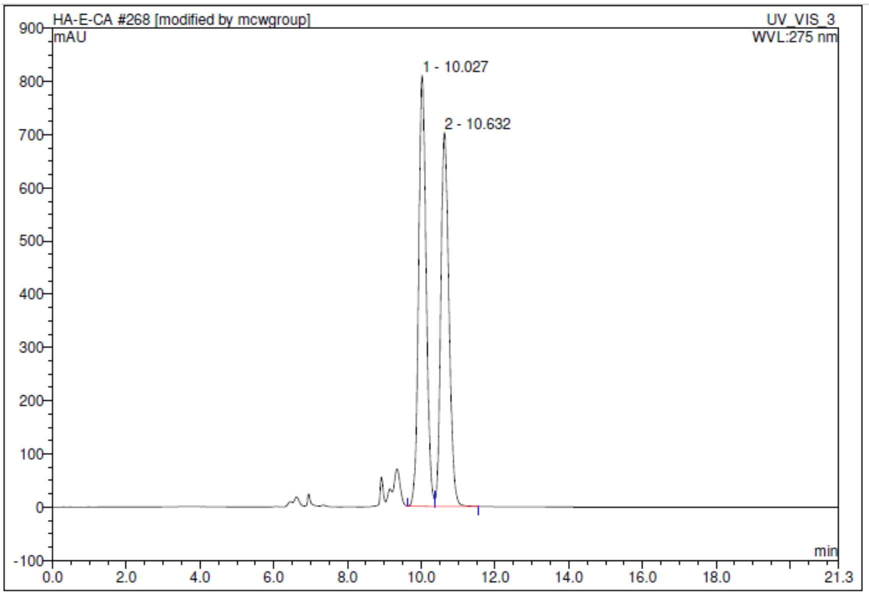

No.	Ret.Time min	Peak Name	Height mAU	Area mAU*min	Rel.Area %	Amount	Type
1	10.03	n.a.	810.235	192.607	51.69	n.a.	BM *
2	10.63	n.a.	703.077	179.989	48.31	n.a.	MB*
Total:			1513.312	372.596	100.00	0.000	

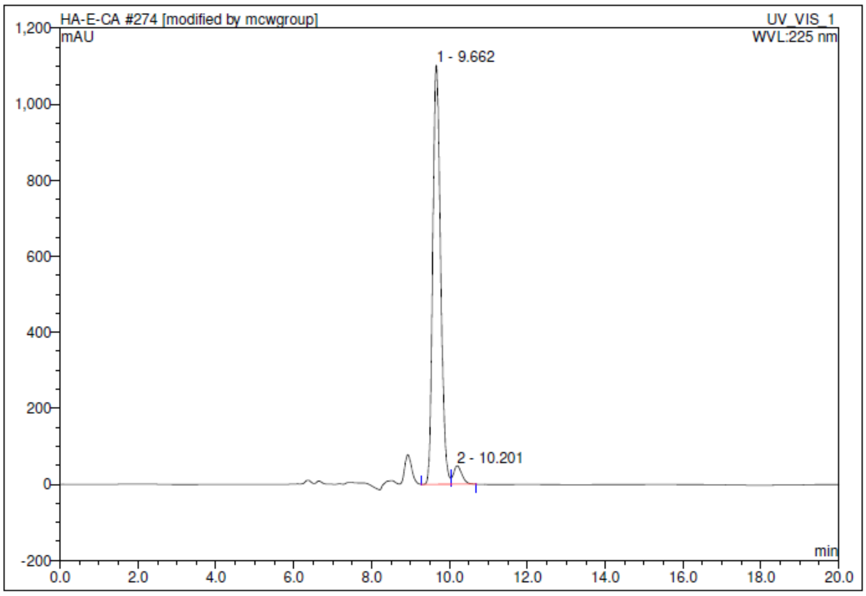

No.	Ret.Time min	Peak Name	Height mAU	Area mAU*min	Rel.Area %	Amount	Type
1	9.66	n.a.	1102.603	256.948	95.43	n.a.	BM *
2	10.20	n.a.	48.540	12.296	4.57	n.a.	MB*
Total:			1151.142	269.243	100.00	0.000	

(*S*)-4-Phenylnonan-2-one

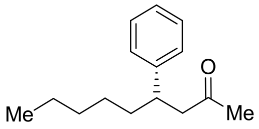

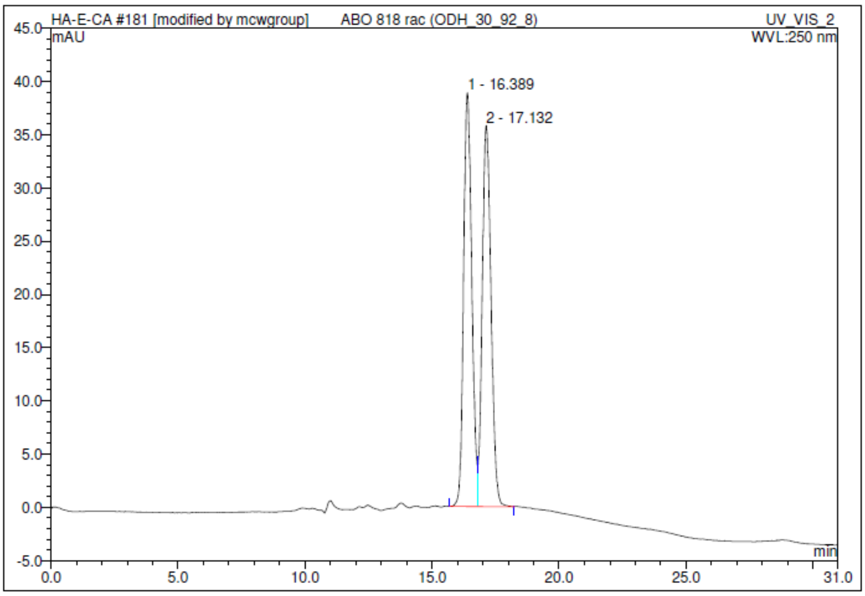

No.	Ret.Time min	Peak Name	Height mAU	Area mAU*min	Rel.Area %	Amount	Type
1	16.39	n.a.	38.872	14.542	50.24	n.a.	BM *
2	17.13	n.a.	35.806	14.403	49.76	n.a.	MB*
Total:			74.677	28.945	100.00	0.000	

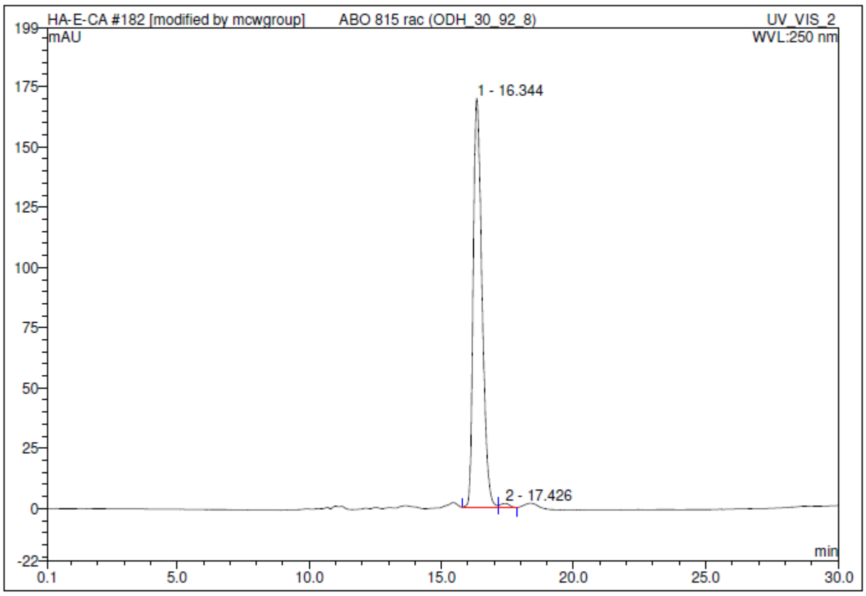

No.	Ret.Time min	Peak Name	Height mAU	Area mAU*min	Rel.Area %	Amount	Type
1	16.34	n.a.	169.604	65.293	98.94	n.a.	M *
2	17.43	n.a.	1.675	0.697	1.06	n.a.	MB*
Total:			171.279	65.990	100.00	0.000	
